# Supplementary figures and images for: Calicivirus assembly and stability are mediated by the N-terminal domain of the capsid protein with the involvement of the viral genome
Source: PLoS Pathog. 2025 Dec 11;21(12):e1013364. doi: 10.1371/journal.ppat.1013364 (PMC12707631; doi:10.1371/journal.ppat.1013364)

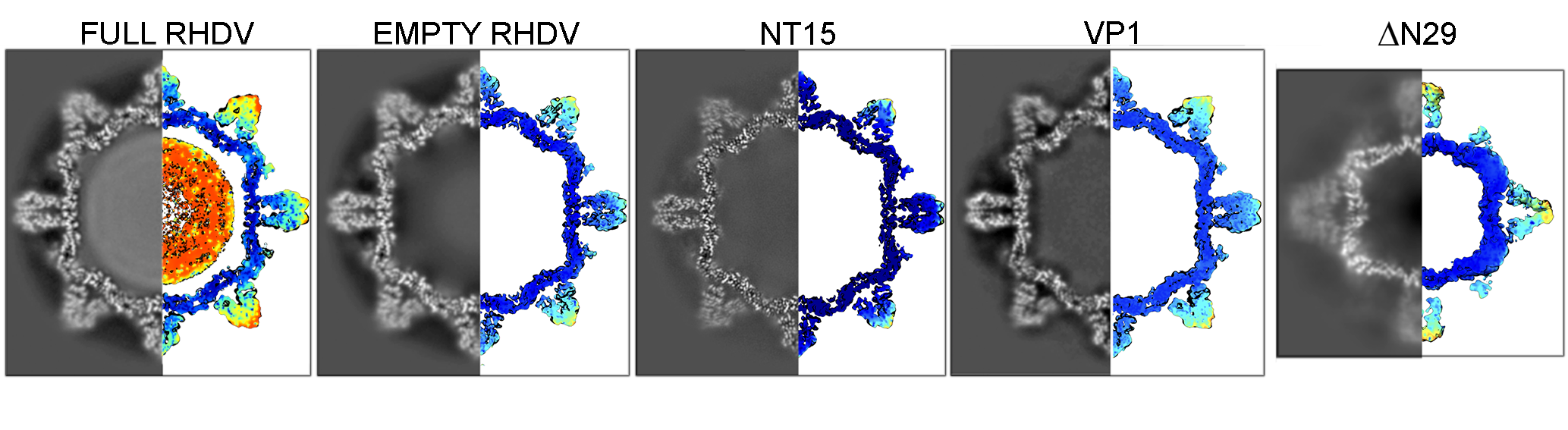

Supplement: S1 Fig — Central sections from the 3DR (left half) and 8 Å–thick slabs (right half) contoured at 1.2σ above the mean density to highlight the RNA density (as shown in Fig 1H–K, lower row). In central sections, brigther shading indicates higher density. The central transverse sections of the 3DRs highlight internal structural differences, particularly emphasizing the density attributable to the genomic RNA in full RHDV virions. (TIF) [file ppat.1013364.s001.tif]

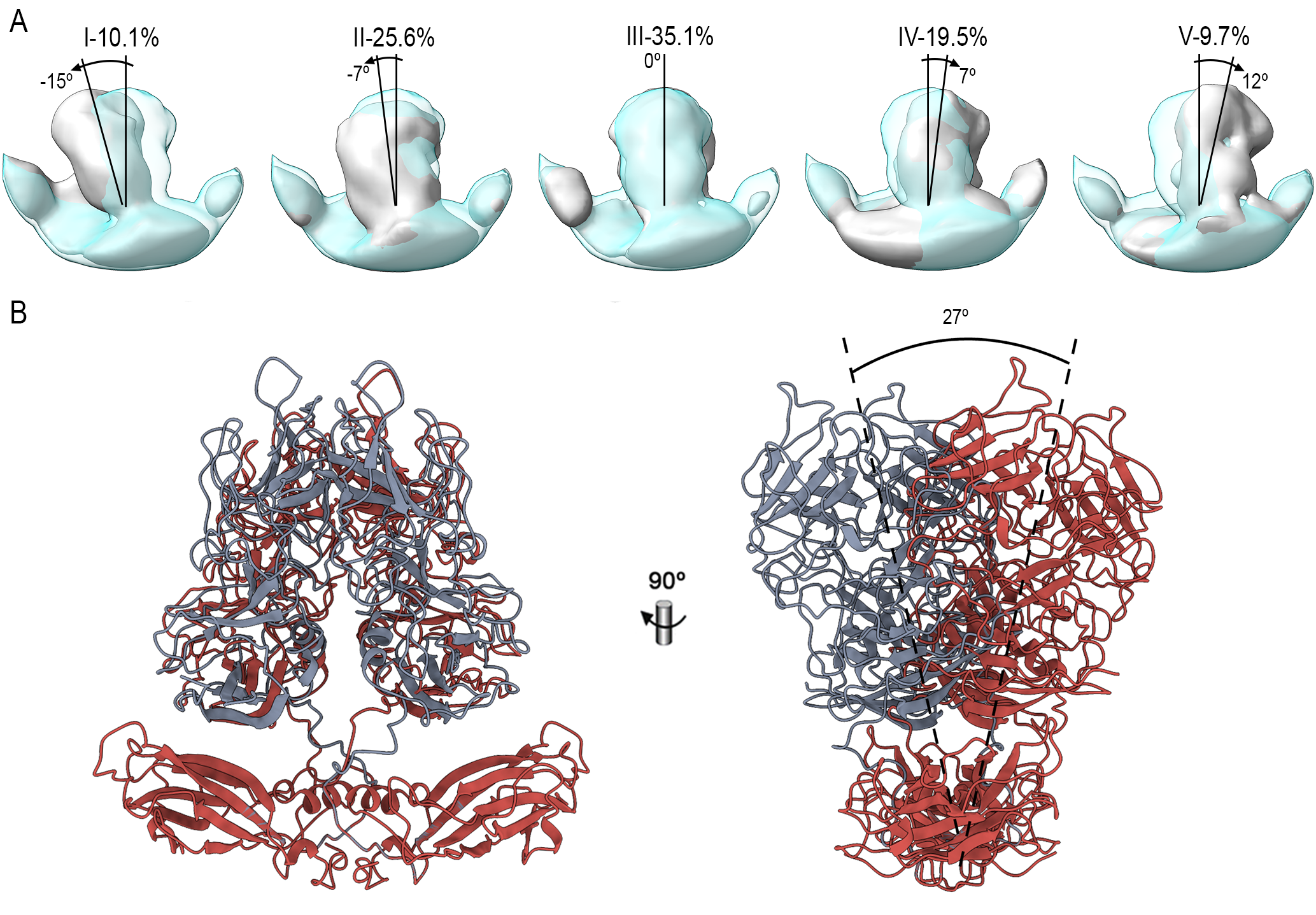

Supplement: S2 Fig — (A) Analysis of Δ29N dimeric spikes leads to five major classes that include 100% of particles (percentages indicated relative to total selected subparticles). The density maps of the different classes (grey) are superimposed with a spike extracted from the Δ29N capsid calculated after imposing icosahedral symmetry (blue transparent surface). Classes I and V show the spikes with the highest swing angles in opposite directions. (B) Superposition of VP1 dimers fitted in the classes I (grey) and V (red). The swing angle between these two VP1 dimers is ~ 27º. (TIF) [file ppat.1013364.s002.tif]

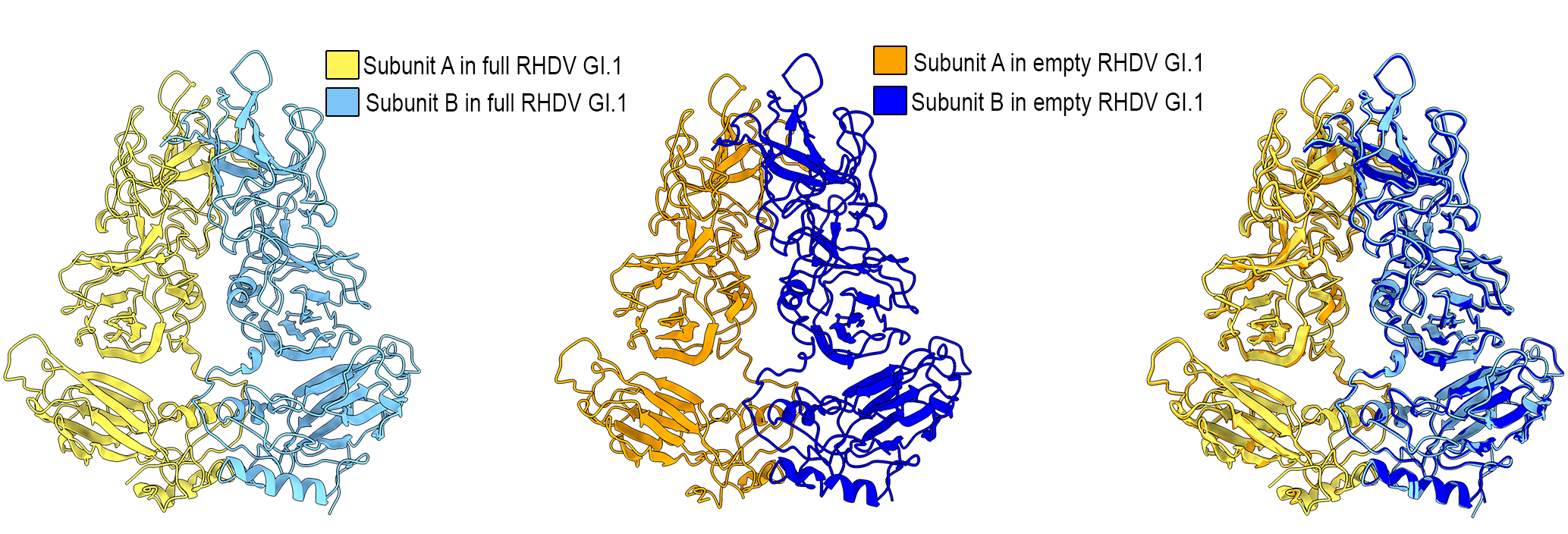

Supplement: S3 Fig — Alignment of S domains of A/B dimers in RHDV GI.1 virions (left) and VLPs (center) showed the superposition of their P domains (right). Whereas the P domain of subunit A adopts an intermediate position between those observed in virions and VLPs in RHDV GI.2 D (displaced by ~4 Å relative to each other), the position of the P domain of subunits B is close to that observed in RHDV GI.2 VLP. (TIF) [file ppat.1013364.s003.tif]

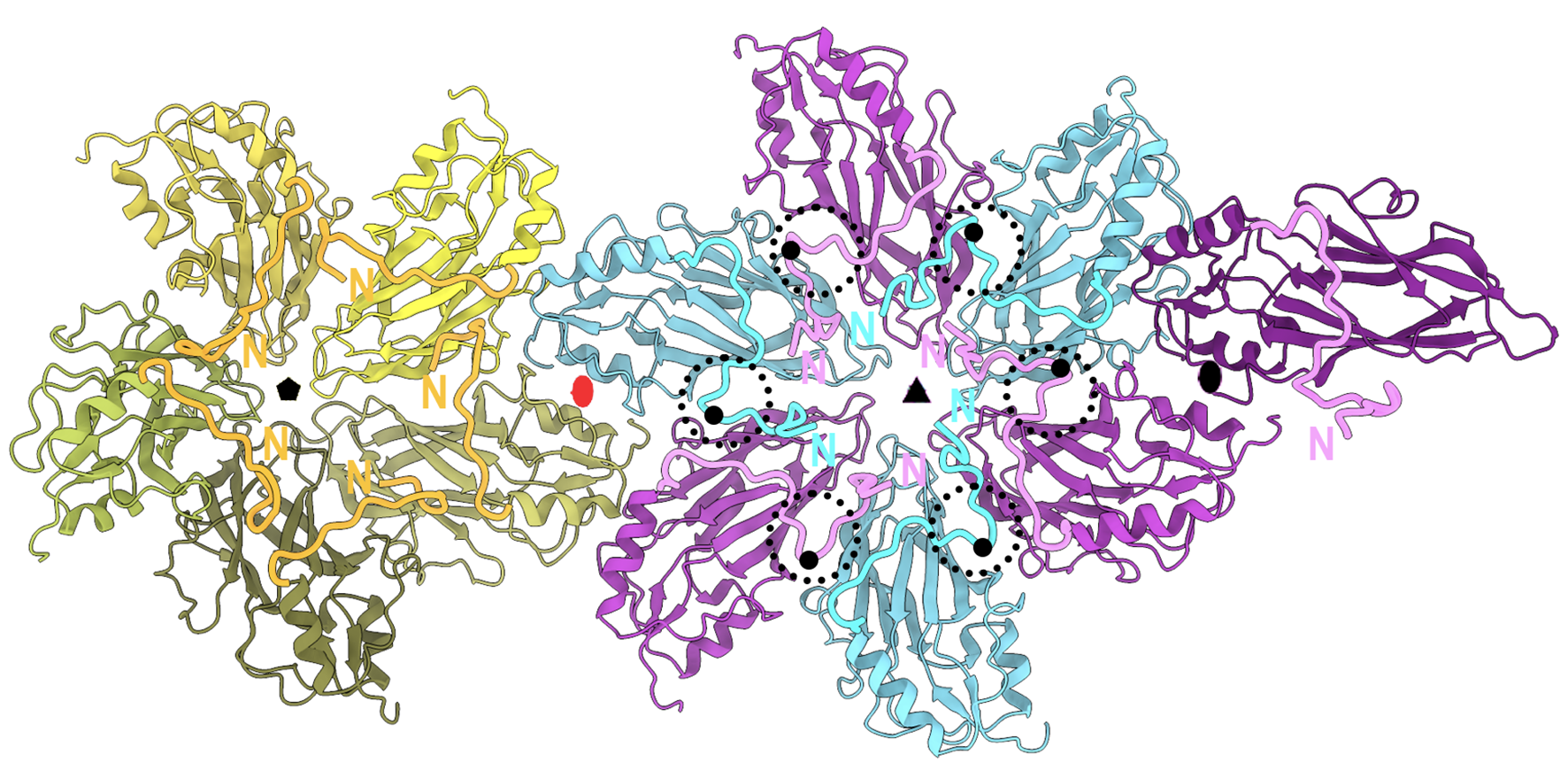

Supplement: S4 Fig — The VP1 pentamer and hexamer lack their P domains and are viewed from inside the capsid (ribbon representations), as in Fig 5B. The A subunit NTA (yellow-green colors) around a fivefold symmetry axis is highlighted in orange. The B and C subunit NTAs are highlighted in bright cyan and pink, respectively, and the Gly29 is shown as a black sphere. The N termini are indicated. The wedge of segment Asp28-Asp31 of B and C subunits is highlighted (black dashed circles). Icosahedral symmetry axes (black symbols) and a local twofold axis (red oval) are indicated. (TIF) [file ppat.1013364.s004.tif]

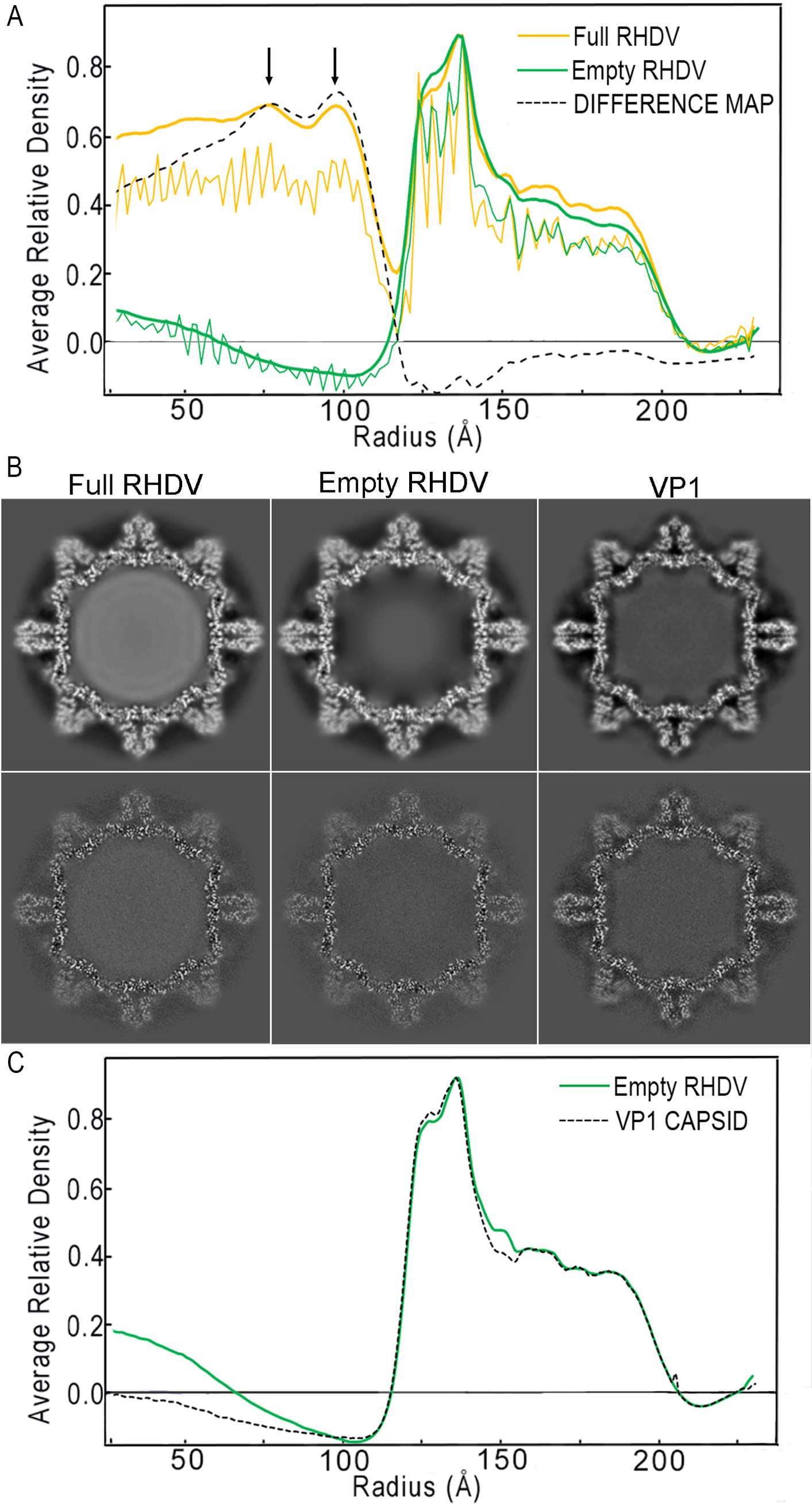

Supplement: S5 Fig — (A) Comparison of full and empty RHDV. Protein shells are assentially superimposable. RNA is located at radii <113Å. The density inside the capsid that corresponds to the packaged ssRNA genome is seen in the radial density profiles from both unsharpened (yellow thick lines) and sharpened (yellow thin lines) 3D maps of full particles. The genome in full RHDV appears as (at least) two concentric shells of density at radii of ~77 and ~98 Å (arrows). In the empty RHDV, the internal density was similar to that of the external solvent (green thick and thin lines for unsharpened and sharpened maps, respectively). The calculated difference map (full subtracted from empty capsid) showed the genome densities (dashed line). (B) Central sections from the 3DR of full and empty RHDV, and VP1 capsid viewed along a twofold axis of symmetry. Upper row, unsharpened central sections; lower row, sharpened central sections. (C) Comparison of empty RHDV and VP1 capsid to show that both profiles are superimposable at the protein shell. (TIF) [file ppat.1013364.s005.tif]

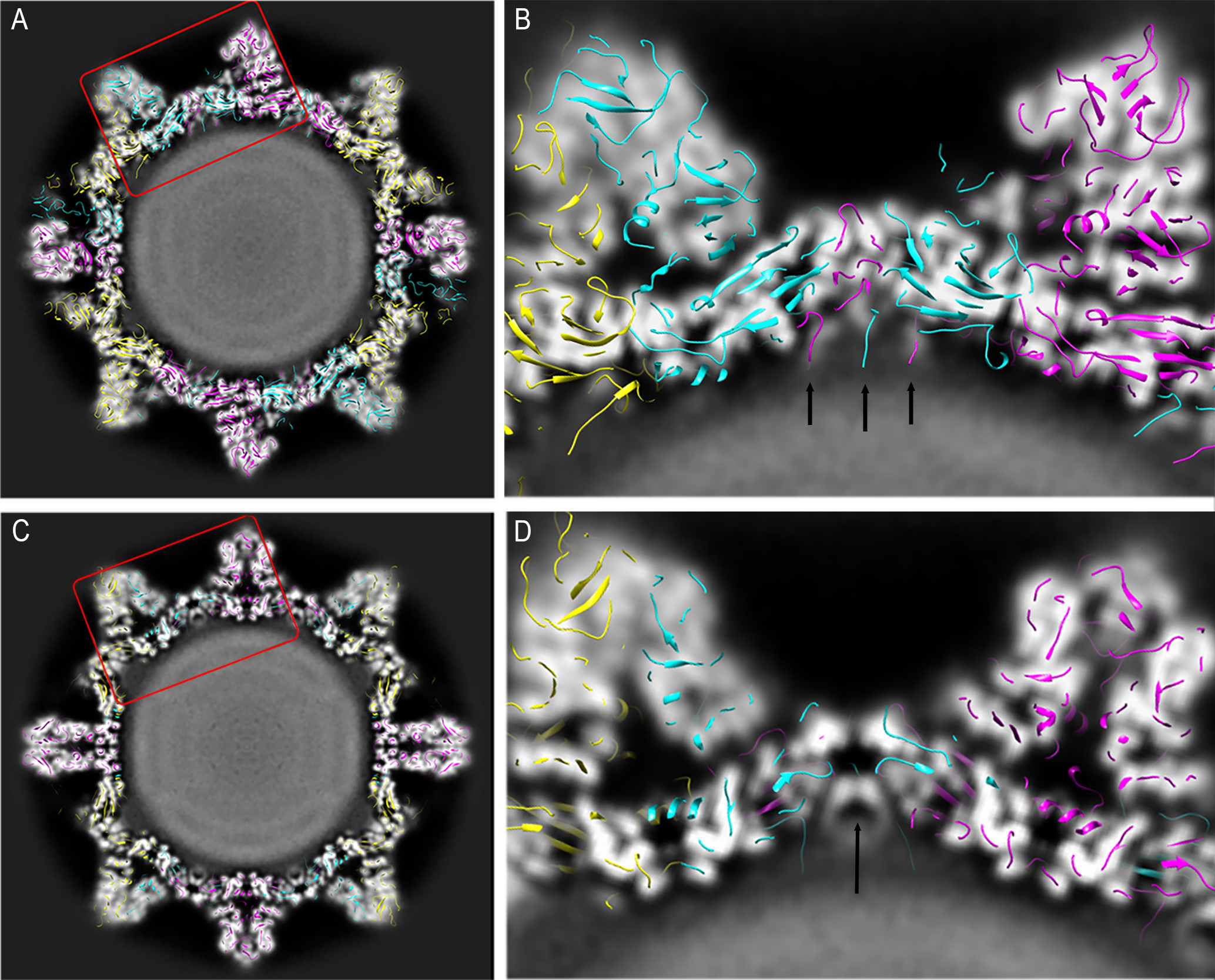

Supplement: S6 Fig — (A) Central section taken from the full RHDV 3DR, parallel to but displaced ~8 Å from the central section viewed along a twofold axis (protein and genome are white). The atomic models of VP1(A, yellow; B, cyan, C, pink) are superimposed on the protein density (white). (B) Magnified view of the box in A. The first visible residue of VP1 for B (cyan) and C (pink), Ala20, is indicated in three VP1 molecules (arrows). (C) Central section taken from the full RHDV 3DR shown as in A. (D) Magnified view of the box in C. The jellyfish-like density is indicated. (TIF) [file ppat.1013364.s006.tif]

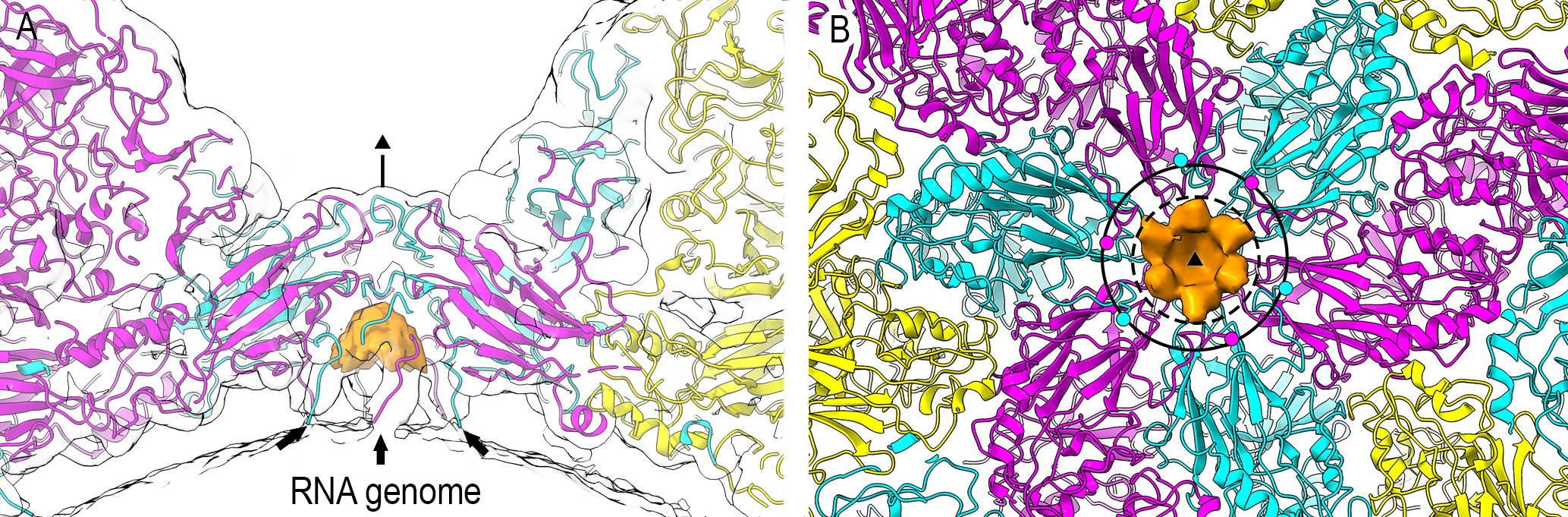

Supplement: S7 Fig — (A) ~50-Å-thick section viewed around an icosahedral threefold axis (side view) of the RHDV virion. A, B, and C subunits are shown in yellow, cyan and pink, respectively. Capsid-viral genome contacts mediated by N termini of B and C subunits’ NTA are indicated (arrows). The uninterpreted, jellyfish-like cryo-EM density observed at the three-fold axis in the RHDV virion is highlighted in orange. The icosahedral threefold axis is indicated by a black triangle. (B) Inside view of the region shown in (A) indicating the radii of 11 (dashed circle) and 15 Å (solid circle) at which the jellyfish-like arms and NTAs are located, respectively. The last residues of the NTAs of B and C subunits are shown as cyan and pink spheres, respectively. (TIF) [file ppat.1013364.s007.tif]

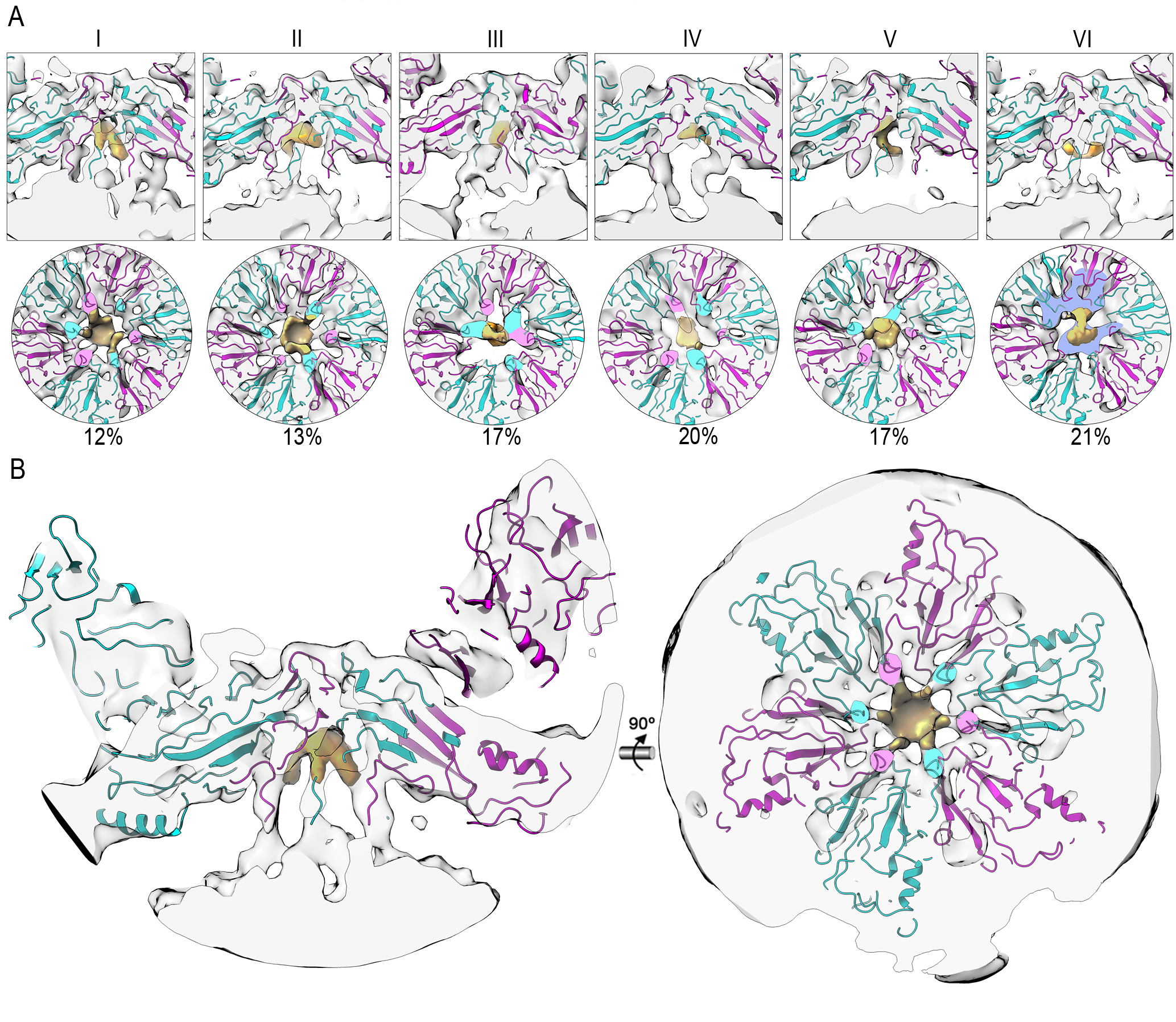

Supplement: S8 Fig — (A) 3D cryo-EM class averages of the region around the threefold axis of the full RHDV capsid, reconstructed without symmetry. Upper row, ~ 30-Å-thick slabs (side views) of the six asymmetric maps (I-VI) obtained after symmetry relaxation of the hexameric regions. Lower row, inside view of the same maps, ~ 30-Å-thick slabs, in which RNA density is not shown for clarity. The contact surfaces between the NTAs of the B and C subunits and the RNA genome density are shown in cyan and pink, respectively. These six classes show arrangements with 6 (classes I and II), 5 (III and IV) and 4 (V) NTA-mediated connections; class VI displays an undefined connection (shown in blue). Classes I-VI made up 12, 13, 17, 20, 17 and 21% of the total subparticles, respectively. The six class averages are contoured at 1σ above the mean density. (B) A ~ 30-Å-thick slab of the asymmetric map calculated by merging and refining classes I-IV obtained by symmetry relaxation of the hexameric regions in the full RHDV capsid (left, side view; right, inside view). The map is contoured at 1σ above the mean density. The colors of the map are as in panel A. (TIF) [file ppat.1013364.s008.tif]
