# Supplementary material for: Calicivirus assembly and stability are mediated by the N-terminal domain of the capsid protein with the involvement of the viral genome
Source: PLoS Pathog. 2025 Dec 11;21(12):e1013364. doi: 10.1371/journal.ppat.1013364 (PMC12707631; doi:10.1371/journal.ppat.1013364)
Supplement: S1 Table — (DOCX) [file ppat.1013364.s016.docx]

|  | **Full RHDV**  **EMD-52757**  **PDB: 9I9D** | **Empty RHDV**  **EMD-52747**  **PDB: 9I90** | **N15**  **EMD-52593**  **PDB: 9I3H** | **VP1**  **EMD-52733**  **PDB: 9I8R** | **Δ29N**  **EMD-52588**  **PDB: 9I3E** | **A/B Pdomain**  **EMD-52727**  **PDB: 9I8J** |
| --- | --- | --- | --- | --- | --- | --- |
| **Data collection and processing** |  |  |  |  |  |  |
| Microscope | TFS Talos Arctica | TFS Talos Arctica | FEI Titan  Krios | FEI Titan Krios | TFS Talos Arctica | FEI Titan Krios |
| Detector | Falcon III | Falcon III | K2 | Falcon III | Falcon III | K2 |
| Magnification | 73,000x | 73,000x | 105,000x | 81,000x | 73,000x | 105,000x |
| Voltage (kV) | 200 | 200 | 300 | 300 | 200 | 300 |
| Electron exposure (e^-^/Å^2^) | 29.7 | 29.7 | 32 | 53 | 31.9 | 32 |
| Exposure per frame (e^-^/Å^2^) | 0.33 | 0.33 | 1 | 1 | 0.54 | 1 |
| Defocus range (µm) | -0.8 to -2.3 | -0.8 to -2.3 | -0.8 to -2.3 | -0.8 to -2.3 | -0.8 to -2.3 | -0.8 to -2.3 |
| Pixel size (Å) | 1.37 | 1.37 | 1.08 | 1.08 | 1.37 | 1.08 |
| Micrographs collected (no.) | 2,824 | 2,824 | 3,580 | 5,515 | 4,860 | 3,580 |
| Initial particles (no.) | 230,852 | 230,852 | 124,519 | 166,047 | 389,808 | 3,456,780 |
| Final particles (no.) | 25,237 | 141,193 | 50,616 | 43,950 | 302,069 | 164,061 |
| Symmetry imposed | I2 | I2 | I2 | I2 | I2 | C1 |
| Map resolution (Å) | 3.3 | 3.2 | 2.5 | 3.3 | 3.0 | 2.9 |
| FSC threshold | 0.143 | 0.143 | 0.143 | 0.143 | 0.143 | 0.143 |
| **Refinement** |  |  |  |  |  |  |
| Mask correlation coefficient | 0.74 | 0.75 | 0.68 | 0.78 | 0.84 | 0.65 |
| Map sharpening B factor | Cryosparc | Cryosparc | Cryosparc | Cryosparc | Cryosparc | LocalDeblur |
| Model composition |  |  |  |  |  |  |
| Non-hydrogen atoms | 727,740 | 723,540 | 729,240 | 723,540 | 79,740 | 4,958 |
| Protein residues | 98,700 | 98,040 | 98,760 | 98,040 | 10,440 | 678 |
| ADP (B-factors) |  |  |  |  |  |  |
| min | 0.00 | 0.00 | 0.57 | 0.00 | 6.97 | 15.10 |
| max | 174.85 | 159.10 | 182.19 | 148.31 | 75.60 | 148.31 |
| mean | 61.87 | 34.75 | 55.76 | 40.40 | 32.71 | 66.50 |
| R.m.s. deviations |  |  |  |  |  |  |
| Bond lengths (Å) | 0.007 | 0.006 | 0.004 | 0.008 | 0.006 | 0.006 |
| Bond angles (˚) | 1.300 | 1.223 | 0.857 | 1.325 | 1.221 | 1.097 |
| Validation |  |  |  |  |  |  |
| MolProbity score | 2.42 | 1.88 | 2.02 | 2.40 | 2.35 | 2.12 |
| Clashscore | 9.68 | 3.16 | 3.95 | 6.02 | 5.12 | 1.85 |
| Rotamer outliers (%) | 3.93 | 3.08 | 3.17 | 5.88 | 5.91 | 6.82 |
| Ramachandran plot |  |  |  |  |  |  |
| Favored (%) | 92.84 | 94.10 | 92.80 | 91.94 | 92.00 | 90.37 |
| Allowed (%) | 6.73 | 5,72 | 6.89 | 7.69 | 6.82 | 9.33 |
| Outliers (%) | 0.43 | 0.18 | 0.31 | 0.37 | 1.18 | 0.30 |

**S1 Table. Cryo-EM data collection and refinement statistics.**
