# Supplementary material for: Calicivirus assembly and stability are mediated by the N-terminal domain of the capsid protein with the involvement of the viral genome
Source: PLoS Pathog. 2025 Dec 11;21(12):e1013364. doi: 10.1371/journal.ppat.1013364 (PMC12707631; doi:10.1371/journal.ppat.1013364)
Supplement: S1 File — (PDF) [file ppat.1013364.s017.pdf]

Preliminary Full wwPDB EM Validation Report (i)

Feb 6, 2025 – 01:29 pm GMT

Deposition ID : D_1292144900

This wwPDB validation report is NOT for manuscript review

This is a Preliminary Full wwPDB EM Validation Report.

This report is produced by the wwPDB Deposition System during initial deposition but before annotation of the structure.

We welcome your comments at *validation@mail.wwpdb.org*
A user guide is available at
https://www.wwpdb.org/validation/2017/EMValidationReportHelp
with specific help available everywhere you see the (i) symbol.

The types of validation reports are described at
http://www.wwpdb.org/validation/2017/FAQs#types.

Not For Manuscript Review

The following versions of software and data (see references (i)) were used in the production of this report:

EMDB validation analysis	:	0.0.1.dev113
MolProbity	:	4.02b-467
Percentile statistics	:	20231227.v01 (using entries in the PDB archive December 27th 2023)
MapQ	:	1.9.13
Ideal geometry (proteins)	:	Engh & Huber (2001)
Ideal geometry (DNA, RNA)	:	Parkinson et al. (1996)
Validation Pipeline (wwPDB-VP)	:	2.40

1 Overall quality at a glance (i)

The following experimental techniques were used to determine the structure:
ELECTRON MICROSCOPY

The reported resolution of this entry is 3.30 Å.

Percentile scores (ranging between 0-100) for global validation metrics of the entry are shown in the following graphic. The table shows the number of entries on which the scores are based.

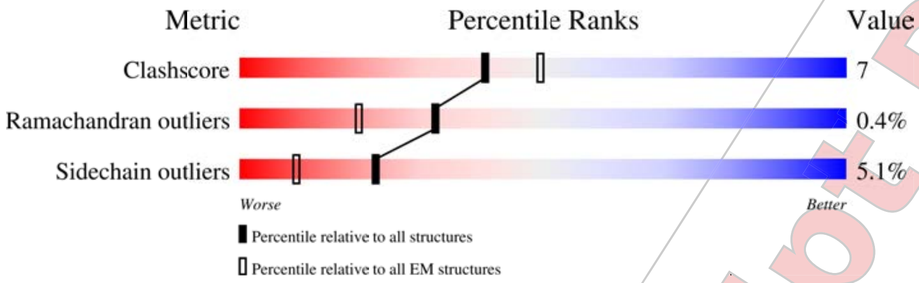

Metric	Whole archive (#Entries)	EM structures (#Entries)
Clashscore	210492	15764
Ramachandran outliers	207382	16835
Sidechain outliers	206894	16415

The table below summarises the geometric issues observed across the polymeric chains and their fit to the map. The red, orange, yellow and green segments of the bar indicate the fraction of residues that contain outliers for >=3, 2, 1 and 0 types of geometric quality criteria respectively. A grey segment represents the fraction of residues that are not modelled. The numeric value for each fraction is indicated below the corresponding segment, with a dot representing fractions <=5% The upper red bar (where present) indicates the fraction of residues that have poor fit to the EM map (all-atom inclusion < 40%). The numeric value is given above the bar.

Mol	Chain	Length	Quality of chain
1	A	542	20% / 83% 15% •
2	B	551	25% / 85% 14% •
3	C	551	• / 86% 13% •

Not For Manuscript Review

2 Entry composition (i)

There are 3 unique types of molecules in this entry. The entry contains 12123 atoms, of which 0 are hydrogens and 0 are deuteriums.

In the tables below, the AltConf column contains the number of residues with at least one atom in alternate conformation and the Trace column contains the number of residues modelled with at most 2 atoms.

- Molecule 1 is a protein called Capsid protein (A).

Mol	Chain	Residues	Atoms					AltConf	Trace
			Total	C	N	O	S		
1	A	542	4002	2541	677	772	12	0	0

- Molecule 2 is a protein called Capsid protein (B).

Mol	Chain	Residues	Atoms					AltConf	Trace
			Total	C	N	O	S		
2	B	551	4060	2575	686	787	12	0	0

- Molecule 3 is a protein called Capsid protein (C).

Mol	Chain	Residues	Atoms					AltConf	Trace
			Total	C	N	O	S		
3	C	551	4061	2575	686	788	12	0	0

Not For Manuscript Review

3 Residue-property plots ⓘ

These plots are drawn for all protein, RNA, DNA and oligosaccharide chains in the entry. The first graphic for a chain summarises the proportions of the various outlier classes displayed in the second graphic. The second graphic shows the sequence view annotated by issues in geometry and atom inclusion in map density. Residues are color-coded according to the number of geometric quality criteria for which they contain at least one outlier: green = 0, yellow = 1, orange = 2 and red = 3 or more. A red diamond above a residue indicates a poor fit to the EM map for this residue (all-atom inclusion < 40%). Stretches of 2 or more consecutive residues without any outlier are shown as a green connector. Residues present in the sample, but not in the model, are shown in grey.

- Molecule 1: Capsid protein (A)

Chain A:

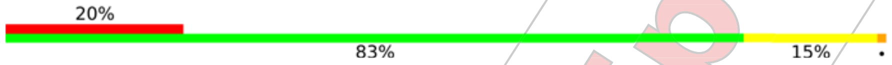

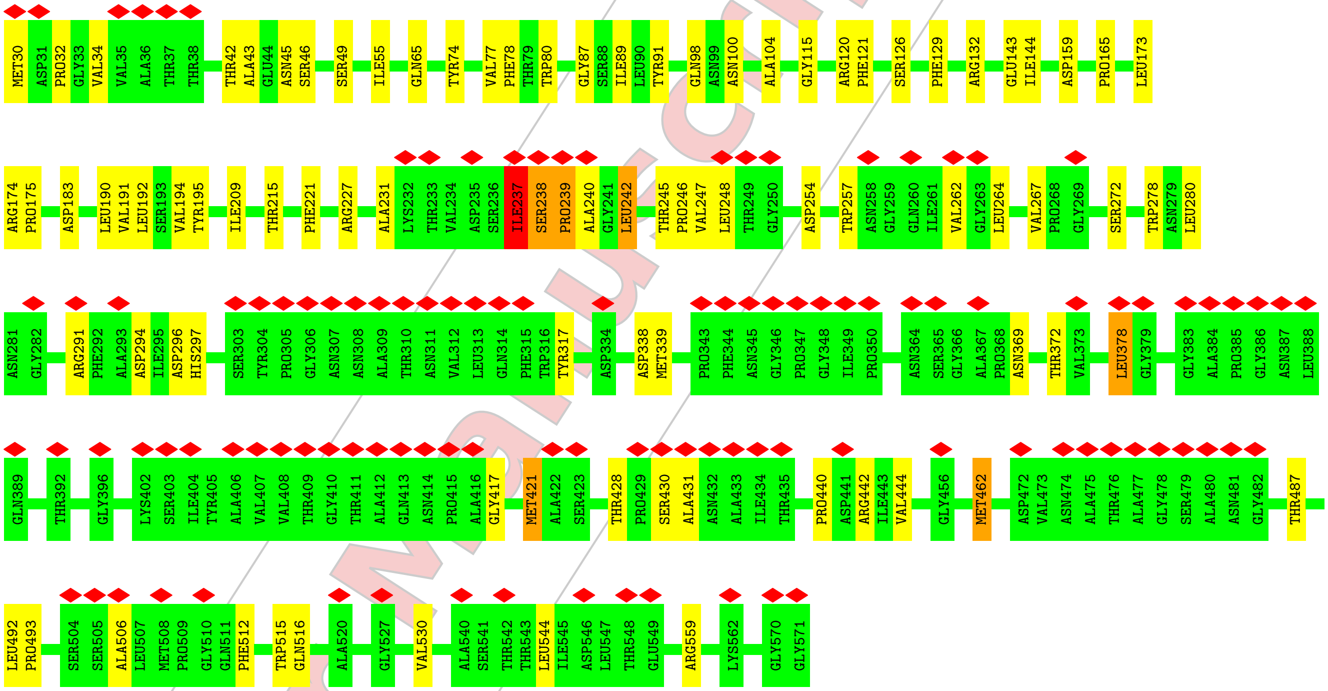

- Molecule 2: Capsid protein (B)

Chain B:

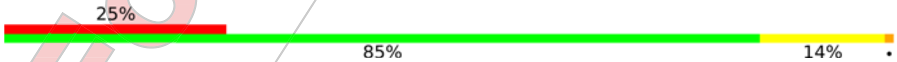

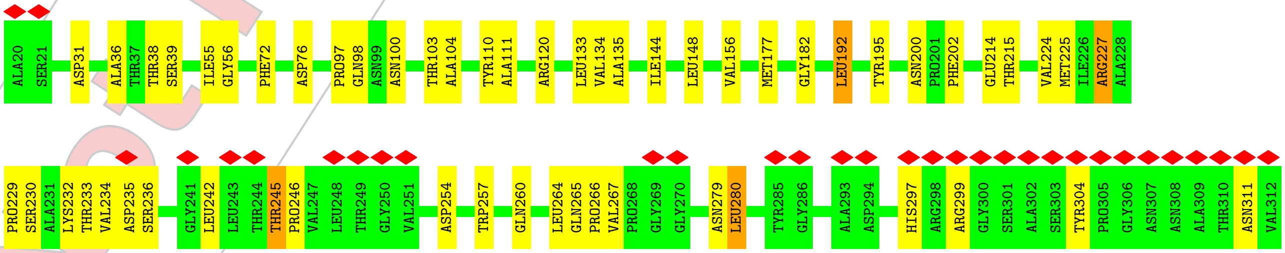

Not For Manuscript Review

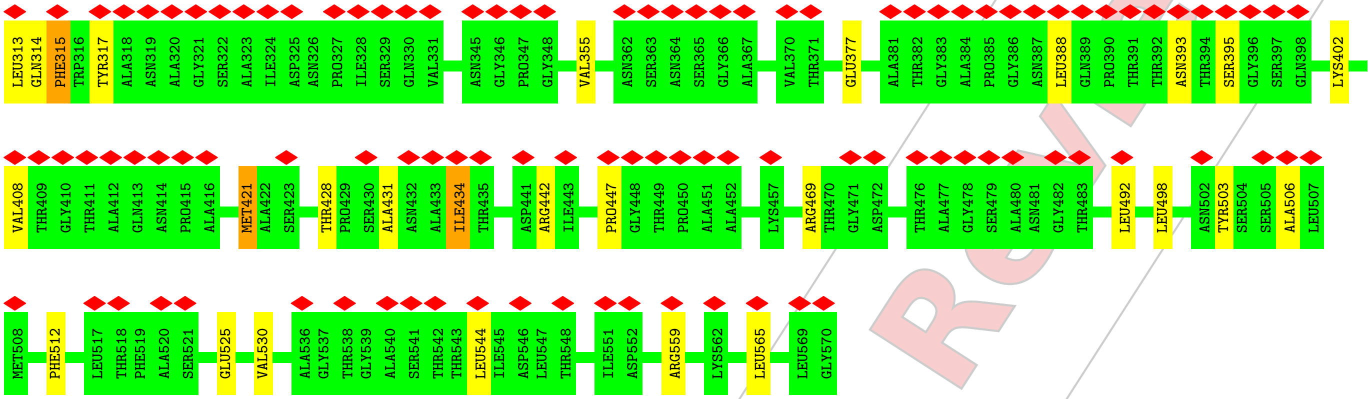

• Molecule 3: Capsid protein (C)

Chain C:
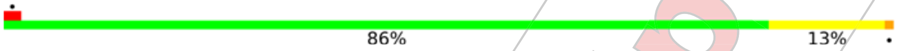

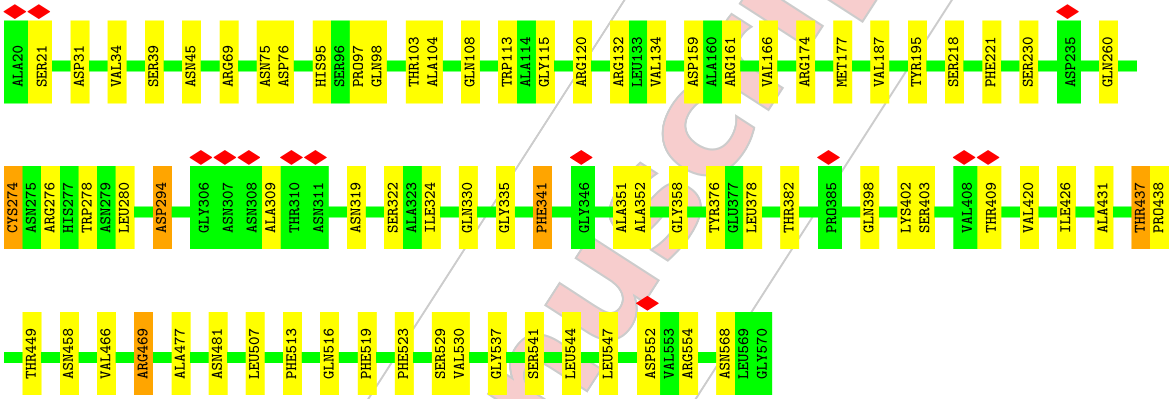

Not For Manuscript Review

4 Experimental information ⓘ

Property	Value	Source
EM reconstruction method	SINGLE PARTICLE	Depositor
Imposed symmetry	POINT, Not provided	
Number of particles used	25237	Depositor
Resolution determination method	FSC 0.143 CUT-OFF	Depositor
CTF correction method	PHASE FLIPPING AND AMPLITUDE CORRECTION	Depositor
Microscope	TFS TALOS	Depositor
Voltage (kV)	200	Depositor
Electron dose $(e^-/\text{Å}^2)$	29.7	Depositor
Minimum defocus (nm)	800	Depositor
Maximum defocus (nm)	2300	Depositor
Magnification	Not provided	
Image detector	FEI FALCON III (4k x 4k)	Depositor
Maximum map value	6.785	Depositor
Minimum map value	-3.194	Depositor
Average map value	0.080	Depositor
Map value standard deviation	0.434	Depositor
Recommended contour level	0.55	Depositor
Map size (Å)	452.1, 452.1, 452.1	wwPDB
Map dimensions	330, 330, 330	wwPDB
Map angles (°)	90.0, 90.0, 90.0	wwPDB
Pixel spacing (Å)	1.37, 1.37, 1.37	Depositor

Not For Manuscript Review

5 Model quality (i)

5.1 Standard geometry (i)

The Z score for a bond length (or angle) is the number of standard deviations the observed value is removed from the expected value. A bond length (or angle) with $|Z| > 5$ is considered an outlier worth inspection. RMSZ is the root-mean-square of all Z scores of the bond lengths (or angles).

Mol	Chain	Bond lengths		Bond angles	
		RMSZ	#$\|Z\|$ >5	RMSZ	#$\|Z\|$ >5
1	A	0.35	0/4116	0.64	4/5656 (0.1%)
2	B	0.38	0/4175	0.64	2/5739 (0.0%)
3	C	0.34	0/4176	0.59	1/5740 (0.0%)
All	All	0.36	0/12467	0.62	7/17135 (0.0%)

Chiral center outliers are detected by calculating the chiral volume of a chiral center and verifying if the center is modelled as a planar moiety or with the opposite hand.A planarity outlier is detected by checking planarity of atoms in a peptide group, atoms in a mainchain group or atoms of a sidechain that are expected to be planar.

Mol	Chain	#Chirality outliers	#Planarity outliers
1	A	0	1

There are no bond length outliers.

All (7) bond angle outliers are listed below:

Mol	Chain	Res	Type	Atoms	Z	Observed(^{o})	Ideal(^{o})
3	C	552	ASP	CB-CG-OD1	8.05	125.55	118.30
1	A	296	ASP	CB-CG-OD1	6.57	124.22	118.30
2	B	498	LEU	CA-CB-CG	6.01	129.11	115.30
1	A	183	ASP	CB-CG-OD1	5.74	123.46	118.30
2	B	544	LEU	CA-CB-CG	5.60	128.17	115.30
1	A	378	LEU	CA-CB-CG	5.55	128.06	115.30
1	A	242	LEU	CA-CB-CG	5.41	127.75	115.30

There are no chirality outliers.

All (1) planarity outliers are listed below:

Mol	Chain	Res	Type	Group
1	A	237	ILE	Peptide

Not For Manuscript Review

5.2 Too-close contacts (i)

In the following table, the Non-H and H(model) columns list the number of non-hydrogen atoms and hydrogen atoms in the chain respectively. The H(added) column lists the number of hydrogen atoms added and optimized by MolProbity. The Clashes column lists the number of clashes within the asymmetric unit, whereas Symm-Clashes lists symmetry-related clashes.

Mol	Chain	Non-H	H(model)	H(added)	Clashes	Symm-Clashes
1	A	4002	0	3871	52	0
2	B	4060	0	3922	81	0
3	C	4061	0	3925	69	0
All	All	12123	0	11718	163	0

The all-atom clashscore is defined as the number of clashes found per 1000 atoms (including hydrogen atoms). The all-atom clashscore for this structure is 7.

All (163) close contacts within the same asymmetric unit are listed below, sorted by their clash magnitude.

Atom-1	Atom-2	Interatomic distance (Å)	Clash overlap (Å)
2:B:393:ASN:CB	3:C:324:ILE:HD13	1.17	1.55
2:B:393:ASN:CB	3:C:324:ILE:CD1	1.86	1.49
2:B:393:ASN:CG	3:C:324:ILE:HD13	1.35	1.42
2:B:447:PRO:CB	3:C:330:GLN:CG	2.19	1.20
2:B:447:PRO:HB3	3:C:330:GLN:CD	1.65	1.17
2:B:447:PRO:HB2	3:C:330:GLN:CG	1.79	1.12
2:B:447:PRO:HG3	3:C:324:ILE:CG2	1.80	1.10
1:A:77:VAL:O	1:A:559:ARG:NH2	1.85	1.08
2:B:393:ASN:HB2	3:C:324:ILE:CD1	1.77	1.08
2:B:447:PRO:CB	3:C:330:GLN:HG3	1.81	1.07
2:B:447:PRO:HG3	3:C:324:ILE:HG22	1.15	1.07
2:B:447:PRO:CB	3:C:330:GLN:CD	2.25	1.04
2:B:393:ASN:OD1	3:C:324:ILE:HD13	1.58	1.02
2:B:393:ASN:CG	3:C:324:ILE:CD1	2.15	1.01
2:B:393:ASN:HB3	3:C:324:ILE:HD13	1.08	1.01
2:B:447:PRO:CG	3:C:330:GLN:HG3	1.90	1.01
1:A:77:VAL:C	1:A:559:ARG:HH21	1.63	1.01
2:B:393:ASN:HB3	3:C:324:ILE:CD1	1.62	0.99
2:B:447:PRO:CG	3:C:324:ILE:HG22	1.91	0.99
2:B:393:ASN:OD1	3:C:324:ILE:CD1	2.14	0.94
2:B:393:ASN:HB2	3:C:324:ILE:HD12	1.49	0.94
2:B:447:PRO:HB2	3:C:330:GLN:HG2	1.50	0.92
1:A:78:PHE:HB3	1:A:559:ARG:NH2	1.84	0.90
2:B:447:PRO:HG2	3:C:330:GLN:HG3	1.54	0.89

Continued on next page...

Not For Manuscript Review

Continued from previous page...

Atom-1	Atom-2	Interatomic distance (Å)	Clash overlap (Å)
2:B:395:SER:OG	3:C:322:SER:HA	1.76	0.84
2:B:393:ASN:HB3	3:C:324:ILE:HD11	1.60	0.81
1:A:78:PHE:CA	1:A:559:ARG:HH21	1.94	0.80
1:A:78:PHE:CA	1:A:559:ARG:NH2	2.44	0.80
2:B:236:SER:HB3	2:B:503:TYR:HA	1.64	0.79
2:B:393:ASN:CB	3:C:324:ILE:HD12	2.04	0.79
1:A:78:PHE:CB	1:A:559:ARG:NH2	2.47	0.78
2:B:76:ASP:CG	2:B:559:ARG:NH2	2.40	0.75
2:B:447:PRO:HB3	3:C:330:GLN:CG	2.04	0.74
2:B:76:ASP:OD2	2:B:559:ARG:NH2	2.21	0.74
3:C:507:LEU:HD11	3:C:513:PHE:HB2	1.69	0.72
1:A:78:PHE:HA	1:A:559:ARG:NE	2.07	0.69
1:A:78:PHE:HA	1:A:559:ARG:NH2	2.07	0.69
1:A:78:PHE:N	1:A:559:ARG:HH21	1.91	0.68
3:C:378:LEU:HD21	3:C:420:VAL:HG21	1.76	0.67
3:C:134:VAL:HB	3:C:195:TYR:HB2	1.76	0.67
1:A:78:PHE:HA	1:A:559:ARG:HE	1.59	0.67
2:B:236:SER:CB	2:B:503:TYR:HA	2.26	0.66
1:A:78:PHE:HA	1:A:559:ARG:HH21	1.61	0.65
2:B:447:PRO:CB	3:C:330:GLN:NE2	2.59	0.65
2:B:236:SER:HB3	2:B:503:TYR:HD1	1.62	0.64
2:B:395:SER:HB3	3:C:324:ILE:HG23	1.78	0.64
2:B:120:ARG:HB3	2:B:214:GLU:HB2	1.81	0.63
1:A:77:VAL:C	1:A:559:ARG:NH2	2.41	0.62
1:A:78:PHE:HA	1:A:559:ARG:CZ	2.31	0.61
2:B:111:ALA:HB2	2:B:227:ARG:HB3	1.83	0.61
1:A:280:LEU:HD22	1:A:530:VAL:HA	1.83	0.60
2:B:447:PRO:HB2	3:C:330:GLN:NE2	2.17	0.60
2:B:447:PRO:CG	3:C:324:ILE:CG2	2.63	0.60
2:B:236:SER:HB2	2:B:506:ALA:HB2	1.82	0.59
1:A:100:ASN:HD22	1:A:215:THR:HG22	1.67	0.59
2:B:447:PRO:HG3	3:C:324:ILE:HG21	1.82	0.59
2:B:76:ASP:CG	2:B:559:ARG:HH21	2.06	0.58
2:B:355:VAL:HG12	2:B:377:GLU:HB2	1.86	0.58
3:C:75:ASN:O	3:C:554:ARG:NH2	2.36	0.58
1:A:238:SER:O	1:A:239:PRO:C	2.42	0.57
3:C:294:ASP:OD1	3:C:294:ASP:N	2.34	0.57
2:B:428:THR:HG21	2:B:434:ILE:HD13	1.88	0.56
1:A:132:ARG:HB2	1:A:195:TYR:HB3	1.87	0.56
2:B:447:PRO:HB2	3:C:330:GLN:CD	2.06	0.56
3:C:280:LEU:HD12	3:C:530:VAL:HA	1.87	0.56

Continued on next page...

Not For Manuscript Review

Continued from previous page...

Atom-1	Atom-2	Interatomic distance (Å)	Clash overlap (Å)
2:B:38:THR:OG1	2:B:39:SER:N	2.38	0.56
2:B:72:PHE:HB3	2:B:214:GLU:HB3	1.88	0.55
1:A:98:GLN:HA	1:A:104:ALA:HB2	1.88	0.55
2:B:182:GLY:O	2:B:235:ASP:N	2.40	0.55
2:B:311:ASN:H	2:B:408:VAL:HA	1.74	0.53
3:C:437:THR:HG23	3:C:438:PRO:HD3	1.90	0.53
3:C:376:TYR:HA	3:C:403:SER:HA	1.90	0.53
2:B:447:PRO:HB3	3:C:330:GLN:HG3	1.75	0.52
3:C:466:VAL:HG21	3:C:477:ALA:HB2	1.91	0.52
1:A:267:VAL:HG21	1:A:440:PRO:HG3	1.90	0.52
2:B:134:VAL:HB	2:B:195:TYR:HB2	1.91	0.52
1:A:78:PHE:HB3	1:A:559:ARG:CZ	2.39	0.52
2:B:76:ASP:CB	2:B:559:ARG:NH2	2.73	0.52
2:B:395:SER:HB3	3:C:324:ILE:CG2	2.39	0.52
2:B:76:ASP:HB3	2:B:559:ARG:NH2	2.25	0.52
1:A:78:PHE:CB	1:A:559:ARG:CZ	2.88	0.52
1:A:126:SER:HB3	1:A:129:PHE:HD2	1.75	0.52
1:A:338:ASP:HB2	1:A:444:VAL:HG22	1.91	0.52
3:C:431:ALA:HB1	3:C:481:ASN:HD21	1.76	0.51
2:B:56:GLY:HA3	2:B:224:VAL:HB	1.91	0.51
1:A:55:ILE:O	3:C:45:ASN:ND2	2.41	0.51
1:A:74:TYR:HE1	1:A:77:VAL:HG13	1.76	0.51
1:A:175:PRO:HA	2:B:177:MET:HB2	1.92	0.51
2:B:447:PRO:HB3	3:C:330:GLN:OE1	2.04	0.51
1:A:297:HIS:O	1:A:417:GLY:HA3	2.11	0.51
3:C:382:THR:O	3:C:398:GLN:NE2	2.44	0.50
2:B:110:TYR:O	2:B:227:ARG:NH1	2.44	0.50
3:C:309:ALA:HB1	3:C:409:THR:HA	1.93	0.50
2:B:76:ASP:HB3	2:B:559:ARG:HH22	1.77	0.50
3:C:115:GLY:HA3	3:C:221:PHE:HA	1.93	0.50
2:B:144:ILE:HG12	2:B:148:LEU:HD22	1.93	0.49
1:A:121:PHE:HZ	1:A:190:LEU:HD21	1.76	0.49
1:A:49:SER:OG	1:A:174:ARG:NH2	2.45	0.49
3:C:274:CYS:HB2	3:C:449:THR:HG21	1.95	0.49
2:B:133:LEU:HD13	2:B:192:LEU:HD21	1.94	0.48
3:C:31:ASP:O	3:C:120:ARG:NH1	2.46	0.48
2:B:97:PRO:HB3	2:B:103:THR:HG22	1.95	0.48
1:A:143:GLU:OE1	2:B:234:VAL:HA	2.14	0.48
1:A:87:GLY:N	1:A:194:VAL:O	2.46	0.48
2:B:200:ASN:O	2:B:202:PHE:N	2.43	0.48
2:B:229:PRO:O	2:B:230:SER:C	2.52	0.48

Continued on next page...

Not For Manuscript Review

Continued from previous page...

Atom-1	Atom-2	Interatomic distance (Å)	Clash overlap (Å)
2:B:280:LEU:HD12	2:B:280:LEU:HA	1.69	0.48
1:A:115:GLY:HA3	1:A:221:PHE:HA	1.95	0.48
2:B:182:GLY:HA2	2:B:233:THR:HB	1.96	0.48
1:A:240:ALA:HA	1:A:515:TRP:HZ2	1.79	0.47
3:C:69:ARG:NH2	3:C:218:SER:O	2.47	0.47
2:B:280:LEU:HD22	2:B:530:VAL:HA	1.97	0.47
1:A:45:ASN:ND2	2:B:55:ILE:O	2.41	0.47
3:C:458:ASN:HA	3:C:537:GLY:HA2	1.96	0.46
1:A:462:MET:HG2	1:A:487:THR:HB	1.97	0.46
2:B:431:ALA:HA	2:B:434:ILE:HD11	1.97	0.46
1:A:91:TYR:HB3	1:A:192:LEU:HB2	1.97	0.46
3:C:529:SER:OG	3:C:530:VAL:N	2.48	0.46
1:A:238:SER:HA	1:A:506:ALA:HB1	1.96	0.46
3:C:174:ARG:NH1	3:C:177:MET:O	2.49	0.46
1:A:42:THR:OG1	1:A:43:ALA:N	2.48	0.45
2:B:36:ALA:HB1	2:B:156:VAL:HG11	1.99	0.45
1:A:421:MET:SD	1:A:421:MET:N	2.90	0.45
3:C:132:ARG:HG2	3:C:159:ASP:HA	1.98	0.45
3:C:519:PHE:HE2	3:C:547:LEU:HA	1.82	0.44
3:C:276:ARG:HH22	3:C:335:GLY:HA2	1.82	0.44
2:B:315:PHE:HZ	2:B:388:LEU:HD11	1.82	0.44
1:A:246:PRO:C	1:A:248:LEU:N	2.71	0.44
2:B:98:GLN:HA	2:B:104:ALA:HB2	2.00	0.44
3:C:97:PRO:HD3	3:C:113:TRP:CE2	2.53	0.44
3:C:95:HIS:HB3	3:C:187:VAL:HB	2.01	0.43
1:A:100:ASN:ND2	1:A:215:THR:O	2.51	0.43
1:A:227:ARG:HE	1:A:231:ALA:HB3	1.84	0.43
1:A:65:GLN:N	1:A:65:GLN:OE1	2.52	0.43
3:C:76:ASP:OD1	3:C:516:GLN:NE2	2.51	0.43
2:B:100:ASN:ND2	2:B:215:THR:O	2.51	0.43
2:B:421:MET:N	2:B:421:MET:SD	2.91	0.43
2:B:559:ARG:HD3	2:B:559:ARG:HA	1.82	0.43
1:A:492:LEU:N	1:A:493:PRO:HD2	2.33	0.43
2:B:229:PRO:O	2:B:230:SER:OG	2.35	0.43
3:C:568:ASN:OD1	3:C:568:ASN:N	2.51	0.43
1:A:32:PRO:HB2	1:A:34:VAL:HG23	2.01	0.43
3:C:426:ILE:HD12	3:C:437:THR:HG21	2.00	0.43
1:A:80:TRP:HB3	1:A:209:ILE:HG22	2.01	0.42
1:A:120:ARG:HG2	1:A:165:PRO:HB2	2.00	0.42
2:B:31:ASP:O	2:B:120:ARG:NH1	2.52	0.42
1:A:89:ILE:HG23	1:A:191:VAL:HG13	2.01	0.42

Continued on next page...

Not For Manuscript Review

Continued from previous page...

Atom-1	Atom-2	Interatomic distance (Å)	Clash overlap (Å)
3:C:426:ILE:HB	3:C:469:ARG:HB3	2.02	0.42
3:C:98:GLN:HA	3:C:104:ALA:HB2	2.02	0.42
3:C:358:GLY:HA3	3:C:420:VAL:HG22	2.02	0.42
2:B:55:ILE:HD12	2:B:55:ILE:HA	1.89	0.42
1:A:173:LEU:HD13	2:B:225:MET:HG2	2.02	0.42
2:B:265:GLN:HA	2:B:266:PRO:HD3	1.95	0.42
1:A:559:ARG:HD3	1:A:559:ARG:HA	1.83	0.41
2:B:245:THR:N	2:B:246:PRO:HD2	2.36	0.41
1:A:144:ILE:HD12	1:A:191:VAL:HG21	2.01	0.41
2:B:135:ALA:HB2	2:B:192:LEU:HD12	2.03	0.41
3:C:341:PHE:HZ	3:C:402:LYS:HB3	1.86	0.41
3:C:34:VAL:HG13	3:C:166:VAL:HG23	2.02	0.40
1:A:209:ILE:HD12	1:A:209:ILE:HA	1.81	0.40
2:B:525:GLU:HB2	2:B:565:LEU:HB3	2.03	0.40
3:C:351:ALA:O	3:C:352:ALA:C	2.60	0.40
3:C:97:PRO:O	3:C:103:THR:OG1	2.34	0.40

There are no symmetry-related clashes.

5.3 Torsion angles (i)

5.3.1 Protein backbone (i)

In the following table, the Percentiles column shows the percent Ramachandran outliers of the chain as a percentile score with respect to all PDB entries followed by that with respect to all EM entries.

The Analysed column shows the number of residues for which the backbone conformation was analysed, and the total number of residues.

Mol	Chain	Analysed	Favoured	Allowed	Outliers	Percentiles	
1	A	540/542 (100%)	486 (90%)	49 (9%)	5 (1%)	14	44
2	B	549/551 (100%)	505 (92%)	42 (8%)	2 (0%)	30	61
3	C	549/551 (100%)	530 (96%)	19 (4%)	0	100	100
All	All	1638/1644 (100%)	1521 (93%)	110 (7%)	7 (0%)	32	61

All (7) Ramachandran outliers are listed below:

Mol	Chain	Res	Type
1	A	239	PRO

Continued on next page...

Not For Manuscript Review

Continued from previous page...

Mol	Chain	Res	Type
1	A	431	ALA
2	B	434	ILE
2	B	227	ARG
1	A	238	SER
1	A	237	ILE
1	A	247	VAL

5.3.2 Protein sidechains (i)

In the following table, the Percentiles column shows the percent sidechain outliers of the chain as a percentile score with respect to all PDB entries followed by that with respect to all EM entries.

The Analysed column shows the number of residues for which the sidechain conformation was analysed, and the total number of residues.

Mol	Chain	Analysed	Rotameric	Outliers	Percentiles	
1	A	430/430 (100%)	403 (94%)	27 (6%)	15	41
2	B	437/437 (100%)	413 (94%)	24 (6%)	18	45
3	C	438/438 (100%)	422 (96%)	16 (4%)	29	56
All	All	1305/1305 (100%)	1238 (95%)	67 (5%)	22	48

All (67) residues with a non-rotameric sidechain are listed below:

Mol	Chain	Res	Type
1	A	30	MET
1	A	46	SER
1	A	159	ASP
1	A	237	ILE
1	A	242	LEU
1	A	245	THR
1	A	254	ASP
1	A	257	TRP
1	A	262	VAL
1	A	264	LEU
1	A	272	SER
1	A	278	TRP
1	A	291	ARG
1	A	294	ASP
1	A	317	TYR
1	A	339	MET

Continued on next page...

Not For Manuscript Review

Continued from previous page...

Mol	Chain	Res	Type
1	A	369	ASN
1	A	372	THR
1	A	378	LEU
1	A	421	MET
1	A	428	THR
1	A	430	SER
1	A	442	ARG
1	A	462	MET
1	A	512	PHE
1	A	516	GLN
1	A	544	LEU
2	B	192	LEU
2	B	232	LYS
2	B	242	LEU
2	B	245	THR
2	B	254	ASP
2	B	257	TRP
2	B	260	GLN
2	B	264	LEU
2	B	267	VAL
2	B	279	ASN
2	B	280	LEU
2	B	297	HIS
2	B	299	ARG
2	B	304	TYR
2	B	313	LEU
2	B	314	GLN
2	B	315	PHE
2	B	317	TYR
2	B	402	LYS
2	B	421	MET
2	B	442	ARG
2	B	469	ARG
2	B	492	LEU
2	B	512	PHE
3	C	21	SER
3	C	39	SER
3	C	108	GLN
3	C	161	ARG
3	C	230	SER
3	C	260	GLN
3	C	274	CYS

Continued on next page...

Not For Manuscript Review

Continued from previous page...

Mol	Chain	Res	Type
3	C	278	TRP
3	C	294	ASP
3	C	319	ASN
3	C	341	PHE
3	C	437	THR
3	C	469	ARG
3	C	523	PHE
3	C	541	SER
3	C	544	LEU

Sometimes sidechains can be flipped to improve hydrogen bonding and reduce clashes. All (7) such sidechains are listed below:

Mol	Chain	Res	Type
1	A	100	ASN
2	B	196	ASN
2	B	200	ASN
2	B	260	GLN
3	C	200	ASN
3	C	330	GLN
3	C	439	GLN

5.3.3 RNA (i)

There are no RNA molecules in this entry.

5.4 Non-standard residues in protein, DNA, RNA chains (i)

There are no non-standard protein/DNA/RNA residues in this entry.

5.5 Carbohydrates (i)

There are no oligosaccharides in this entry.

5.6 Ligand geometry (i)

There are no ligands in this entry.

Not For Manuscript Review

5.7 Other polymers (i)

There are no such residues in this entry.

5.8 Polymer linkage issues (i)

There are no chain breaks in this entry.

Not For Manuscript Review

6 Map visualisation (i)

This section contains visualisations of the EMDB entry D_1292144900. These allow visual inspection of the internal detail of the map and identification of artifacts.

Images derived from a raw map, generated by summing the deposited half-maps, are presented below the corresponding image components of the primary map to allow further visual inspection and comparison with those of the primary map.

6.1 Orthogonal projections (i)

6.1.1 Primary map

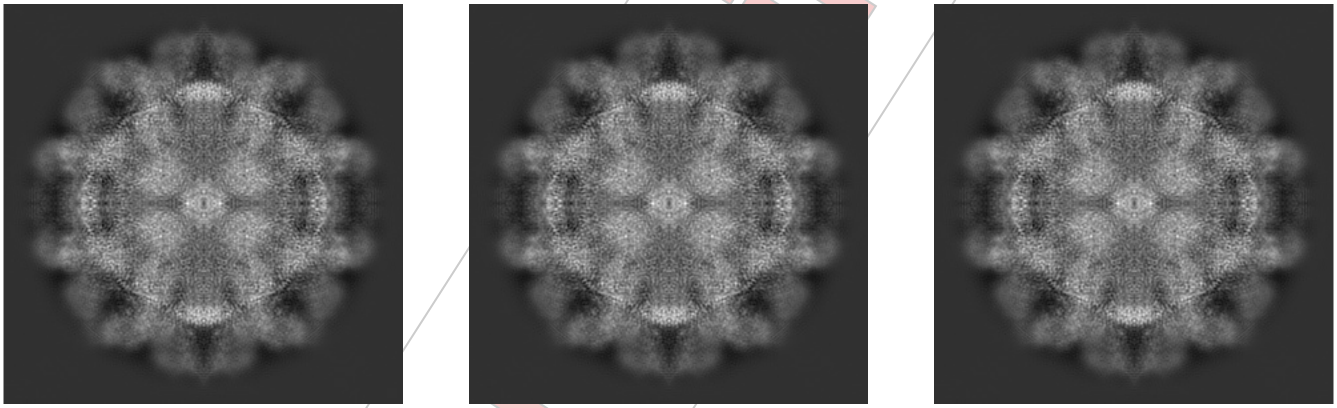

X Y Z

6.1.2 Raw map

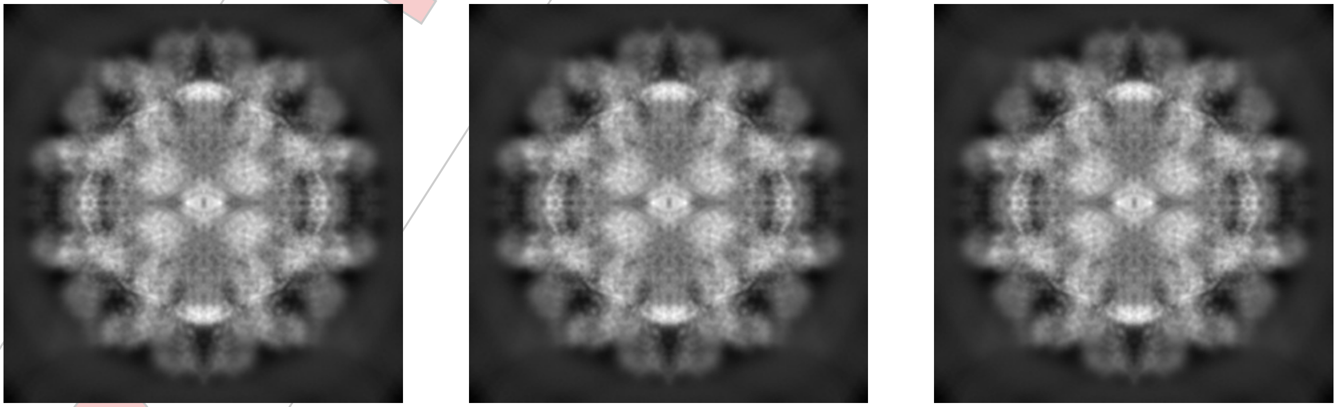

X Y Z

The images above show the map projected in three orthogonal directions.

Not For Manuscript Review

6.2 Central slices (i)

6.2.1 Primary map

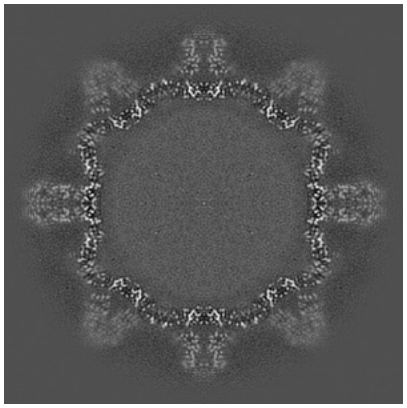

X Index: 165

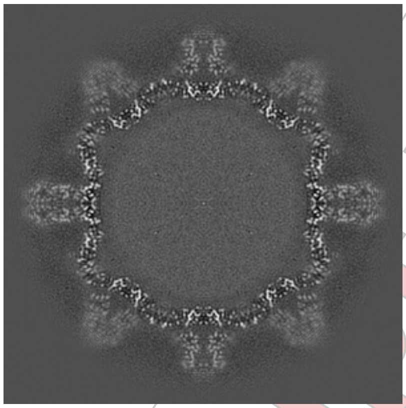

Y Index: 165

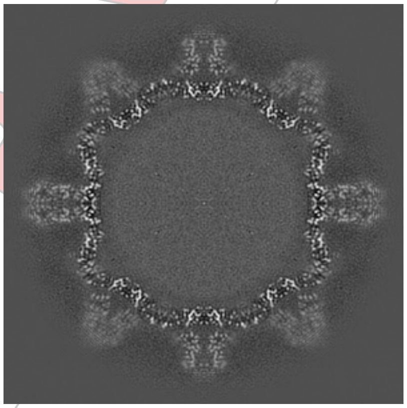

Z Index: 165

6.2.2 Raw map

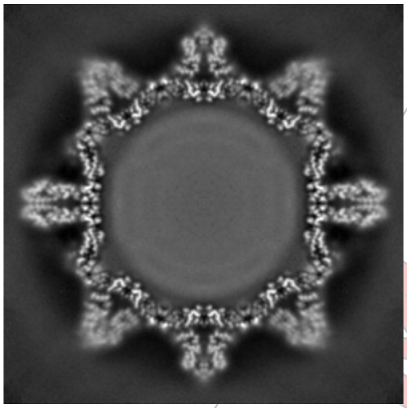

X Index: 165

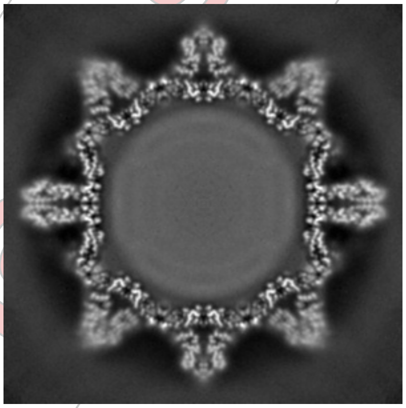

Y Index: 165

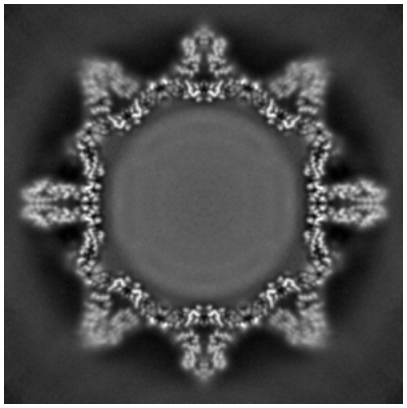

Z Index: 165

The images above show central slices of the map in three orthogonal directions.

Not For Manuscript Review

6.3 Largest variance slices (i)

6.3.1 Primary map

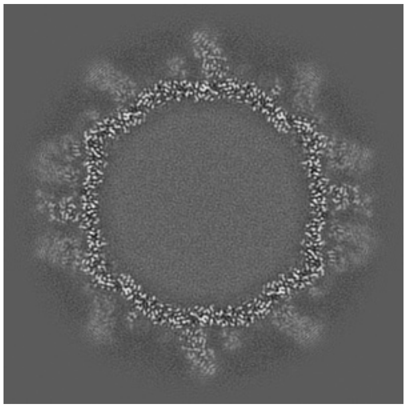
X Index: 178

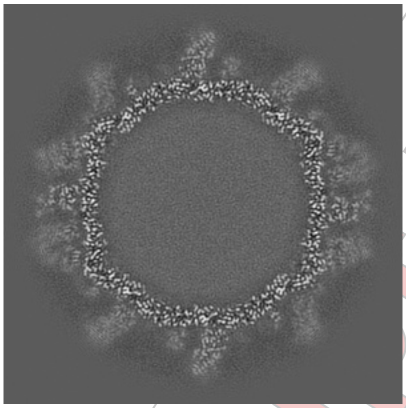
Y Index: 152

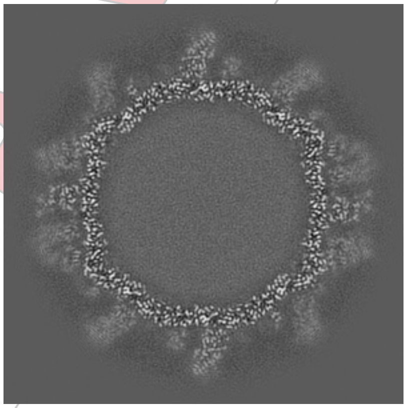
Z Index: 152

6.3.2 Raw map

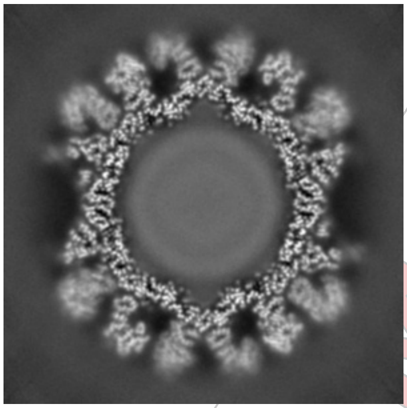
X Index: 117

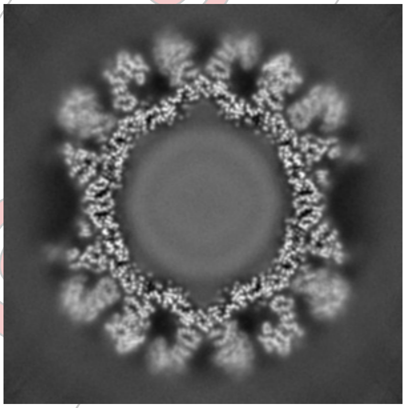
Y Index: 213

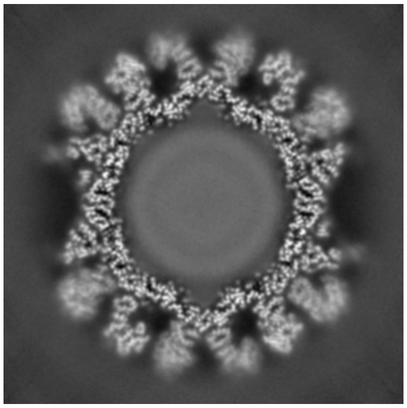
Z Index: 117

The images above show the largest variance slices of the map in three orthogonal directions.

Not For Manuscript Review

6.4 Orthogonal standard-deviation projections (False-color) (i)

6.4.1 Primary map

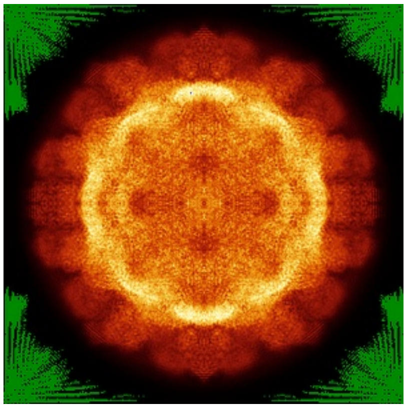
X

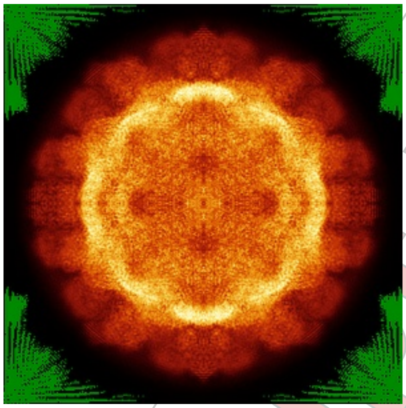
Y

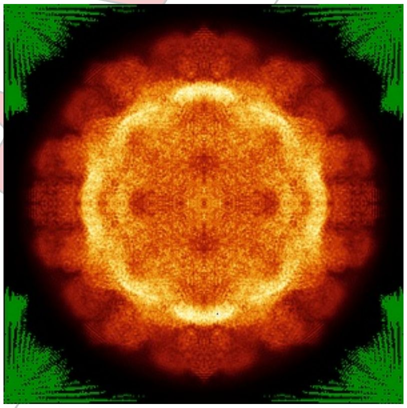
Z

6.4.2 Raw map

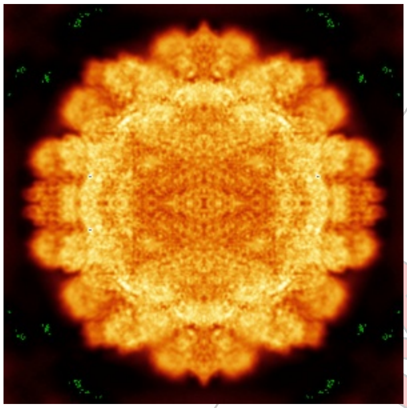
X

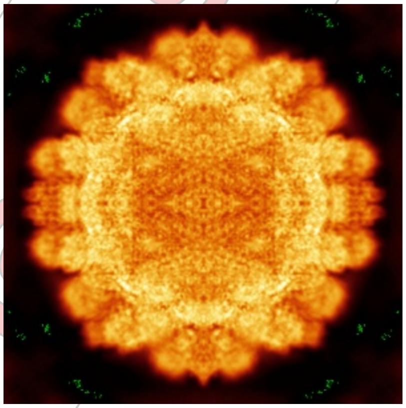
Y

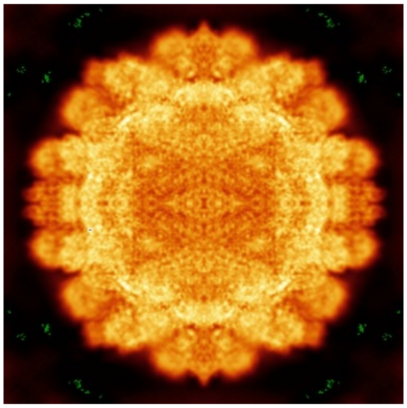
Z

The images above show the map standard deviation projections with false color in three orthogonal directions. Minimum values are shown in green, max in blue, and dark to light orange shades represent small to large values respectively.

Not For Manuscript Review

6.5 Orthogonal surface views (i)

6.5.1 Primary map

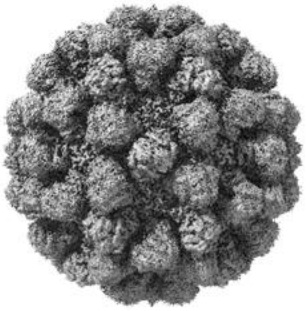
X

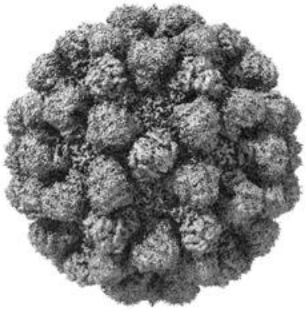
Y

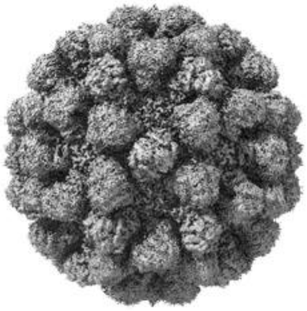
Z

The images above show the 3D surface view of the map at the recommended contour level 0.55. These images, in conjunction with the slice images, may facilitate assessment of whether an appropriate contour level has been provided.

6.5.2 Raw map

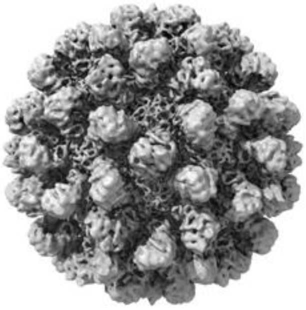
X

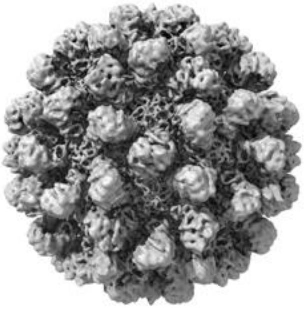
Y

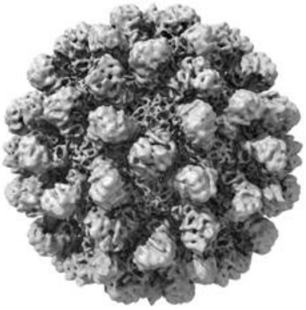
Z

These images show the 3D surface of the raw map. The raw map's contour level was selected so that its surface encloses the same volume as the primary map does at its recommended contour level.

6.6 Mask visualisation (i)

This section was not generated. No masks/segmentation were deposited.

Not For Manuscript Review

7 Map analysis (i)

This section contains the results of statistical analysis of the map.

7.1 Map-value distribution (i)

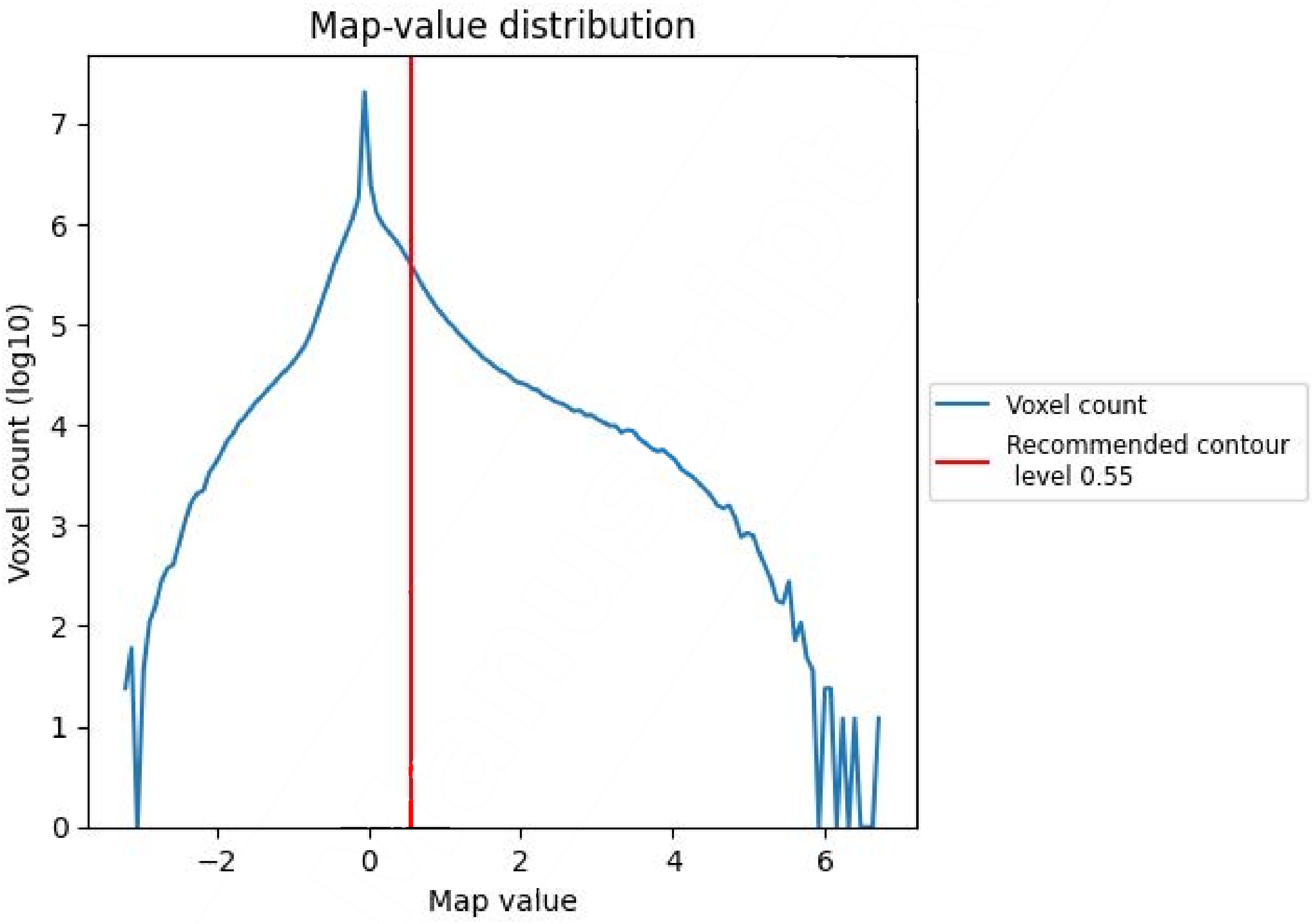

The map-value distribution is plotted in 128 intervals along the x-axis. The y-axis is logarithmic. A spike in this graph at zero usually indicates that the volume has been masked.

Not For Manuscript Review

7.2 Volume estimate (i)

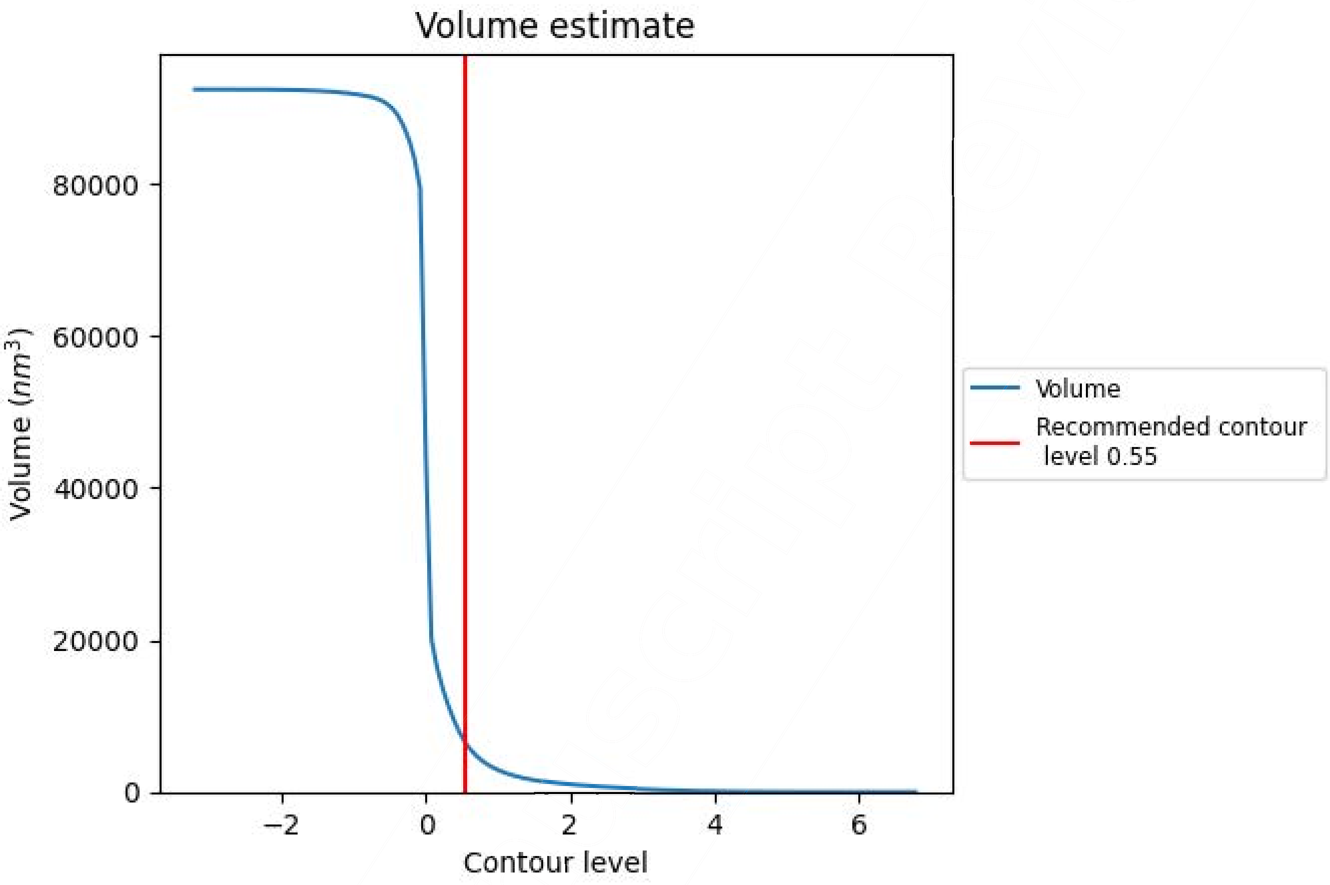

The volume at the recommended contour level is 6510 nm^3; this corresponds to an approximate mass of 5880 kDa.

The volume estimate graph shows how the enclosed volume varies with the contour level. The recommended contour level is shown as a vertical line and the intersection between the line and the curve gives the volume of the enclosed surface at the given level.

Not For Manuscript Review

7.3 Rotationally averaged power spectrum (i)

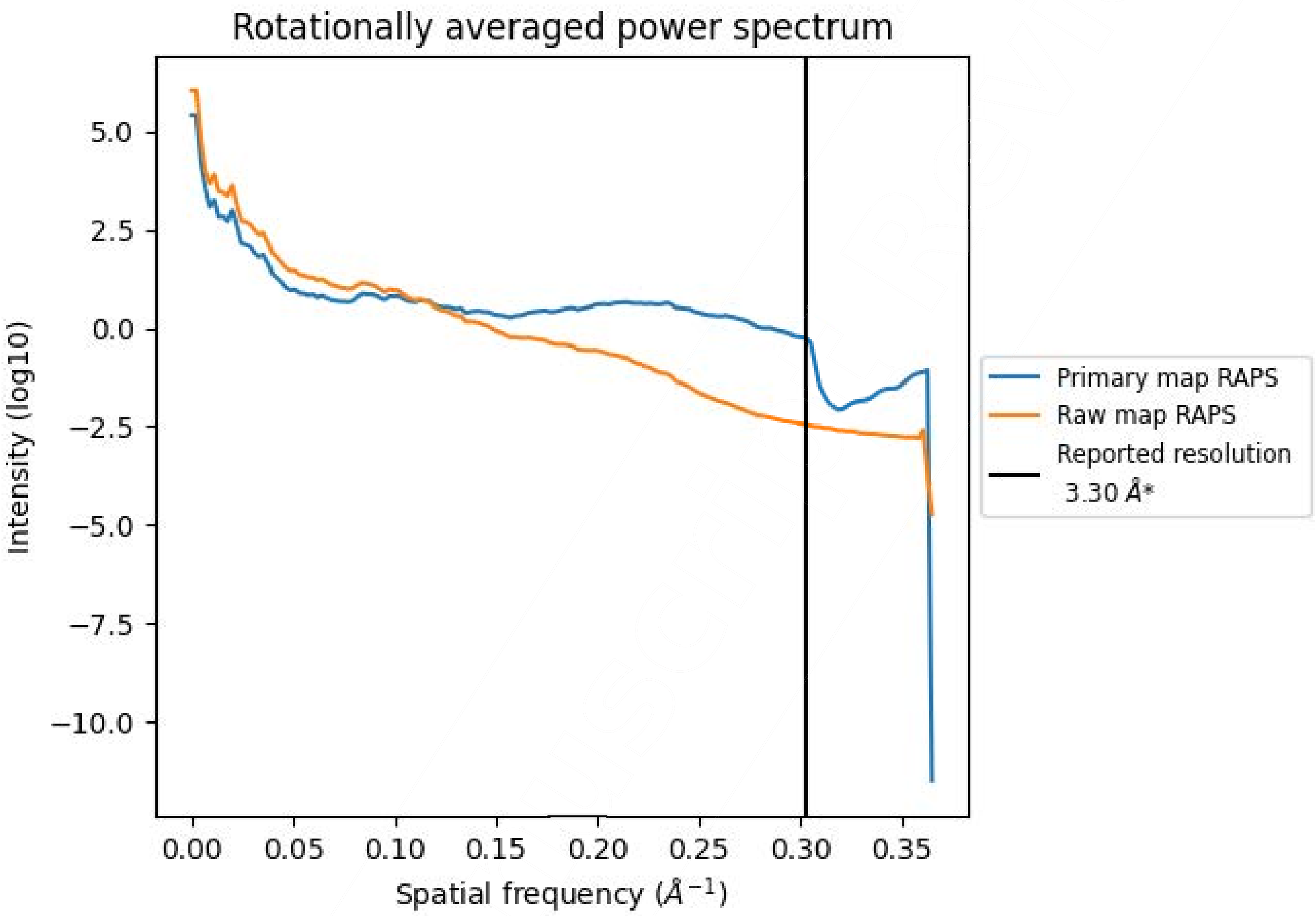

*Reported resolution corresponds to spatial frequency of 0.303 Å^{-1}

Not For Manuscript Review

8 Fourier-Shell correlation (i)

Fourier-Shell Correlation (FSC) is the most commonly used method to estimate the resolution of single-particle and subtomogram-averaged maps. The shape of the curve depends on the imposed symmetry, mask and whether or not the two 3D reconstructions used were processed from a common reference. The reported resolution is shown as a black line. A curve is displayed for the half-bit criterion in addition to lines showing the 0.143 gold standard cut-off and 0.5 cut-off.

8.1 FSC (i)

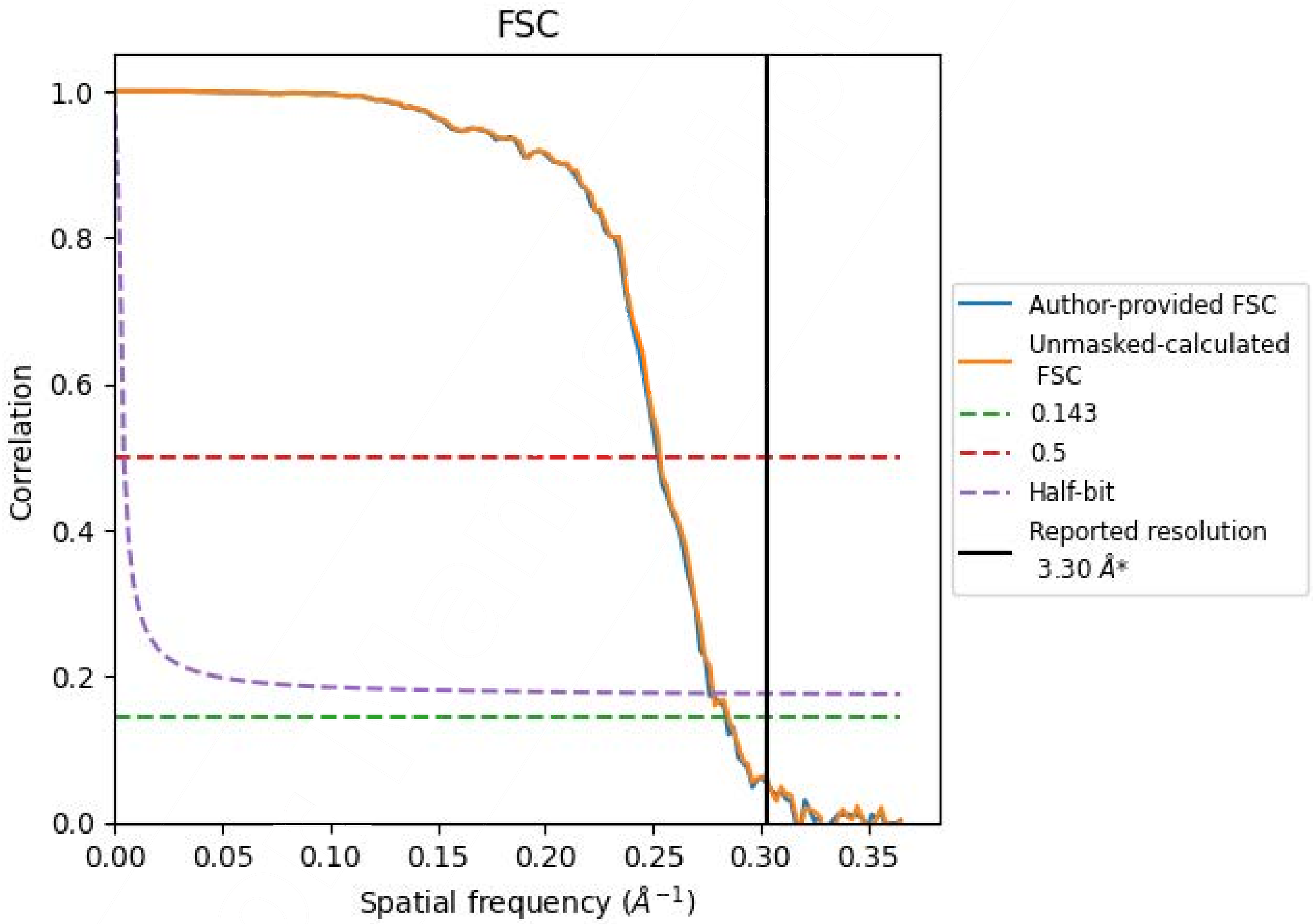

*Reported resolution corresponds to spatial frequency of 0.303 Å^{-1}

Not For Manuscript Review

8.2 Resolution estimates (i)

Resolution estimate (Å)	Estimation criterion (FSC cut-off)		
	0.143	0.5	Half-bit
Reported by author	3.30	-	-
Author-provided FSC curve	3.52	3.96	3.62
Unmasked-calculated*	3.51	3.95	3.60

*Resolution estimate based on FSC curve calculated by comparison of deposited half-maps.

Not For Manuscript Review

9 Map-model fit (i)

This section contains information regarding the fit between EMDB map D_1292144900 and PDB model D_1292144900. Per-residue inclusion information can be found in section 3 on page 4.

9.1 Map-model overlays

9.1.1 Map-model overlay (i)

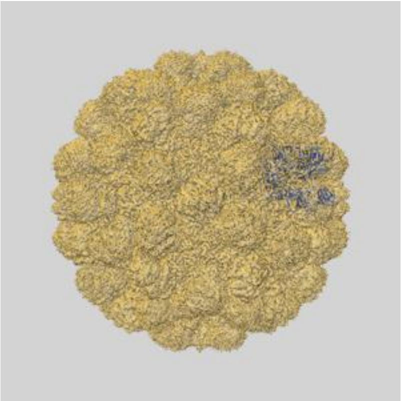

X

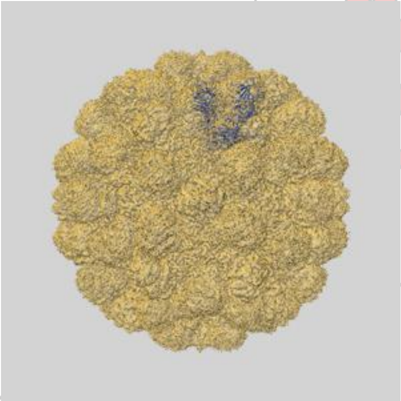

Y

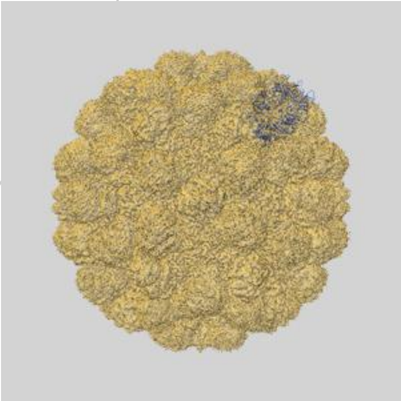

Z

9.1.2 Map-model assembly overlay (i)

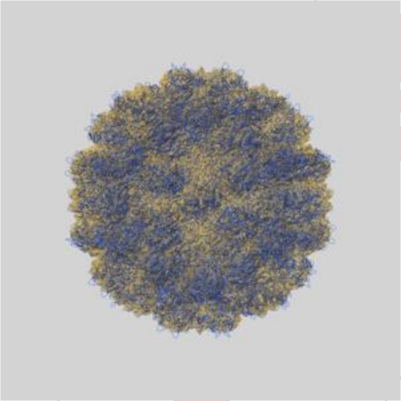

X

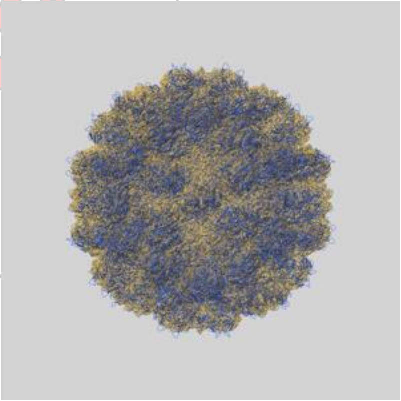

Y

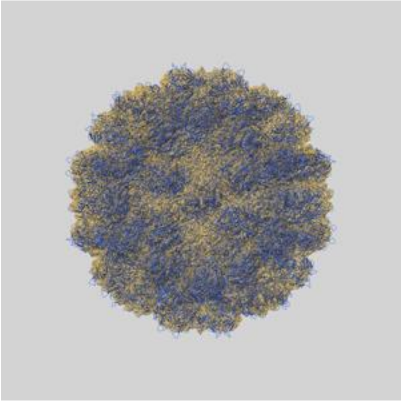

Z

The images above show the 3D surface view of the map at the recommended contour level 0.55 at 50% transparency in yellow overlaid with a ribbon representation of the model coloured in blue. These images allow for the visual assessment of the quality of fit between the atomic model and the map.

Not For Manuscript Review

9.2 Q-score mapped to coordinate model (i)

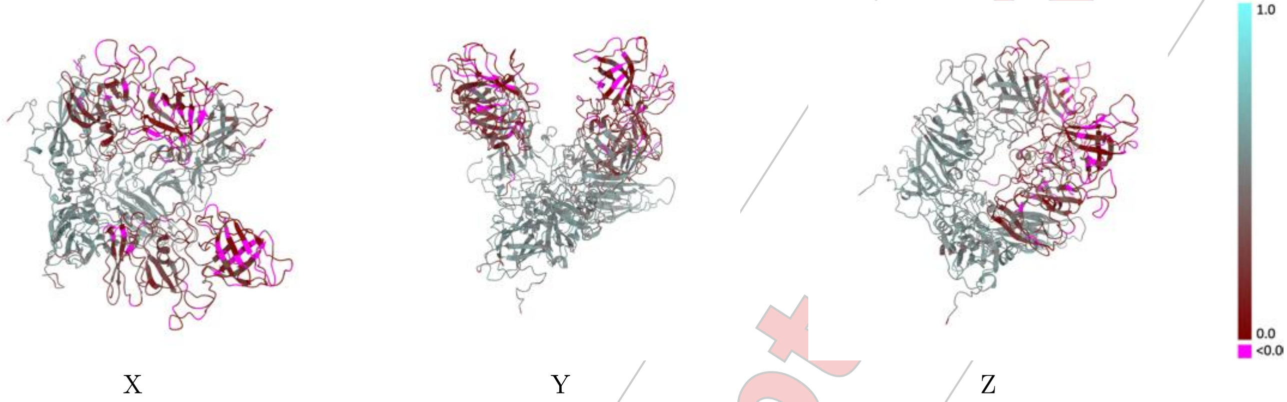

The images above show the model with each residue coloured according its Q-score. This shows their resolvability in the map with higher Q-score values reflecting better resolvability. Please note: Q-score is calculating the resolvability of atoms, and thus high values are only expected at resolutions at which atoms can be resolved. Low Q-score values may therefore be expected for many entries.

9.3 Atom inclusion mapped to coordinate model (i)

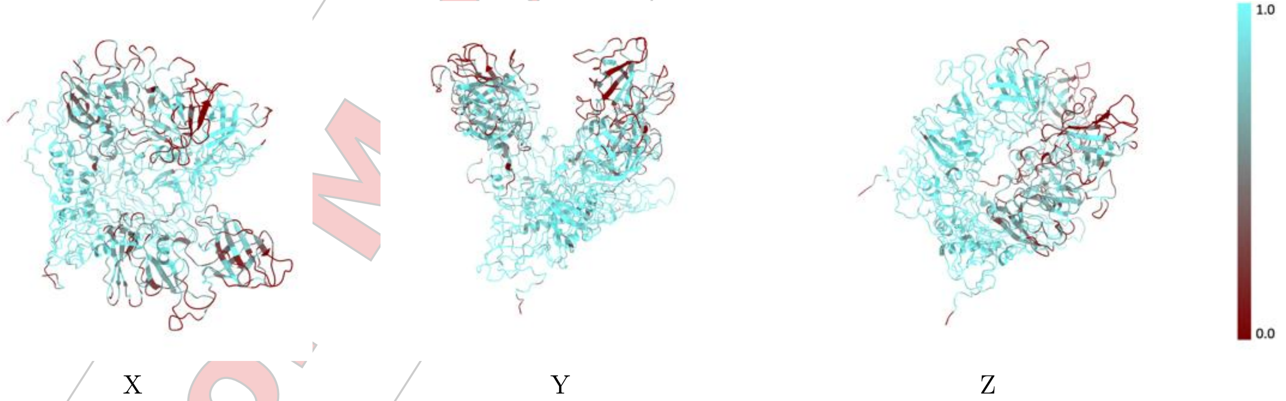

The images above show the model with each residue coloured according to its atom inclusion. This shows to what extent they are inside the map at the recommended contour level (0.55).

Not For Manuscript Review

9.4 Atom inclusion (i)

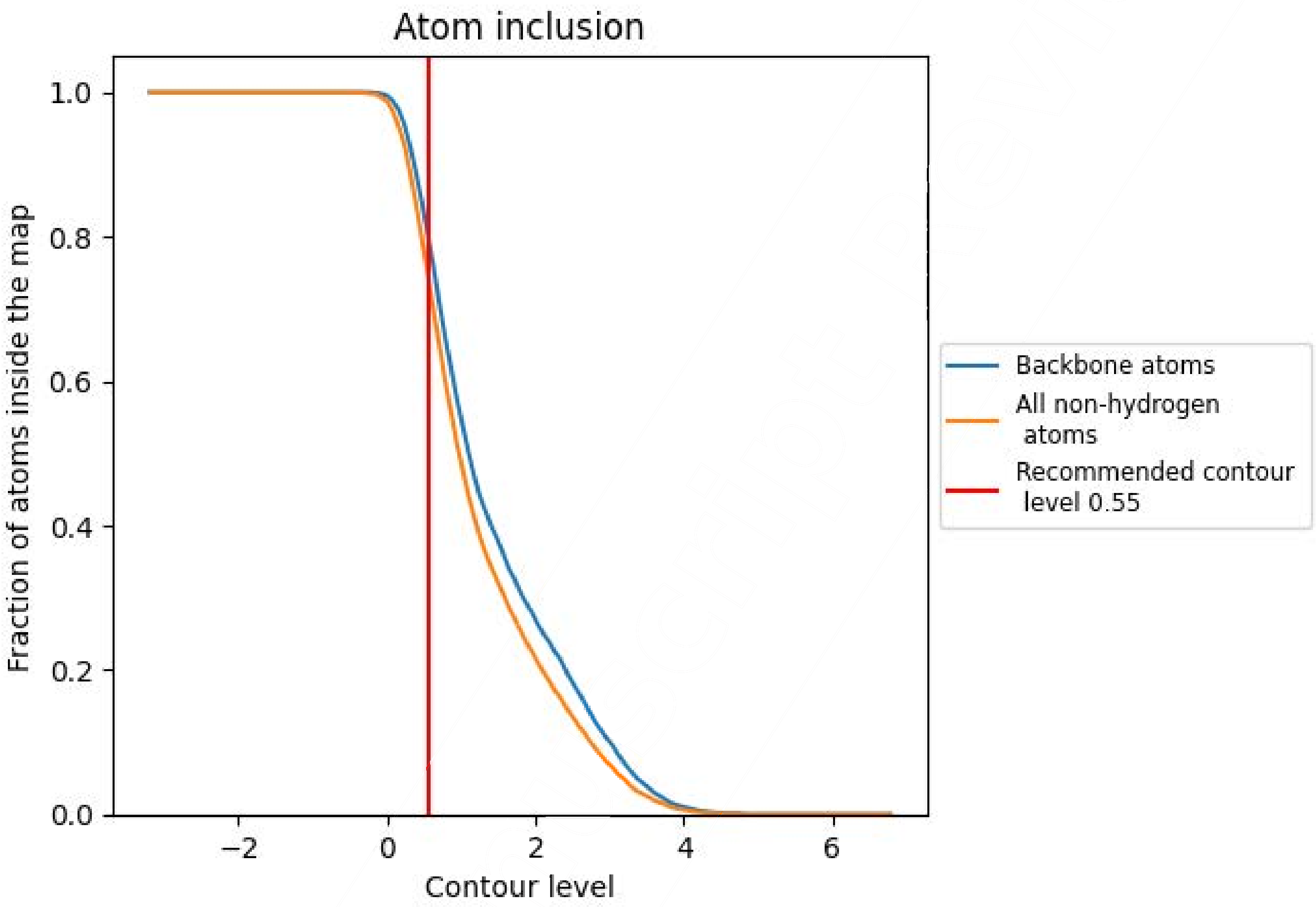

At the recommended contour level, 80% of all backbone atoms, 75% of all non-hydrogen atoms, are inside the map.

Not For Manuscript Review

Preliminary Full wwPDB EM Validation Report (i)

Feb 6, 2025 – 10:34 am GMT

Deposition ID : D_1292145342

This wwPDB validation report is NOT for manuscript review

This is a Preliminary Full wwPDB EM Validation Report.

This report is produced by the wwPDB Deposition System during initial deposition but before annotation of the structure.

We welcome your comments at *validation@mail.wwpdb.org*
A user guide is available at
https://www.wwpdb.org/validation/2017/EMValidationReportHelp
with specific help available everywhere you see the (i) symbol.

The types of validation reports are described at
http://www.wwpdb.org/validation/2017/FAQs#types.

Not For Manuscript Review

The following versions of software and data (see references (i)) were used in the production of this report:

EMDB validation analysis	:	0.0.1.dev113
MolProbity	:	4.02b-467
Percentile statistics	:	20231227.v01 (using entries in the PDB archive December 27th 2023)
MapQ	:	1.9.13
Ideal geometry (proteins)	:	Engh & Huber (2001)
Ideal geometry (DNA, RNA)	:	Parkinson et al. (1996)
Validation Pipeline (wwPDB-VP)	:	2.40

1 Overall quality at a glance (i)

The following experimental techniques were used to determine the structure:
ELECTRON MICROSCOPY

The reported resolution of this entry is unknown.

Percentile scores (ranging between 0-100) for global validation metrics of the entry are shown in the following graphic. The table shows the number of entries on which the scores are based.

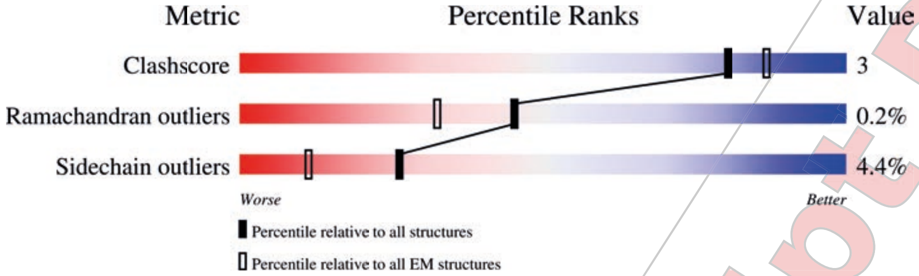

Metric	Whole archive (#Entries)	EM structures (#Entries)
Clashscore	210492	15764
Ramachandran outliers	207382	16835
Sidechain outliers	206894	16415

The table below summarises the geometric issues observed across the polymeric chains and their fit to the map. The red, orange, yellow and green segments of the bar indicate the fraction of residues that contain outliers for >=3, 2, 1 and 0 types of geometric quality criteria respectively. A grey segment represents the fraction of residues that are not modelled. The numeric value for each fraction is indicated below the corresponding segment, with a dot representing fractions <=5% The upper red bar (where present) indicates the fraction of residues that have poor fit to the EM map (all-atom inclusion < 40%). The numeric value is given above the bar.

Mol	Chain	Length	Quality of chain
1	A	534	59% / 90% 10% •
2	B	550	58% / 90% 9% •
3	C	550	15% / 90% 10%

Not For Manuscript Review

2 Entry composition (i)

There are 3 unique types of molecules in this entry. The entry contains 12059 atoms, of which 0 are hydrogens and 0 are deuteriums.

In the tables below, the AltConf column contains the number of residues with at least one atom in alternate conformation and the Trace column contains the number of residues modelled with at most 2 atoms.

- Molecule 1 is a protein.

Mol	Chain	Residues	Atoms					AltConf	Trace
			Total	C	N	O	S		
1	A	534	3949	2508	669	761	11	0	0

- Molecule 2 is a protein.

Mol	Chain	Residues	Atoms					AltConf	Trace
			Total	C	N	O	S		
2	B	550	4054	2571	685	786	12	0	0

- Molecule 3 is a protein.

Mol	Chain	Residues	Atoms					AltConf	Trace
			Total	C	N	O	S		
3	C	550	4056	2572	685	787	12	0	0

Not For Manuscript Review

3 Residue-property plots ⓘ

These plots are drawn for all protein, RNA, DNA and oligosaccharide chains in the entry. The first graphic for a chain summarises the proportions of the various outlier classes displayed in the second graphic. The second graphic shows the sequence view annotated by issues in geometry and atom inclusion in map density. Residues are color-coded according to the number of geometric quality criteria for which they contain at least one outlier: green = 0, yellow = 1, orange = 2 and red = 3 or more. A red diamond above a residue indicates a poor fit to the EM map for this residue (all-atom inclusion < 40%). Stretches of 2 or more consecutive residues without any outlier are shown as a green connector. Residues present in the sample, but not in the model, are shown in grey.

- Molecule 1:

Chain A:
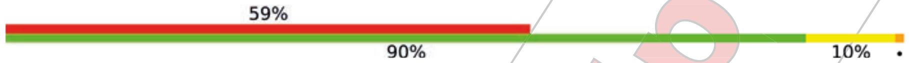

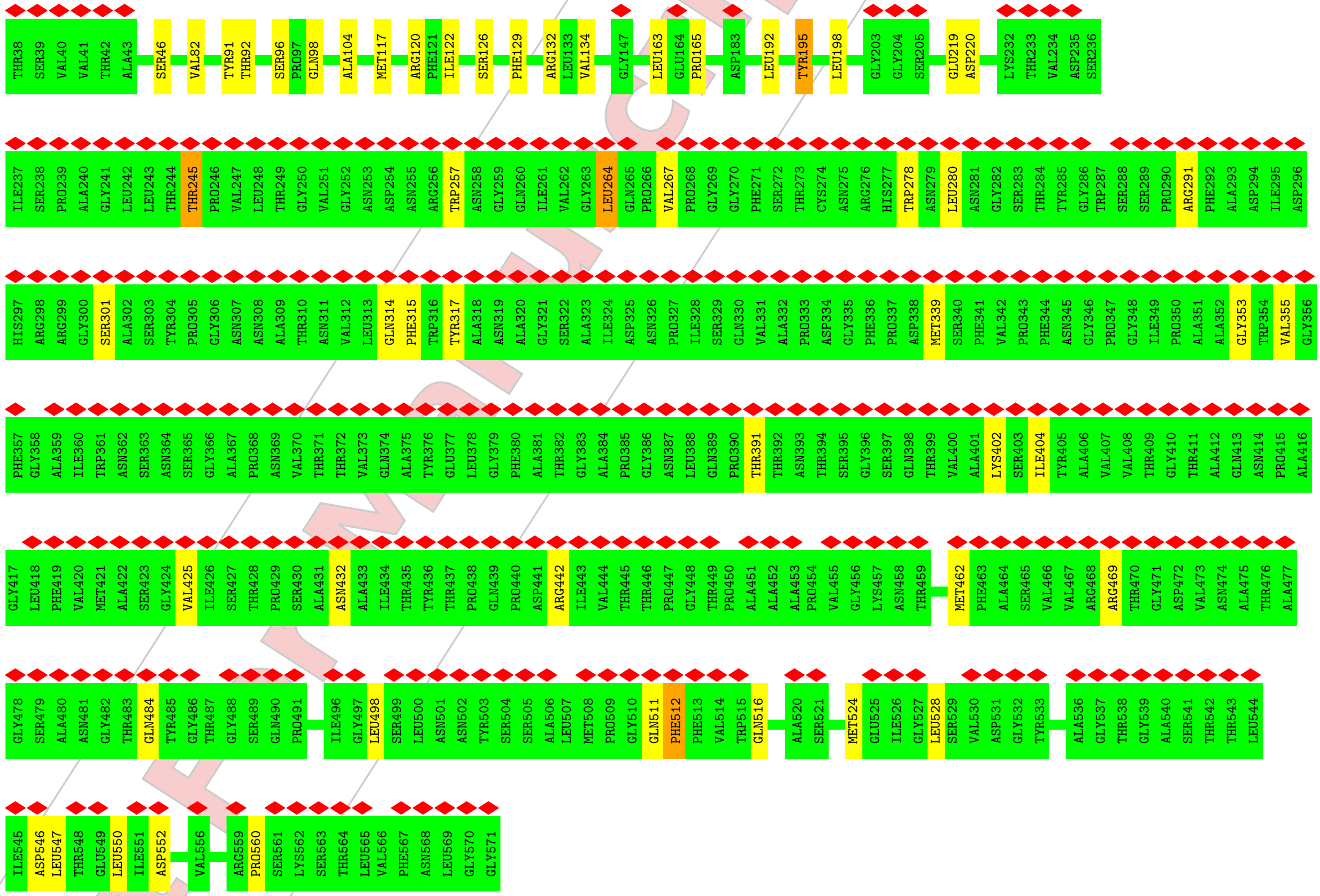

- Molecule 2:

Chain B:
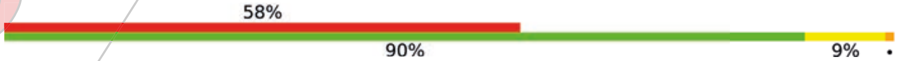

Not For Manuscript Review

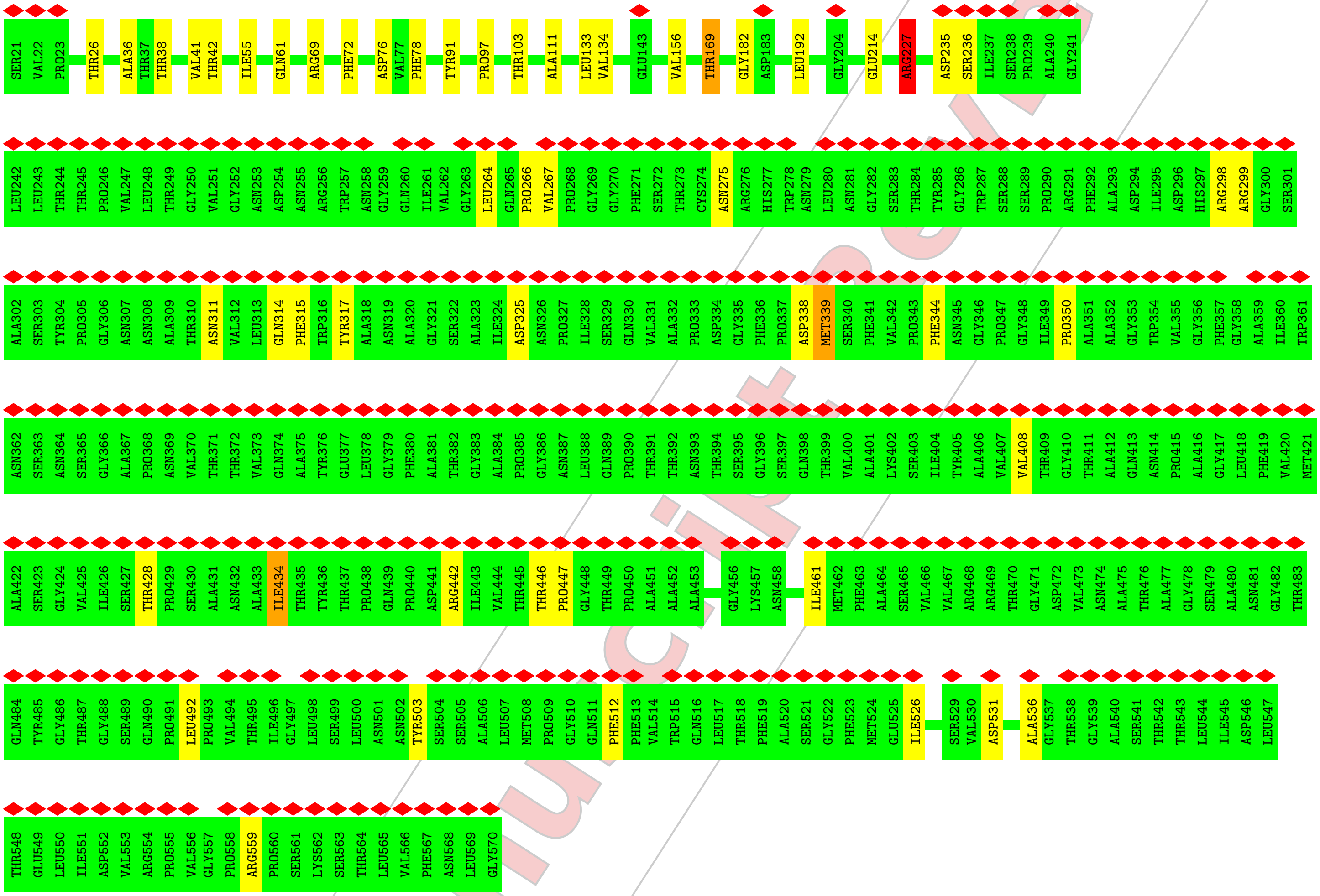

- Molecule 3:

Chain C:

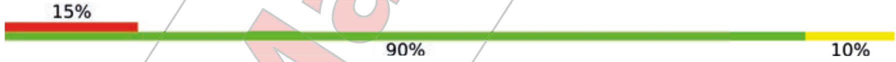

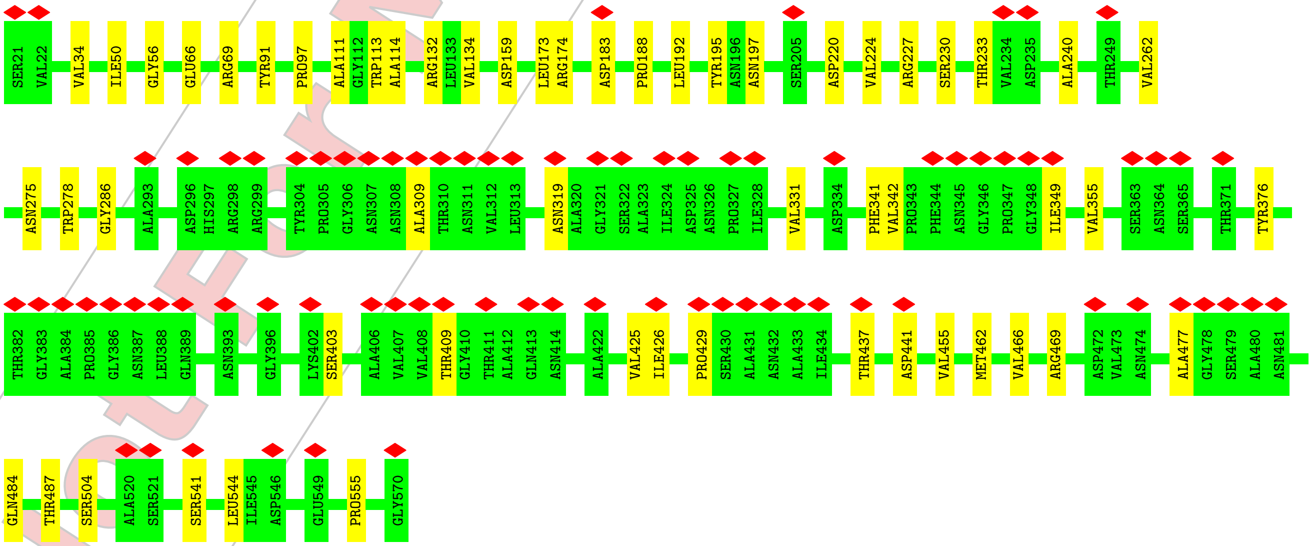

Not For Manuscript Review

4 Experimental information (i)

Property	Value	Source
EM reconstruction method	SINGLE PARTICLE	Depositor
Imposed symmetry	POINT, Not provided	
Number of particles used	Not provided	
Resolution determination method	Not provided	
CTF correction method	Not provided	
Microscope	Not provided	
Voltage (kV)	Not provided	
Electron dose ($e^-/\text{Å}^2$)	Not provided	
Minimum defocus (nm)	Not provided	
Maximum defocus (nm)	Not provided	
Magnification	Not provided	
Image detector	Not provided	
Maximum map value	8.744	Depositor
Minimum map value	-4.868	Depositor
Average map value	0.061	Depositor
Map value standard deviation	0.535	Depositor
Recommended contour level	1.2	Depositor
Map size (Å)	452.1, 452.1, 452.1	wwPDB
Map dimensions	330, 330, 330	wwPDB
Map angles (°)	90.0, 90.0, 90.0	wwPDB
Pixel spacing (Å)	1.37, 1.37, 1.37	Depositor

Not For Manuscript Review

5 Model quality (i)

5.1 Standard geometry (i)

The Z score for a bond length (or angle) is the number of standard deviations the observed value is removed from the expected value. A bond length (or angle) with $|Z| > 5$ is considered an outlier worth inspection. RMSZ is the root-mean-square of all Z scores of the bond lengths (or angles).

Mol	Chain	Bond lengths		Bond angles	
		RMSZ	#|Z| >5	RMSZ	#|Z| >5
1	A	0.31	0/4061	0.60	2/5578 (0.0%)
2	B	0.31	0/4169	0.59	1/5731 (0.0%)
3	C	0.31	0/4171	0.57	1/5733 (0.0%)
All	All	0.31	0/12401	0.59	4/17042 (0.0%)

There are no bond length outliers.

All (4) bond angle outliers are listed below:

Mol	Chain	Res	Type	Atoms	Z	Observed(^{o})	Ideal(^{o})
2	B	76	ASP	CB-CG-OD1	6.67	124.30	118.30
1	A	264	LEU	CA-CB-CG	6.27	129.72	115.30
3	C	183	ASP	CB-CG-OD1	5.82	123.54	118.30
1	A	220	ASP	CB-CG-OD2	5.70	123.43	118.30

There are no chirality outliers.

There are no planarity outliers.

5.2 Too-close contacts (i)

In the following table, the Non-H and H(model) columns list the number of non-hydrogen atoms and hydrogen atoms in the chain respectively. The H(added) column lists the number of hydrogen atoms added and optimized by MolProbity. The Clashes column lists the number of clashes within the asymmetric unit, whereas Symm-Clashes lists symmetry-related clashes.

Mol	Chain	Non-H	H(model)	H(added)	Clashes	Symm-Clashes
1	A	3949	0	3817	17	0
2	B	4054	0	3918	21	0
3	C	4056	0	3920	22	0
All	All	12059	0	11655	60	0

The all-atom clashscore is defined as the number of clashes found per 1000 atoms (including

Not For Manuscript Review

hydrogen atoms). The all-atom clashscore for this structure is 3.

All (60) close contacts within the same asymmetric unit are listed below, sorted by their clash magnitude.

Atom-1	Atom-2	Interatomic distance (Å)	Clash overlap (Å)
3:C:134:VAL:HB	3:C:195:TYR:HB2	1.81	0.62
1:A:353:GLY:HA2	1:A:442:ARG:HH22	1.66	0.61
2:B:111:ALA:HB2	2:B:227:ARG:HB3	1.84	0.60
1:A:547:LEU:HD23	1:A:550:LEU:HD13	1.84	0.60
3:C:275:ASN:HA	3:C:286:GLY:HA3	1.86	0.58
1:A:126:SER:HB3	1:A:129:PHE:HD2	1.69	0.57
3:C:91:TYR:HB3	3:C:192:LEU:HB2	1.87	0.57
3:C:376:TYR:HA	3:C:403:SER:HA	1.87	0.57
3:C:66:GLU:HB2	3:C:69:ARG:HG3	1.87	0.56
1:A:120:ARG:HG2	1:A:165:PRO:HB2	1.88	0.55
2:B:339:MET:HG3	2:B:442:ARG:HH21	1.71	0.55
3:C:309:ALA:HB1	3:C:409:THR:HA	1.88	0.55
3:C:56:GLY:HA3	3:C:224:VAL:HB	1.88	0.55
3:C:426:ILE:HD12	3:C:437:THR:HG21	1.90	0.54
3:C:469:ARG:HH11	3:C:484:GLN:HB2	1.74	0.53
1:A:280:LEU:HA	1:A:498:LEU:HD11	1.89	0.53
3:C:349:ILE:HB	3:C:429:PRO:HA	1.90	0.53
1:A:512:PHE:HB2	1:A:528:LEU:H	1.74	0.53
2:B:275:ASN:HD22	2:B:338:ASP:HB3	1.74	0.52
2:B:38:THR:HG22	2:B:156:VAL:HG13	1.92	0.51
3:C:114:ALA:HB1	3:C:174:ARG:HH21	1.74	0.51
2:B:91:TYR:HD2	2:B:559:ARG:HH22	1.58	0.51
3:C:262:VAL:HG21	3:C:466:VAL:HG23	1.92	0.51
1:A:355:VAL:HG23	1:A:425:VAL:HG23	1.91	0.51
3:C:173:LEU:HD12	3:C:188:PRO:HB3	1.93	0.51
3:C:466:VAL:HG21	3:C:477:ALA:HB2	1.93	0.51
2:B:428:THR:HG21	2:B:434:ILE:HD13	1.93	0.50
2:B:78:PHE:HB3	2:B:559:ARG:HH21	1.77	0.50
2:B:311:ASN:H	2:B:408:VAL:HA	1.77	0.49
2:B:97:PRO:HB3	2:B:103:THR:HG22	1.96	0.48
1:A:132:ARG:HB2	1:A:195:TYR:HB3	1.95	0.48
2:B:182:GLY:HA2	2:B:235:ASP:HB2	1.96	0.47
1:A:91:TYR:HB3	1:A:192:LEU:HB2	1.95	0.47
2:B:236:SER:HB3	2:B:503:TYR:HD1	1.78	0.47
2:B:526:ILE:HG22	2:B:536:ALA:HB2	1.97	0.47
1:A:122:ILE:HG12	1:A:165:PRO:HB3	1.97	0.47
2:B:133:LEU:HD13	2:B:192:LEU:HD21	1.95	0.46
3:C:132:ARG:HG2	3:C:159:ASP:HA	1.96	0.46

Continued on next page...

Not For Manuscript Review

Continued from previous page...

Atom-1	Atom-2	Interatomic distance (Å)	Clash overlap (Å)
2:B:266:PRO:HA	2:B:461:ILE:HA	1.98	0.46
3:C:462:MET:HB3	3:C:487:THR:HB	1.97	0.46
1:A:245:THR:HB	1:A:552:ASP:HA	1.97	0.46
1:A:315:PHE:HD1	1:A:402:LYS:HB2	1.81	0.45
1:A:469:ARG:HD3	1:A:484:GLN:HB2	2.00	0.44
3:C:97:PRO:HD3	3:C:113:TRP:CE2	2.53	0.44
2:B:446:THR:HA	2:B:447:PRO:HD3	1.84	0.44
3:C:111:ALA:HB2	3:C:227:ARG:HG2	2.00	0.43
2:B:339:MET:HB2	2:B:442:ARG:HE	1.83	0.43
1:A:355:VAL:HG11	1:A:404:ILE:HD13	2.00	0.43
2:B:344:PHE:HB3	2:B:350:PRO:HB3	2.01	0.43
2:B:72:PHE:HB3	2:B:214:GLU:HB3	2.01	0.43
1:A:82:VAL:HA	1:A:198:LEU:HB3	2.00	0.42
3:C:426:ILE:HB	3:C:469:ARG:HB3	2.01	0.42
2:B:36:ALA:HB1	2:B:156:VAL:HG11	2.01	0.42
2:B:55:ILE:HD12	2:B:55:ILE:HA	1.90	0.41
1:A:98:GLN:HA	1:A:104:ALA:HB2	2.02	0.41
3:C:355:VAL:HG13	3:C:425:VAL:HG23	2.03	0.41
2:B:41:VAL:HG21	2:B:169:THR:HG23	2.02	0.41
3:C:240:ALA:HB1	3:C:555:PRO:HG3	2.03	0.40
1:A:511:GLN:HA	1:A:560:PRO:HA	2.02	0.40
3:C:50:ILE:HD11	3:C:220:ASP:HB2	2.03	0.40

There are no symmetry-related clashes.

5.3 Torsion angles (i)

5.3.1 Protein backbone (i)

In the following table, the Percentiles column shows the percent Ramachandran outliers of the chain as a percentile score with respect to all PDB entries followed by that with respect to all EM entries.

The Analysed column shows the number of residues for which the backbone conformation was analysed, and the total number of residues.

Mol	Chain	Analysed	Favoured	Allowed	Outliers	Percentiles	
1	A	530/534 (99%)	495 (93%)	34 (6%)	1 (0%)	44	44
2	B	548/550 (100%)	510 (93%)	36 (7%)	2 (0%)	30	30
3	C	548/550 (100%)	525 (96%)	23 (4%)	0	100	100

Continued on next page...

Not For Manuscript Review

Continued from previous page...

Mol	Chain	Analysed	Favoured	Allowed	Outliers	Percentiles	
All	All	1626/1634 (100%)	1530 (94%)	93 (6%)	3 (0%)	45	44

All (3) Ramachandran outliers are listed below:

Mol	Chain	Res	Type
1	A	432	ASN
2	B	227	ARG
2	B	434	ILE

5.3.2 Protein sidechains ⓘ

In the following table, the Percentiles column shows the percent sidechain outliers of the chain as a percentile score with respect to all PDB entries followed by that with respect to all EM entries.

The Analysed column shows the number of residues for which the sidechain conformation was analysed, and the total number of residues.

Mol	Chain	Analysed	Rotameric	Outliers	Percentiles	
1	A	424/424 (100%)	400 (94%)	24 (6%)	17	17
2	B	437/437 (100%)	418 (96%)	19 (4%)	25	25
3	C	438/438 (100%)	424 (97%)	14 (3%)	34	34
All	All	1299/1299 (100%)	1242 (96%)	57 (4%)	26	24

All (57) residues with a non-rotameric sidechain are listed below:

Mol	Chain	Res	Type
1	A	46	SER
1	A	92	THR
1	A	96	SER
1	A	117	MET
1	A	134	VAL
1	A	163	LEU
1	A	195	TYR
1	A	219	GLU
1	A	245	THR
1	A	257	TRP
1	A	264	LEU
1	A	267	VAL
1	A	278	TRP

Continued on next page...

Not For Manuscript Review

Continued from previous page...

Mol	Chain	Res	Type
1	A	291	ARG
1	A	301	SER
1	A	314	GLN
1	A	317	TYR
1	A	339	MET
1	A	391	THR
1	A	462	MET
1	A	512	PHE
1	A	516	GLN
1	A	524	MET
1	A	546	ASP
2	B	26	THR
2	B	42	THR
2	B	61	GLN
2	B	69	ARG
2	B	134	VAL
2	B	169	THR
2	B	227	ARG
2	B	264	LEU
2	B	267	VAL
2	B	298	ARG
2	B	299	ARG
2	B	314	GLN
2	B	315	PHE
2	B	317	TYR
2	B	325	ASP
2	B	339	MET
2	B	492	LEU
2	B	512	PHE
2	B	531	ASP
3	C	34	VAL
3	C	197	ASN
3	C	230	SER
3	C	233	THR
3	C	278	TRP
3	C	319	ASN
3	C	331	VAL
3	C	341	PHE
3	C	342	VAL
3	C	441	ASP
3	C	455	VAL
3	C	504	SER

Continued on next page...

Not For Manuscript Review

Continued from previous page...

Mol	Chain	Res	Type
3	C	541	SER
3	C	544	LEU

Sometimes sidechains can be flipped to improve hydrogen bonding and reduce clashes. All (2) such sidechains are listed below:

Mol	Chain	Res	Type
1	A	516	GLN
1	A	568	ASN

5.3.3 RNA (i)

There are no RNA molecules in this entry.

5.4 Non-standard residues in protein, DNA, RNA chains (i)

There are no non-standard protein/DNA/RNA residues in this entry.

5.5 Carbohydrates (i)

There are no oligosaccharides in this entry.

5.6 Ligand geometry (i)

There are no ligands in this entry.

5.7 Other polymers (i)

There are no such residues in this entry.

5.8 Polymer linkage issues (i)

The following chains have linkage breaks:

Mol	Chain	Number of breaks
1	A	1

All chain breaks are listed below:

Not For Manuscript Review

Model	Chain	Residue-1	Atom-1	Residue-2	Atom-2	Distance (Å)
1	A	237:ILE	C	238:SER	N	6.13

Not For Manuscript Review

6 Map visualisation (i)

This section contains visualisations of the EMDB entry D_1292145342. These allow visual inspection of the internal detail of the map and identification of artifacts.

Images derived from a raw map, generated by summing the deposited half-maps, are presented below the corresponding image components of the primary map to allow further visual inspection and comparison with those of the primary map.

6.1 Orthogonal projections (i)

6.1.1 Primary map

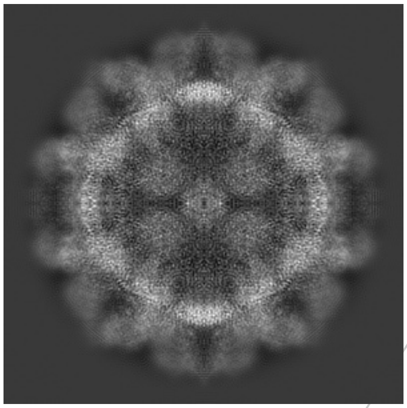

X

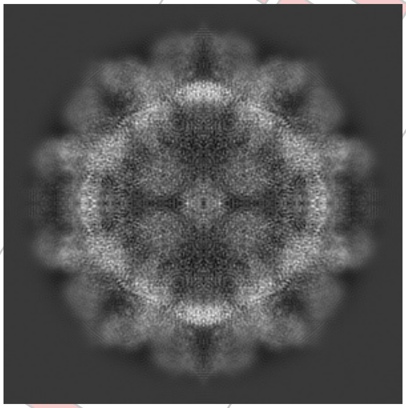

Y

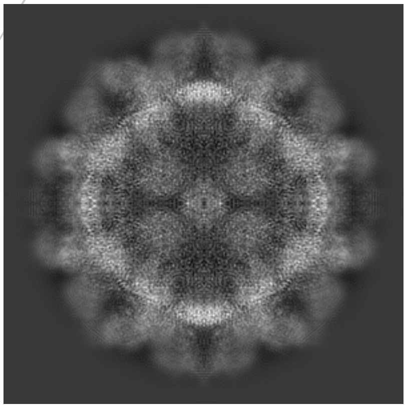

Z

6.1.2 Raw map

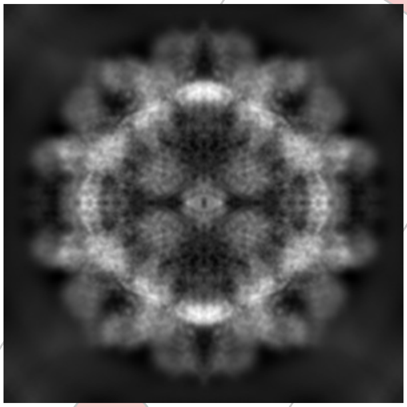

X

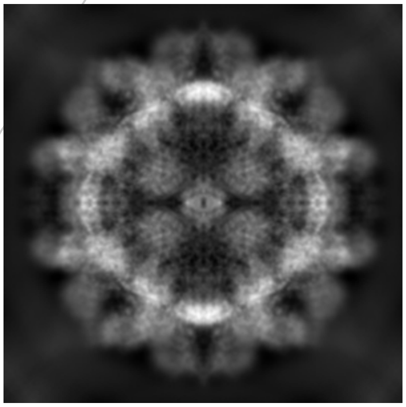

Y

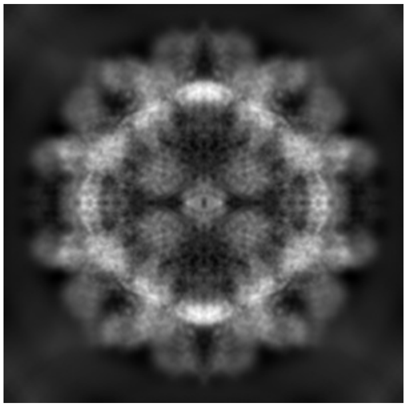

Z

The images above show the map projected in three orthogonal directions.

Not For Manuscript Review

6.2 Central slices (i)

6.2.1 Primary map

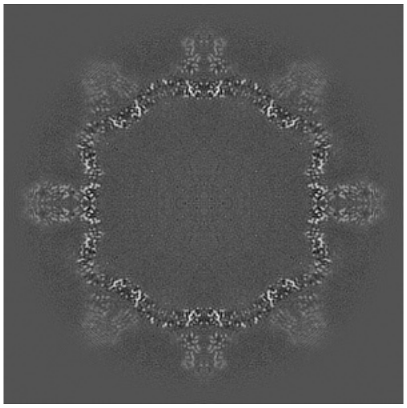
X Index: 165

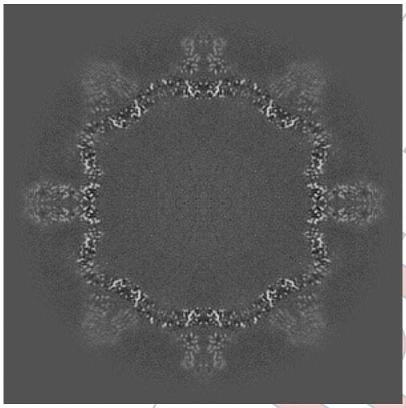
Y Index: 165

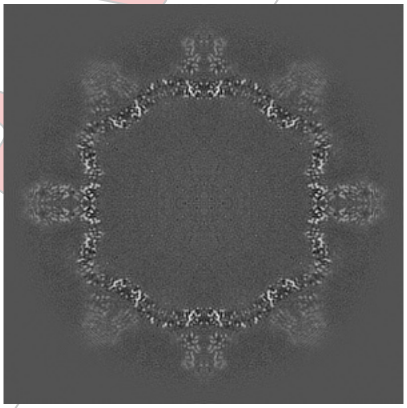
Z Index: 165

6.2.2 Raw map

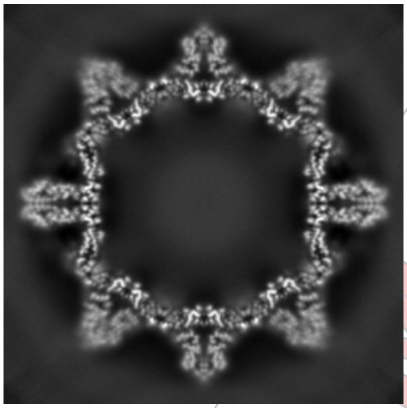
X Index: 165

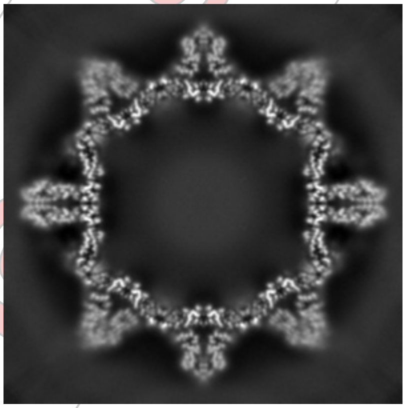
Y Index: 165

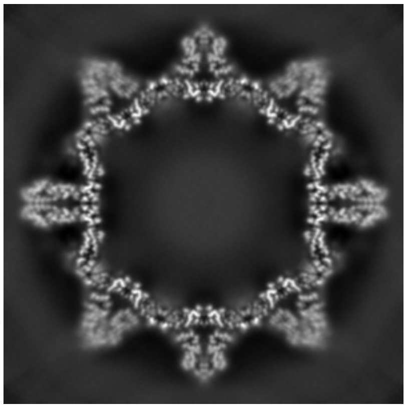
Z Index: 165

The images above show central slices of the map in three orthogonal directions.

Not For Manuscript Review

6.3 Largest variance slices

6.3.1 Primary map

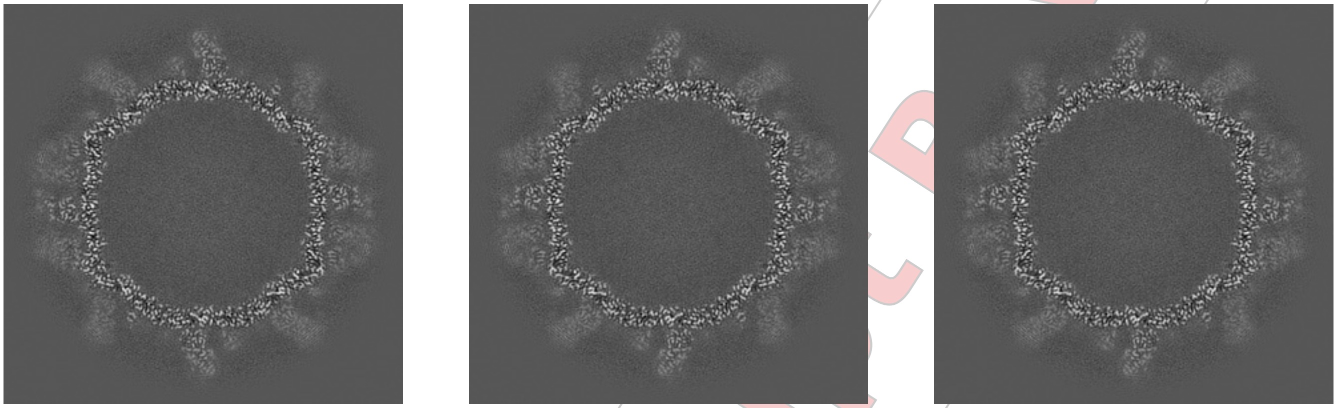

X Index: 178 Y Index: 152 Z Index: 152

6.3.2 Raw map

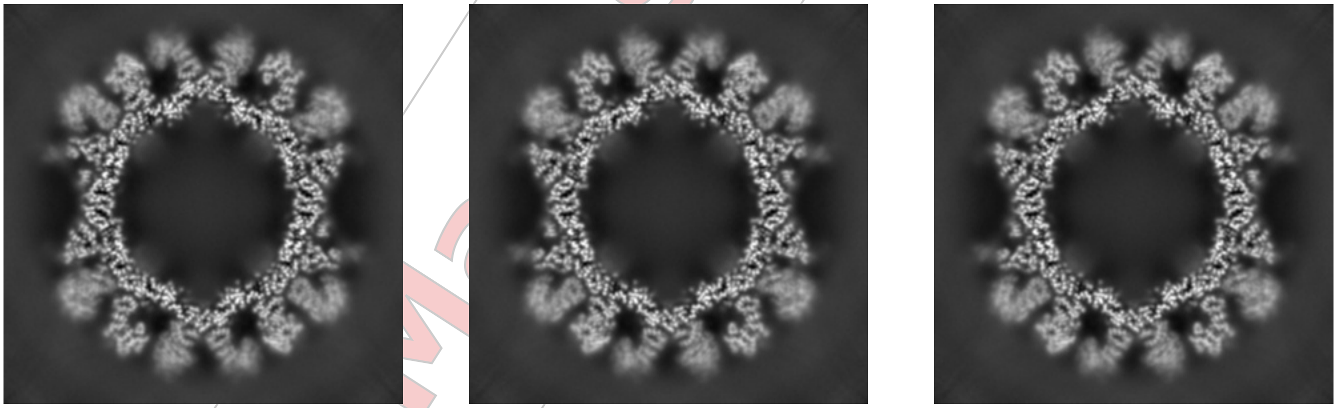

X Index: 117 Y Index: 213 Z Index: 213

The images above show the largest variance slices of the map in three orthogonal directions.

Not For Manuscript Review

6.4 Orthogonal standard-deviation projections (False-color) (i)

6.4.1 Primary map

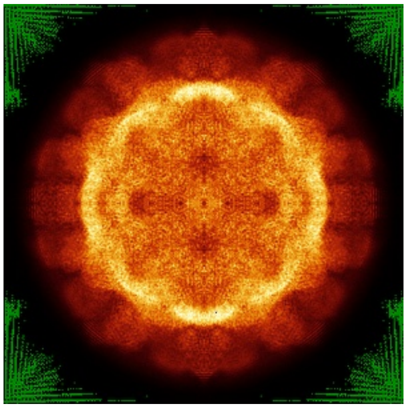
X

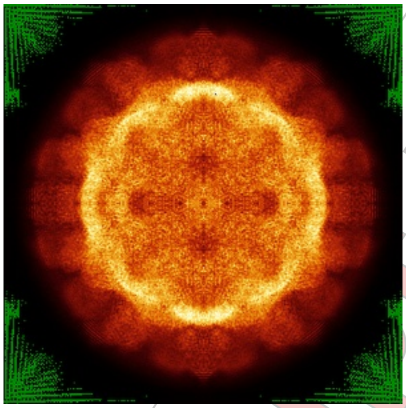
Y

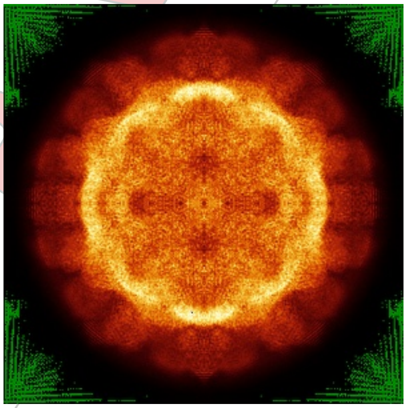
Z

6.4.2 Raw map

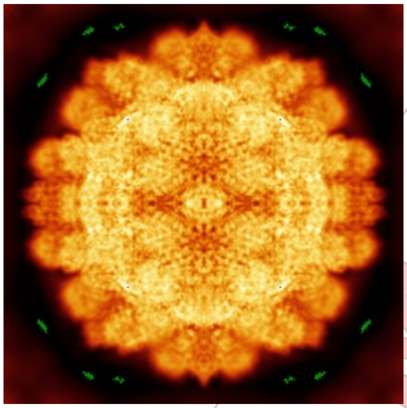
X

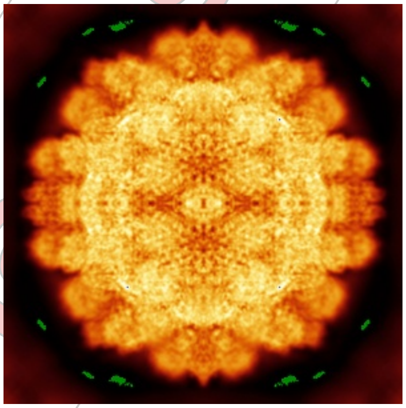
Y

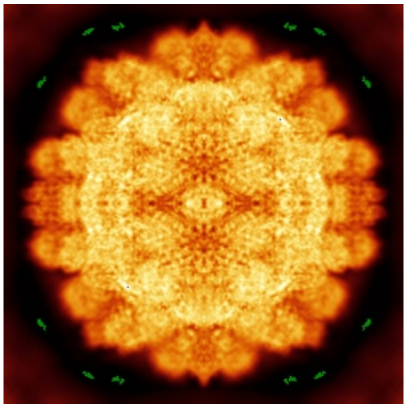
Z

The images above show the map standard deviation projections with false color in three orthogonal directions. Minimum values are shown in green, max in blue, and dark to light orange shades represent small to large values respectively.

Not For Manuscript Review

6.5 Orthogonal surface views (i)

6.5.1 Primary map

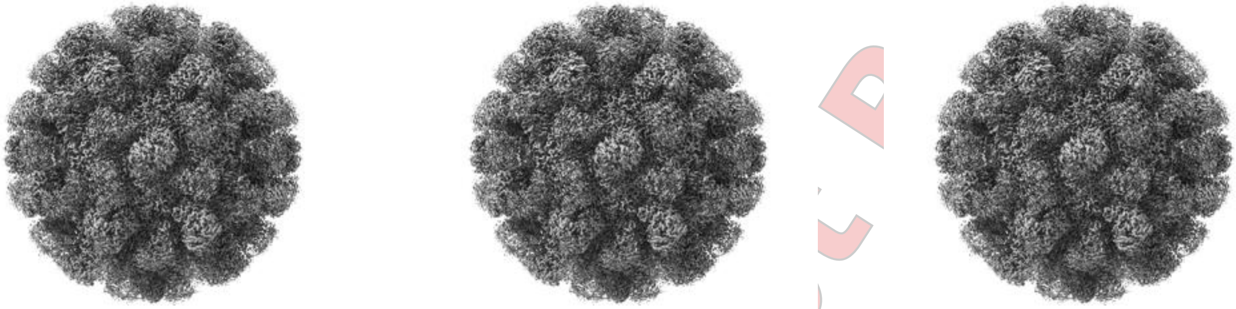

X Y Z

The images above show the 3D surface view of the map at the recommended contour level 1.2. These images, in conjunction with the slice images, may facilitate assessment of whether an appropriate contour level has been provided.

6.5.2 Raw map

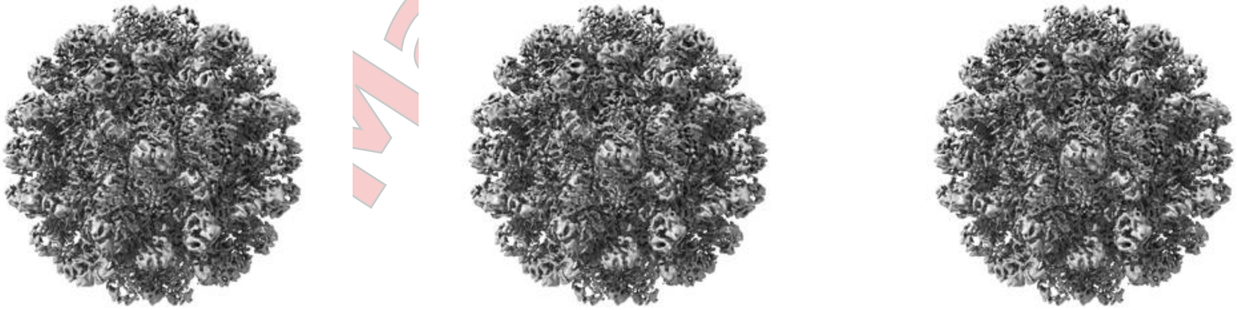

X Y Z

These images show the 3D surface of the raw map. The raw map's contour level was selected so that its surface encloses the same volume as the primary map does at its recommended contour level.

6.6 Mask visualisation (i)

This section was not generated. No masks/segmentation were deposited.

Not For Manuscript Review

7 Map analysis (i)

This section contains the results of statistical analysis of the map.

7.1 Map-value distribution (i)

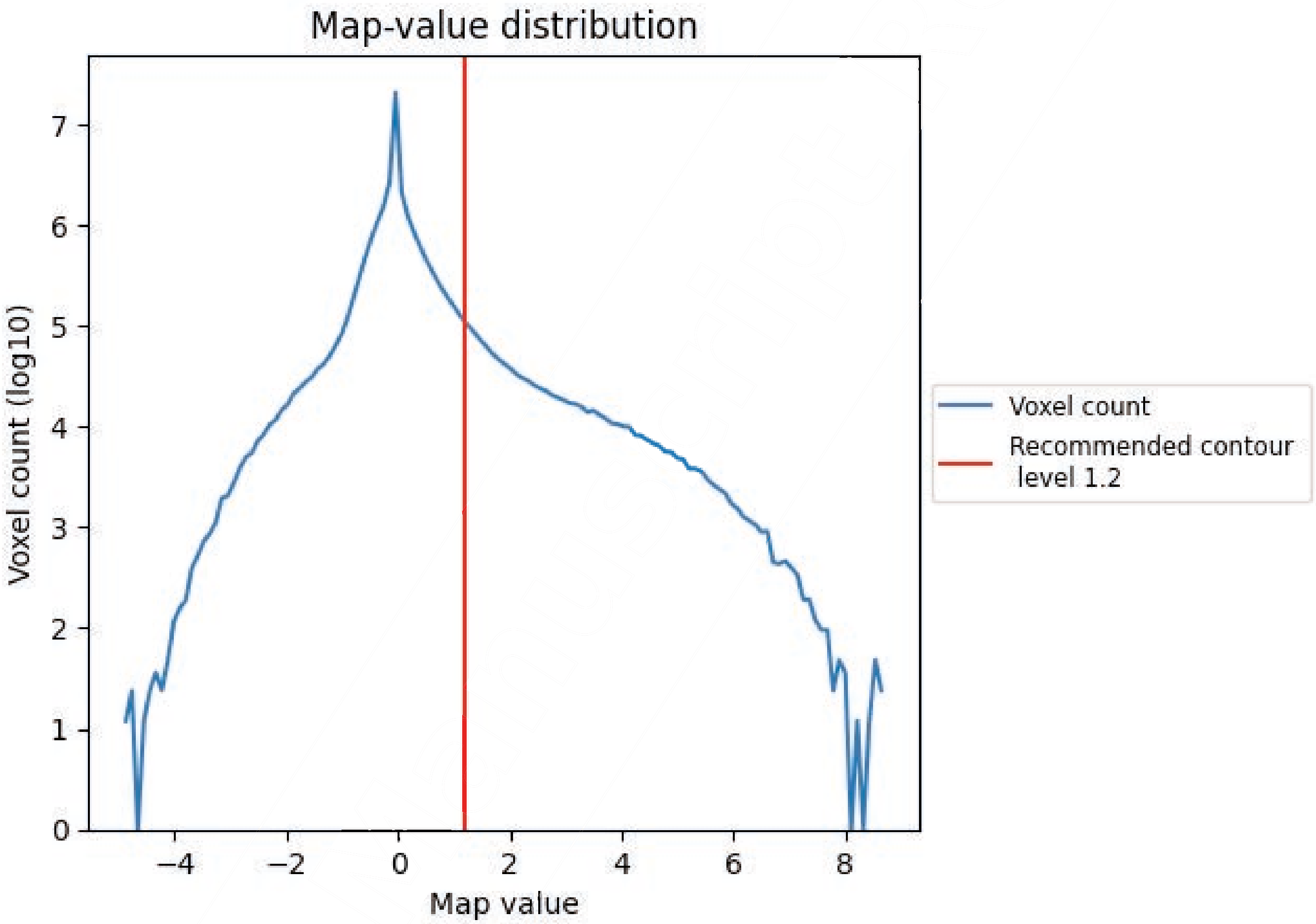

The map-value distribution is plotted in 128 intervals along the x-axis. The y-axis is logarithmic. A spike in this graph at zero usually indicates that the volume has been masked.

Not For Manuscript Review

7.2 Volume estimate ⓘ

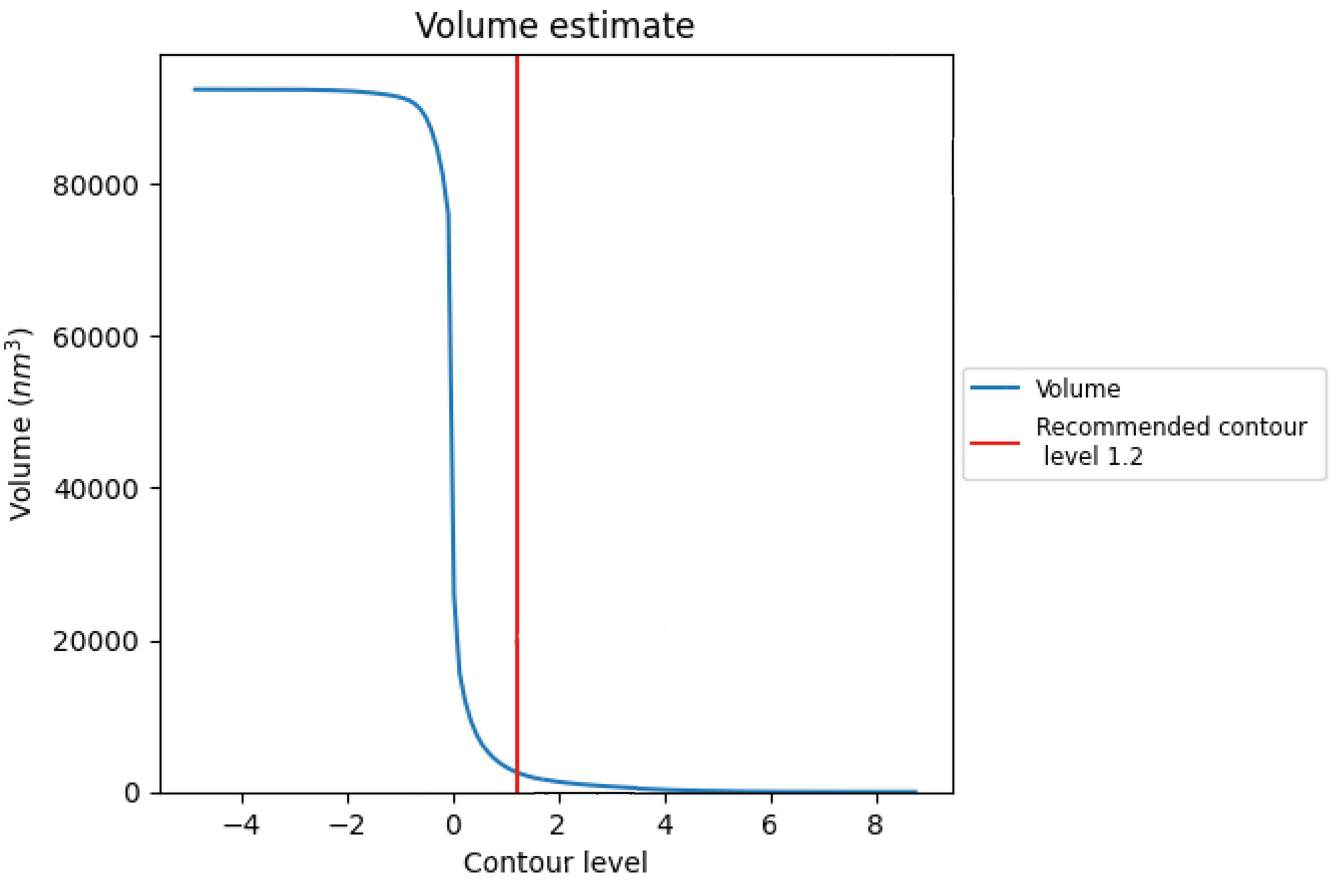

The volume at the recommended contour level is 2600 nm^3; this corresponds to an approximate mass of 2349 kDa.

The volume estimate graph shows how the enclosed volume varies with the contour level. The recommended contour level is shown as a vertical line and the intersection between the line and the curve gives the volume of the enclosed surface at the given level.

Not For Manuscript Review

7.3 Rotationally averaged power spectrum (i)

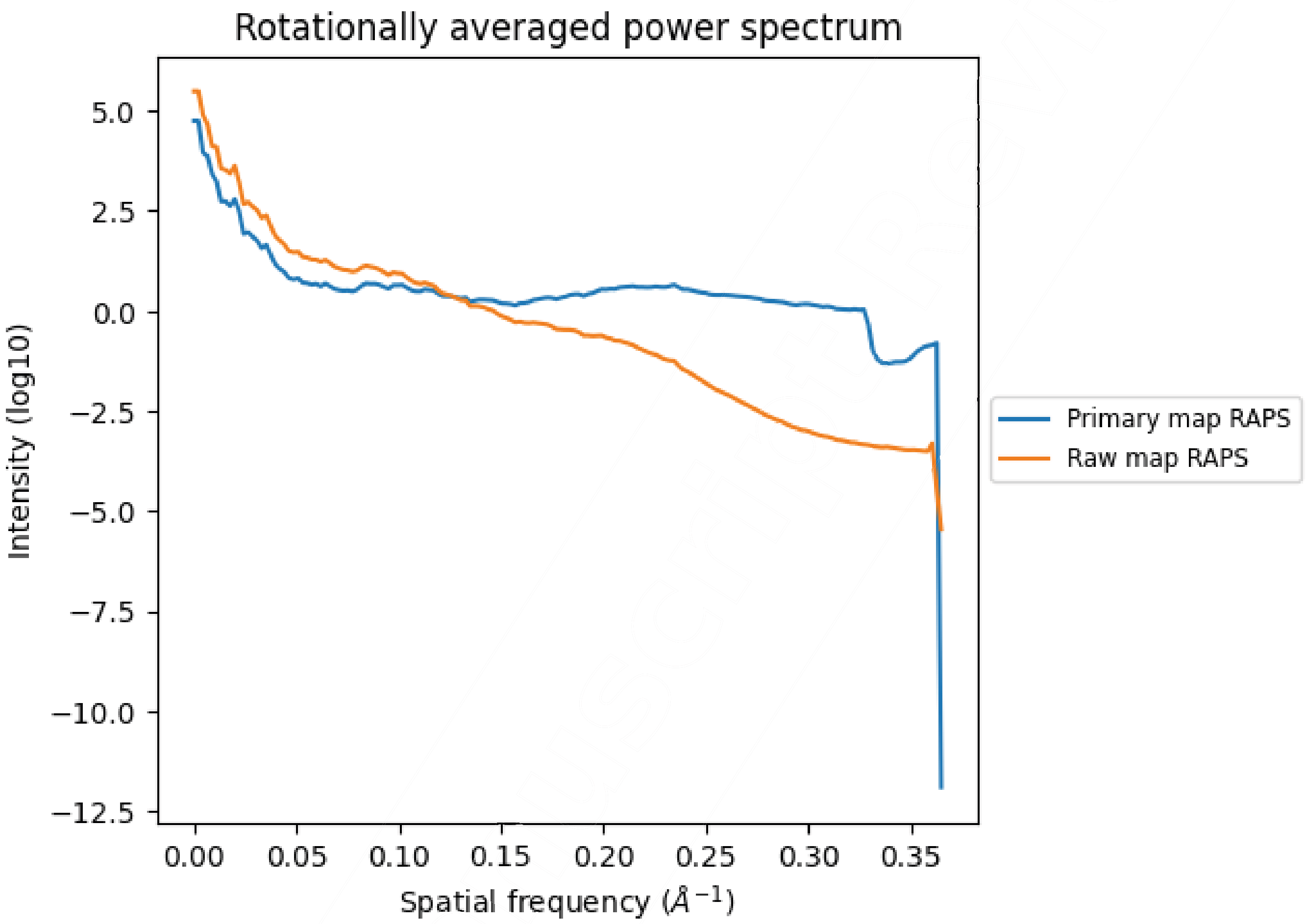

Not For Manuscript Review

8 Fourier-Shell correlation (i)

Fourier-Shell Correlation (FSC) is the most commonly used method to estimate the resolution of single-particle and subtomogram-averaged maps. The shape of the curve depends on the imposed symmetry, mask and whether or not the two 3D reconstructions used were processed from a common reference. The reported resolution is shown as a black line. A curve is displayed for the half-bit criterion in addition to lines showing the 0.143 gold standard cut-off and 0.5 cut-off.

8.1 FSC (i)

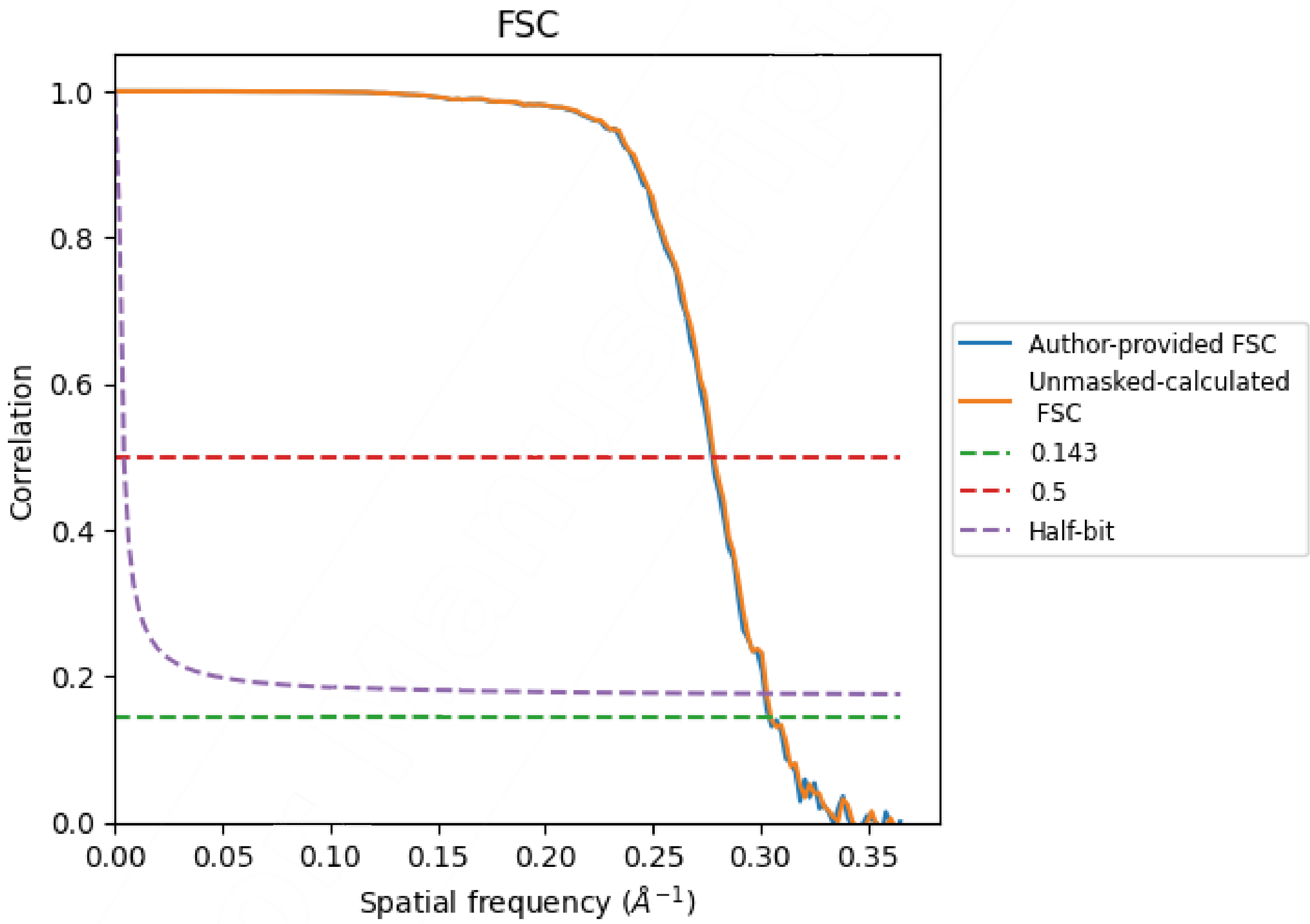

Not For Manuscript Review

8.2 Resolution estimates (i)

Resolution estimate (Å)	Estimation criterion (FSC cut-off)		
	0.143	0.5	Half-bit
Reported by author	-	-	-
Author-provided FSC curve	3.29	3.60	3.31
Unmasked-calculated*	3.28	3.59	3.30

*Resolution estimate based on FSC curve calculated by comparison of deposited half-maps.

Not For Manuscript Review

9 Map-model fit ⓘ

This section contains information regarding the fit between EMDB map D_1292145342 and PDB model D_1292145342. Per-residue inclusion information can be found in section 3 on page 4.

9.1 Map-model overlay ⓘ

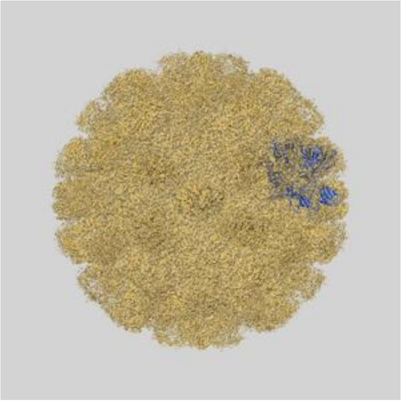

X

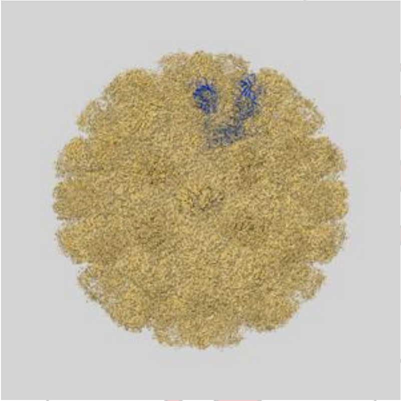

Y

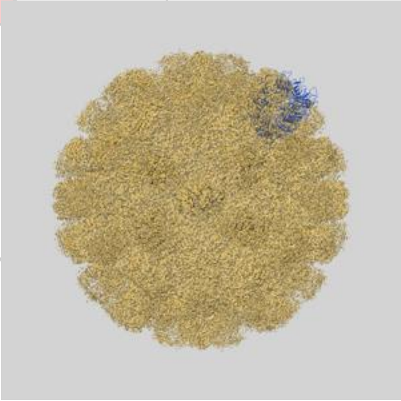

Z

The images above show the 3D surface view of the map at the recommended contour level 1.2 at 50% transparency in yellow overlaid with a ribbon representation of the model coloured in blue. These images allow for the visual assessment of the quality of fit between the atomic model and the map.

Not For Manuscript Review

9.2 Q-score mapped to coordinate model (i)

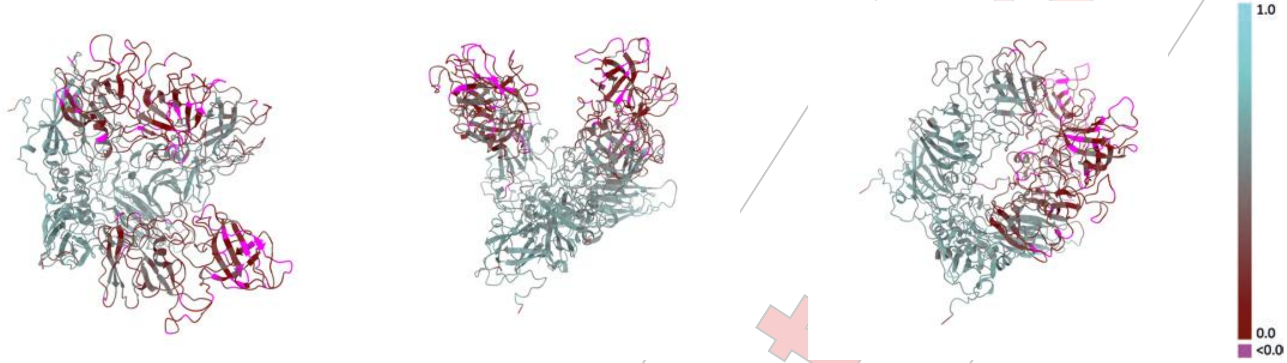

X Y Z

The images above show the model with each residue coloured according its Q-score. This shows their resolvability in the map with higher Q-score values reflecting better resolvability. Please note: Q-score is calculating the resolvability of atoms, and thus high values are only expected at resolutions at which atoms can be resolved. Low Q-score values may therefore be expected for many entries.

9.3 Atom inclusion mapped to coordinate model (i)

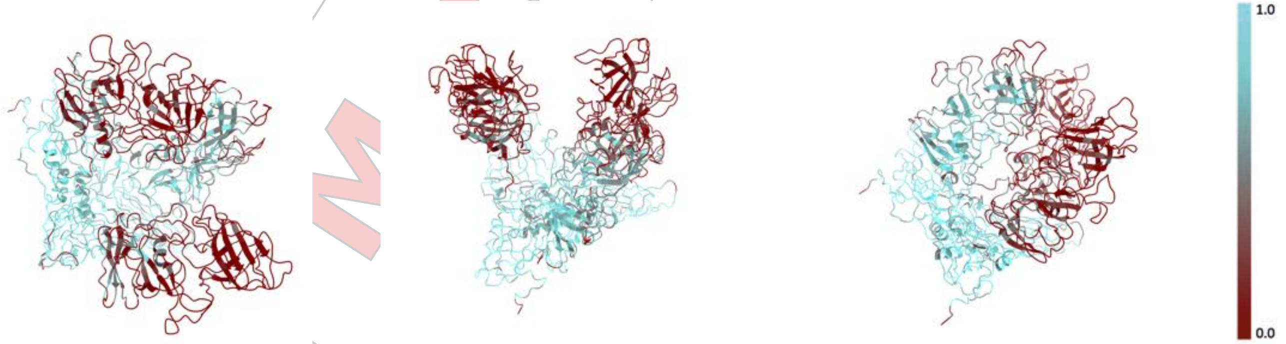

X Y Z

The images above show the model with each residue coloured according to its atom inclusion. This shows to what extent they are inside the map at the recommended contour level (1.2).

Not For Manuscript Review

9.4 Atom inclusion (i)

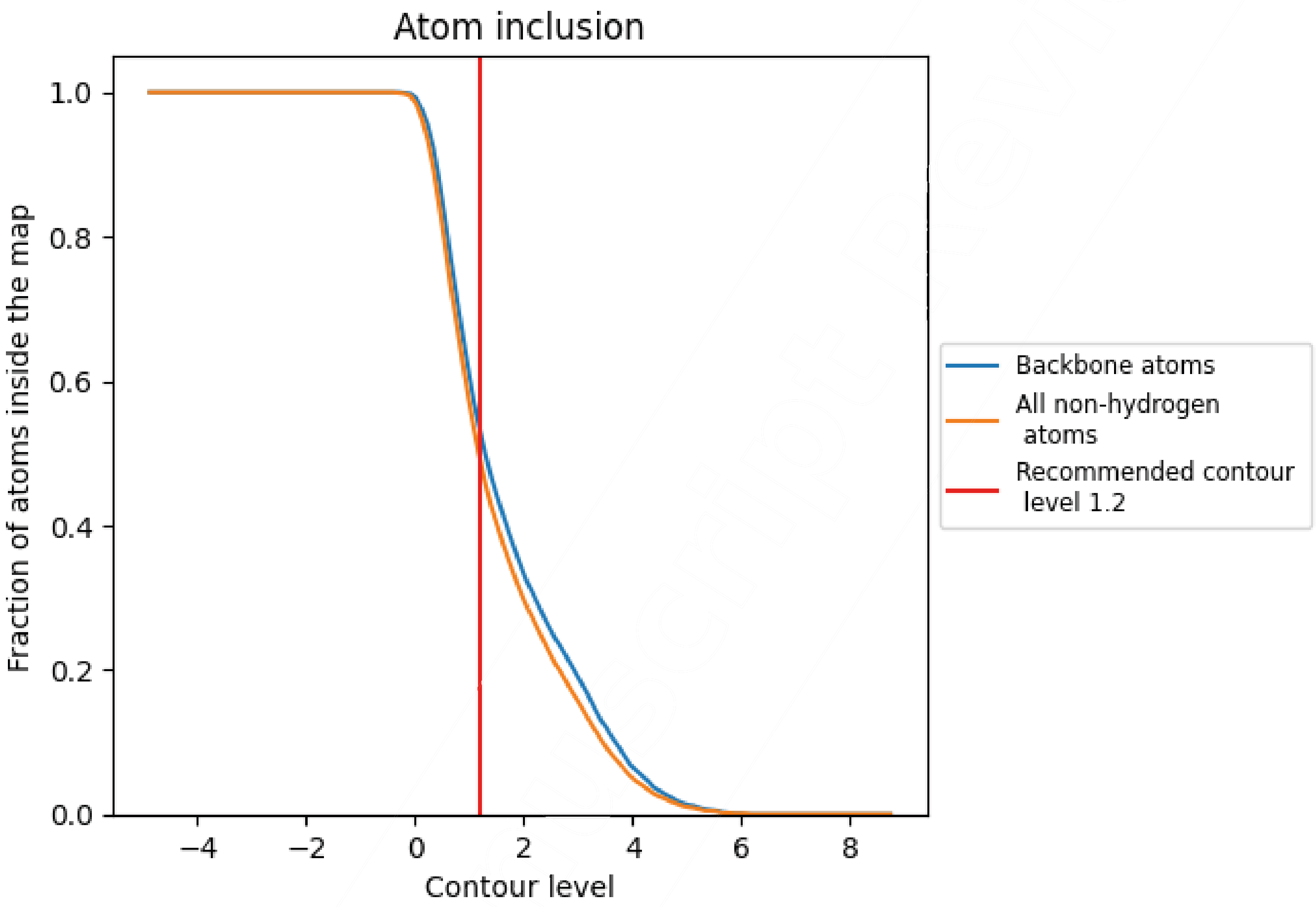

At the recommended contour level, 53% of all backbone atoms, 49% of all non-hydrogen atoms, are inside the map.

Not For Manuscript Review

9.5 Map-model fit summary (i)

The table lists the average atom inclusion at the recommended contour level (1.2) and Q-score for the entire model and for each chain.

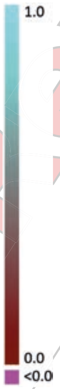

Chain	Atom inclusion	Q-score
All	0.4910	0.4140
A	0.3850	0.3550
B	0.3880	0.3500
C	0.6970	0.5350

Not For Manuscript Review

Preliminary Full wwPDB EM Validation Report ⓘ

Jan 22, 2025 – 05:50 pm GMT

Deposition ID : D_1292144858

This wwPDB validation report is NOT for manuscript review

This is a Preliminary Full wwPDB EM Validation Report.

This report is produced by the wwPDB Deposition System during initial deposition but before annotation of the structure.

We welcome your comments at *validation@mail.wwpdb.org*
A user guide is available at
https://www.wwpdb.org/validation/2017/EMValidationReportHelp
with specific help available everywhere you see the ⓘ symbol.

The types of validation reports are described at
http://www.wwpdb.org/validation/2017/FAQs#types.

The following versions of software and data (see references ⓘ) were used in the production of this report:

EMDB validation analysis	:	0.0.1.dev113
MolProbity	:	4.02b-467
Percentile statistics	:	20231227.v01 (using entries in the PDB archive December 27th 2023)
MapQ	:	1.9.13
Ideal geometry (proteins)	:	Engh & Huber (2001)
Ideal geometry (DNA, RNA)	:	Parkinson et al. (1996)
Validation Pipeline (wwPDB-VP)	:	2.40

Not For Manuscript Review

1 Overall quality at a glance (i)

The following experimental techniques were used to determine the structure:
ELECTRON MICROSCOPY

The reported resolution of this entry is 2.50 Å.

Percentile scores (ranging between 0-100) for global validation metrics of the entry are shown in the following graphic. The table shows the number of entries on which the scores are based.

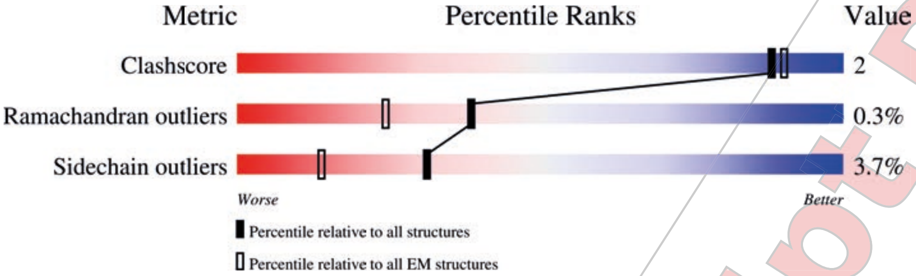

Metric	Whole archive (#Entries)	EM structures (#Entries)
Clashscore	210492	15764
Ramachandran outliers	207382	16835
Sidechain outliers	206894	16415

The table below summarises the geometric issues observed across the polymeric chains and their fit to the map. The red, orange, yellow and green segments of the bar indicate the fraction of residues that contain outliers for >=3, 2, 1 and 0 types of geometric quality criteria respectively. A grey segment represents the fraction of residues that are not modelled. The numeric value for each fraction is indicated below the corresponding segment, with a dot representing fractions <=5% The upper red bar (where present) indicates the fraction of residues that have poor fit to the EM map (all-atom inclusion < 40%). The numeric value is given above the bar.

Mol	Chain	Length	Quality of chain
1	A	534	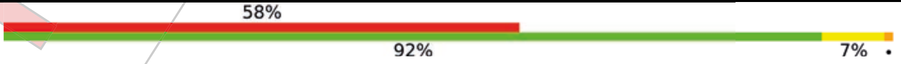
2	B	550	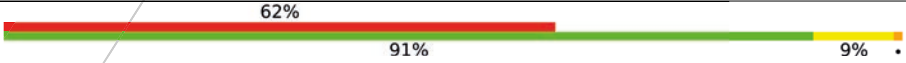
3	C	550	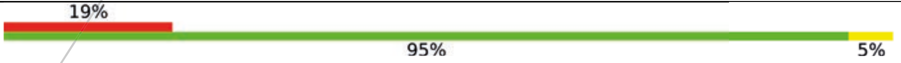
4	D	12	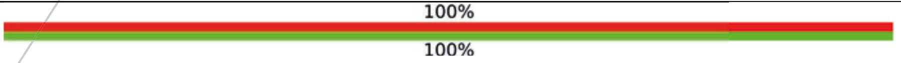

Not For Manuscript Review

2 Entry composition (i)

There are 4 unique types of molecules in this entry. The entry contains 12154 atoms, of which 0 are hydrogens and 0 are deuteriums.

In the tables below, the AltConf column contains the number of residues with at least one atom in alternate conformation and the Trace column contains the number of residues modelled with at most 2 atoms.

- Molecule 1 is a protein.

Mol	Chain	Residues	Atoms					AltConf	Trace
1	A	534	Total<br>3950	C<br>2508	N<br>669	O<br>762	S<br>11	0	0

- Molecule 2 is a protein.

Mol	Chain	Residues	Atoms					AltConf	Trace
2	B	550	Total<br>4055	C<br>2571	N<br>685	O<br>787	S<br>12	0	0

- Molecule 3 is a protein.

Mol	Chain	Residues	Atoms					AltConf	Trace
3	C	550	Total<br>4056	C<br>2572	N<br>685	O<br>787	S<br>12	0	0

- Molecule 4 is a protein called ALA-ALA-GLU-PHE-PHE-GLU-GLY-MET-VAL-HIS-ASP-SER.

Mol	Chain	Residues	Atoms					AltConf	Trace
4	D	12	Total<br>93	C<br>59	N<br>14	O<br>19	S<br>1	0	0

Not For Manuscript Review

3 Residue-property plots (i)

These plots are drawn for all protein, RNA, DNA and oligosaccharide chains in the entry. The first graphic for a chain summarises the proportions of the various outlier classes displayed in the second graphic. The second graphic shows the sequence view annotated by issues in geometry and atom inclusion in map density. Residues are color-coded according to the number of geometric quality criteria for which they contain at least one outlier: green = 0, yellow = 1, orange = 2 and red = 3 or more. A red diamond above a residue indicates a poor fit to the EM map for this residue (all-atom inclusion < 40%). Stretches of 2 or more consecutive residues without any outlier are shown as a green connector. Residues present in the sample, but not in the model, are shown in grey.

- Molecule 1:

Chain A:

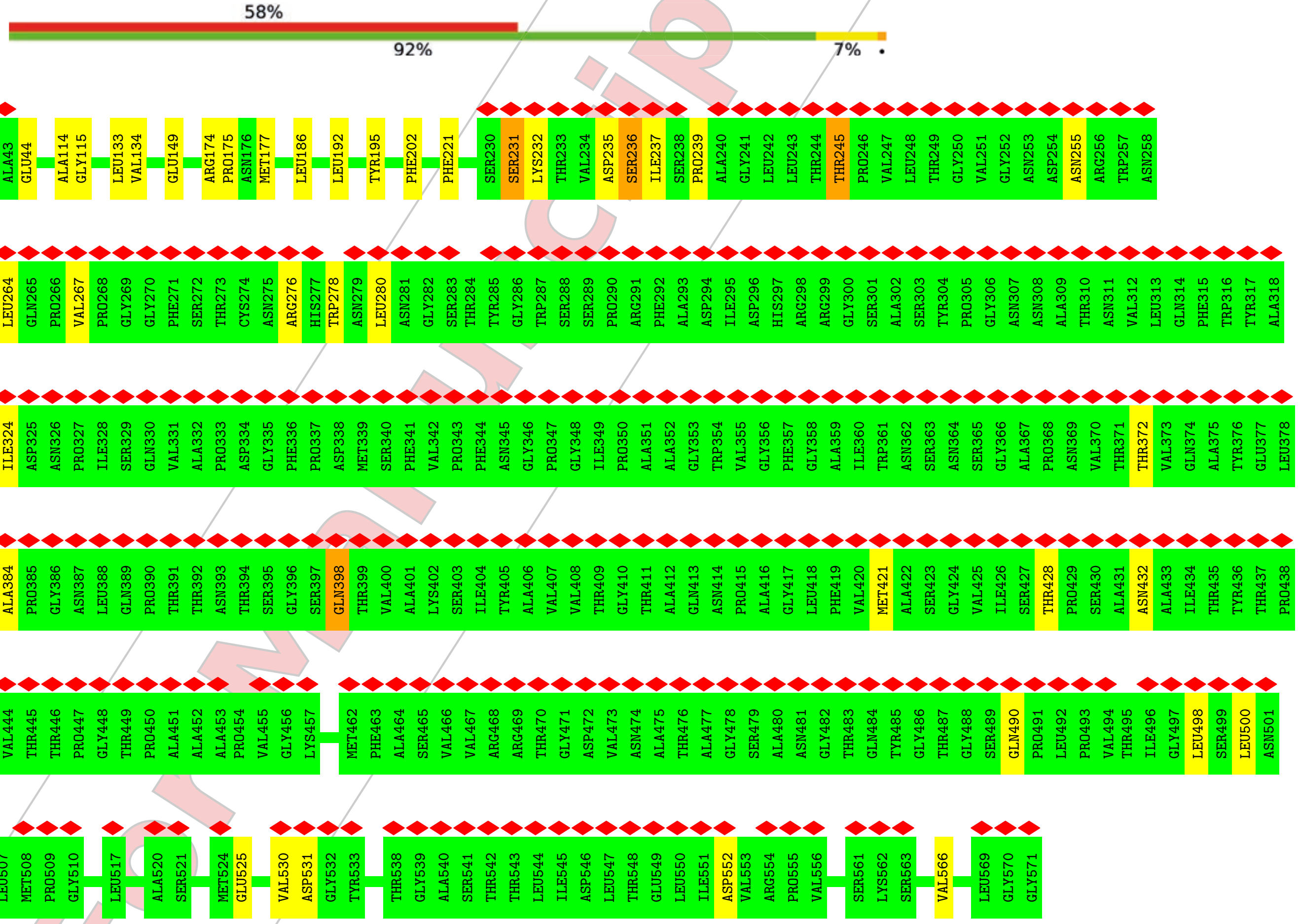

- Molecule 2:

Chain B:

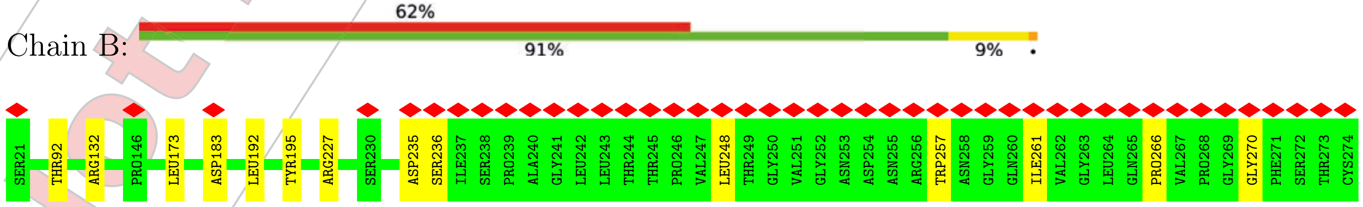

Not For Manuscript Review

ASN275 ARG276 HIS277 TRP278 ASN279 LEU280 ASN281 GLY282 SER283 THR284 TYR285 GLY286 TRP287 SER288 SER289 PRO290 ARG291 PHE292 ALA293 ASP294 ILE295 ASP296 HIS297 ARG298 ARG299 GLY300 SER301 ALA302 SER303 TYR304 PRO305 GLY306 ASN307 ASN308 ALA309 THR310 ASN311 VAL312 LEU313 GLN314 PHE315 TRP316 TYR317 ALA318 ASN319 ALA320 GLY321 SER322 ALA323 ILE324 ASP325 ASN326 PRO327 ILE328 SER329 GLN330 VAL331 ALA332 PRO333 ASP334

GLY335 PHE336 PRO337 ASP338 MET339 SER340 PHE341 VAL342 PRO343 PHE344 ASN345 GLY346 PRO347 GLY348 ILE349 PRO350 ALA351 ALA352 GLY353 TRP354 VAL355 GLY356 PHE357 GLY358 ALA359 ILE360 TRP361 ASN362 SER363 ASN364 SER365 GLY366 ALA367 PRO368 ASN369 VAL370 THR371 THR372 VAL373 GLN374 ALA375 TYR376 GLU377 LEU378 GLY379 PHE380 ALA381 THR382 GLY383 ALA384 PRO385 GLY386 ASN387 LEU388 GLN389 PRO390 THR391 THR392 ASN393 THR394

SER395 GLY396 SER397 GLN398 THR399 VAL400 ALA401 LYS402 SER403 ILE404 TYR405 ALA406 VAL407 VAL408 THR409 GLY410 THR411 ALA412 GLN413 ASN414 PRO415 ALA416 GLY417 LEU418 PHE419 VAL420 MET421 ALA422 SER423 GLY424 VAL425 ILE426 SER427 THR428 PRO429 SER430 ALA431 ASN432 ALA433 ILE434 THR435 TYR436 THR437 PRO438 GLN439 PRO440 ASP441 ARG442 ILE443 VAL444 THR445 THR446 PRO447 GLY448 THR449 PRO450 ALA451 ALA452 ALA453 PRO454

VAL455 GLY456 LYS457 ASN458 THR459 PRO460 ILE461 MET462 PHE463 ALA464 SER465 VAL466 VAL467 ARG468 ARG469 THR470 GLY471 ASP472 VAL473 ASN474 ALA475 THR476 ALA477 GLY478 SER479 ALA480 ASN481 GLY482 THR483 GLN484 TYR485 GLY486 THR487 GLY488 SER489 GLN490 PRO491 LEU492 PRO493 VAL494 THR495 ILE496 GLY497 LEU498 SER499 LEU500 ASN501 ASN502 TYR503 SER504 SER505 ALA506 LEU507 MET508 PRO509 GLY510 GLN511 PHE512 PHE513 VAL514

TRP515 GLN516 LEU517 THR518 PHE519 ALA520 SER521 GLY522 PHE523 MET524 GLU525 ILE526 GLY527 LEU528 SER529 VAL530 ASP531 GLY532 TYR533 PHE534 TYR535 ALA536 GLY537 THR538 GLY539 ALA540 SER541 GLY542 THR543 LEU544 ILE545 ASP546 LEU547 THR548 GLU549 LEU550 ILE551 ASP552 VAL553 ARG554 PRO555 VAL556 GLY557 PRO558 ARG559 PRO560 SER561 LYS562 SER563 THR564 LEU565 VAL566 PHE567 ASN568 LEU569 GLY570

- Molecule 3:

Chain C:

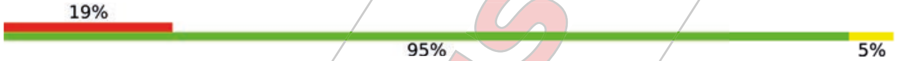

SER21 VAL22 ALA36 GLN65 ILE168 GLU222 PHE223 VAL224 LYS232 ASP235 LEU242 TRP278 ALA293 ASP294 ILE295 ASP296 HIS297 ARG298 ARG299 GLY300 SER301 ALA302 SER303 TYR304 PRO305 GLY306 ASN307 ASN308 ALA309 THR310 ASN311 VAL312 LEU313 GLN314 PHE315 TRP316 TYR317 ALA318 ASN319 SER322 ALA323 ILE324 ASP325 ASN326 PRO327 ILE328 PHE341 VAL342 PRO343

PHE344 ASN345 GLY346 PRO347 GLY348 ILE349 PRO350 ALA351 ALA352 GLY353 GLY356 PHE357 ILE360 TRP361 ASN362 SER363 ASN364 SER365 GLY366 ALA367 PRO368 ASN369 VAL370 THR371 THR372 VAL373 GLN374 ALA375 TYR376 GLU377 THR382 GLY383 ALA384 PRO385 GLY386 ASN387 LEU388 GLN389 ASN393 THR394 SER395 GLN398 LYS402 SER403 ILE404 TYR405 ALA406 VAL407 VAL408 THR409 GLY410 THR411 ALA412 GLN413

ASN414 PRO415 ALA416 VAL420 MET421 ALA422 SER423 ILE426 SER427 THR428 PRO429 SER430 ALA431 ASN432 THR437 PRO447 ARG468 GLY471 ASP472 ALA475 THR476 ALA477 GLY478 SER479 ALA480 ASN481 GLY482 GLY497 LEU517 ALA520 SER521 SER541 ASP546 GLU549 LEU550 SER561 GLY570

- Molecule 4: ALA-ALA-GLU-PHE-PHE-GLU-GLY-MET-VAL-HIS-ASP-SER

Chain D:

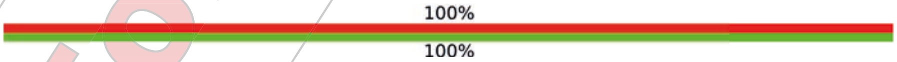

ALA173 ALA174 GLU175 PHE176 PHE177 GLU178 GLY179 MET180 VAL181 HIS182 ASP183 SER184

Not For Manuscript Review

4 Experimental information (i)

Property	Value	Source
EM reconstruction method	SINGLE PARTICLE	Depositor
Imposed symmetry	POINT, Not provided	
Number of particles used	Not provided	
Resolution determination method	Not provided	
CTF correction method	Not provided	
Microscope	Not provided	
Voltage (kV)	Not provided	
Electron dose ($e^-/\text{Å}^2$)	Not provided	
Minimum defocus (nm)	Not provided	
Maximum defocus (nm)	Not provided	
Magnification	Not provided	
Image detector	Not provided	
Maximum map value	6.794	Depositor
Minimum map value	-3.762	Depositor
Average map value	0.039	Depositor
Map value standard deviation	0.390	Depositor
Recommended contour level	1.1	Depositor
Map size (Å)	475.2, 475.2, 475.2	wwPDB
Map dimensions	440, 440, 440	wwPDB
Map angles (°)	90.0, 90.0, 90.0	wwPDB
Pixel spacing (Å)	1.08, 1.08, 1.08	Depositor

Not For Manuscript Review

5 Model quality (i)

5.1 Standard geometry (i)

The Z score for a bond length (or angle) is the number of standard deviations the observed value is removed from the expected value. A bond length (or angle) with $|Z| > 5$ is considered an outlier worth inspection. RMSZ is the root-mean-square of all Z scores of the bond lengths (or angles).

Mol	Chain	Bond lengths		Bond angles	
		RMSZ	#$\|Z\|$ >5	RMSZ	#$\|Z\|$ >5
1	A	0.31	0/4063	0.57	1/5582 (0.0%)
2	B	0.30	0/4170	0.59	0/5732
3	C	0.28	0/4171	0.54	0/5733
4	D	0.28	0/95	0.38	0/126
All	All	0.30	0/12499	0.57	1/17173 (0.0%)

There are no bond length outliers.

All (1) bond angle outliers are listed below:

Mol	Chain	Res	Type	Atoms	Z	Observed(^{o})	Ideal(^{o})
1	A	498	LEU	CA-CB-CG	6.10	129.33	115.30

There are no chirality outliers.

There are no planarity outliers.

5.2 Too-close contacts (i)

In the following table, the Non-H and H(model) columns list the number of non-hydrogen atoms and hydrogen atoms in the chain respectively. The H(added) column lists the number of hydrogen atoms added and optimized by MolProbity. The Clashes column lists the number of clashes within the asymmetric unit, whereas Symm-Clashes lists symmetry-related clashes.

Mol	Chain	Non-H	H(model)	H(added)	Clashes	Symm-Clashes
1	A	3950	0	3818	16	0
2	B	4055	0	3918	19	0
3	C	4056	0	3920	8	0
4	D	93	0	76	0	0
All	All	12154	0	11732	43	0

The all-atom clashscore is defined as the number of clashes found per 1000 atoms (including hydrogen atoms). The all-atom clashscore for this structure is 2.

Not For Manuscript Review

All (43) close contacts within the same asymmetric unit are listed below, sorted by their clash magnitude.

Atom-1	Atom-2	Interatomic distance (Å)	Clash overlap (Å)
2:B:462:MET:HG2	2:B:487:THR:HB	1.83	0.60
3:C:352:ALA:HB1	3:C:437:THR:HG23	1.84	0.59
1:A:255:ASN:HB3	1:A:276:ARG:HB2	1.88	0.56
2:B:355:VAL:HG13	2:B:425:VAL:HG23	1.90	0.53
2:B:477:ALA:HA	2:B:483:THR:HG21	1.91	0.53
1:A:133:LEU:HD13	1:A:192:LEU:HD11	1.92	0.52
1:A:239:PRO:HD3	1:A:506:ALA:HB1	1.93	0.51
2:B:353:GLY:H	2:B:442:ARG:HH12	1.58	0.50
3:C:242:LEU:HD21	3:C:497:GLY:HA3	1.95	0.48
1:A:235:ASP:O	1:A:236:SER:C	2.51	0.48
1:A:231:SER:O	1:A:232:LYS:C	2.52	0.48
2:B:270:GLY:HA2	2:B:460:PRO:HG2	1.96	0.48
2:B:248:LEU:HA	2:B:261:ILE:HD11	1.96	0.47
2:B:374:GLN:HE21	2:B:405:TYR:HB3	1.80	0.47
2:B:132:ARG:HB2	2:B:195:TYR:HB3	1.96	0.47
2:B:514:VAL:HG13	2:B:556:VAL:HB	1.98	0.46
1:A:114:ALA:HB1	1:A:174:ARG:HH21	1.79	0.46
1:A:280:LEU:HD12	1:A:530:VAL:HG23	1.97	0.46
2:B:405:TYR:HD1	2:B:406:ALA:H	1.63	0.46
1:A:245:THR:HG22	1:A:552:ASP:HA	1.97	0.46
2:B:280:LEU:HG	2:B:503:TYR:HB2	1.98	0.45
2:B:431:ALA:HA	2:B:434:ILE:HD11	1.98	0.45
3:C:341:PHE:HE2	3:C:388:LEU:HD13	1.82	0.45
3:C:517:LEU:HB3	3:C:550:LEU:HD11	1.98	0.45
3:C:413:GLN:HG2	3:C:415:PRO:HD2	1.97	0.45
1:A:115:GLY:HA3	1:A:221:PHE:HA	1.99	0.44
1:A:175:PRO:HG2	1:A:186:LEU:HD12	2.00	0.44
3:C:36:ALA:HB3	3:C:168:ILE:HG22	2.00	0.43
1:A:267:VAL:HG21	1:A:440:PRO:HG3	2.01	0.43
2:B:402:LYS:HE3	2:B:402:LYS:HB2	1.73	0.43
1:A:525:GLU:HB2	1:A:566:VAL:HG22	2.01	0.42
1:A:324:ILE:H	1:A:324:ILE:HG13	1.72	0.41
1:A:384:ALA:HB3	1:A:398:GLN:HE22	1.86	0.41
2:B:314:GLN:HE22	2:B:410:GLY:HA2	1.86	0.41
3:C:349:ILE:HB	3:C:429:PRO:HA	2.03	0.41
1:A:235:ASP:O	1:A:237:ILE:N	2.54	0.41
1:A:174:ARG:HA	1:A:175:PRO:HD3	1.97	0.41
2:B:352:ALA:N	2:B:427:SER:O	2.54	0.40
2:B:235:ASP:HB3	2:B:236:SER:H	1.60	0.40
2:B:464:ALA:HB1	2:B:485:TYR:HD2	1.87	0.40

Continued on next page...

Not For Manuscript Review

Continued from previous page...

Atom-1	Atom-2	Interatomic distance (Å)	Clash overlap (Å)
2:B:266:PRO:HG3	2:B:538:THR:HG21	2.02	0.40
2:B:496:ILE:HD13	2:B:496:ILE:HA	1.93	0.40
3:C:411:THR:HG22	3:C:413:GLN:H	1.87	0.40

There are no symmetry-related clashes.

5.3 Torsion angles (i)

5.3.1 Protein backbone (i)

In the following table, the Percentiles column shows the percent Ramachandran outliers of the chain as a percentile score with respect to all PDB entries followed by that with respect to all EM entries.

The Analysed column shows the number of residues for which the backbone conformation was analysed, and the total number of residues.

Mol	Chain	Analysed	Favoured	Allowed	Outliers	Percentiles	
1	A	532/534 (100%)	486 (91%)	43 (8%)	3 (1%)	22	39
2	B	548/550 (100%)	509 (93%)	37 (7%)	2 (0%)	30	49
3	C	548/550 (100%)	515 (94%)	33 (6%)	0	100	100
4	D	10/12 (83%)	10 (100%)	0	0	100	100
All	All	1638/1646 (100%)	1520 (93%)	113 (7%)	5 (0%)	38	56

All (5) Ramachandran outliers are listed below:

Mol	Chain	Res	Type
1	A	236	SER
2	B	227	ARG
2	B	434	ILE
1	A	231	SER
1	A	432	ASN

5.3.2 Protein sidechains (i)

In the following table, the Percentiles column shows the percent sidechain outliers of the chain as a percentile score with respect to all PDB entries followed by that with respect to all EM entries.

Not For Manuscript Review

The Analysed column shows the number of residues for which the sidechain conformation was analysed, and the total number of residues.

Mol	Chain	Analysed	Rotameric	Outliers	Percentiles	
1	A	425/425 (100%)	409 (96%)	16 (4%)	28	53
2	B	438/438 (100%)	420 (96%)	18 (4%)	26	50
3	C	438/438 (100%)	423 (97%)	15 (3%)	32	58
4	D	9/9 (100%)	9 (100%)	0	100	100
All	All	1310/1310 (100%)	1261 (96%)	49 (4%)	31	55

All (49) residues with a non-rotameric sidechain are listed below:

Mol	Chain	Res	Type
1	A	44	GLU
1	A	134	VAL
1	A	149	GLU
1	A	177	MET
1	A	195	TYR
1	A	202	PHE
1	A	245	THR
1	A	264	LEU
1	A	278	TRP
1	A	372	THR
1	A	398	GLN
1	A	421	MET
1	A	428	THR
1	A	490	GLN
1	A	500	LEU
1	A	531	ASP
2	B	92	THR
2	B	173	LEU
2	B	183	ASP
2	B	192	LEU
2	B	257	TRP
2	B	291	ARG
2	B	297	HIS
2	B	299	ARG
2	B	308	ASN
2	B	315	PHE
2	B	374	GLN
2	B	405	TYR
2	B	428	THR
2	B	436	TYR

Continued on next page...

Not For Manuscript Review

Continued from previous page...

Mol	Chain	Res	Type
2	B	462	MET
2	B	505	SER
2	B	512	PHE
2	B	567	PHE
3	C	65	GLN
3	C	222	GLU
3	C	224	VAL
3	C	235	ASP
3	C	278	TRP
3	C	326	ASN
3	C	342	VAL
3	C	343	PRO
3	C	360	ILE
3	C	395	SER
3	C	407	VAL
3	C	409	THR
3	C	420	VAL
3	C	541	SER
3	C	561	SER

Sometimes sidechains can be flipped to improve hydrogen bonding and reduce clashes. All (3) such sidechains are listed below:

Mol	Chain	Res	Type
2	B	369	ASN
2	B	374	GLN
2	B	393	ASN

5.3.3 RNA ⓘ

There are no RNA molecules in this entry.

5.4 Non-standard residues in protein, DNA, RNA chains ⓘ

There are no non-standard protein/DNA/RNA residues in this entry.

5.5 Carbohydrates ⓘ

There are no oligosaccharides in this entry.

Not For Manuscript Review

5.6 Ligand geometry (i)

There are no ligands in this entry.

5.7 Other polymers (i)

There are no such residues in this entry.

5.8 Polymer linkage issues (i)

There are no chain breaks in this entry.

Not For Manuscript Review

6 Map visualisation (i)

This section contains visualisations of the EMDB entry D_1292144858. These allow visual inspection of the internal detail of the map and identification of artifacts.

Images derived from a raw map, generated by summing the deposited half-maps, are presented below the corresponding image components of the primary map to allow further visual inspection and comparison with those of the primary map.

6.1 Orthogonal projections (i)

6.1.1 Primary map

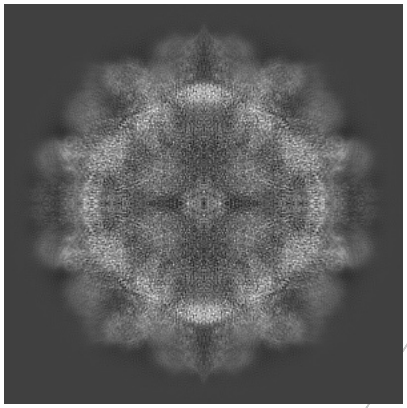

X

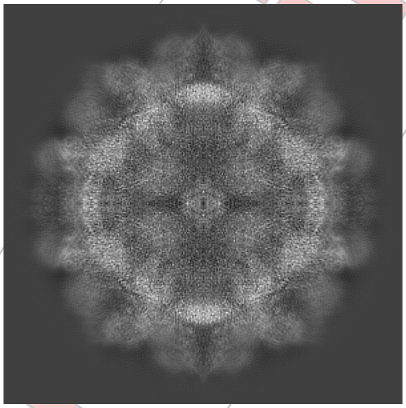

Y

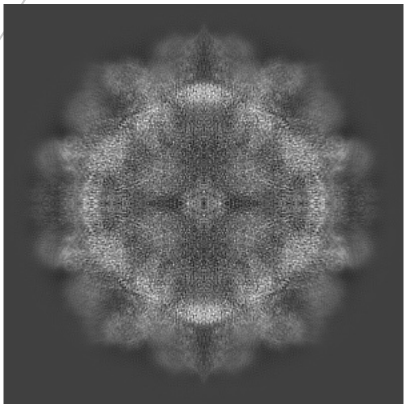

Z

6.1.2 Raw map

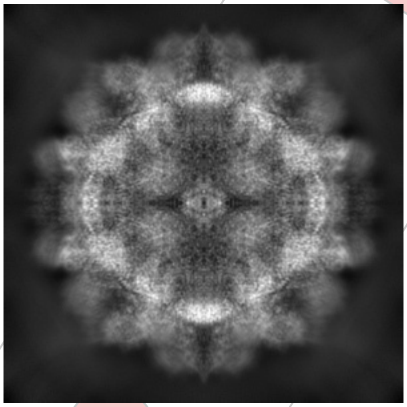

X

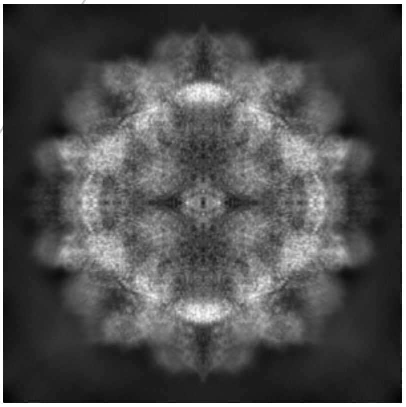

Y

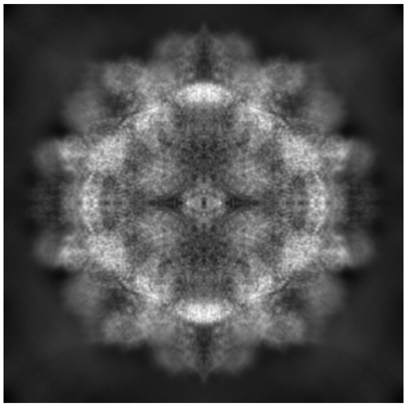

Z

The images above show the map projected in three orthogonal directions.

Not For Manuscript Review

6.2 Central slices (i)

6.2.1 Primary map

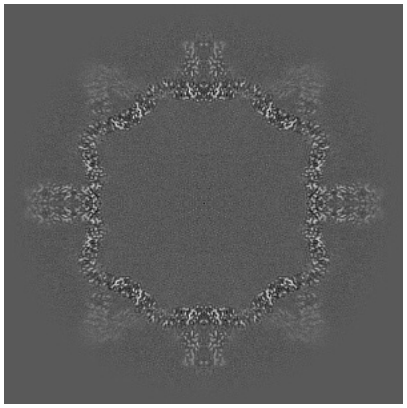
X Index: 220

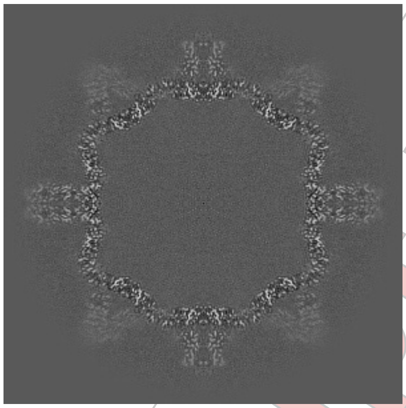
Y Index: 220

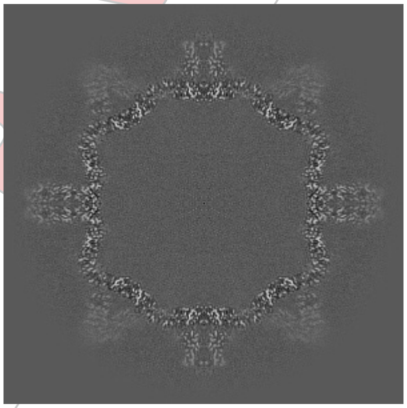
Z Index: 220

6.2.2 Raw map

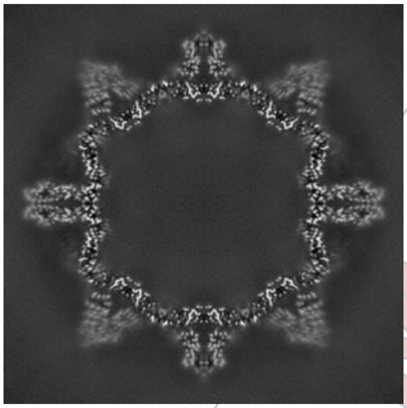
X Index: 220

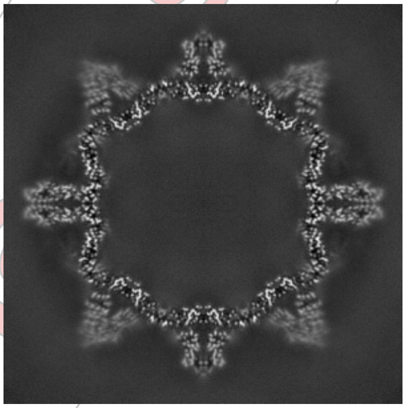
Y Index: 220

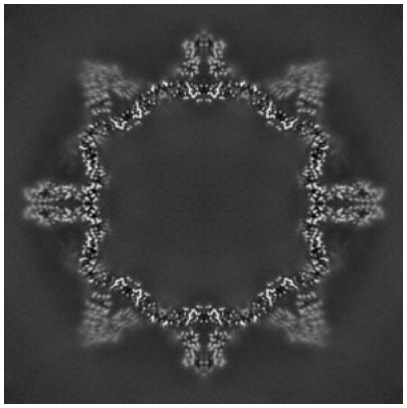
Z Index: 220

The images above show central slices of the map in three orthogonal directions.

Not For Manuscript Review

6.3 Largest variance slices (i)

6.3.1 Primary map

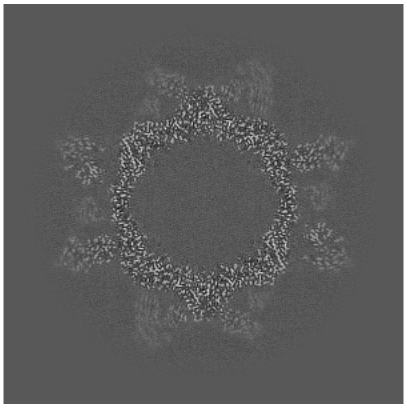
X Index: 133

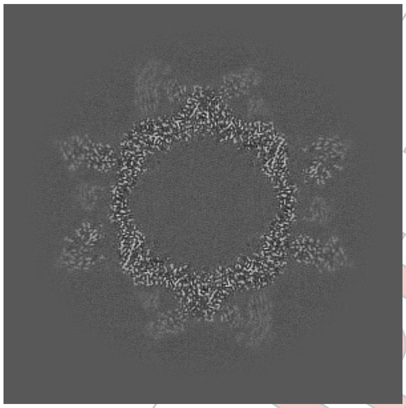
Y Index: 307

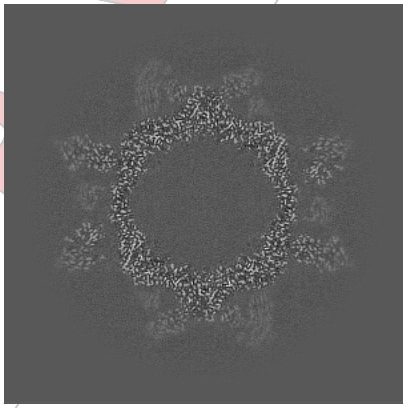
Z Index: 307

6.3.2 Raw map

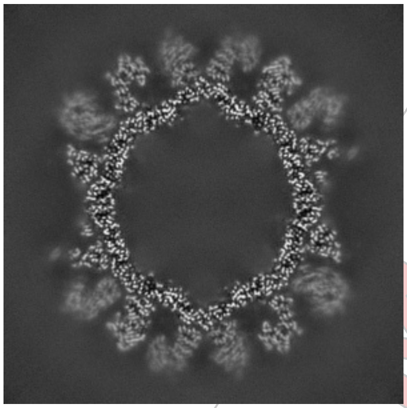
X Index: 283

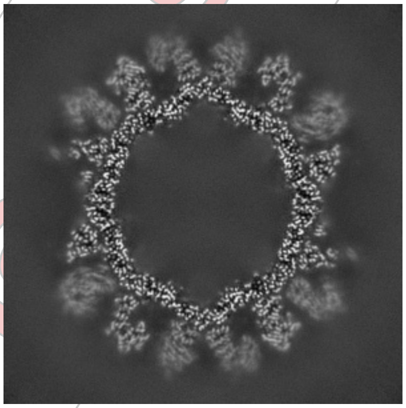
Y Index: 157

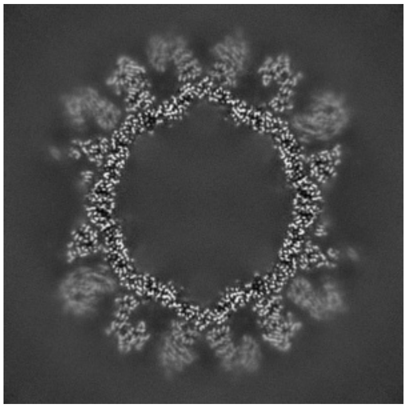
Z Index: 157

The images above show the largest variance slices of the map in three orthogonal directions.

Not For Manuscript Review

6.4 Orthogonal standard-deviation projections (False-color) (i)

6.4.1 Primary map

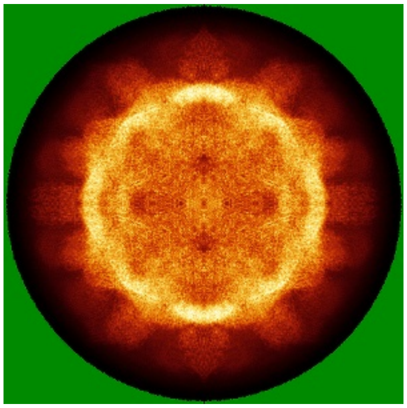
X

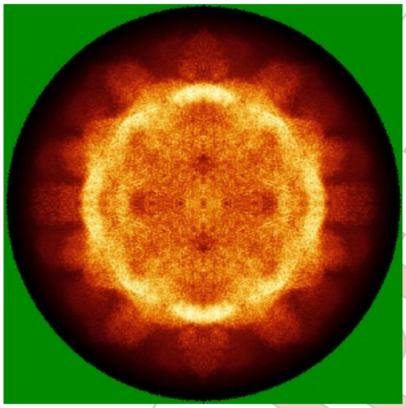
Y

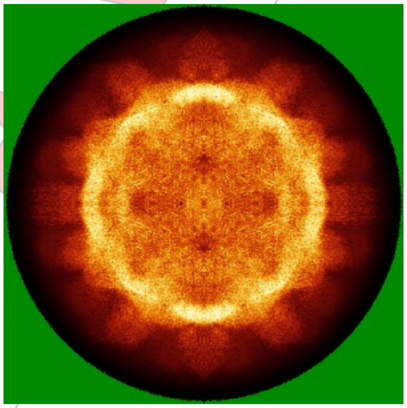
Z

6.4.2 Raw map

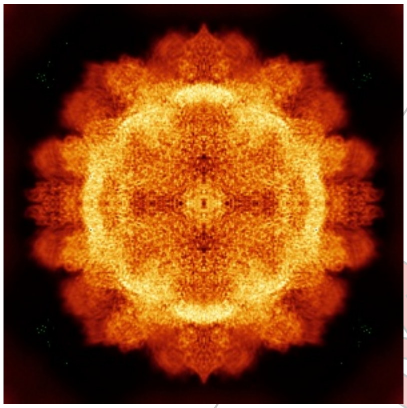
X

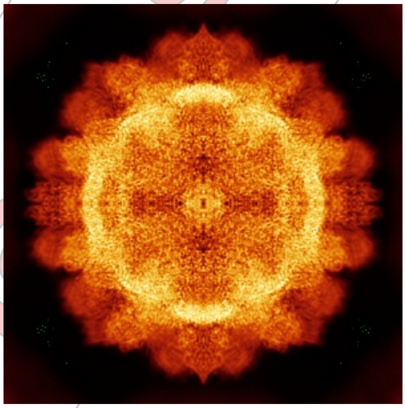
Y

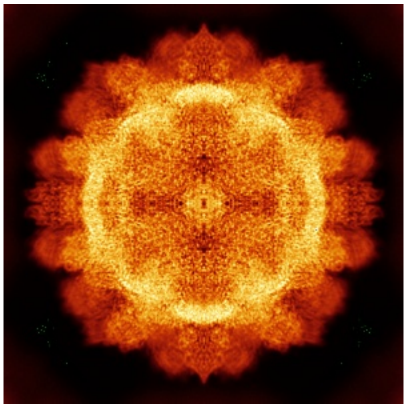
Z

The images above show the map standard deviation projections with false color in three orthogonal directions. Minimum values are shown in green, max in blue, and dark to light orange shades represent small to large values respectively.

Not For Manuscript Review

6.5 Orthogonal surface views (i)

6.5.1 Primary map

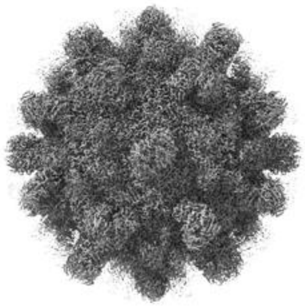
X

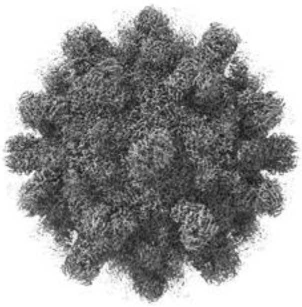
Y

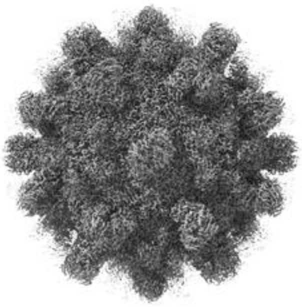
Z

The images above show the 3D surface view of the map at the recommended contour level 1.1. These images, in conjunction with the slice images, may facilitate assessment of whether an appropriate contour level has been provided.

6.5.2 Raw map

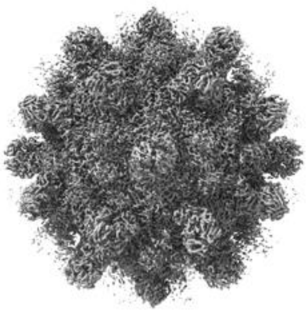
X

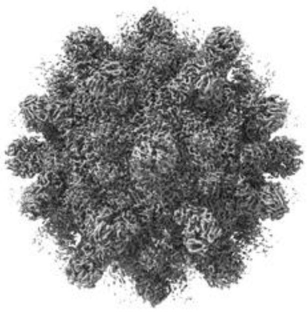
Y

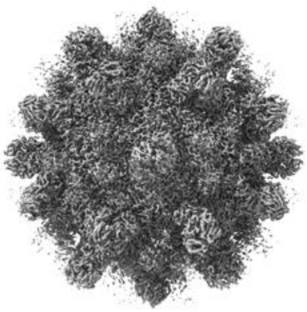
Z

These images show the 3D surface of the raw map. The raw map's contour level was selected so that its surface encloses the same volume as the primary map does at its recommended contour level.

6.6 Mask visualisation (i)

This section was not generated. No masks/segmentation were deposited.

Not For Manuscript Review

7 Map analysis (i)

This section contains the results of statistical analysis of the map.

7.1 Map-value distribution (i)

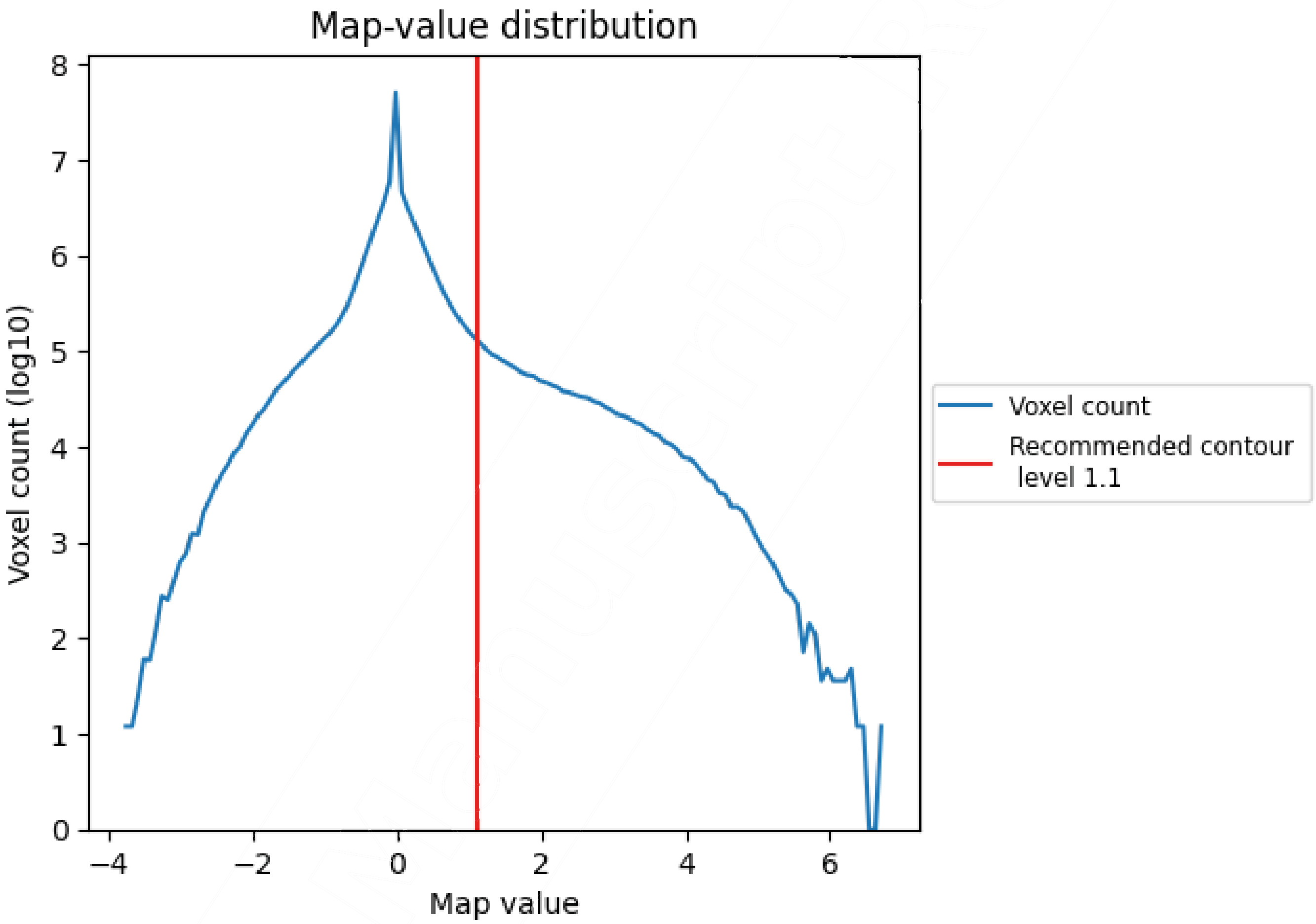

The map-value distribution is plotted in 128 intervals along the x-axis. The y-axis is logarithmic. A spike in this graph at zero usually indicates that the volume has been masked.

Not For Manuscript Review

7.2 Volume estimate (i)

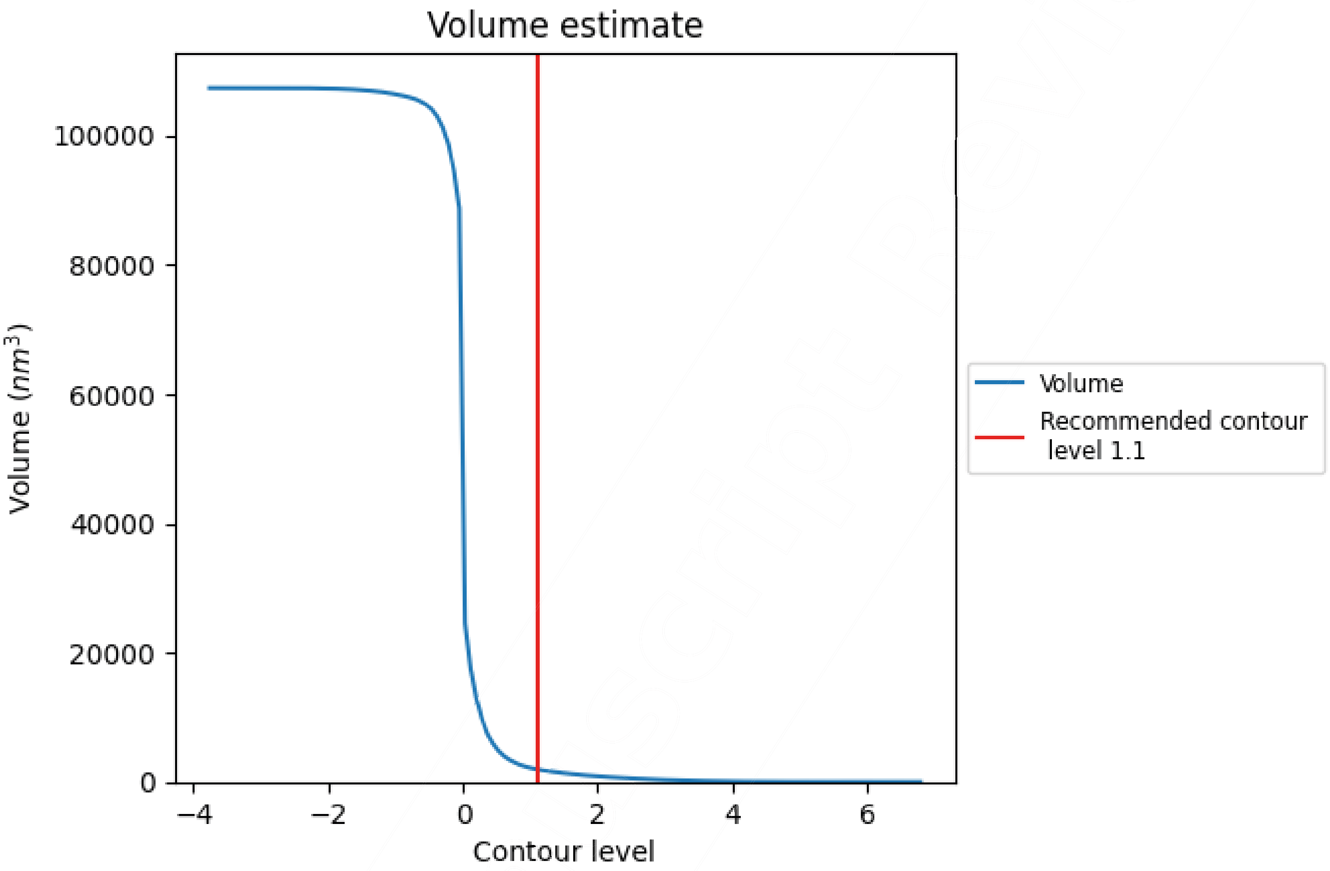

The volume at the recommended contour level is 1971 nm^3; this corresponds to an approximate mass of 1781 kDa.

The volume estimate graph shows how the enclosed volume varies with the contour level. The recommended contour level is shown as a vertical line and the intersection between the line and the curve gives the volume of the enclosed surface at the given level.

Not For Manuscript Review

7.3 Rotationally averaged power spectrum (i)

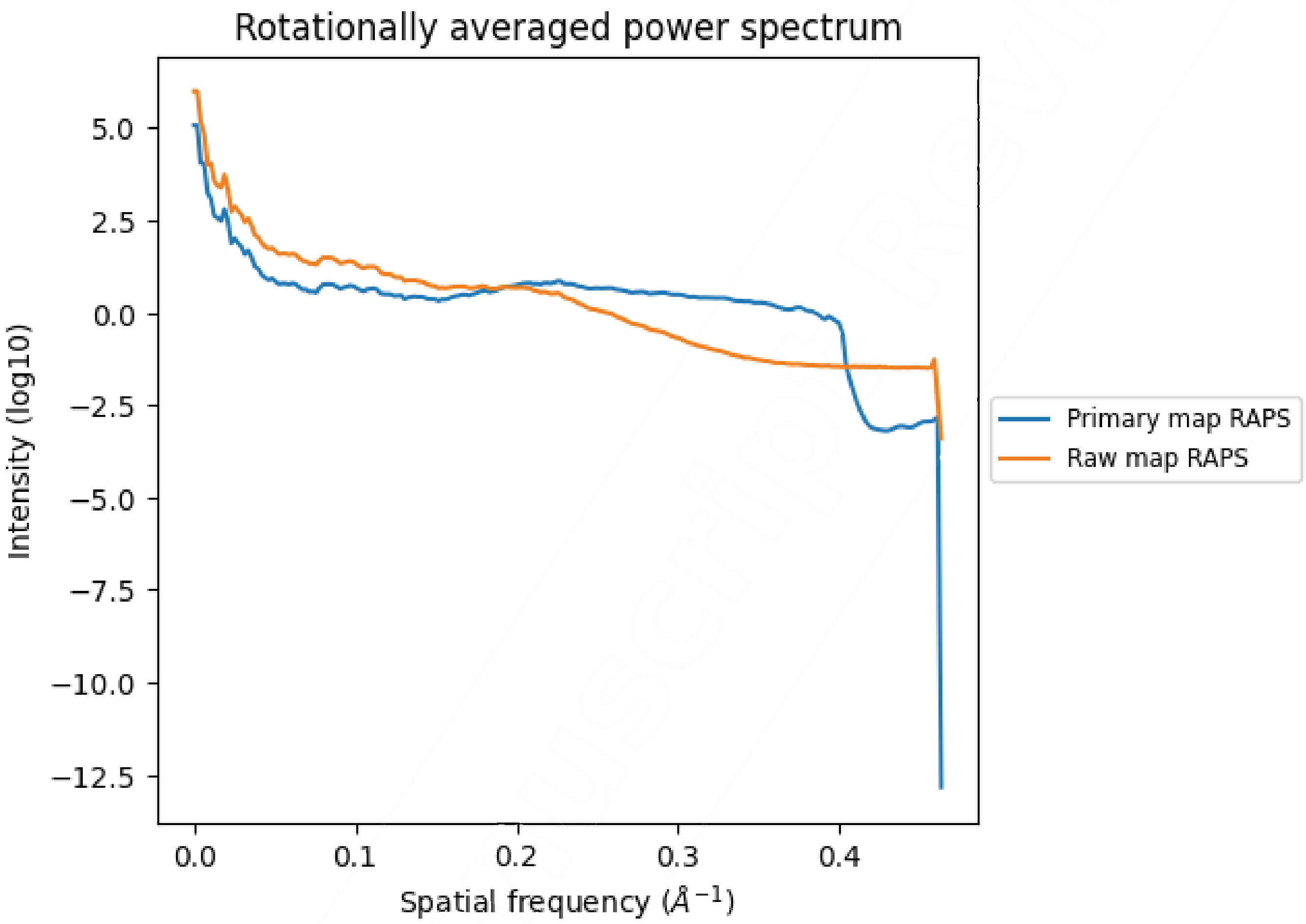

Not For Manuscript Review

8 Fourier-Shell correlation (i)

Fourier-Shell Correlation (FSC) is the most commonly used method to estimate the resolution of single-particle and subtomogram-averaged maps. The shape of the curve depends on the imposed symmetry, mask and whether or not the two 3D reconstructions used were processed from a common reference. The reported resolution is shown as a black line. A curve is displayed for the half-bit criterion in addition to lines showing the 0.143 gold standard cut-off and 0.5 cut-off.

8.1 FSC (i)

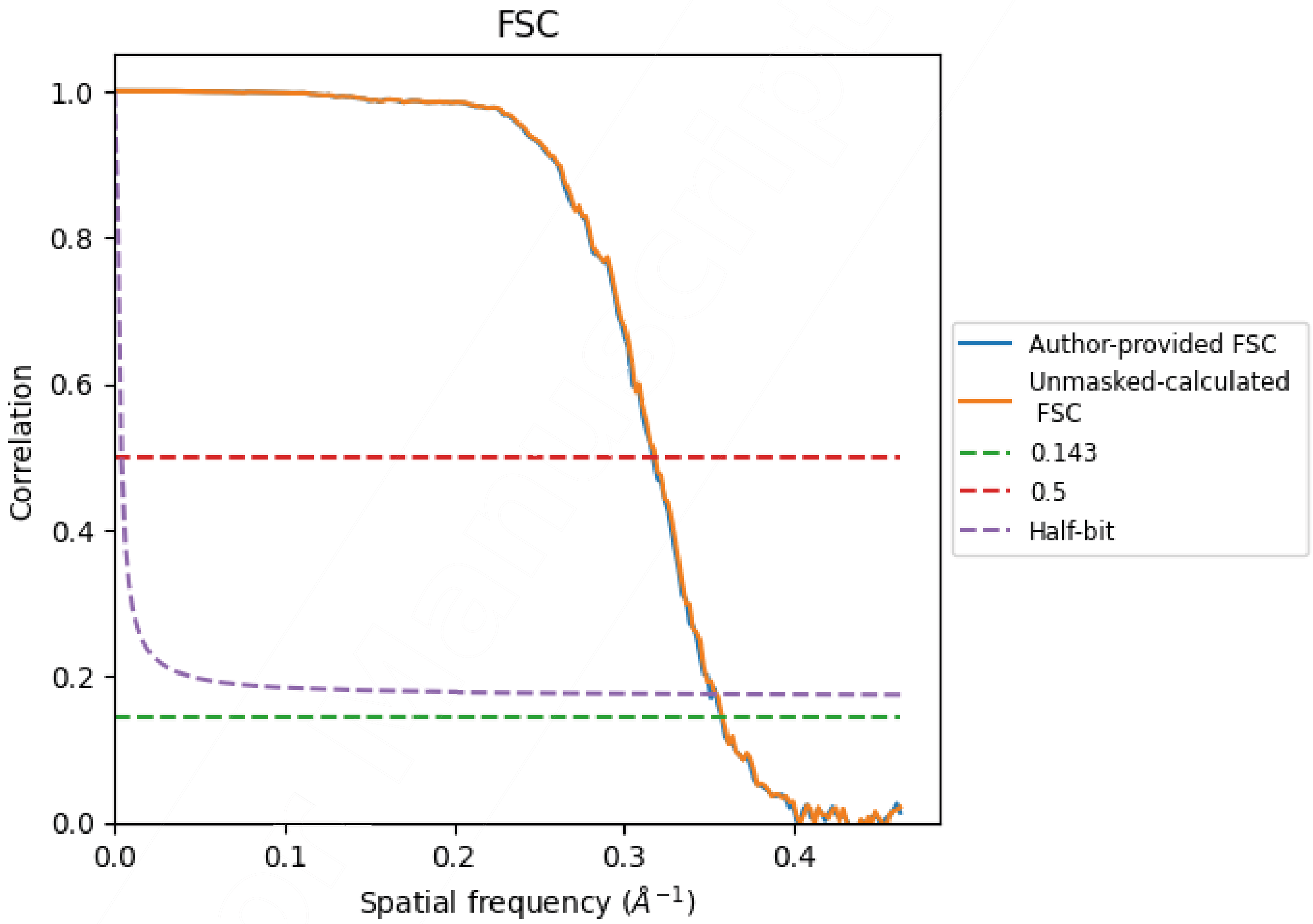

Not For Manuscript Review

8.2 Resolution estimates (i)

Resolution estimate (Å)	Estimation criterion (FSC cut-off)		
	0.143	0.5	Half-bit
Reported by author	-	-	-
Author-provided FSC curve	2.79	3.15	2.85
Unmasked-calculated*	2.79	3.14	2.82

*Resolution estimate based on FSC curve calculated by comparison of deposited half-maps.

Not For Manuscript Review

9 Map-model fit (i)

This section contains information regarding the fit between EMDB map D_1292144858 and PDB model D_1292144858. Per-residue inclusion information can be found in section 3 on page 4.

9.1 Map-model overlay (i)

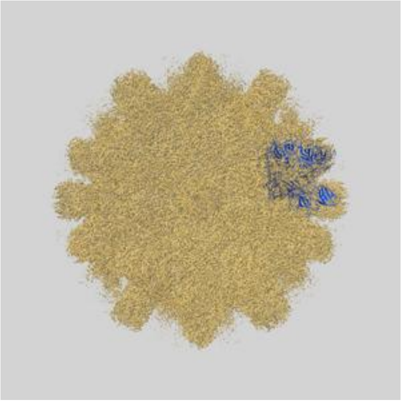

X

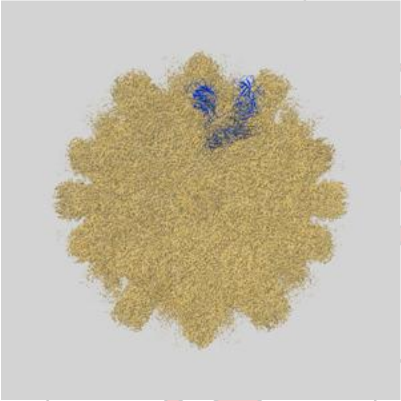

Y

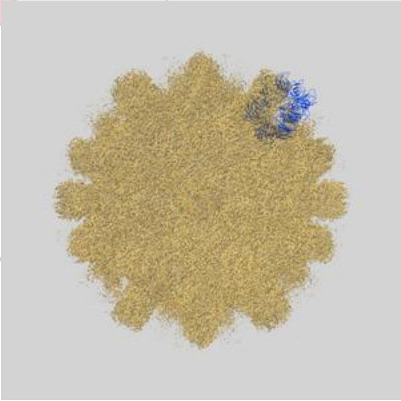

Z

The images above show the 3D surface view of the map at the recommended contour level 1.1 at 50% transparency in yellow overlaid with a ribbon representation of the model coloured in blue. These images allow for the visual assessment of the quality of fit between the atomic model and the map.

Not For Manuscript Review

9.2 Q-score mapped to coordinate model (i)

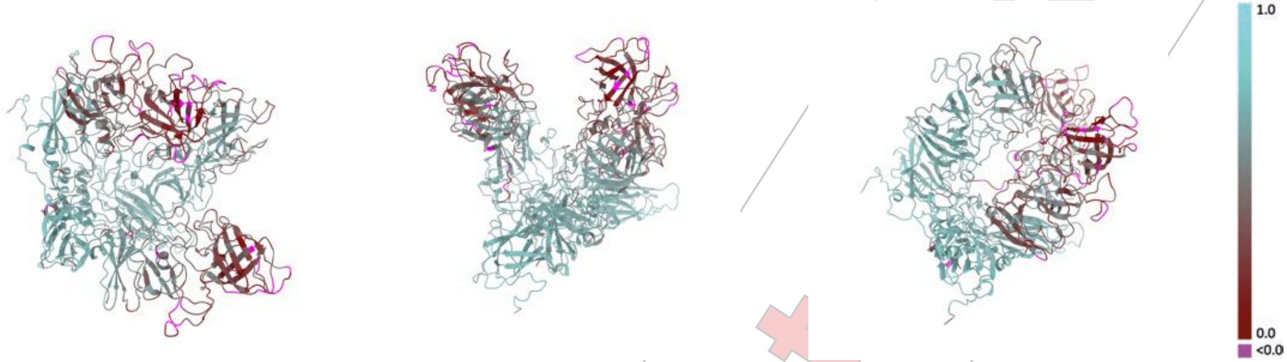

X Y Z

The images above show the model with each residue coloured according its Q-score. This shows their resolvability in the map with higher Q-score values reflecting better resolvability. Please note: Q-score is calculating the resolvability of atoms, and thus high values are only expected at resolutions at which atoms can be resolved. Low Q-score values may therefore be expected for many entries.

9.3 Atom inclusion mapped to coordinate model (i)

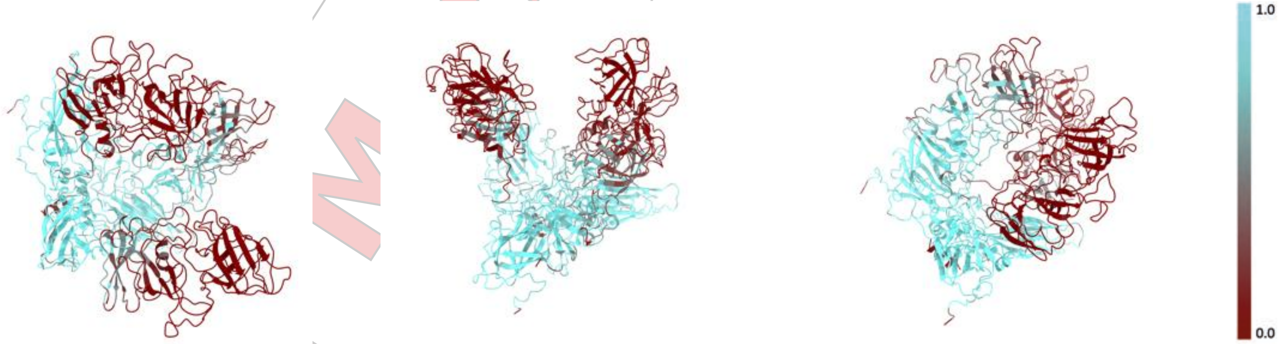

X Y Z

The images above show the model with each residue coloured according to its atom inclusion. This shows to what extent they are inside the map at the recommended contour level (1.1).

Not For Manuscript Review

9.4 Atom inclusion (i)

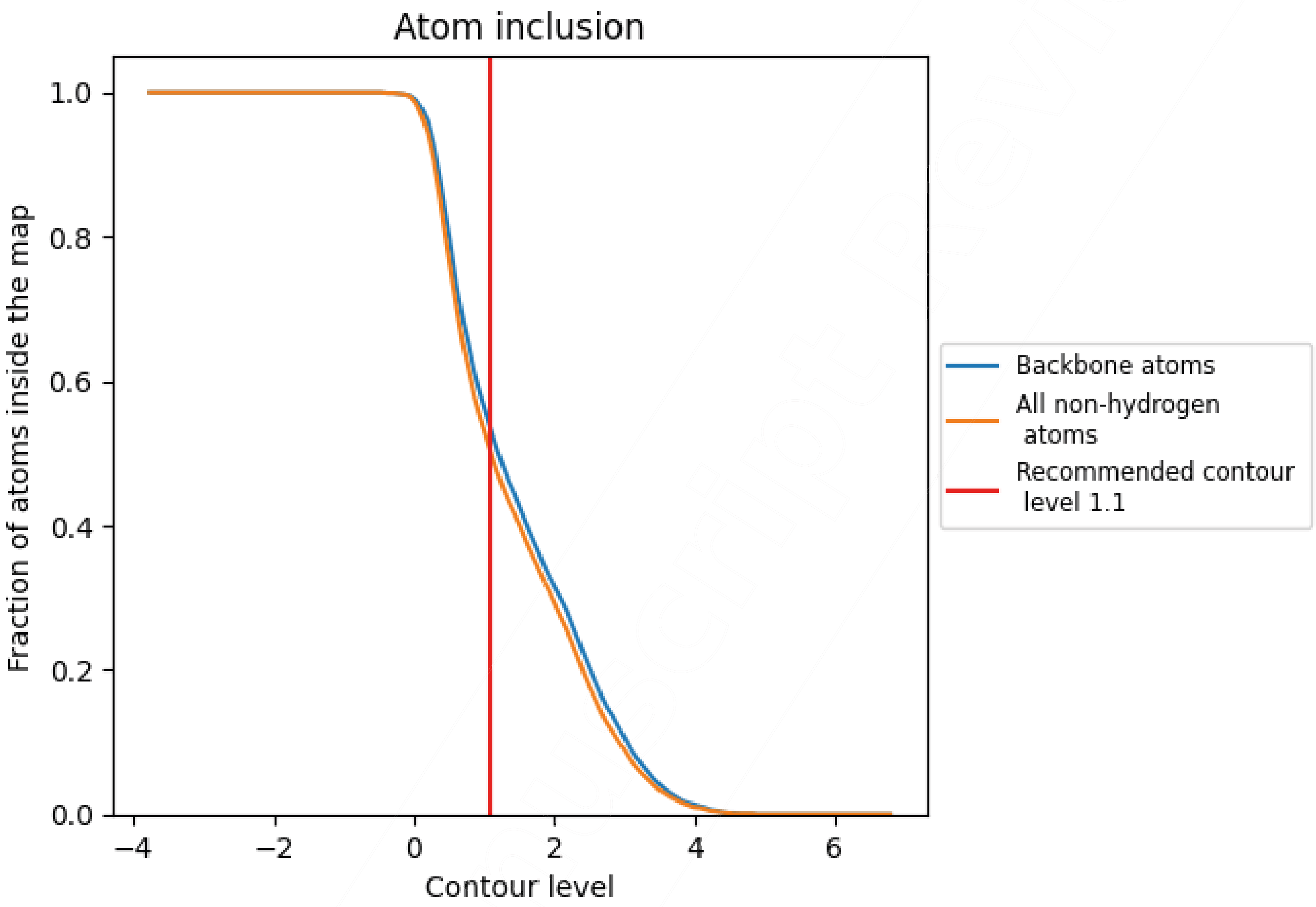

At the recommended contour level, 53% of all backbone atoms, 50% of all non-hydrogen atoms, are inside the map.

Not For Manuscript Review

9.5 Map-model fit summary (i)

The table lists the average atom inclusion at the recommended contour level (1.1) and Q-score for the entire model and for each chain.

Chain	Atom inclusion	Q-score
All	0.5000	0.5050
A	0.4100	0.4790
B	0.3730	0.4270
C	0.7240	0.6120
D	0.0430	0.3380

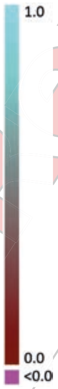

Not For Manuscript Review

Preliminary Full wwPDB EM Validation Report (i)

Feb 5, 2025 – 04:33 pm GMT

Deposition ID : D_1292144934

This wwPDB validation report is NOT for manuscript review

This is a Preliminary Full wwPDB EM Validation Report.

This report is produced by the wwPDB Deposition System during initial deposition but before annotation of the structure.

We welcome your comments at *validation@mail.wwpdb.org*
A user guide is available at
https://www.wwpdb.org/validation/2017/EMValidationReportHelp
with specific help available everywhere you see the (i) symbol.

The types of validation reports are described at
http://www.wwpdb.org/validation/2017/FAQs#types.

Not For Manuscript Review

The following versions of software and data (see references (i)) were used in the production of this report:

EMDB validation analysis	:	0.0.1.dev113
MolProbity	:	4.02b-467
Percentile statistics	:	20231227.v01 (using entries in the PDB archive December 27th 2023)
MapQ	:	1.9.13
Ideal geometry (proteins)	:	Engh & Huber (2001)
Ideal geometry (DNA, RNA)	:	Parkinson et al. (1996)
Validation Pipeline (wwPDB-VP)	:	2.40

1 Overall quality at a glance (i)

The following experimental techniques were used to determine the structure:
ELECTRON MICROSCOPY

The reported resolution of this entry is 3.30 Å.

Percentile scores (ranging between 0-100) for global validation metrics of the entry are shown in the following graphic. The table shows the number of entries on which the scores are based.

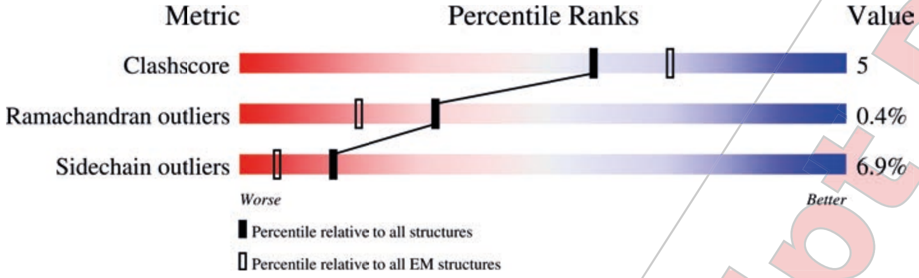

Metric	Whole archive (#Entries)	EM structures (#Entries)
Clashscore	210492	15764
Ramachandran outliers	207382	16835
Sidechain outliers	206894	16415

The table below summarises the geometric issues observed across the polymeric chains and their fit to the map. The red, orange, yellow and green segments of the bar indicate the fraction of residues that contain outliers for >=3, 2, 1 and 0 types of geometric quality criteria respectively. A grey segment represents the fraction of residues that are not modelled. The numeric value for each fraction is indicated below the corresponding segment, with a dot representing fractions <=5% The upper red bar (where present) indicates the fraction of residues that have poor fit to the EM map (all-atom inclusion < 40%). The numeric value is given above the bar.

Mol	Chain	Length	Quality of chain
1	A	534	45%<br>86% 13% •
2	B	550	55%<br>85% 13% •
3	C	550	8%<br>87% 12% •

Not For Manuscript Review

2 Entry composition (i)

There are 3 unique types of molecules in this entry. The entry contains 12059 atoms, of which 0 are hydrogens and 0 are deuteriums.

In the tables below, the AltConf column contains the number of residues with at least one atom in alternate conformation and the Trace column contains the number of residues modelled with at most 2 atoms.

- Molecule 1 is a protein called Capsid protein (subunit A).

Mol	Chain	Residues	Atoms					AltConf	Trace
			Total	C	N	O	S		
1	A	534	3949	2508	669	761	11	0	0

- Molecule 2 is a protein called Capsid protein (subunit B).

Mol	Chain	Residues	Atoms					AltConf	Trace
			Total	C	N	O	S		
2	B	550	4054	2571	685	786	12	0	0

- Molecule 3 is a protein called Capsid protein (subunit C).

Mol	Chain	Residues	Atoms					AltConf	Trace
			Total	C	N	O	S		
3	C	550	4056	2572	685	787	12	0	0

Not For Manuscript Review

3 Residue-property plots (i)

These plots are drawn for all protein, RNA, DNA and oligosaccharide chains in the entry. The first graphic for a chain summarises the proportions of the various outlier classes displayed in the second graphic. The second graphic shows the sequence view annotated by issues in geometry and atom inclusion in map density. Residues are color-coded according to the number of geometric quality criteria for which they contain at least one outlier: green = 0, yellow = 1, orange = 2 and red = 3 or more. A red diamond above a residue indicates a poor fit to the EM map for this residue (all-atom inclusion < 40%). Stretches of 2 or more consecutive residues without any outlier are shown as a green connector. Residues present in the sample, but not in the model, are shown in grey.

- Molecule 1: Capsid protein (subunit A)

Chain A:

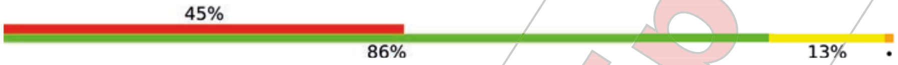

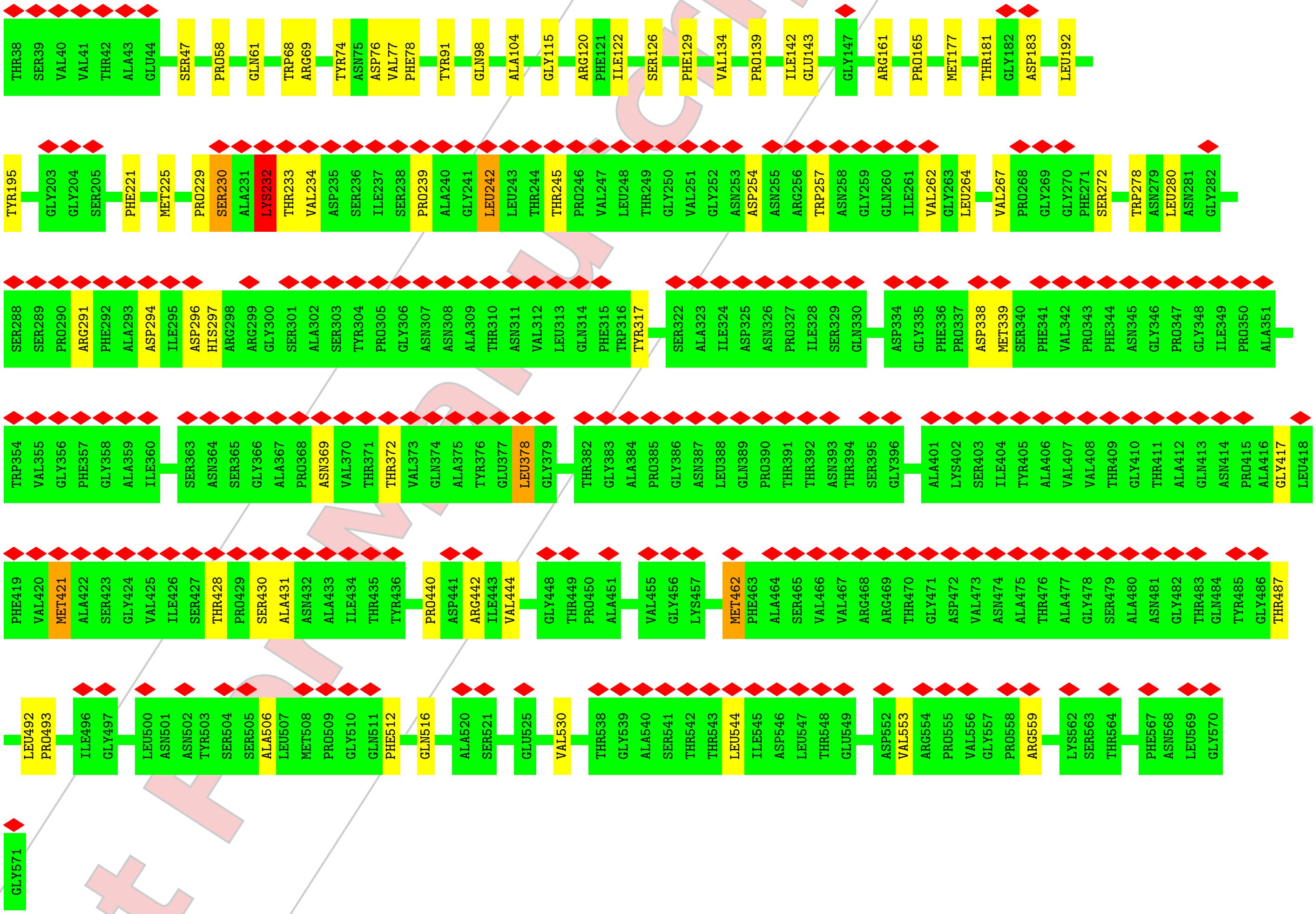

- Molecule 2: Capsid protein (subunit B)

Chain B:

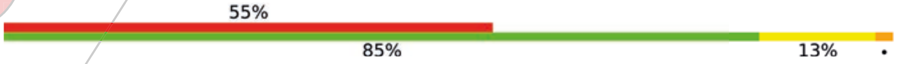

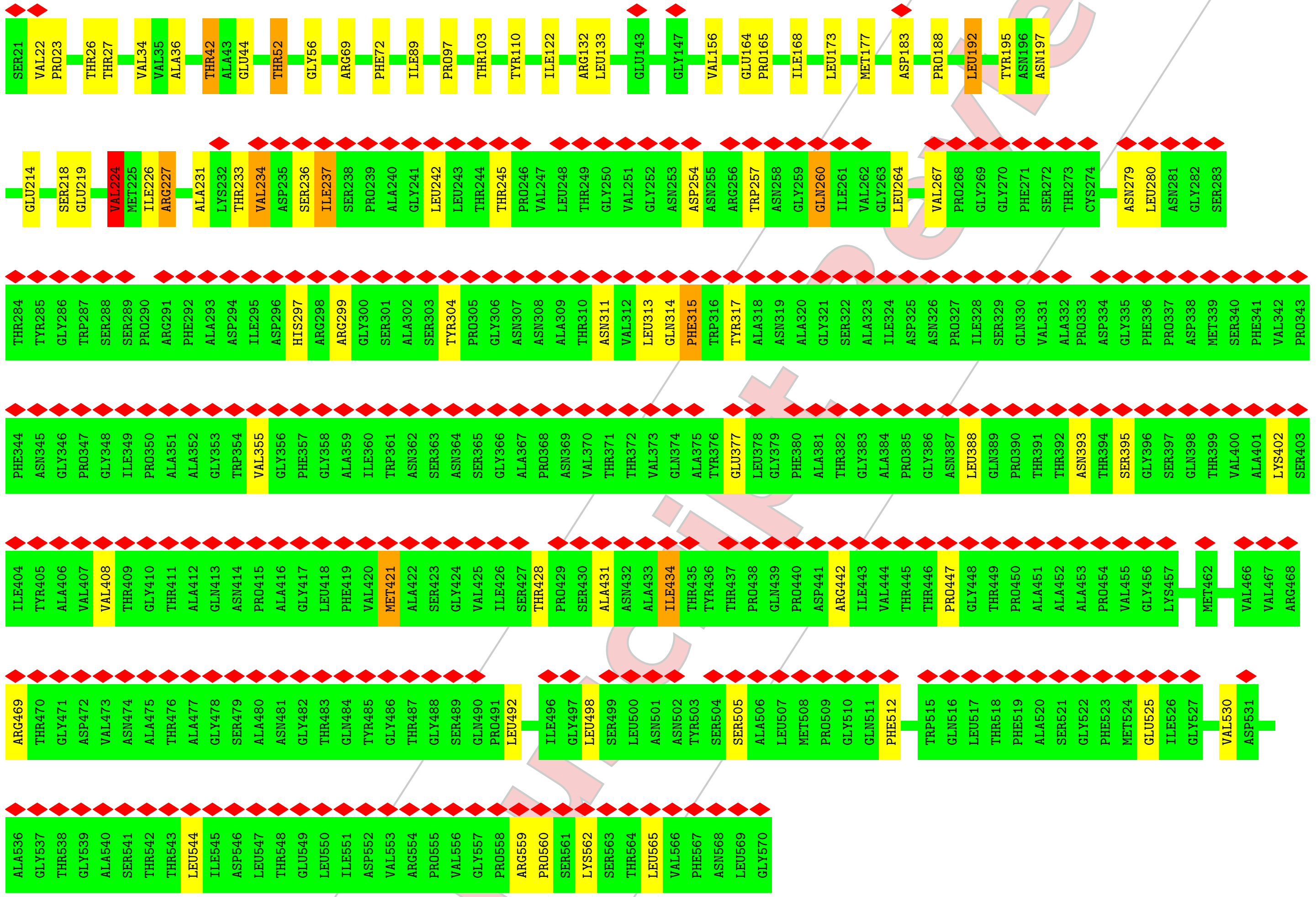

• Molecule 3: Capsid protein (subunit C)

Chain C:

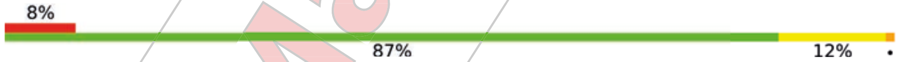

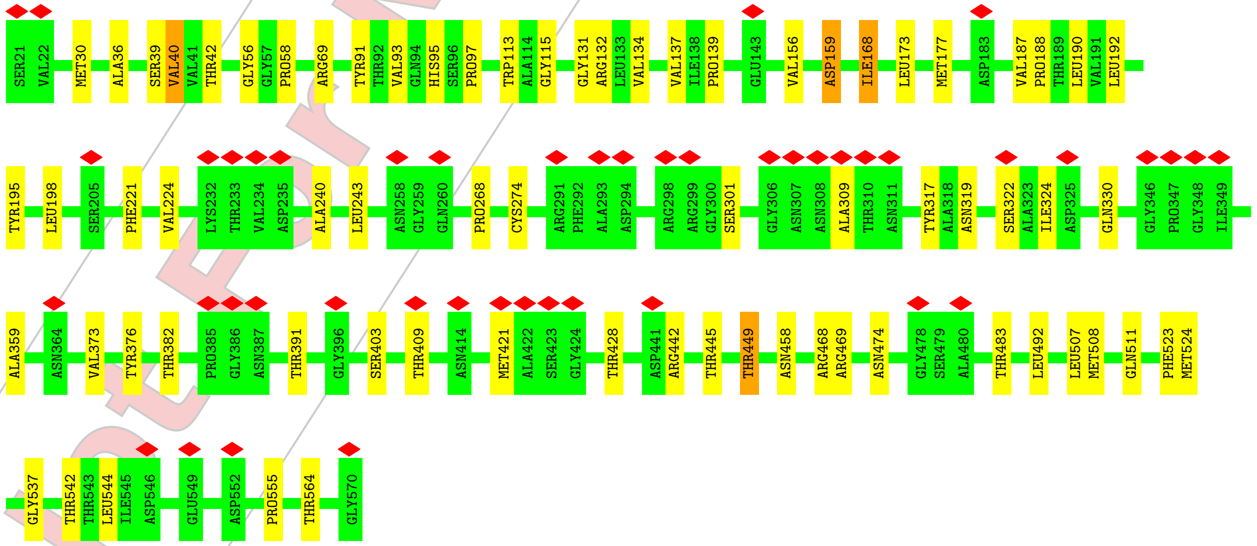

Not For Manuscript Review

4 Experimental information (i)

Property	Value	Source
EM reconstruction method	SINGLE PARTICLE	Depositor
Imposed symmetry	POINT, Not provided	
Number of particles used	43950	Depositor
Resolution determination method	FSC 0.143 CUT-OFF	Depositor
CTF correction method	PHASE FLIPPING AND AMPLITUDE CORRECTION	Depositor
Microscope	TFS KRIOS	Depositor
Voltage (kV)	300	Depositor
Electron dose ($e^-/\text{Å}^2$)	53	Depositor
Minimum defocus (nm)	800	Depositor
Maximum defocus (nm)	2300	Depositor
Magnification	Not provided	
Image detector	FEI FALCON III (4k x 4k)	Depositor
Maximum map value	2.207	Depositor
Minimum map value	-1.136	Depositor
Average map value	0.007	Depositor
Map value standard deviation	0.177	Depositor
Recommended contour level	0.47	Depositor
Map size (Å)	466.39996, 466.39996, 466.39996	wwPDB
Map dimensions	440, 440, 440	wwPDB
Map angles (°)	90.0, 90.0, 90.0	wwPDB
Pixel spacing (Å)	1.06, 1.06, 1.06	Depositor

Not For Manuscript Review

5 Model quality (i)

5.1 Standard geometry (i)

The Z score for a bond length (or angle) is the number of standard deviations the observed value is removed from the expected value. A bond length (or angle) with $|Z| > 5$ is considered an outlier worth inspection. RMSZ is the root-mean-square of all Z scores of the bond lengths (or angles).

Mol	Chain	Bond lengths		Bond angles	
		RMSZ	#$\|Z\|$ >5	RMSZ	#$\|Z\|$ >5
1	A	0.41	0/4062	0.65	3/5581 (0.1%)
2	B	0.42	0/4169	0.63	2/5731 (0.0%)
3	C	0.39	0/4171	0.60	0/5733
All	All	0.41	0/12402	0.63	5/17045 (0.0%)

Chiral center outliers are detected by calculating the chiral volume of a chiral center and verifying if the center is modelled as a planar moiety or with the opposite hand.A planarity outlier is detected by checking planarity of atoms in a peptide group, atoms in a mainchain group or atoms of a sidechain that are expected to be planar.

Mol	Chain	#Chirality outliers	#Planarity outliers
1	A	0	1

There are no bond length outliers.

All (5) bond angle outliers are listed below:

Mol	Chain	Res	Type	Atoms	Z	Observed(^{o})	Ideal(^{o})
1	A	296	ASP	CB-CG-OD1	6.50	124.15	118.30
2	B	498	LEU	CA-CB-CG	5.98	129.05	115.30
2	B	544	LEU	CA-CB-CG	5.62	128.22	115.30
1	A	378	LEU	CA-CB-CG	5.51	127.97	115.30
1	A	242	LEU	CA-CB-CG	5.47	127.88	115.30

There are no chirality outliers.

All (1) planarity outliers are listed below:

Mol	Chain	Res	Type	Group
1	A	69	ARG	Sidechain

Not For Manuscript Review

5.2 Too-close contacts (i)

In the following table, the Non-H and H(model) columns list the number of non-hydrogen atoms and hydrogen atoms in the chain respectively. The H(added) column lists the number of hydrogen atoms added and optimized by MolProbity. The Clashes column lists the number of clashes within the asymmetric unit, whereas Symm-Clashes lists symmetry-related clashes.

Mol	Chain	Non-H	H(model)	H(added)	Clashes	Symm-Clashes
1	A	3949	0	3818	36	0
2	B	4054	0	3915	48	0
3	C	4056	0	3918	43	0
All	All	12059	0	11651	107	0

The all-atom clashscore is defined as the number of clashes found per 1000 atoms (including hydrogen atoms). The all-atom clashscore for this structure is 5.

All (107) close contacts within the same asymmetric unit are listed below, sorted by their clash magnitude.

Atom-1	Atom-2	Interatomic distance (Å)	Clash overlap (Å)
2:B:393:ASN:CG	3:C:324:ILE:HG21	1.12	1.49
2:B:393:ASN:OD1	3:C:324:ILE:CG1	1.74	1.35
2:B:393:ASN:OD1	3:C:324:ILE:CG2	1.76	1.33
2:B:393:ASN:OD1	3:C:324:ILE:HG12	1.13	1.24
2:B:393:ASN:CG	3:C:324:ILE:CG2	2.06	1.22
2:B:393:ASN:OD1	3:C:324:ILE:HG21	1.31	1.18
2:B:393:ASN:ND2	3:C:324:ILE:HG21	1.66	1.11
1:A:77:VAL:O	1:A:559:ARG:NH2	1.83	1.09
2:B:393:ASN:OD1	3:C:324:ILE:CB	2.00	1.09
2:B:227:ARG:HH21	2:B:231:ALA:H	1.12	0.95
1:A:77:VAL:C	1:A:559:ARG:HH21	1.72	0.92
1:A:143:GLU:OE2	2:B:233:THR:HA	1.79	0.82
2:B:227:ARG:NH2	2:B:231:ALA:H	1.79	0.80
2:B:395:SER:HB3	3:C:324:ILE:HG12	1.69	0.74
2:B:393:ASN:ND2	3:C:324:ILE:CG2	2.44	0.73
2:B:395:SER:HB3	3:C:324:ILE:CG1	2.22	0.70
1:A:78:PHE:HA	1:A:559:ARG:NH2	2.08	0.68
1:A:120:ARG:HG2	1:A:165:PRO:HB2	1.78	0.66
1:A:78:PHE:CA	1:A:559:ARG:NH2	2.61	0.63
1:A:98:GLN:HA	1:A:104:ALA:HB2	1.83	0.61
2:B:447:PRO:HG3	3:C:324:ILE:HG23	1.82	0.61
3:C:36:ALA:HB1	3:C:156:VAL:HG21	1.84	0.60
1:A:280:LEU:HD22	1:A:530:VAL:HA	1.83	0.60
2:B:122:ILE:HG12	2:B:165:PRO:HB3	1.84	0.59

Continued on next page...

Not For Manuscript Review

Continued from previous page...

Atom-1	Atom-2	Interatomic distance (Å)	Clash overlap (Å)
2:B:227:ARG:HH21	2:B:231:ALA:N	1.94	0.59
3:C:309:ALA:HB1	3:C:409:THR:HA	1.85	0.59
1:A:77:VAL:C	1:A:559:ARG:NH2	2.47	0.58
1:A:78:PHE:CA	1:A:559:ARG:HH21	2.16	0.57
2:B:355:VAL:HG12	2:B:377:GLU:HB2	1.86	0.57
1:A:78:PHE:HA	1:A:559:ARG:HH21	1.66	0.57
3:C:36:ALA:HB3	3:C:168:ILE:HG22	1.87	0.57
2:B:428:THR:HG21	2:B:434:ILE:HD13	1.88	0.56
1:A:91:TYR:HB3	1:A:192:LEU:HB2	1.87	0.55
3:C:91:TYR:HB3	3:C:192:LEU:HB2	1.90	0.54
1:A:267:VAL:HG21	1:A:440:PRO:HG3	1.90	0.54
3:C:134:VAL:HB	3:C:195:TYR:HB2	1.88	0.54
1:A:338:ASP:HB2	1:A:444:VAL:HG22	1.90	0.53
3:C:274:CYS:HB2	3:C:449:THR:HG21	1.91	0.53
1:A:239:PRO:HD3	1:A:506:ALA:HB1	1.89	0.53
2:B:311:ASN:H	2:B:408:VAL:HA	1.74	0.53
2:B:395:SER:O	3:C:324:ILE:HD11	2.09	0.52
1:A:115:GLY:HA3	1:A:221:PHE:HA	1.90	0.52
1:A:122:ILE:HG12	1:A:165:PRO:HB3	1.92	0.52
2:B:236:SER:O	2:B:237:ILE:C	2.48	0.52
3:C:56:GLY:HA3	3:C:224:VAL:HB	1.91	0.52
2:B:234:VAL:HG12	2:B:505:SER:OG	2.10	0.51
1:A:297:HIS:O	1:A:417:GLY:HA3	2.11	0.50
1:A:225:MET:HE2	3:C:139:PRO:HB3	1.94	0.50
1:A:245:THR:HG22	1:A:553:VAL:H	1.77	0.49
1:A:233:THR:HG22	1:A:234:VAL:H	1.77	0.49
1:A:74:TYR:HE1	1:A:77:VAL:HG23	1.77	0.49
3:C:376:TYR:HA	3:C:403:SER:HA	1.93	0.49
1:A:78:PHE:HA	1:A:559:ARG:CZ	2.43	0.49
1:A:78:PHE:N	1:A:559:ARG:HH21	2.11	0.49
2:B:233:THR:HG22	2:B:234:VAL:N	2.28	0.49
1:A:229:PRO:O	1:A:230:SER:C	2.51	0.48
1:A:126:SER:HB3	1:A:129:PHE:HD1	1.78	0.48
3:C:268:PRO:HG2	3:C:542:THR:HG22	1.96	0.48
2:B:133:LEU:HD13	2:B:192:LEU:HD21	1.94	0.48
2:B:89:ILE:CG2	2:B:560:PRO:HG2	2.43	0.47
2:B:89:ILE:O	2:B:562:LYS:CD	2.54	0.47
2:B:280:LEU:HD13	2:B:530:VAL:HA	1.96	0.47
2:B:260:GLN:H	2:B:260:GLN:HG2	1.60	0.46
3:C:458:ASN:HA	3:C:537:GLY:HA2	1.97	0.46
1:A:462:MET:HG2	1:A:487:THR:HB	1.97	0.45

Continued on next page...

Not For Manuscript Review

Continued from previous page...

Atom-1	Atom-2	Interatomic distance (Å)	Clash overlap (Å)
3:C:40:VAL:HG11	3:C:137:VAL:HB	1.98	0.45
2:B:56:GLY:HA3	2:B:224:VAL:HG11	1.99	0.45
3:C:507:LEU:HD22	3:C:511:GLN:HB3	1.97	0.45
1:A:232:LYS:HA	1:A:232:LYS:HD2	1.41	0.45
3:C:382:THR:HG23	3:C:391:THR:HB	1.98	0.45
2:B:89:ILE:HG22	2:B:560:PRO:HG2	1.98	0.45
2:B:431:ALA:HA	2:B:434:ILE:HD11	1.97	0.45
2:B:173:LEU:HD12	2:B:188:PRO:HB3	1.98	0.44
1:A:58:PRO:HD3	3:C:42:THR:HG21	1.99	0.44
2:B:52:THR:HG23	2:B:177:MET:HG2	1.98	0.44
3:C:95:HIS:HB3	3:C:187:VAL:HB	2.00	0.44
3:C:115:GLY:HA3	3:C:221:PHE:HA	2.00	0.44
1:A:61:GLN:HB3	1:A:68:TRP:HZ2	1.82	0.44
2:B:89:ILE:O	2:B:562:LYS:HD2	2.17	0.44
2:B:395:SER:OG	3:C:322:SER:HA	2.18	0.44
2:B:421:MET:N	2:B:421:MET:SD	2.91	0.44
2:B:72:PHE:HB3	2:B:214:GLU:HB3	1.99	0.43
1:A:78:PHE:HB3	1:A:559:ARG:NH2	2.33	0.43
1:A:421:MET:SD	1:A:421:MET:N	2.90	0.43
1:A:492:LEU:N	1:A:493:PRO:HD2	2.33	0.43
2:B:36:ALA:HB1	2:B:156:VAL:HG11	2.00	0.43
2:B:315:PHE:HZ	2:B:388:LEU:HD11	1.82	0.43
3:C:359:ALA:HB2	3:C:373:VAL:HG22	2.00	0.43
2:B:97:PRO:HB3	2:B:103:THR:HG22	1.99	0.43
1:A:139:PRO:HD2	1:A:142:ILE:HD12	2.01	0.43
2:B:42:THR:HG21	3:C:58:PRO:HD3	2.01	0.43
2:B:559:ARG:HD3	2:B:559:ARG:HA	1.81	0.42
3:C:132:ARG:HG2	3:C:159:ASP:HA	2.01	0.42
1:A:181:THR:HG22	1:A:233:THR:HA	2.00	0.42
1:A:177:MET:HE2	3:C:177:MET:HA	2.00	0.42
3:C:97:PRO:HD3	3:C:113:TRP:CE2	2.55	0.42
3:C:173:LEU:HD12	3:C:188:PRO:HB3	2.03	0.41
2:B:525:GLU:HB2	2:B:565:LEU:HB3	2.02	0.41
2:B:447:PRO:HB2	3:C:330:GLN:HG3	1.90	0.41
2:B:22:VAL:HA	2:B:23:PRO:HD3	1.92	0.41
3:C:428:THR:HG21	3:C:469:ARG:HD2	2.03	0.41
3:C:508:MET:H	3:C:508:MET:HG3	1.71	0.41
3:C:131:GLY:HA3	3:C:198:LEU:HA	2.02	0.41
3:C:93:VAL:HG12	3:C:190:LEU:HB3	2.04	0.40
3:C:240:ALA:HB1	3:C:555:PRO:HG3	2.04	0.40
2:B:110:TYR:HA	2:B:226:ILE:O	2.21	0.40

Continued on next page...

Not For Manuscript Review

Continued from previous page...

Atom-1	Atom-2	Interatomic distance (Å)	Clash overlap (Å)
2:B:132:ARG:HB2	2:B:195:TYR:HB3	2.03	0.40

There are no symmetry-related clashes.

5.3 Torsion angles (i)

5.3.1 Protein backbone (i)

In the following table, the Percentiles column shows the percent Ramachandran outliers of the chain as a percentile score with respect to all PDB entries followed by that with respect to all EM entries.

The Analysed column shows the number of residues for which the backbone conformation was analysed, and the total number of residues.

Mol	Chain	Analysed	Favoured	Allowed	Outliers	Percentiles	
1	A	532/534 (100%)	479 (90%)	50 (9%)	3 (1%)	22	53
2	B	548/550 (100%)	504 (92%)	41 (8%)	3 (0%)	25	56
3	C	548/550 (100%)	514 (94%)	34 (6%)	0	100	100
All	All	1628/1634 (100%)	1497 (92%)	125 (8%)	6 (0%)	32	61

All (6) Ramachandran outliers are listed below:

Mol	Chain	Res	Type
1	A	232	LYS
1	A	431	ALA
2	B	434	ILE
2	B	237	ILE
1	A	230	SER
2	B	224	VAL

5.3.2 Protein sidechains (i)

In the following table, the Percentiles column shows the percent sidechain outliers of the chain as a percentile score with respect to all PDB entries followed by that with respect to all EM entries.

The Analysed column shows the number of residues for which the sidechain conformation was analysed, and the total number of residues.

Not For Manuscript Review

Mol	Chain	Analysed	Rotameric	Outliers	Percentiles	
1	A	424/424 (100%)	395 (93%)	29 (7%)	13	38
2	B	437/437 (100%)	399 (91%)	38 (9%)	8	29
3	C	438/438 (100%)	416 (95%)	22 (5%)	20	48
All	All	1299/1299 (100%)	1210 (93%)	89 (7%)	15	38

All (89) residues with a non-rotameric sidechain are listed below:

Mol	Chain	Res	Type
1	A	47	SER
1	A	76	ASP
1	A	134	VAL
1	A	161	ARG
1	A	183	ASP
1	A	195	TYR
1	A	232	LYS
1	A	242	LEU
1	A	254	ASP
1	A	257	TRP
1	A	262	VAL
1	A	264	LEU
1	A	272	SER
1	A	278	TRP
1	A	291	ARG
1	A	294	ASP
1	A	317	TYR
1	A	339	MET
1	A	369	ASN
1	A	372	THR
1	A	378	LEU
1	A	421	MET
1	A	428	THR
1	A	430	SER
1	A	442	ARG
1	A	462	MET
1	A	512	PHE
1	A	516	GLN
1	A	544	LEU
2	B	26	THR
2	B	27	THR
2	B	34	VAL
2	B	42	THR
2	B	44	GLU

Continued on next page...

Not For Manuscript Review

Continued from previous page...

Mol	Chain	Res	Type
2	B	52	THR
2	B	69	ARG
2	B	164	GLU
2	B	168	ILE
2	B	183	ASP
2	B	192	LEU
2	B	197	ASN
2	B	218	SER
2	B	219	GLU
2	B	224	VAL
2	B	227	ARG
2	B	234	VAL
2	B	242	LEU
2	B	245	THR
2	B	254	ASP
2	B	257	TRP
2	B	260	GLN
2	B	264	LEU
2	B	267	VAL
2	B	279	ASN
2	B	297	HIS
2	B	299	ARG
2	B	304	TYR
2	B	313	LEU
2	B	314	GLN
2	B	315	PHE
2	B	317	TYR
2	B	402	LYS
2	B	421	MET
2	B	442	ARG
2	B	469	ARG
2	B	492	LEU
2	B	512	PHE
3	C	30	MET
3	C	39	SER
3	C	40	VAL
3	C	69	ARG
3	C	159	ASP
3	C	168	ILE
3	C	243	LEU
3	C	301	SER
3	C	317	TYR

Continued on next page...

Not For Manuscript Review

Continued from previous page...

Mol	Chain	Res	Type
3	C	319	ASN
3	C	421	MET
3	C	442	ARG
3	C	445	THR
3	C	449	THR
3	C	468	ARG
3	C	474	ASN
3	C	483	THR
3	C	492	LEU
3	C	523	PHE
3	C	524	MET
3	C	544	LEU
3	C	564	THR

Sometimes sidechains can be flipped to improve hydrogen bonding and reduce clashes. All (2) such sidechains are listed below:

Mol	Chain	Res	Type
2	B	260	GLN
3	C	474	ASN

5.3.3 RNA (i)

There are no RNA molecules in this entry.

5.4 Non-standard residues in protein, DNA, RNA chains (i)

There are no non-standard protein/DNA/RNA residues in this entry.

5.5 Carbohydrates (i)

There are no oligosaccharides in this entry.

5.6 Ligand geometry (i)

There are no ligands in this entry.

Not For Manuscript Review

5.7 Other polymers (i)

There are no such residues in this entry.

5.8 Polymer linkage issues (i)

There are no chain breaks in this entry.

Not For Manuscript Review

6 Map visualisation (i)

This section contains visualisations of the EMDB entry D_1292144934. These allow visual inspection of the internal detail of the map and identification of artifacts.

Images derived from a raw map, generated by summing the deposited half-maps, are presented below the corresponding image components of the primary map to allow further visual inspection and comparison with those of the primary map.

6.1 Orthogonal projections (i)

6.1.1 Primary map

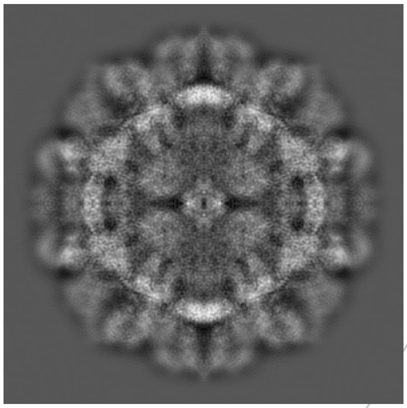
X

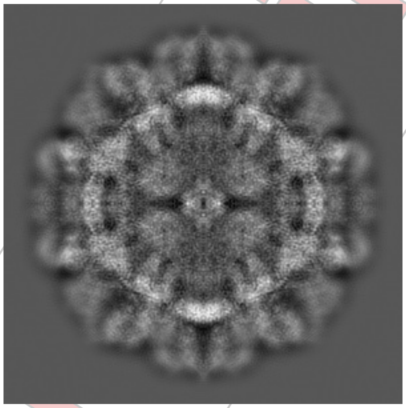
Y

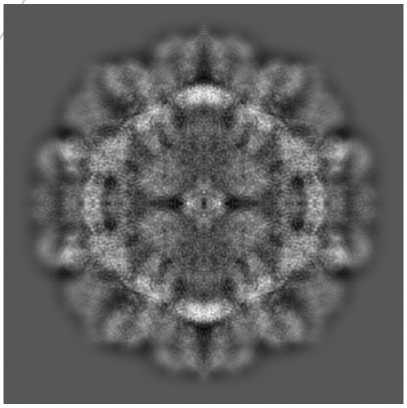
Z

6.1.2 Raw map

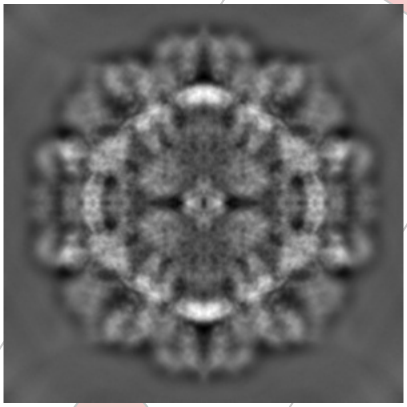
X

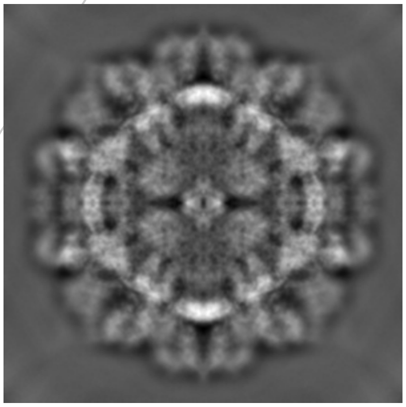
Y

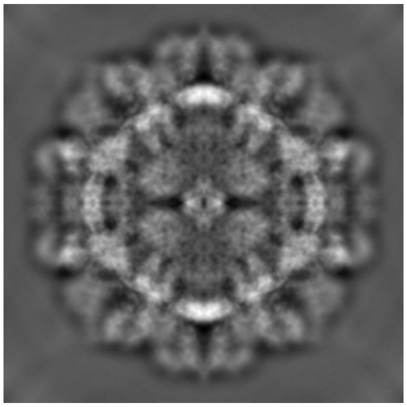
Z

The images above show the map projected in three orthogonal directions.

Not For Manuscript Review

6.2 Central slices (i)

6.2.1 Primary map

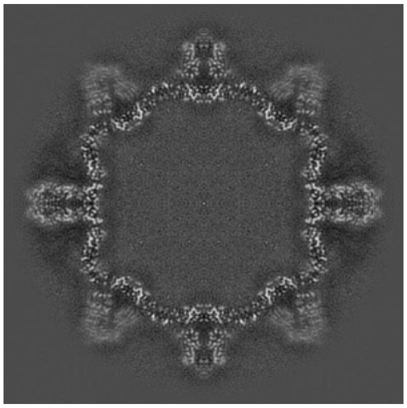

X Index: 220

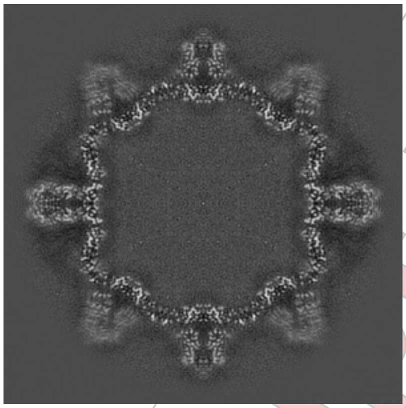

Y Index: 220

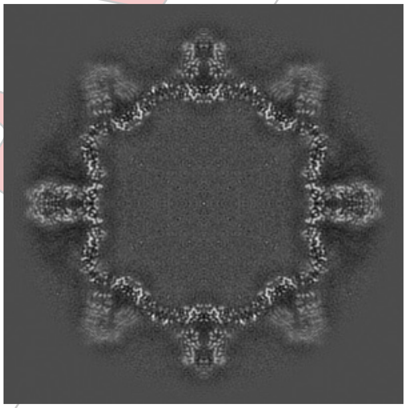

Z Index: 220

6.2.2 Raw map

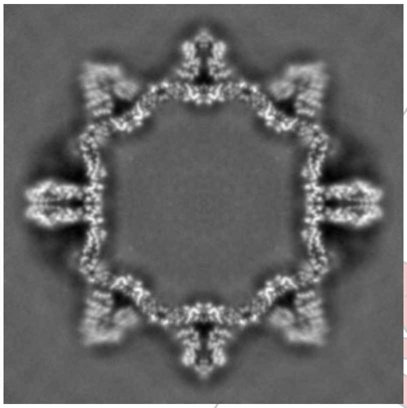

X Index: 220

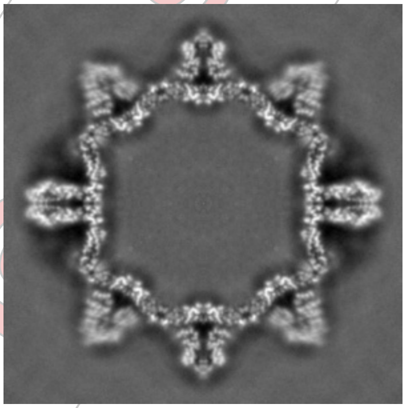

Y Index: 220

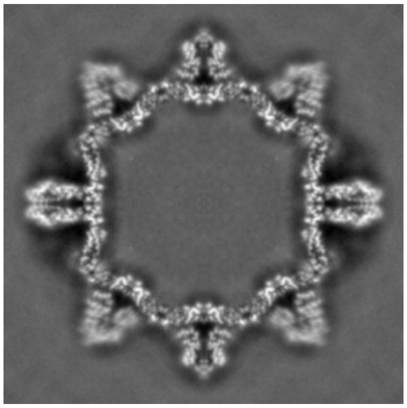

Z Index: 220

The images above show central slices of the map in three orthogonal directions.

Not For Manuscript Review

6.3 Largest variance slices (i)

6.3.1 Primary map

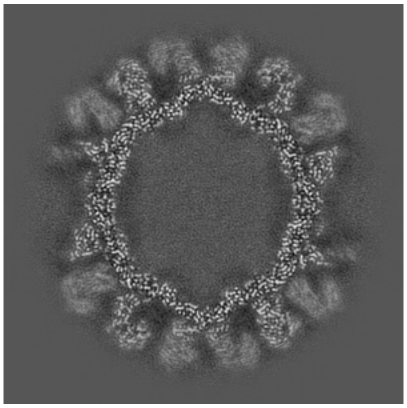

X Index: 159

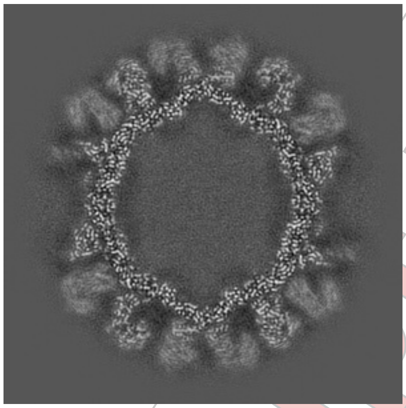

Y Index: 159

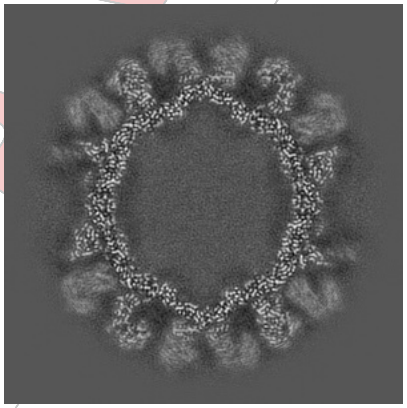

Z Index: 159

6.3.2 Raw map

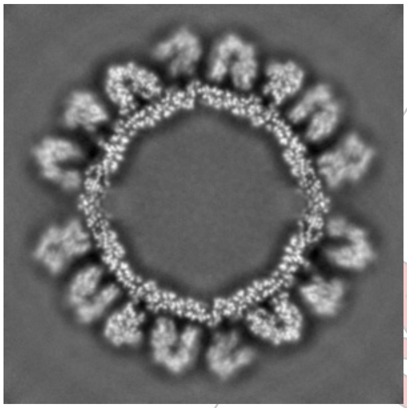

X Index: 261

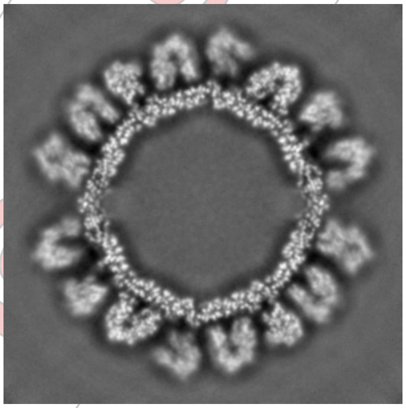

Y Index: 179

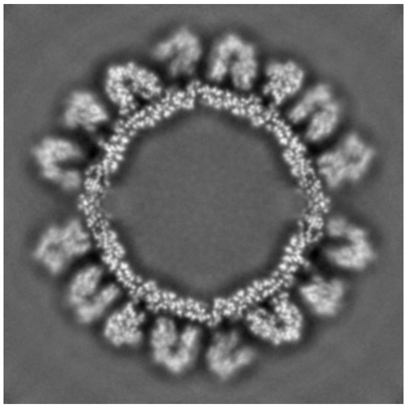

Z Index: 261

The images above show the largest variance slices of the map in three orthogonal directions.

Not For Manuscript Review

6.4 Orthogonal standard-deviation projections (False-color) (i)

6.4.1 Primary map

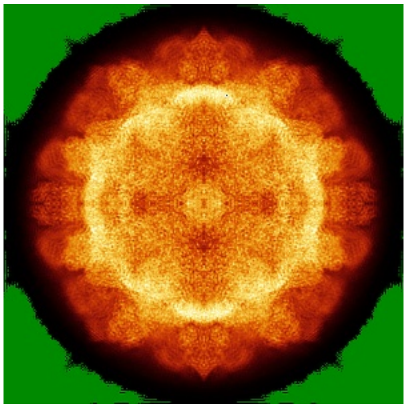
X

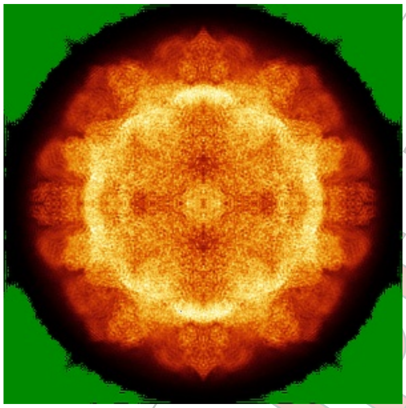
Y

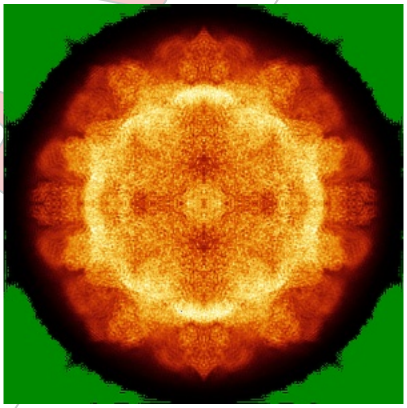
Z

6.4.2 Raw map

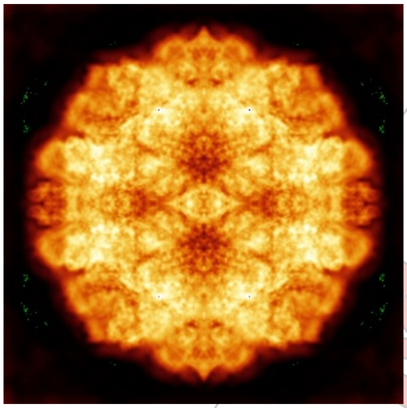
X

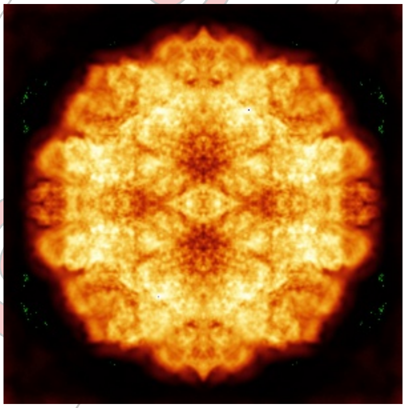
Y

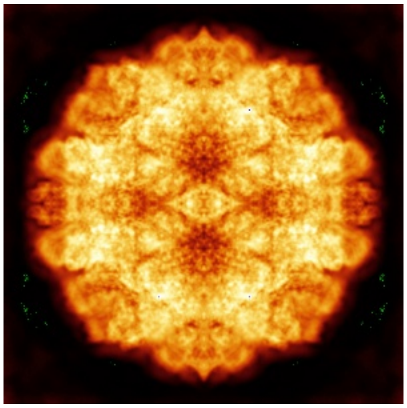
Z

The images above show the map standard deviation projections with false color in three orthogonal directions. Minimum values are shown in green, max in blue, and dark to light orange shades represent small to large values respectively.

Not For Manuscript Review

6.5 Orthogonal surface views (i)

6.5.1 Primary map

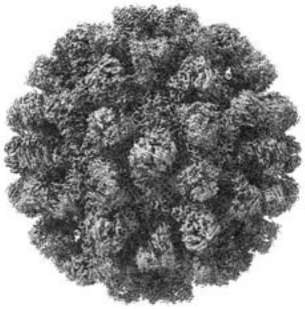

X

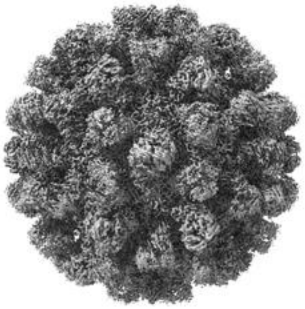

Y

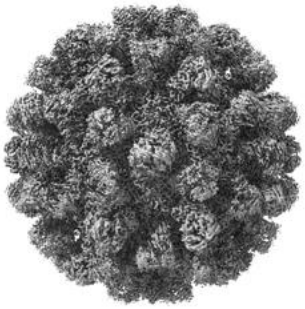

Z

The images above show the 3D surface view of the map at the recommended contour level 0.47. These images, in conjunction with the slice images, may facilitate assessment of whether an appropriate contour level has been provided.

6.5.2 Raw map

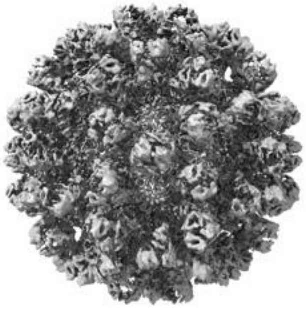

X

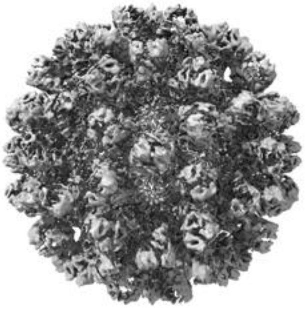

Y

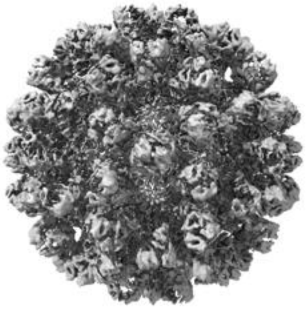

Z

These images show the 3D surface of the raw map. The raw map's contour level was selected so that its surface encloses the same volume as the primary map does at its recommended contour level.

6.6 Mask visualisation (i)

This section was not generated. No masks/segmentation were deposited.

Not For Manuscript Review

7 Map analysis (i)

This section contains the results of statistical analysis of the map.

7.1 Map-value distribution (i)

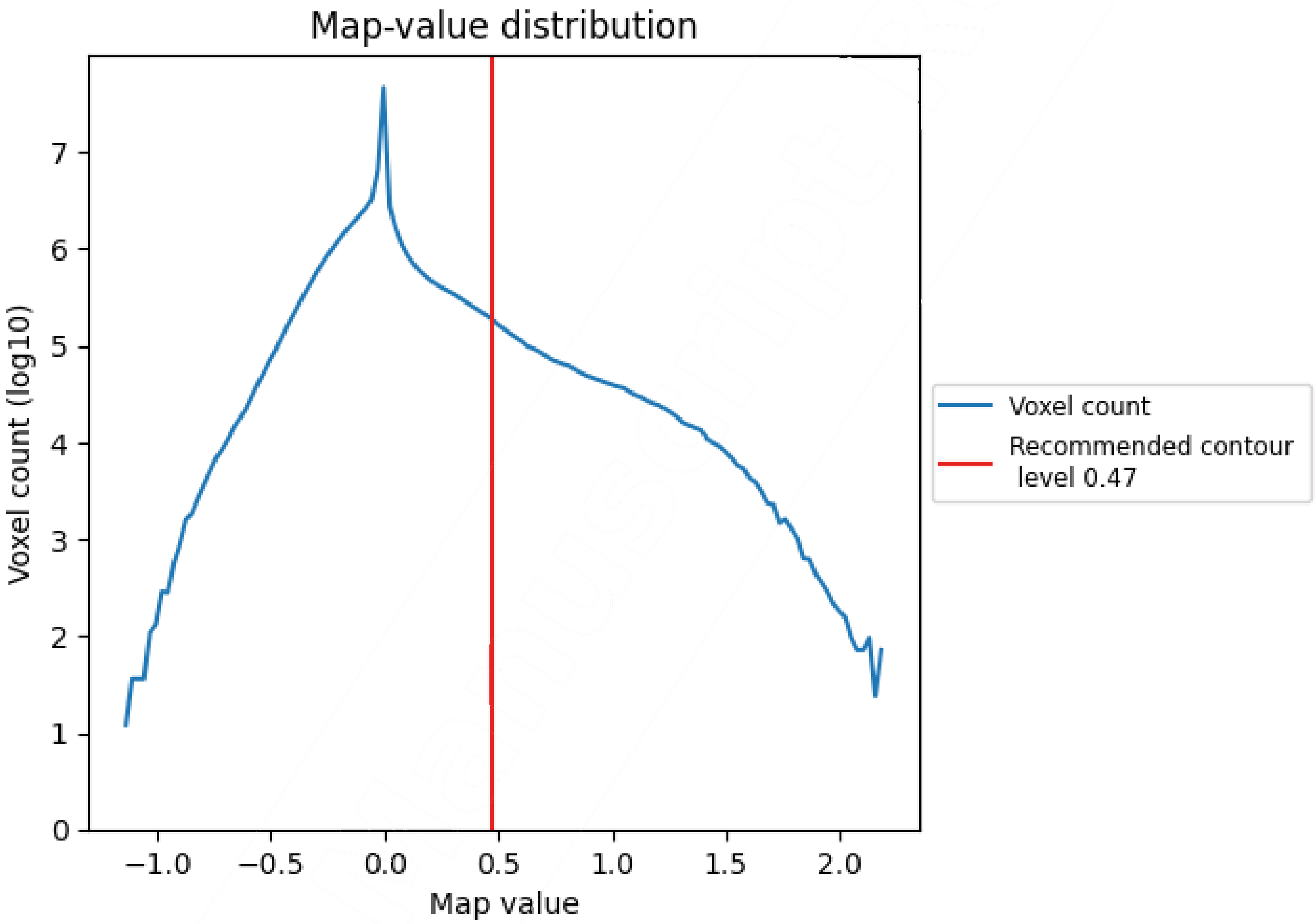

The map-value distribution is plotted in 128 intervals along the x-axis. The y-axis is logarithmic. A spike in this graph at zero usually indicates that the volume has been masked.

Not For Manuscript Review

7.2 Volume estimate (i)

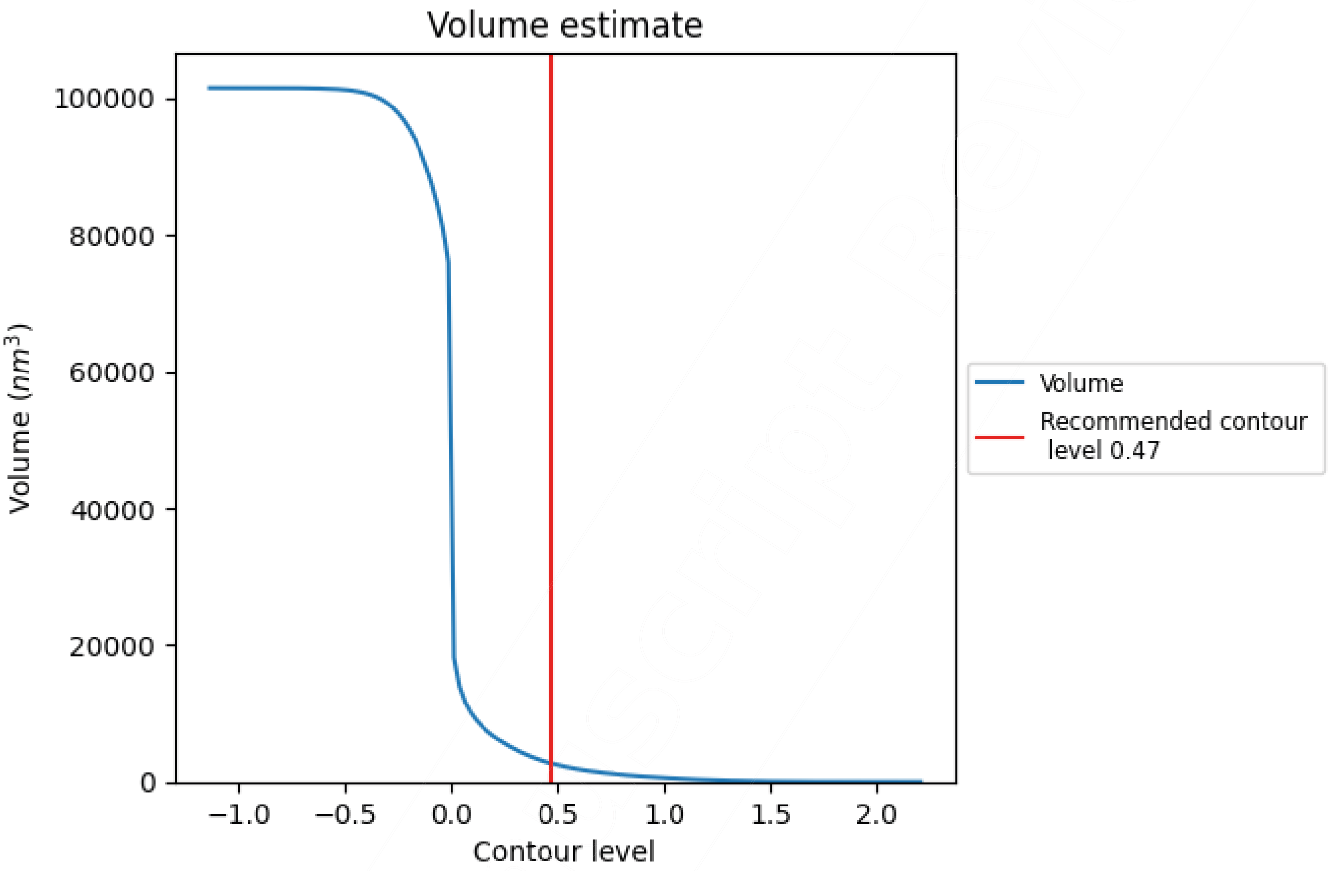

The volume at the recommended contour level is 2700 nm^3; this corresponds to an approximate mass of 2439 kDa.

The volume estimate graph shows how the enclosed volume varies with the contour level. The recommended contour level is shown as a vertical line and the intersection between the line and the curve gives the volume of the enclosed surface at the given level.

Not For Manuscript Review

7.3 Rotationally averaged power spectrum (i)

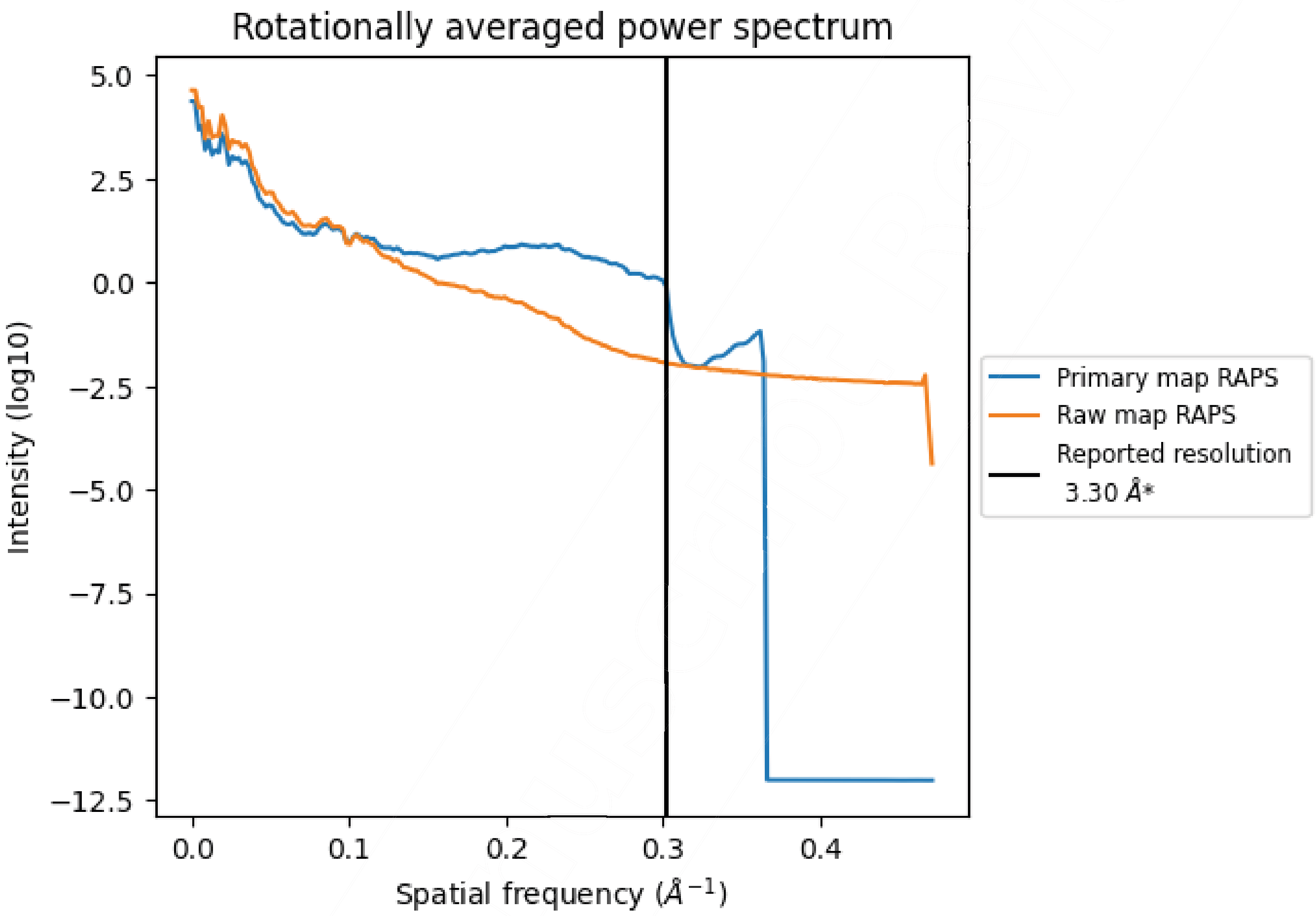

*Reported resolution corresponds to spatial frequency of 0.303 $Å^{-1}$

Not For Manuscript Review

8 Fourier-Shell correlation (i)

Fourier-Shell Correlation (FSC) is the most commonly used method to estimate the resolution of single-particle and subtomogram-averaged maps. The shape of the curve depends on the imposed symmetry, mask and whether or not the two 3D reconstructions used were processed from a common reference. The reported resolution is shown as a black line. A curve is displayed for the half-bit criterion in addition to lines showing the 0.143 gold standard cut-off and 0.5 cut-off.

8.1 FSC (i)

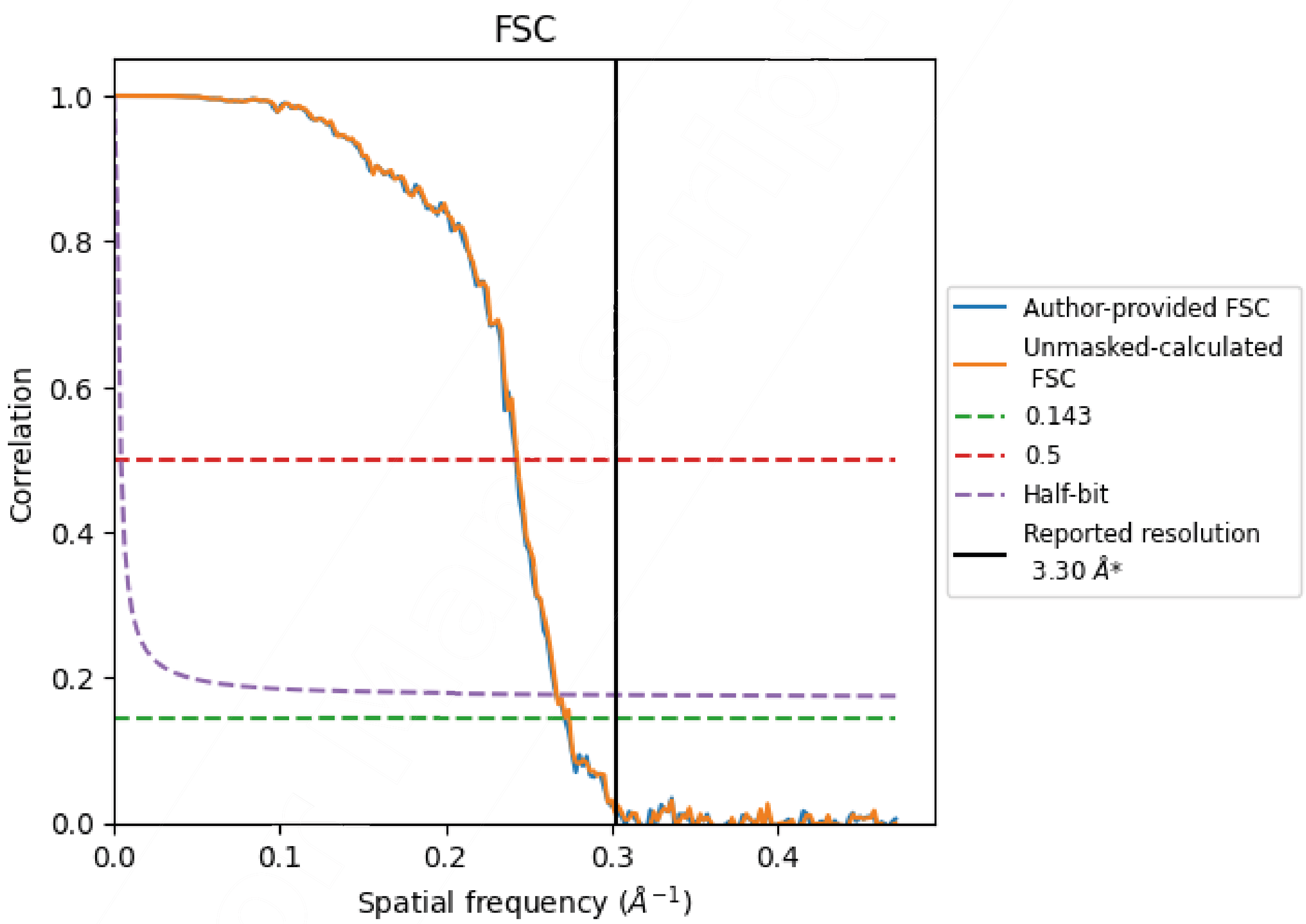

*Reported resolution corresponds to spatial frequency of 0.303 Å^{-1}

Not For Manuscript Review

8.2 Resolution estimates (i)

Resolution estimate (Å)	Estimation criterion (FSC cut-off)		
	0.143	0.5	Half-bit
Reported by author	3.30	-	-
Author-provided FSC curve	3.67	4.12	3.75
Unmasked-calculated*	3.64	4.11	3.74

*Resolution estimate based on FSC curve calculated by comparison of deposited half-maps. The value from author-provided FSC intersecting FSC 0.143 CUT-OFF 3.67 differs from the reported value 3.3 by more than 10 %

The value from deposited half-maps intersecting FSC 0.143 CUT-OFF 3.64 differs from the reported value 3.3 by more than 10 %

Not For Manuscript Review

9 Map-model fit (i)

This section contains information regarding the fit between EMDB map D_1292144934 and PDB model D_1292144934. Per-residue inclusion information can be found in section 3 on page 4.

9.1 Map-model overlays

9.1.1 Map-model overlay (i)

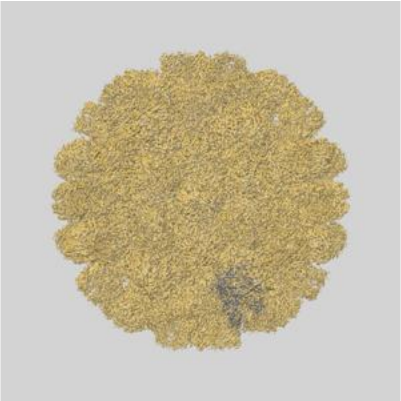

X

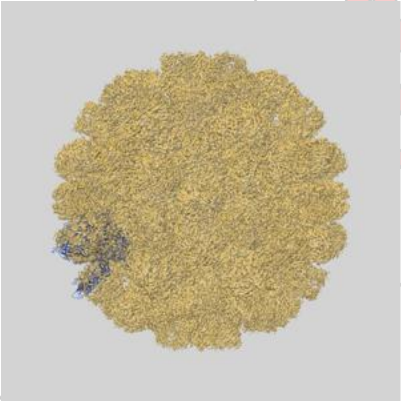

Y

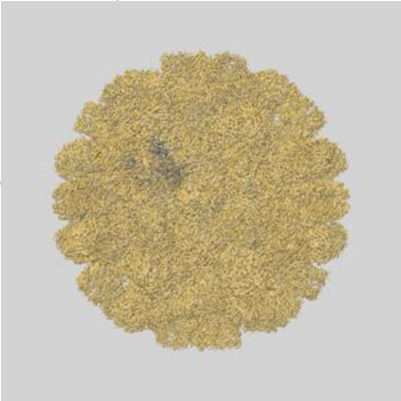

Z

The images above show the 3D surface view of the map at the recommended contour level 0.47 at 50% transparency in yellow overlaid with a ribbon representation of the model coloured in blue. These images allow for the visual assessment of the quality of fit between the atomic model and the map.

Not For Manuscript Review

9.2 Q-score mapped to coordinate model ⓘ

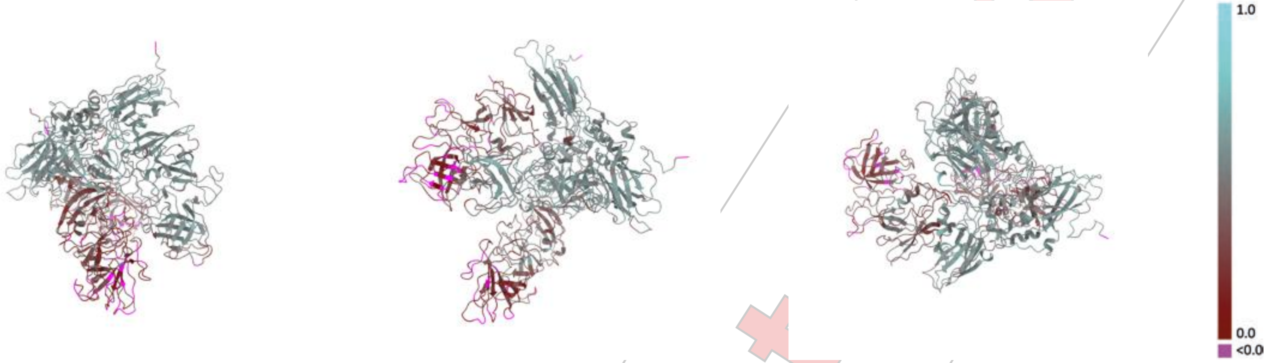

X Y Z

The images above show the model with each residue coloured according its Q-score. This shows their resolvability in the map with higher Q-score values reflecting better resolvability. Please note: Q-score is calculating the resolvability of atoms, and thus high values are only expected at resolutions at which atoms can be resolved. Low Q-score values may therefore be expected for many entries.

9.3 Atom inclusion mapped to coordinate model ⓘ

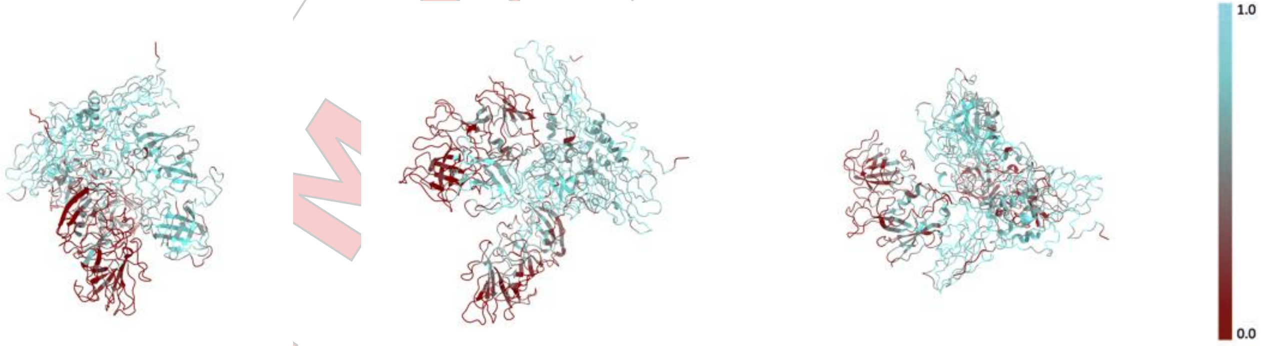

X Y Z

The images above show the model with each residue coloured according to its atom inclusion. This shows to what extent they are inside the map at the recommended contour level (0.47).

Not For Manuscript Review

9.4 Atom inclusion (i)

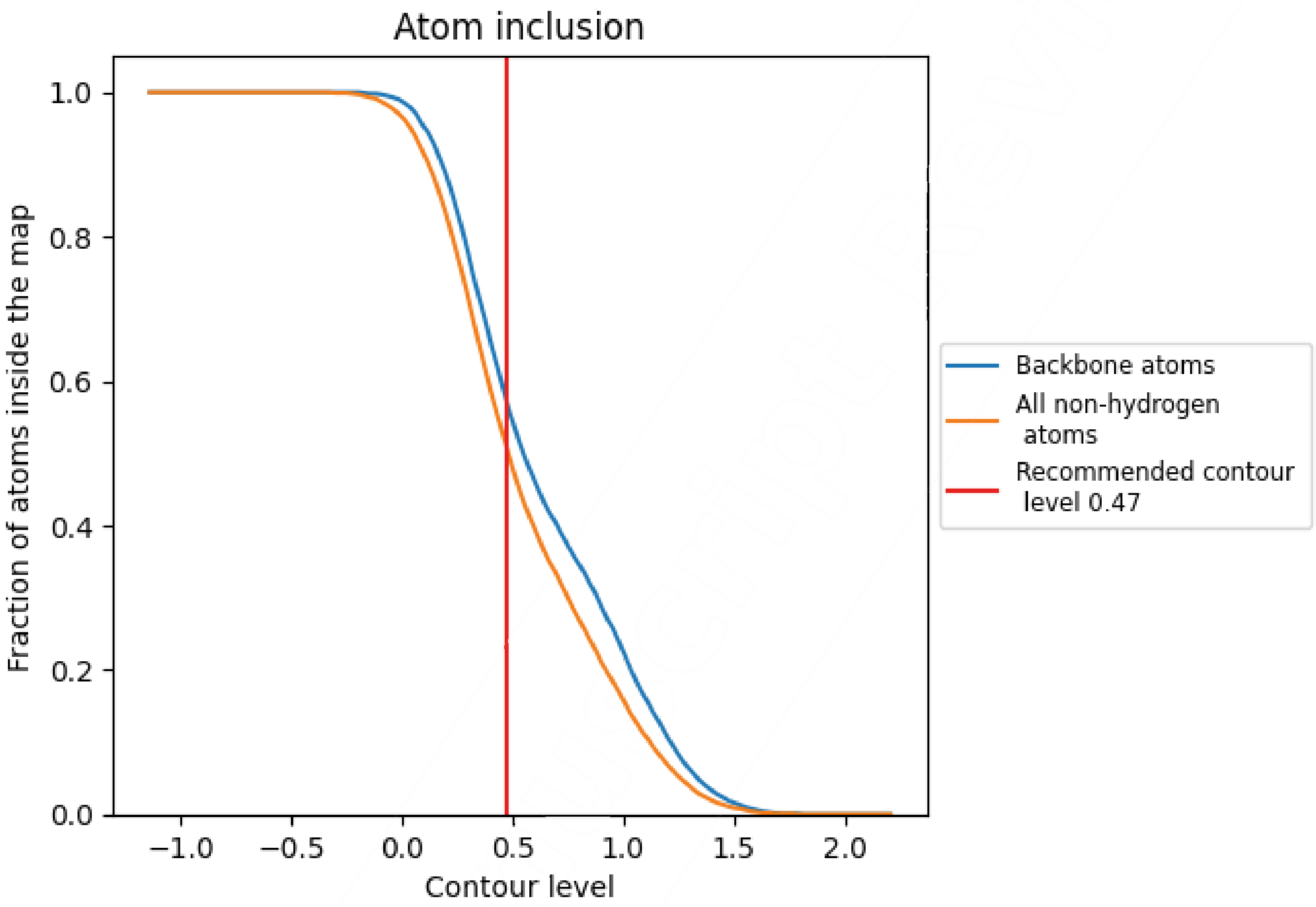

At the recommended contour level, 58% of all backbone atoms, 51% of all non-hydrogen atoms, are inside the map.

Not For Manuscript Review

9.5 Map-model fit summary (i)

The table lists the average atom inclusion at the recommended contour level (0.47) and Q-score for the entire model and for each chain.

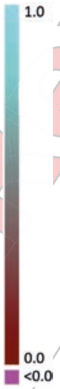

Chain	Atom inclusion	Q-score
All	0.5120	0.4130
A	0.4630	0.3800
B	0.3720	0.3410
C	0.7000	0.5160

Not For Manuscript Review

WORLDWIDE PDB PROTEIN DATA BANK

Full wwPDB EM Validation Report (i)

Jan 23, 2025 – 02:14 pm GMT

PDB ID	:	9I3E
EMDB ID	:	EMD-52588
Title	:	CP of RHDV mutant - 29N
Deposited on	:	2025-01-22
Resolution	:	3.00 Å(reported)

This wwPDB validation report is for manuscript review

For Manuscript Review

This is a Full wwPDB EM Validation Report.

This report is produced by the wwPDB biocuration pipeline after annotation of the structure.

We welcome your comments at *validation@mail.wwpdb.org*
A user guide is available at
https://www.wwpdb.org/validation/2017/EMValidationReportHelp
with specific help available everywhere you see the (i) symbol.

The types of validation reports are described at
http://www.wwpdb.org/validation/2017/FAQs#types.

The following versions of software and data (see references (i)) were used in the production of this report:

EMDB validation analysis	:	0.0.1.dev113
MolProbity	:	4.02b-467
Percentile statistics	:	20231227.v01 (using entries in the PDB archive December 27th 2023)
MapQ	:	1.9.13
Ideal geometry (proteins)	:	Engh & Huber (2001)
Ideal geometry (DNA, RNA)	:	Parkinson et al. (1996)
Validation Pipeline (wwPDB-VP)	:	2.40

1 Overall quality at a glance (i)

The following experimental techniques were used to determine the structure:
ELECTRON MICROSCOPY

The reported resolution of this entry is 3.00 Å.

Percentile scores (ranging between 0-100) for global validation metrics of the entry are shown in the following graphic. The table shows the number of entries on which the scores are based.

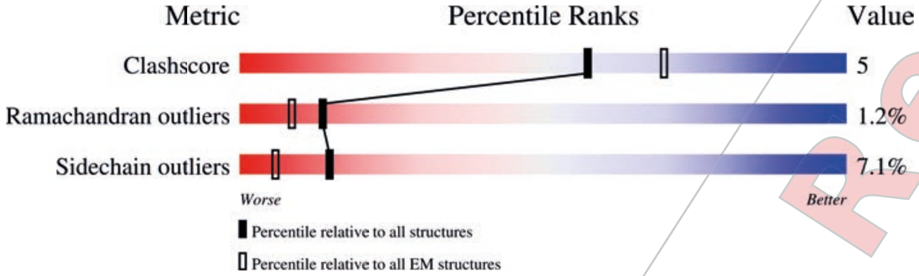

Metric	Whole archive (#Entries)	EM structures (#Entries)
Clashscore	210492	15764
Ramachandran outliers	207382	16835
Sidechain outliers	206894	16415

The table below summarises the geometric issues observed across the polymeric chains and their fit to the map. The red, orange, yellow and green segments of the bar indicate the fraction of residues that contain outliers for >=3, 2, 1 and 0 types of geometric quality criteria respectively. A grey segment represents the fraction of residues that are not modelled. The numeric value for each fraction is indicated below the corresponding segment, with a dot representing fractions <=5% The upper red bar (where present) indicates the fraction of residues that have poor fit to the EM map (all-atom inclusion < 40%). The numeric value is given above the bar.

Mol	Chain	Length	Quality of chain
1	0	174	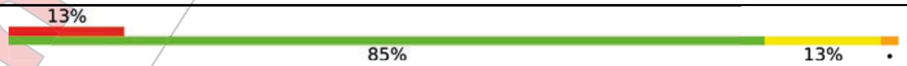

For Manuscript Review

2 Entry composition (i)

There is only 1 type of molecule in this entry. The entry contains 1329 atoms, of which 0 are hydrogens and 0 are deuteriums.

In the tables below, the AltConf column contains the number of residues with at least one atom in alternate conformation and the Trace column contains the number of residues modelled with at most 2 atoms.

- Molecule 1 is a protein called Capsid structural protein VP60.

Mol	Chain	Residues	Atoms					AltConf	Trace
			Total	C	N	O	S		
1	0	174	1329	852	225	247	5	0	0

There are 6 discrepancies between the modelled and reference sequences:

Chain	Residue	Modelled	Actual	Comment	Reference
0	122	ALA	ILE	conflict	UNP Q9YND5
0	128	ALA	VAL	conflict	UNP Q9YND5
0	133	VAL	LEU	conflict	UNP Q9YND5
0	134	ALA	VAL	conflict	UNP Q9YND5
0	193	ALA	SER	conflict	UNP Q9YND5
0	197	ALA	ASN	conflict	UNP Q9YND5

For Manuscript Review

3 Residue-property plots (i)

These plots are drawn for all protein, RNA, DNA and oligosaccharide chains in the entry. The first graphic for a chain summarises the proportions of the various outlier classes displayed in the second graphic. The second graphic shows the sequence view annotated by issues in geometry and atom inclusion in map density. Residues are color-coded according to the number of geometric quality criteria for which they contain at least one outlier: green = 0, yellow = 1, orange = 2 and red = 3 or more. A red diamond above a residue indicates a poor fit to the EM map for this residue (all-atom inclusion < 40%). Stretches of 2 or more consecutive residues without any outlier are shown as a green connector. Residues present in the sample, but not in the model, are shown in grey.

- Molecule 1: Capsid structural protein VP60

Chain 0:

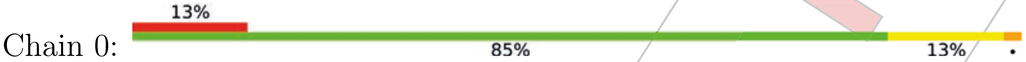

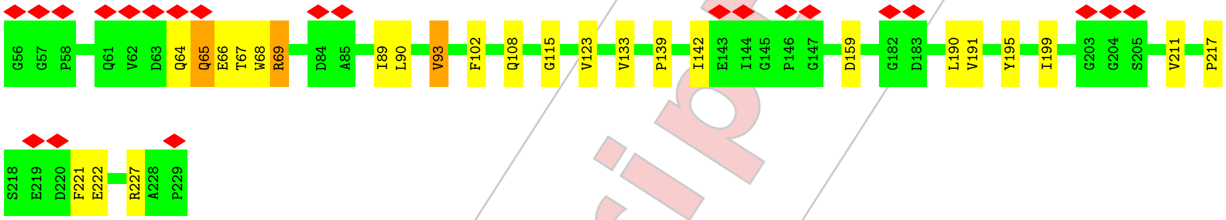

For Manuscript Review

4 Experimental information (i)

Property	Value	Source
EM reconstruction method	SINGLE PARTICLE	Depositor
Imposed symmetry	POINT, Not provided	
Number of particles used	302069	Depositor
Resolution determination method	FSC 0.143 CUT-OFF	Depositor
CTF correction method	PHASE FLIPPING AND AMPLITUDE CORRECTION	Depositor
Microscope	TFS TALOS	Depositor
Voltage (kV)	200	Depositor
Electron dose ($e^-/\text{Å}^2$)	31.9	Depositor
Minimum defocus (nm)	800	Depositor
Maximum defocus (nm)	2300	Depositor
Magnification	Not provided	
Image detector	FEI FALCON III (4k x 4k)	Depositor
Maximum map value	6.088	Depositor
Minimum map value	-3.422	Depositor
Average map value	0.062	Depositor
Map value standard deviation	0.382	Depositor
Recommended contour level	1.4	Depositor
Map size (Å)	328.8, 328.8, 328.8	wwPDB
Map dimensions	240, 240, 240	wwPDB
Map angles (°)	90.0, 90.0, 90.0	wwPDB
Pixel spacing (Å)	1.37, 1.37, 1.37	Depositor

For Manuscript Review

5 Model quality (i)

5.1 Standard geometry (i)

The Z score for a bond length (or angle) is the number of standard deviations the observed value is removed from the expected value. A bond length (or angle) with $|Z| > 5$ is considered an outlier worth inspection. RMSZ is the root-mean-square of all Z scores of the bond lengths (or angles).

Mol	Chain	Bond lengths		Bond angles	
		RMSZ	#$|Z|$ >5	RMSZ	#$|Z|$ >5
1	0	0.35	0/1371	0.58	0/1882

Chiral center outliers are detected by calculating the chiral volume of a chiral center and verifying if the center is modelled as a planar moiety or with the opposite hand.A planarity outlier is detected by checking planarity of atoms in a peptide group, atoms in a mainchain group or atoms of a sidechain that are expected to be planar.

Mol	Chain	#Chirality outliers	#Planarity outliers
1	0	0	1

There are no bond length outliers.

There are no bond angle outliers.

There are no chirality outliers.

All (1) planarity outliers are listed below:

Mol	Chain	Res	Type	Group
1	0	227	ARG	Sidechain

5.2 Too-close contacts (i)

In the following table, the Non-H and H(model) columns list the number of non-hydrogen atoms and hydrogen atoms in the chain respectively. The H(added) column lists the number of hydrogen atoms added and optimized by MolProbity. The Clashes column lists the number of clashes within the asymmetric unit, whereas Symm-Clashes lists symmetry-related clashes.

Mol	Chain	Non-H	H(model)	H(added)	Clashes	Symm-Clashes
1	0	1329	0	1276	12	0
All	All	1329	0	1276	12	0

The all-atom clashscore is defined as the number of clashes found per 1000 atoms (including hydrogen atoms). The all-atom clashscore for this structure is 5.

For Manuscript Review

All (12) close contacts within the same asymmetric unit are listed below, sorted by their clash magnitude.

Atom-1	Atom-2	Interatomic distance (Å)	Clash overlap (Å)
1:0:69:ARG:HA	1:0:217:PRO:HD2	1.75	0.67
1:0:139:PRO:HB2	1:0:142:ILE:HD13	1.83	0.60
1:0:68:TRP:CD1	1:0:102:PHE:HB2	2.41	0.56
1:0:123:VAL:HG13	1:0:211:VAL:HG22	1.89	0.53
1:0:115:GLY:HA3	1:0:221:PHE:HA	1.93	0.51
1:0:64:GLN:O	1:0:66:GLU:N	2.43	0.51
1:0:69:ARG:HB2	1:0:217:PRO:HB2	1.95	0.48
1:0:93:VAL:HG12	1:0:190:LEU:HB3	1.97	0.46
1:0:66:GLU:O	1:0:68:TRP:N	2.51	0.44
1:0:64:GLN:HA	1:0:69:ARG:HD2	2.00	0.43
1:0:69:ARG:HH12	1:0:221:PHE:HB3	1.86	0.41
1:0:89:ILE:HG23	1:0:191:VAL:HG13	2.03	0.40

There are no symmetry-related clashes.

5.3 Torsion angles (i)

5.3.1 Protein backbone (i)

In the following table, the Percentiles column shows the percent Ramachandran outliers of the chain as a percentile score with respect to all PDB entries followed by that with respect to all EM entries.

The Analysed column shows the number of residues for which the backbone conformation was analysed, and the total number of residues.

Mol	Chain	Analysed	Favoured	Allowed	Outliers	Percentiles
1	0	172/174 (99%)	158 (92%)	12 (7%)	2 (1%)	11 41

All (2) Ramachandran outliers are listed below:

Mol	Chain	Res	Type
1	0	65	GLN
1	0	67	THR

For Manuscript Review

5.3.2 Protein sidechains (i)

In the following table, the Percentiles column shows the percent sidechain outliers of the chain as a percentile score with respect to all PDB entries followed by that with respect to all EM entries.

The Analysed column shows the number of residues for which the sidechain conformation was analysed, and the total number of residues.

Mol	Chain	Analysed	Rotameric	Outliers	Percentiles
1	0	141/141 (100%)	131 (93%)	10 (7%)	12 40

All (10) residues with a non-rotameric sidechain are listed below:

Mol	Chain	Res	Type
1	0	65	GLN
1	0	69	ARG
1	0	90	LEU
1	0	93	VAL
1	0	108	GLN
1	0	133	VAL
1	0	159	ASP
1	0	195	TYR
1	0	199	ILE
1	0	222	GLU

Sometimes sidechains can be flipped to improve hydrogen bonding and reduce clashes. There are no such sidechains identified.

5.3.3 RNA (i)

There are no RNA molecules in this entry.

5.4 Non-standard residues in protein, DNA, RNA chains (i)

There are no non-standard protein/DNA/RNA residues in this entry.

5.5 Carbohydrates (i)

There are no oligosaccharides in this entry.

For Manuscript Review

5.6 Ligand geometry (i)

There are no ligands in this entry.

5.7 Other polymers (i)

There are no such residues in this entry.

5.8 Polymer linkage issues (i)

There are no chain breaks in this entry.

For Manuscript Review

6 Map visualisation ⓘ

This section contains visualisations of the EMDB entry EMD-52588. These allow visual inspection of the internal detail of the map and identification of artifacts.

Images derived from a raw map, generated by summing the deposited half-maps, are presented below the corresponding image components of the primary map to allow further visual inspection and comparison with those of the primary map.

6.1 Orthogonal projections ⓘ

6.1.1 Primary map

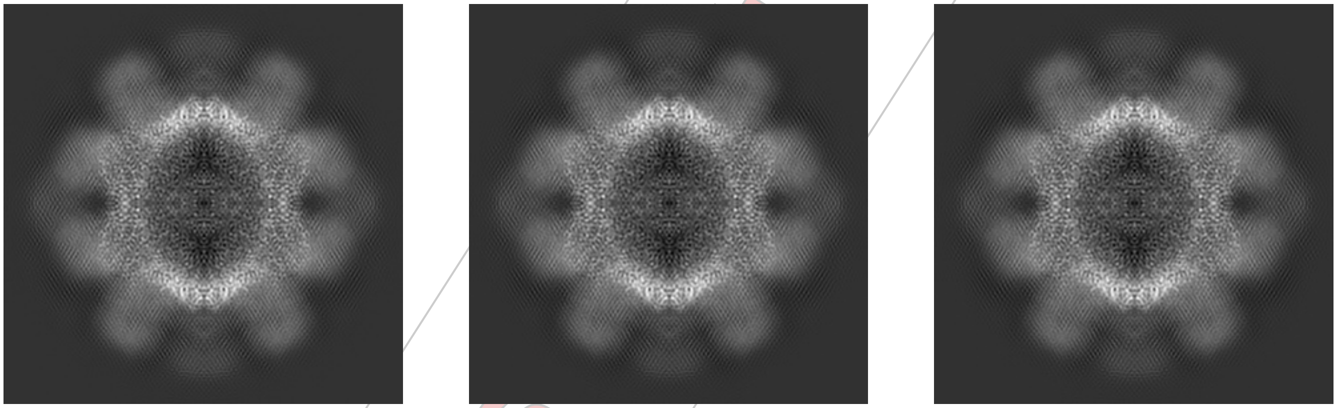

X Y Z

6.1.2 Raw map

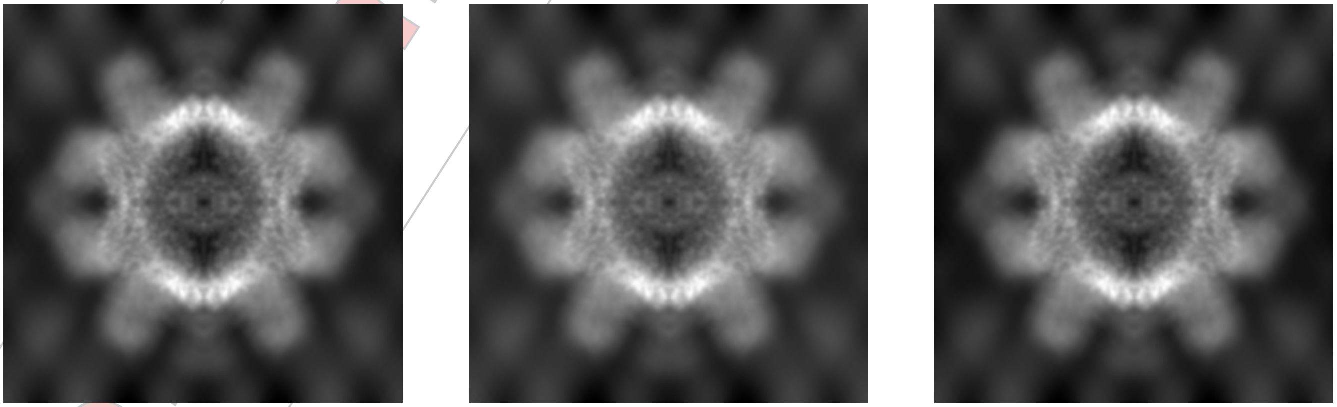

X Y Z

The images above show the map projected in three orthogonal directions.

For Manuscript Review

6.2 Central slices ⓘ

6.2.1 Primary map

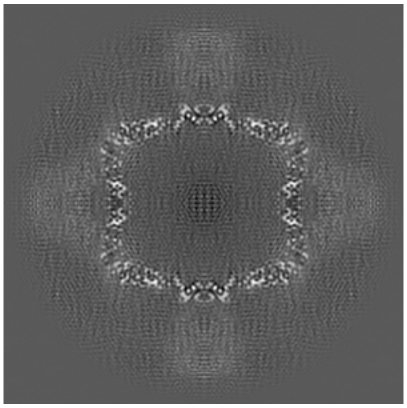
X Index: 120

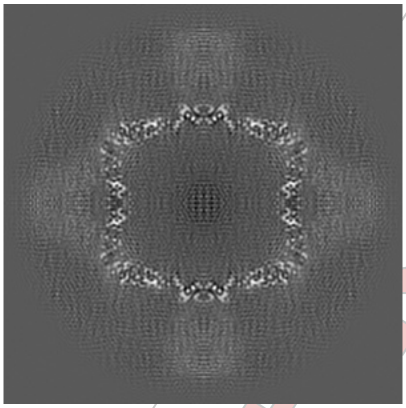
Y Index: 120

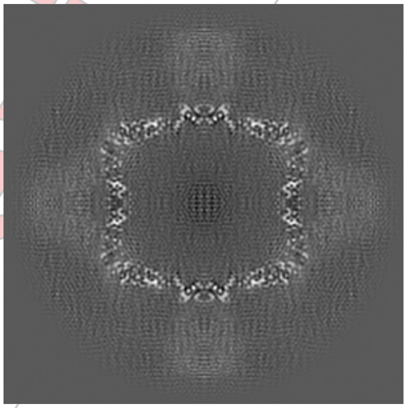
Z Index: 120

6.2.2 Raw map

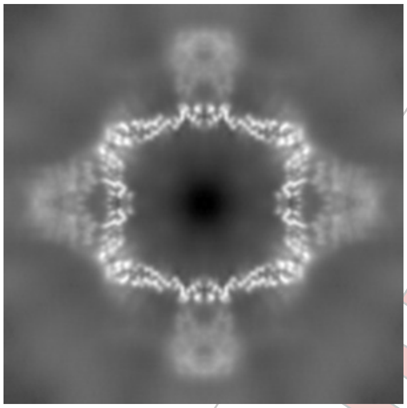
X Index: 120

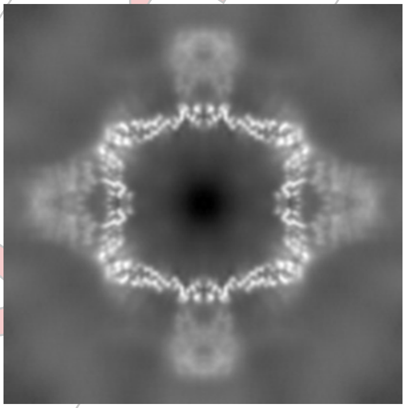
Y Index: 120

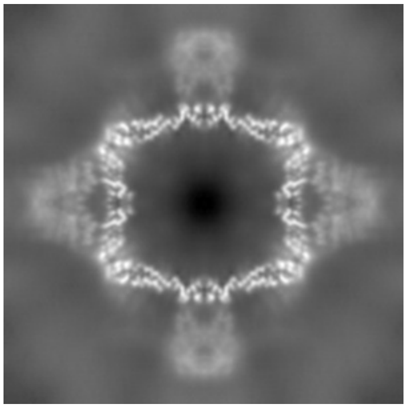
Z Index: 120

The images above show central slices of the map in three orthogonal directions.

For Manuscript Review

6.3 Largest variance slices (i)

6.3.1 Primary map

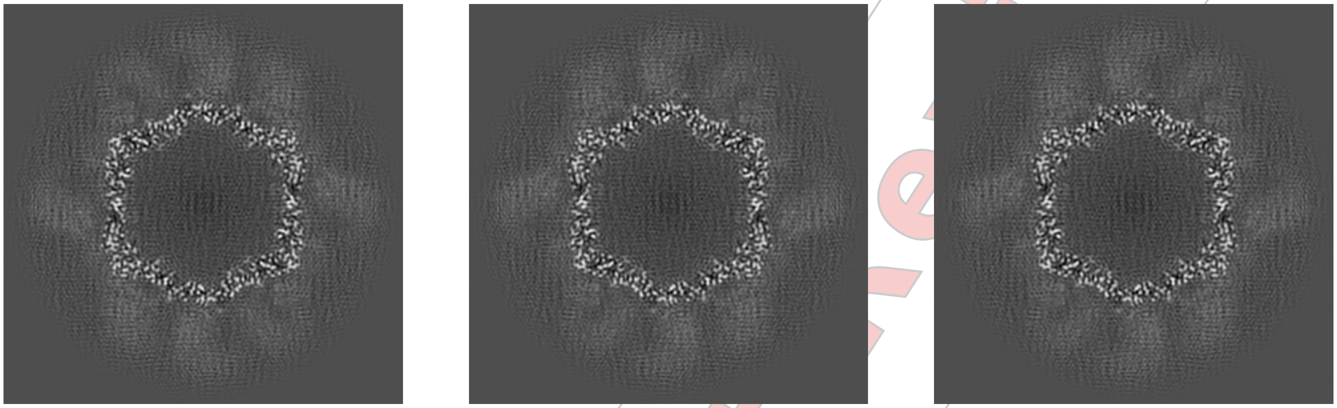

X Index: 107 Y Index: 133 Z Index: 133

6.3.2 Raw map

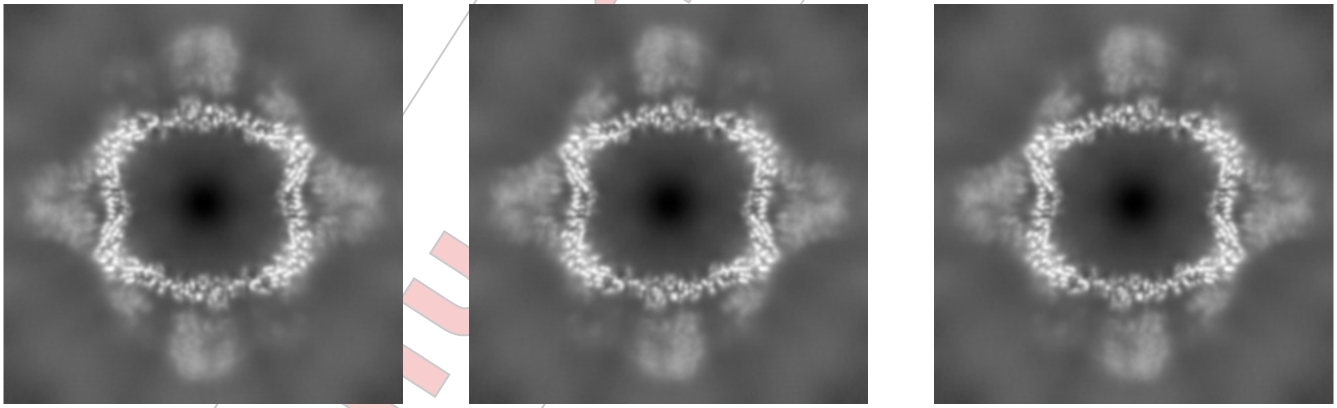

X Index: 116 Y Index: 124 Z Index: 124

The images above show the largest variance slices of the map in three orthogonal directions.

For Manuscript Review

6.4 Orthogonal standard-deviation projections (False-color) (i)

6.4.1 Primary map

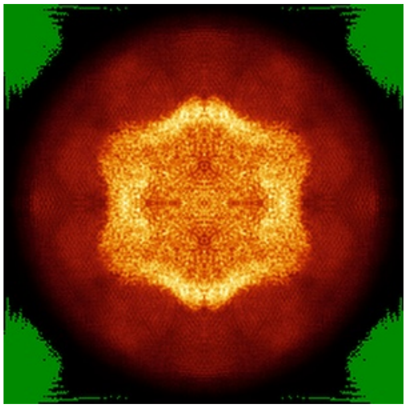
X

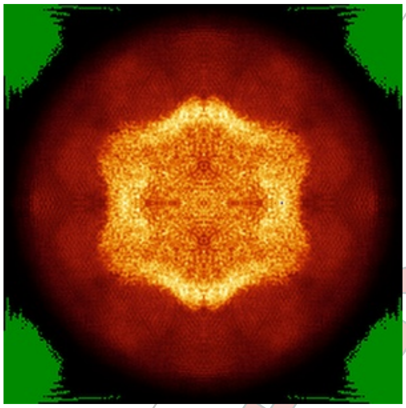
Y

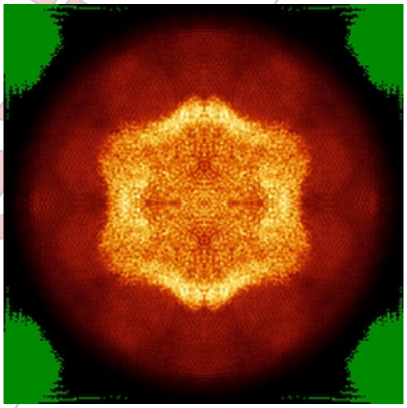
Z

6.4.2 Raw map

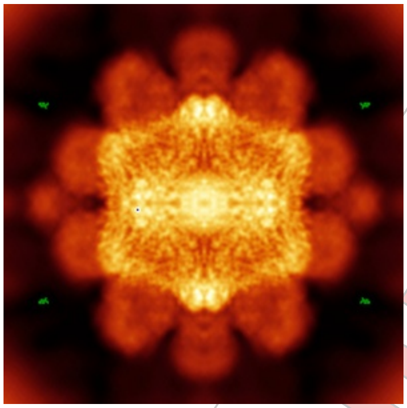
X

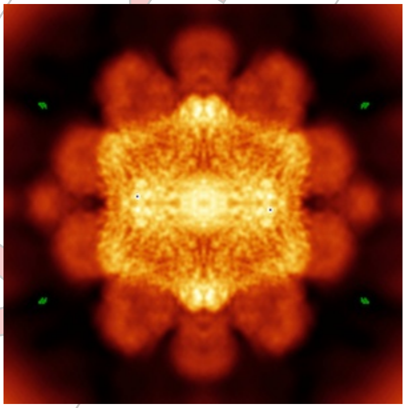
Y

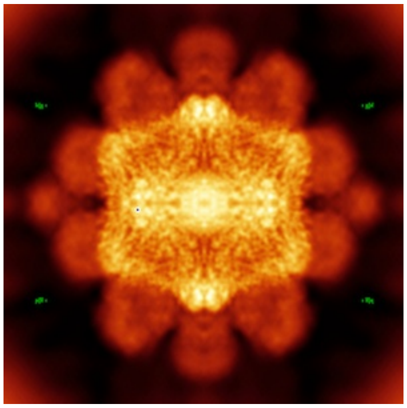
Z

The images above show the map standard deviation projections with false color in three orthogonal directions. Minimum values are shown in green, max in blue, and dark to light orange shades represent small to large values respectively.

For Manuscript Review

6.5 Orthogonal surface views (i)

6.5.1 Primary map

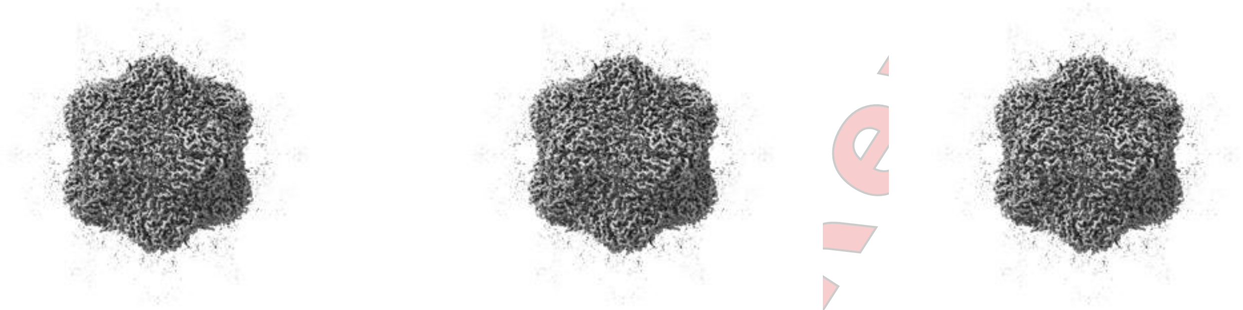

X Y Z

The images above show the 3D surface view of the map at the recommended contour level 1.4. These images, in conjunction with the slice images, may facilitate assessment of whether an appropriate contour level has been provided.

6.5.2 Raw map

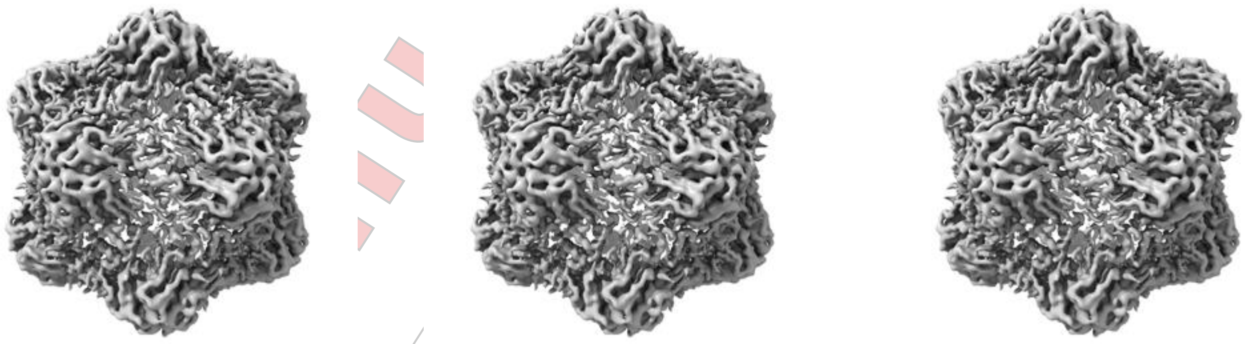

X Y Z

These images show the 3D surface of the raw map. The raw map's contour level was selected so that its surface encloses the same volume as the primary map does at its recommended contour level.

6.6 Mask visualisation (i)

This section was not generated. No masks/segmentation were deposited.

For Manuscript Review

7 Map analysis (i)

This section contains the results of statistical analysis of the map.

7.1 Map-value distribution (i)

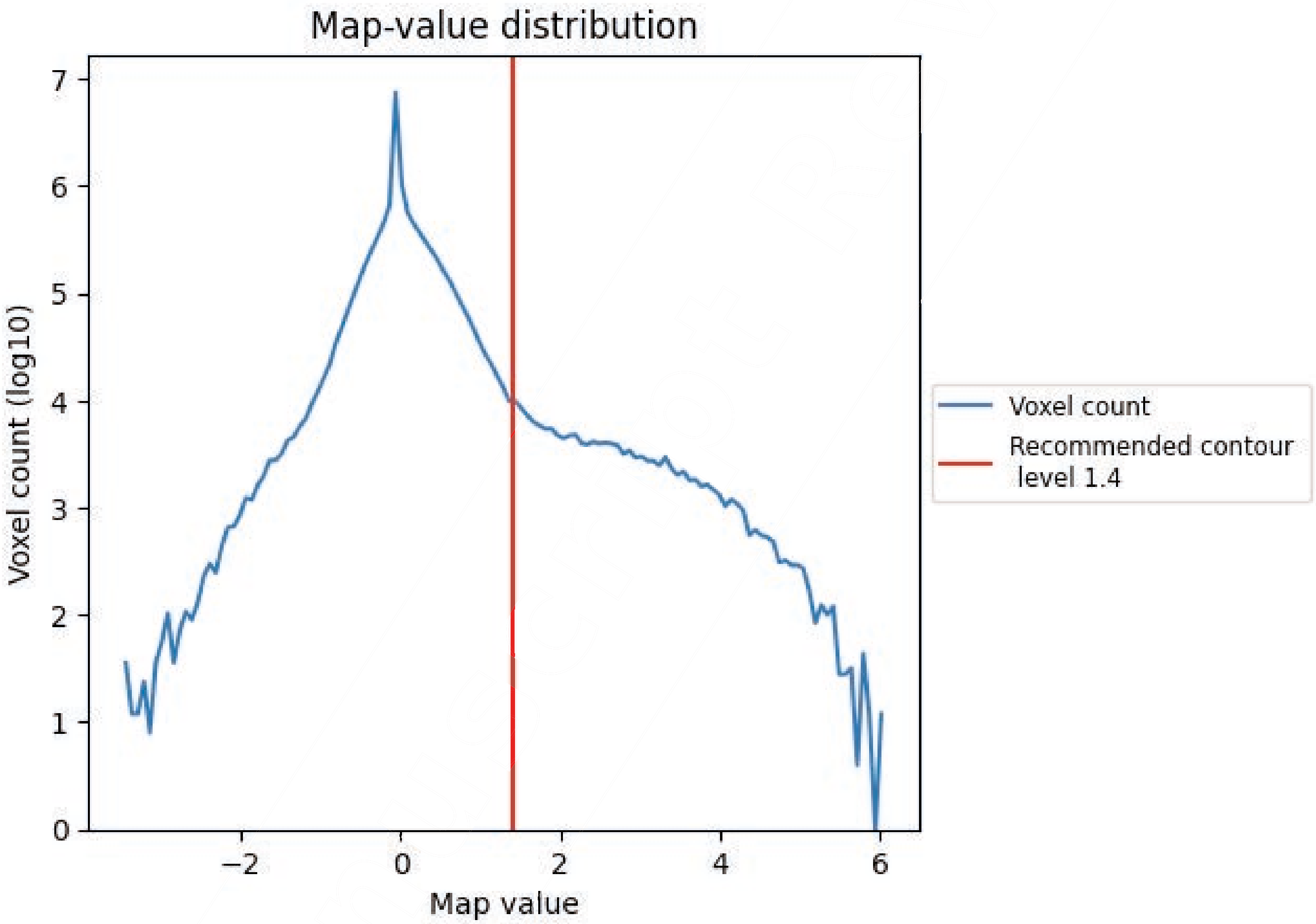

The map-value distribution is plotted in 128 intervals along the x-axis. The y-axis is logarithmic. A spike in this graph at zero usually indicates that the volume has been masked.

For Manuscript Review

7.2 Volume estimate (i)

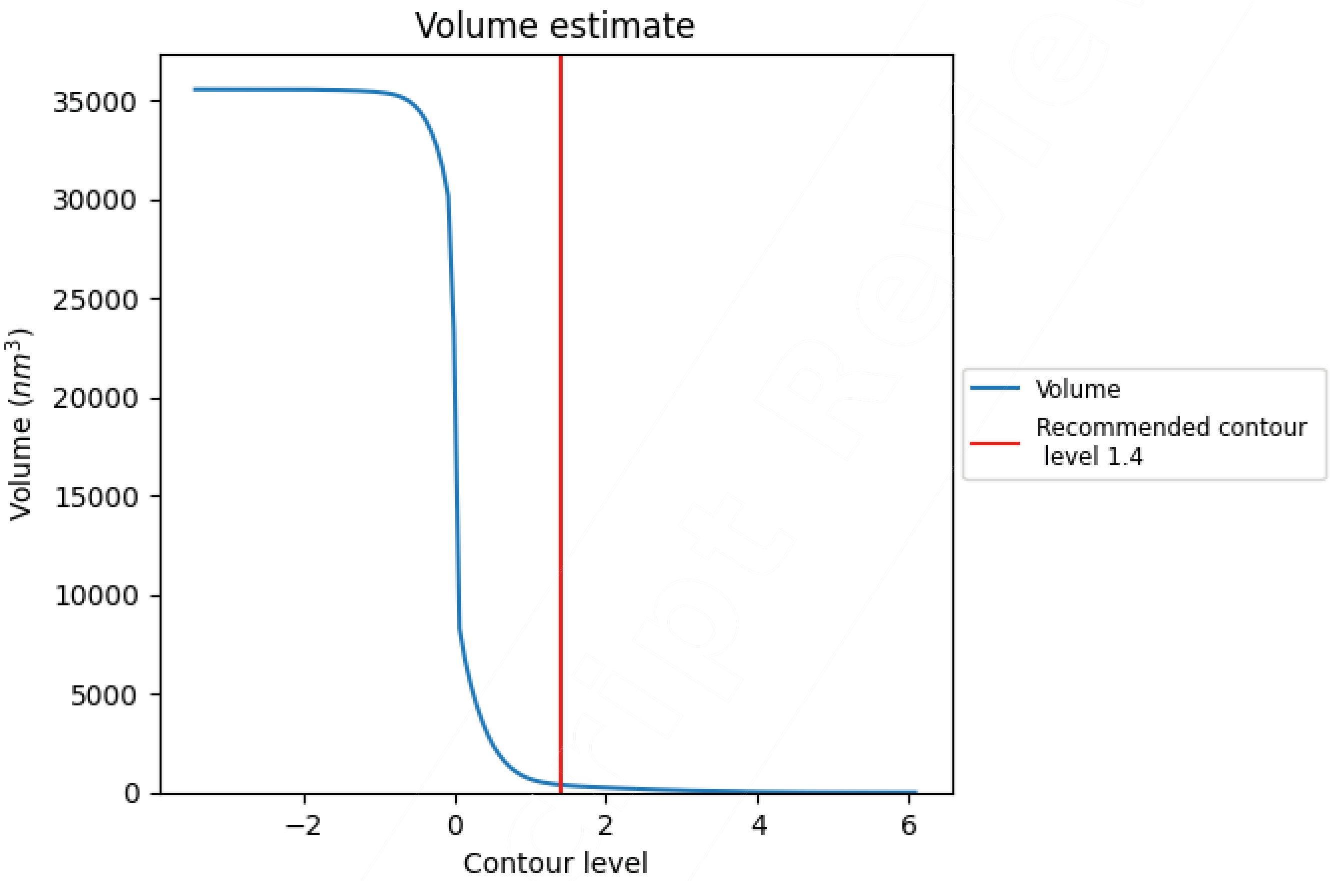

The volume at the recommended contour level is 384 nm^3; this corresponds to an approximate mass of 347 kDa.

The volume estimate graph shows how the enclosed volume varies with the contour level. The recommended contour level is shown as a vertical line and the intersection between the line and the curve gives the volume of the enclosed surface at the given level.

For Manuscript Review

7.3 Rotationally averaged power spectrum (i)

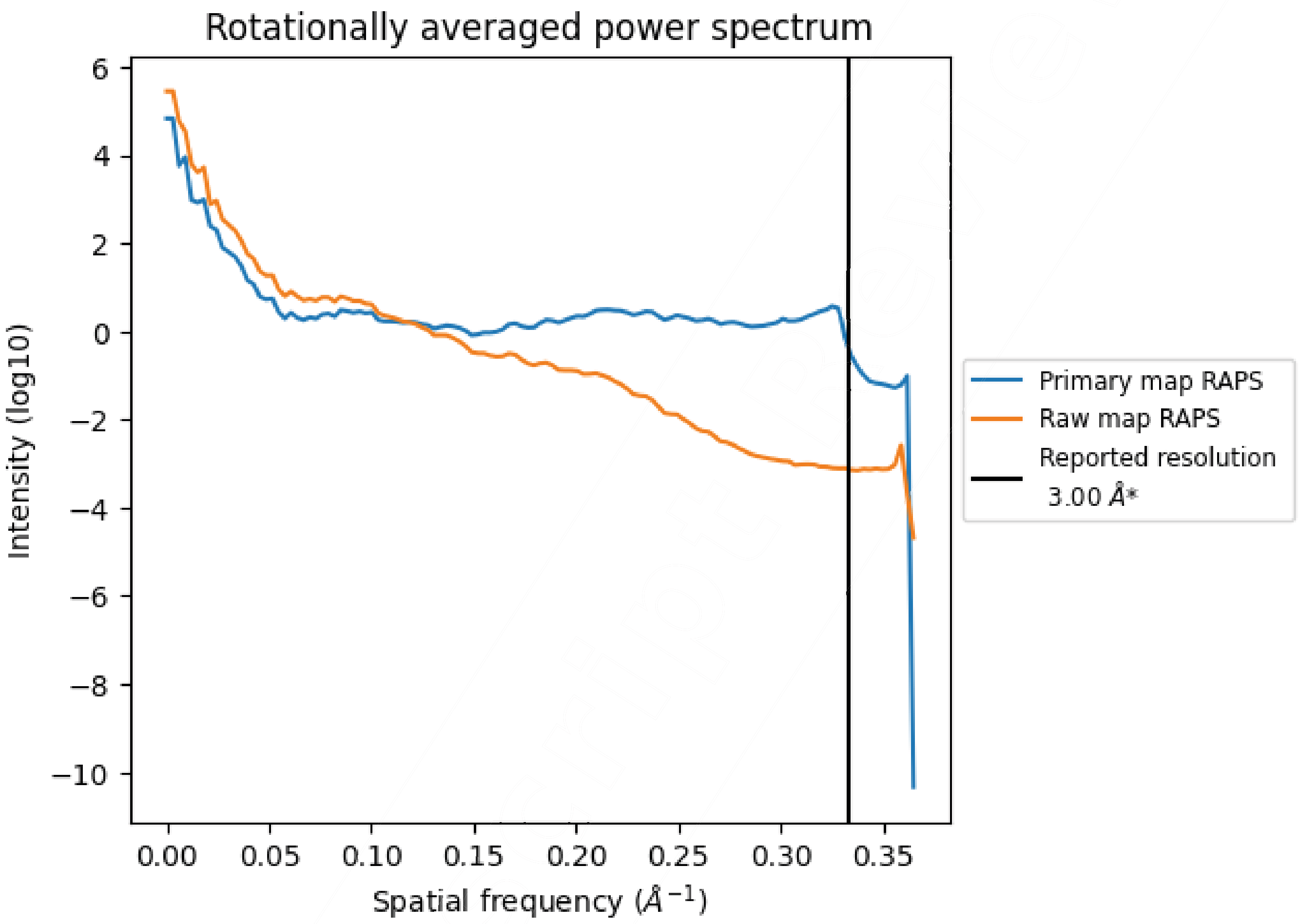

*Reported resolution corresponds to spatial frequency of 0.333 Å^{-1}

For Manuscript Review

8 Fourier-Shell correlation ⓘ

Fourier-Shell Correlation (FSC) is the most commonly used method to estimate the resolution of single-particle and subtomogram-averaged maps. The shape of the curve depends on the imposed symmetry, mask and whether or not the two 3D reconstructions used were processed from a common reference. The reported resolution is shown as a black line. A curve is displayed for the half-bit criterion in addition to lines showing the 0.143 gold standard cut-off and 0.5 cut-off.

8.1 FSC ⓘ

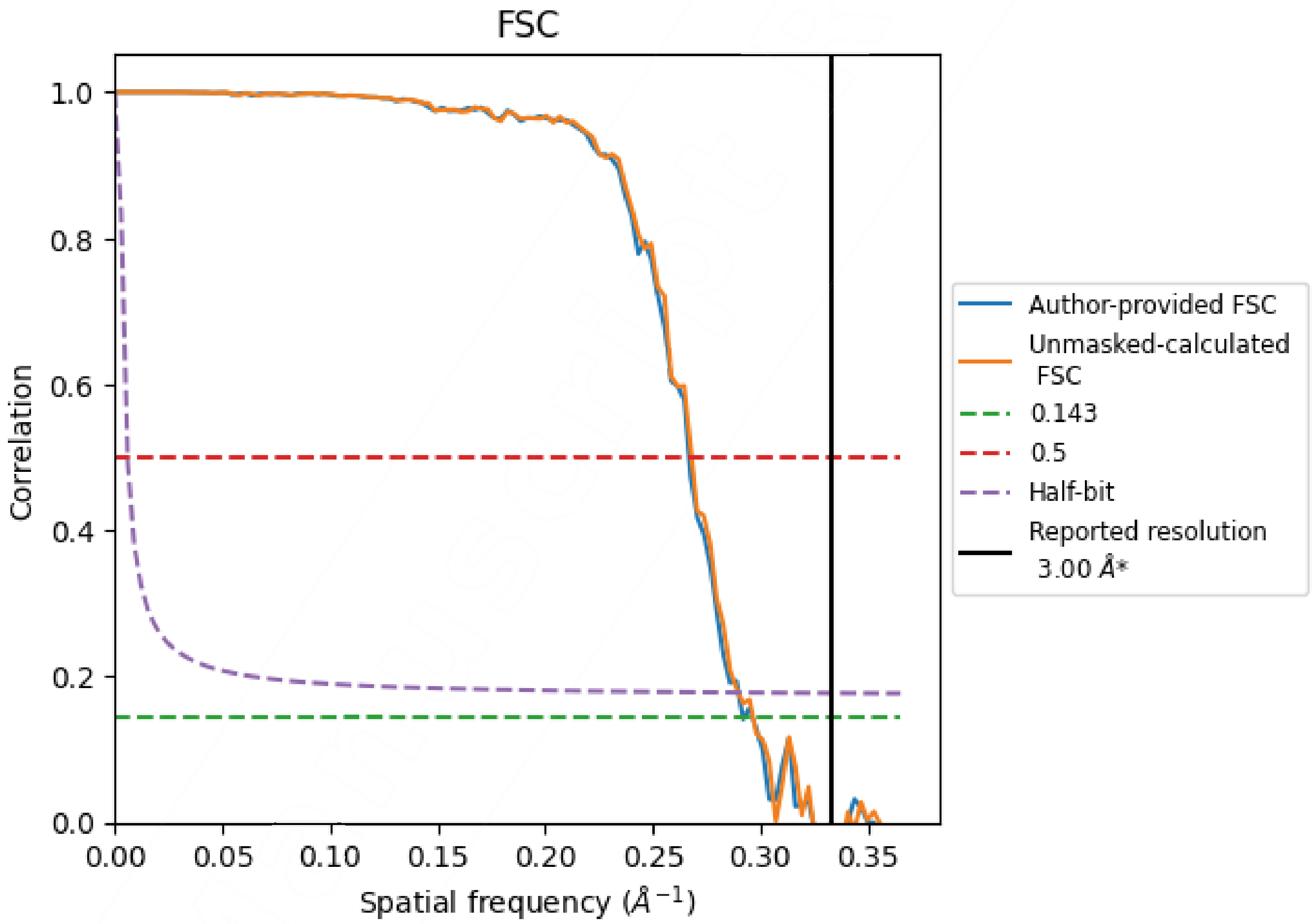

*Reported resolution corresponds to spatial frequency of 0.333 $Å^{-1}$

For Manuscript Review

8.2 Resolution estimates (i)

Resolution estimate (Å)	Estimation criterion (FSC cut-off)		
	0.143	0.5	Half-bit
Reported by author	3.00	-	-
Author-provided FSC curve	3.43	3.75	3.45
Unmasked-calculated*	3.37	3.73	3.45

*Resolution estimate based on FSC curve calculated by comparison of deposited half-maps. The value from author-provided FSC intersecting FSC 0.143 CUT-OFF 3.43 differs from the reported value 3.0 by more than 10 %

The value from deposited half-maps intersecting FSC 0.143 CUT-OFF 3.37 differs from the reported value 3.0 by more than 10 %

For Manuscript Review

9 Map-model fit (i)

This section contains information regarding the fit between EMDB map EMD-52588 and PDB model 9I3E. Per-residue inclusion information can be found in section 3 on page 4.

9.1 Map-model overlays

9.1.1 Map-model overlay (i)

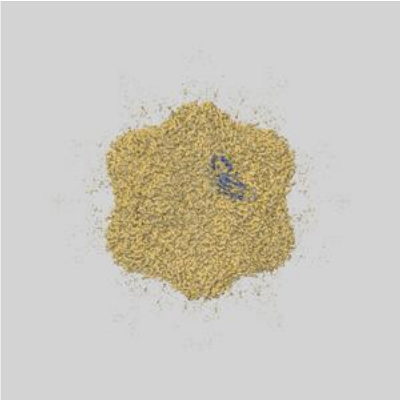

X

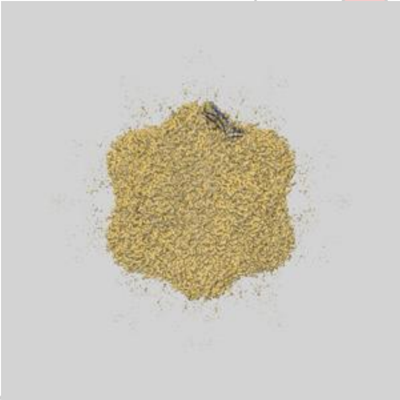

Y

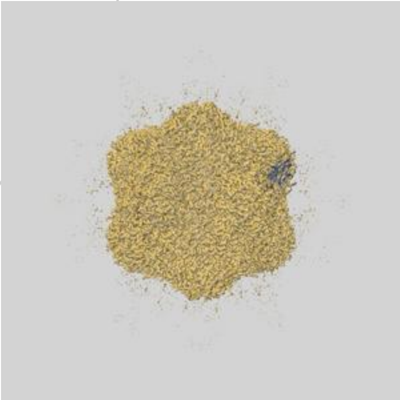

Z

9.1.2 Map-model assembly overlay (i)

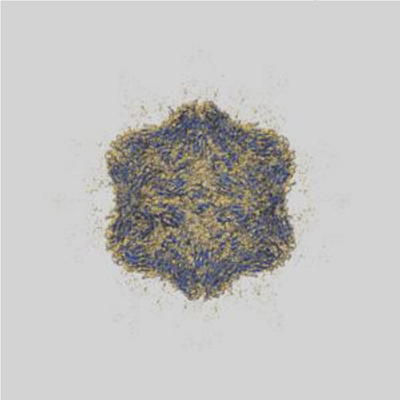

X

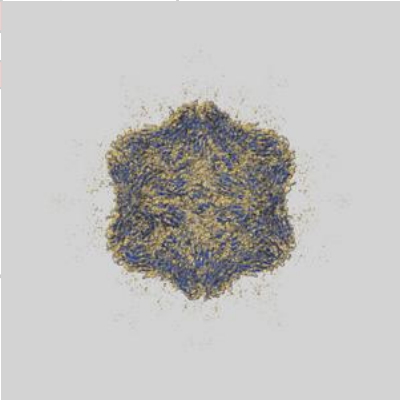

Y

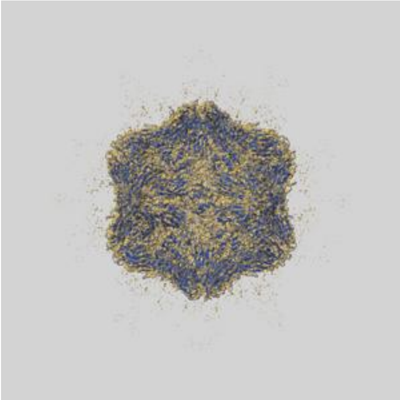

Z

The images above show the 3D surface view of the map at the recommended contour level 1.4 at 50% transparency in yellow overlaid with a ribbon representation of the model coloured in blue. These images allow for the visual assessment of the quality of fit between the atomic model and the map.

For Manuscript Review

9.2 Q-score mapped to coordinate model (i)

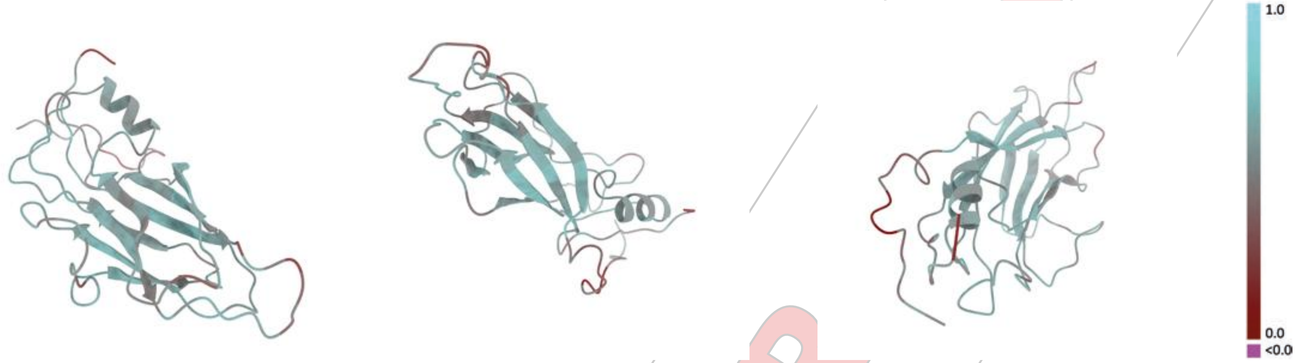

X Y Z

The images above show the model with each residue coloured according its Q-score. This shows their resolvability in the map with higher Q-score values reflecting better resolvability. Please note: Q-score is calculating the resolvability of atoms, and thus high values are only expected at resolutions at which atoms can be resolved. Low Q-score values may therefore be expected for many entries.

9.3 Atom inclusion mapped to coordinate model (i)

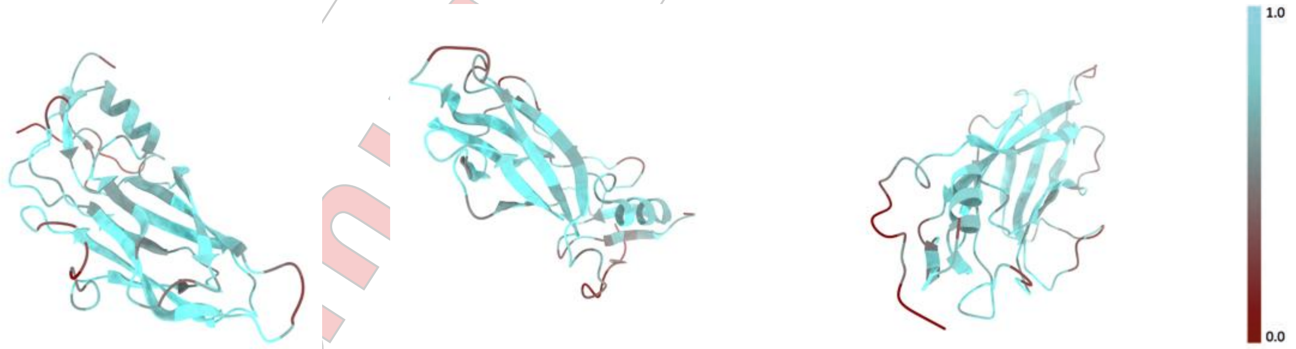

X Y Z

The images above show the model with each residue coloured according to its atom inclusion. This shows to what extent they are inside the map at the recommended contour level (1.4).

For Manuscript Review

9.4 Atom inclusion (i)

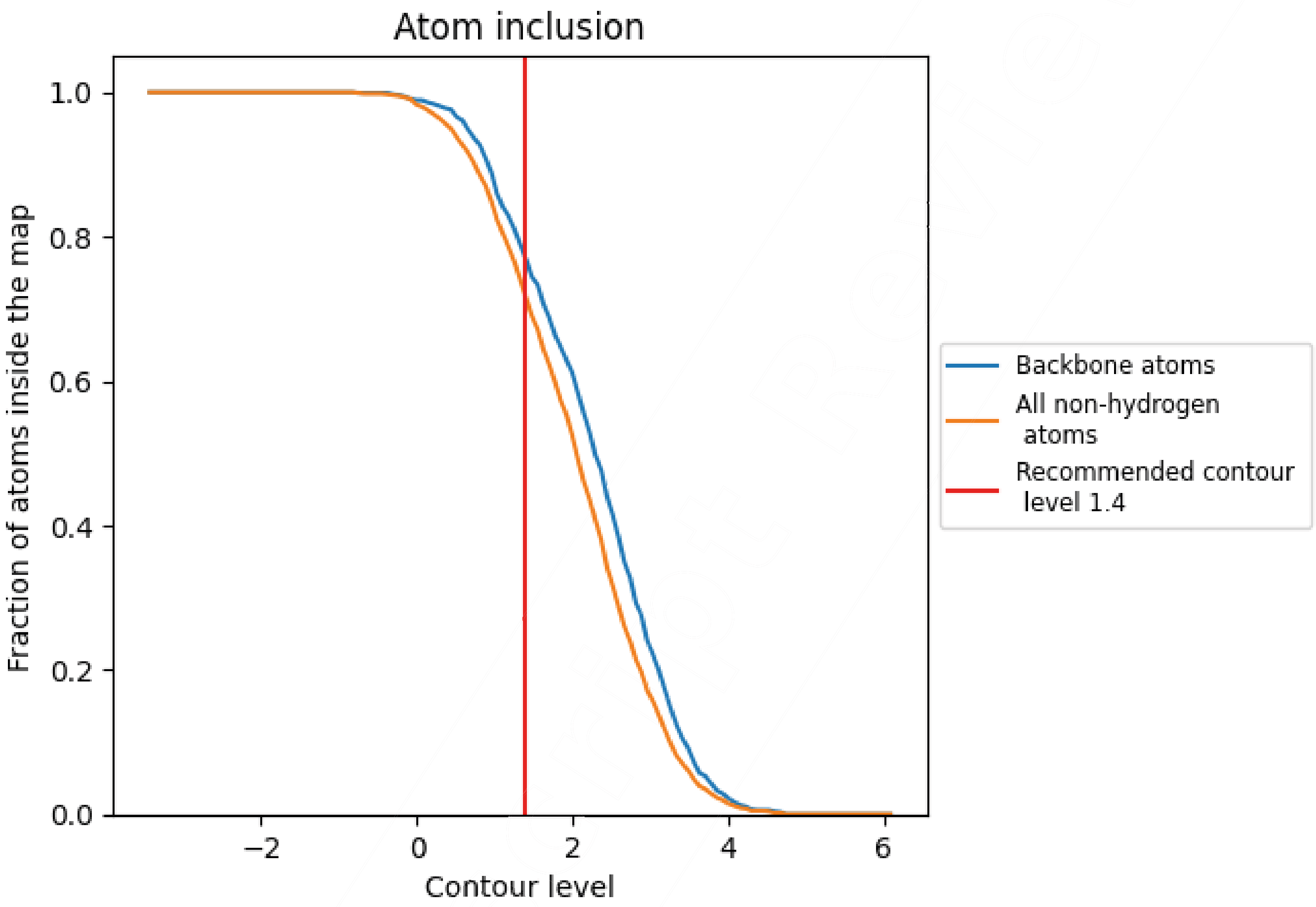

At the recommended contour level, 77% of all backbone atoms, 72% of all non-hydrogen atoms, are inside the map.

For Manuscript Review

9.5 Map-model fit summary (i)

The table lists the average atom inclusion at the recommended contour level (1.4) and Q-score for the entire model and for each chain.

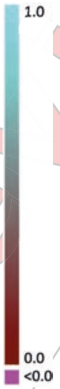

Chain	Atom inclusion	Q-score
All	0.7170	0.5360
0	0.7170	0.5360

For Manuscript Review

Preliminary Full wwPDB EM Validation Report (i)

Feb 5, 2025 – 08:35 am GMT

Deposition ID : D_1292144938

This wwPDB validation report is NOT for manuscript review

This is a Preliminary Full wwPDB EM Validation Report.

This report is produced by the wwPDB Deposition System during initial deposition but before annotation of the structure.

We welcome your comments at *validation@mail.wwpdb.org*
A user guide is available at
https://www.wwpdb.org/validation/2017/EMValidationReportHelp
with specific help available everywhere you see the (i) symbol.

The types of validation reports are described at
http://www.wwpdb.org/validation/2017/FAQs#types.

Not For Manuscript Review

The following versions of software and data (see references (i)) were used in the production of this report:

EMDB validation analysis	:	0.0.1.dev113
MolProbity	:	4.02b-467
Percentile statistics	:	20231227.v01 (using entries in the PDB archive December 27th 2023)
MapQ	:	1.9.13
Ideal geometry (proteins)	:	Engh & Huber (2001)
Ideal geometry (DNA, RNA)	:	Parkinson et al. (1996)
Validation Pipeline (wwPDB-VP)	:	2.40

1 Overall quality at a glance (i)

The following experimental techniques were used to determine the structure:
ELECTRON MICROSCOPY

The reported resolution of this entry is 3.00 Å.

Percentile scores (ranging between 0-100) for global validation metrics of the entry are shown in the following graphic. The table shows the number of entries on which the scores are based.

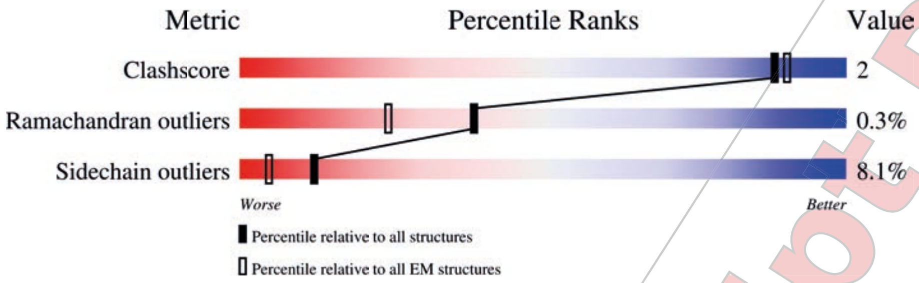

Metric	Whole archive (#Entries)	EM structures (#Entries)
Clashscore	210492	15764
Ramachandran outliers	207382	16835
Sidechain outliers	206894	16415

The table below summarises the geometric issues observed across the polymeric chains and their fit to the map. The red, orange, yellow and green segments of the bar indicate the fraction of residues that contain outliers for >=3, 2, 1 and 0 types of geometric quality criteria respectively. A grey segment represents the fraction of residues that are not modelled. The numeric value for each fraction is indicated below the corresponding segment, with a dot representing fractions <=5% The upper red bar (where present) indicates the fraction of residues that have poor fit to the EM map (all-atom inclusion < 40%). The numeric value is given above the bar.

Mol	Chain	Length	Quality of chain
1	A	340	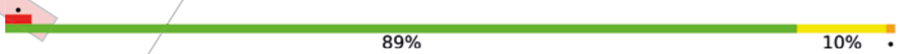
2	B	339	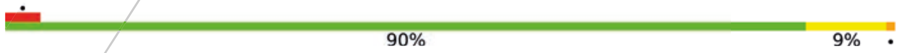

Not For Manuscript Review

2 Entry composition (i)

There are 2 unique types of molecules in this entry. The entry contains 4958 atoms, of which 0 are hydrogens and 0 are deuteriums.

In the tables below, the AltConf column contains the number of residues with at least one atom in alternate conformation and the Trace column contains the number of residues modelled with at most 2 atoms.

- Molecule 1 is a protein.

Mol	Chain	Residues	Atoms					AltConf	Trace
			Total	C	N	O	S		
1	A	340	2481	1570	423	482	6	0	0

- Molecule 2 is a protein.

Mol	Chain	Residues	Atoms					AltConf	Trace
			Total	C	N	O	S		
2	B	339	2477	1568	422	481	6	0	0

Not For Manuscript Review

3 Residue-property plots ⓘ

These plots are drawn for all protein, RNA, DNA and oligosaccharide chains in the entry. The first graphic for a chain summarises the proportions of the various outlier classes displayed in the second graphic. The second graphic shows the sequence view annotated by issues in geometry and atom inclusion in map density. Residues are color-coded according to the number of geometric quality criteria for which they contain at least one outlier: green = 0, yellow = 1, orange = 2 and red = 3 or more. A red diamond above a residue indicates a poor fit to the EM map for this residue (all-atom inclusion < 40%). Stretches of 2 or more consecutive residues without any outlier are shown as a green connector. Residues present in the sample, but not in the model, are shown in grey.

- Molecule 1:

Chain A: 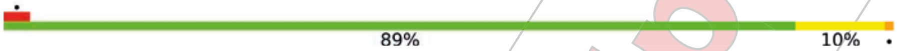

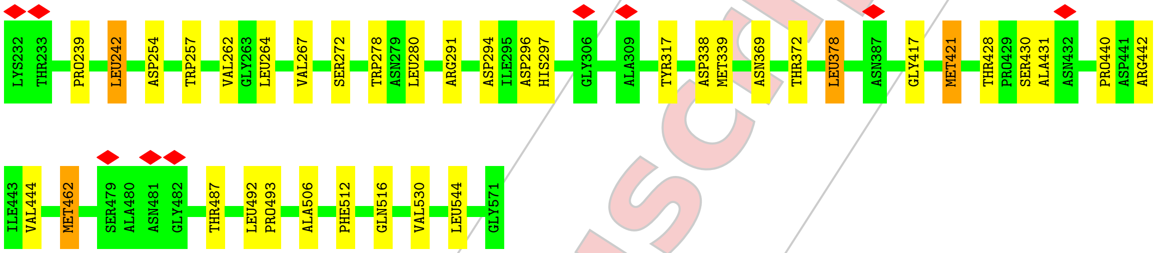

- Molecule 2:

Chain B: 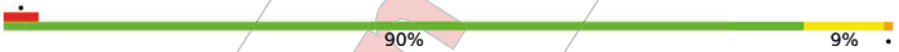

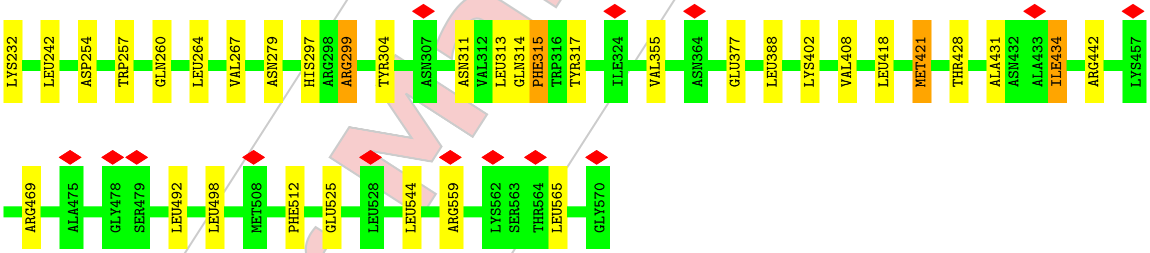

Not For Manuscript Review

4 Experimental information (i)

Property	Value	Source
EM reconstruction method	SINGLE PARTICLE	Depositor
Imposed symmetry	POINT, Not provided	
Number of particles used	Not provided	
Resolution determination method	Not provided	
CTF correction method	Not provided	
Microscope	Not provided	
Voltage (kV)	Not provided	
Electron dose $(e^-/\text{Å}^2)$	Not provided	
Minimum defocus (nm)	Not provided	
Maximum defocus (nm)	Not provided	
Magnification	Not provided	
Image detector	Not provided	
Maximum map value	0.629	Depositor
Minimum map value	-0.032	Depositor
Average map value	0.005	Depositor
Map value standard deviation	0.029	Depositor
Recommended contour level	0.014	Depositor
Map size (Å)	74.520004, 75.600006, 83.16	wwPDB
Map dimensions	69, 70, 77	wwPDB
Map angles (°)	90.0, 90.0, 90.0	wwPDB
Pixel spacing (Å)	1.08, 1.08, 1.08	Depositor

Not For Manuscript Review

5 Model quality (i)

5.1 Standard geometry (i)

The Z score for a bond length (or angle) is the number of standard deviations the observed value is removed from the expected value. A bond length (or angle) with $|Z| > 5$ is considered an outlier worth inspection. RMSZ is the root-mean-square of all Z scores of the bond lengths (or angles).

Mol	Chain	Bond lengths		Bond angles	
		RMSZ	#$\|Z\|$ >5	RMSZ	#$\|Z\|$ >5
1	A	0.34	0/2551	0.67	3/3505 (0.1%)
2	B	0.35	0/2547	0.66	3/3500 (0.1%)
All	All	0.34	0/5098	0.66	6/7005 (0.1%)

There are no bond length outliers.

All (6) bond angle outliers are listed below:

Mol	Chain	Res	Type	Atoms	Z	Observed(^{o})	Ideal(^{o})
1	A	296	ASP	CB-CG-OD1	6.50	124.15	118.30
2	B	498	LEU	CA-CB-CG	5.99	129.08	115.30
2	B	544	LEU	CA-CB-CG	5.61	128.20	115.30
1	A	378	LEU	CA-CB-CG	5.53	128.03	115.30
1	A	242	LEU	CA-CB-CG	5.42	127.78	115.30
2	B	418	LEU	CA-CB-CG	5.01	126.82	115.30

There are no chirality outliers.

There are no planarity outliers.

5.2 Too-close contacts (i)

In the following table, the Non-H and H(model) columns list the number of non-hydrogen atoms and hydrogen atoms in the chain respectively. The H(added) column lists the number of hydrogen atoms added and optimized by MolProbity. The Clashes column lists the number of clashes within the asymmetric unit, whereas Symm-Clashes lists symmetry-related clashes.

Mol	Chain	Non-H	H(model)	H(added)	Clashes	Symm-Clashes
1	A	2481	0	2393	8	0
2	B	2477	0	2390	9	0
All	All	4958	0	4783	17	0

The all-atom clashscore is defined as the number of clashes found per 1000 atoms (including

Not For Manuscript Review

hydrogen atoms). The all-atom clashscore for this structure is 2.

All (17) close contacts within the same asymmetric unit are listed below, sorted by their clash magnitude.

Atom-1	Atom-2	Interatomic distance (Å)	Clash overlap (Å)
1:A:280:LEU:HD22	1:A:530:VAL:HA	1.83	0.59
2:B:355:VAL:HG12	2:B:377:GLU:HB2	1.86	0.58
2:B:428:THR:HG21	2:B:434:ILE:HD13	1.87	0.56
1:A:239:PRO:HD3	1:A:506:ALA:HB1	1.89	0.54
1:A:267:VAL:HG21	1:A:440:PRO:HG3	1.90	0.53
2:B:311:ASN:H	2:B:408:VAL:HA	1.74	0.53
1:A:338:ASP:HB2	1:A:444:VAL:HG22	1.91	0.52
1:A:297:HIS:O	1:A:417:GLY:HA3	2.11	0.50
2:B:431:ALA:HA	2:B:434:ILE:HD11	1.97	0.46
2:B:315:PHE:HZ	2:B:388:LEU:HD11	1.82	0.45
1:A:462:MET:HG2	1:A:487:THR:HB	1.97	0.45
1:A:421:MET:N	1:A:421:MET:SD	2.90	0.44
2:B:559:ARG:HD3	2:B:559:ARG:HA	1.82	0.43
1:A:492:LEU:N	1:A:493:PRO:HD2	2.33	0.43
2:B:421:MET:N	2:B:421:MET:SD	2.91	0.42
2:B:525:GLU:HB2	2:B:565:LEU:HB3	2.02	0.41
2:B:299:ARG:H	2:B:299:ARG:HG2	1.76	0.40

There are no symmetry-related clashes.

5.3 Torsion angles (i)

5.3.1 Protein backbone (i)

In the following table, the Percentiles column shows the percent Ramachandran outliers of the chain as a percentile score with respect to all PDB entries followed by that with respect to all EM entries.

The Analysed column shows the number of residues for which the backbone conformation was analysed, and the total number of residues.

Mol	Chain	Analysed	Favoured	Allowed	Outliers	Percentiles	
1	A	338/340 (99%)	302 (89%)	35 (10%)	1 (0%)	37	70
2	B	337/339 (99%)	308 (91%)	28 (8%)	1 (0%)	37	70
All	All	675/679 (99%)	610 (90%)	63 (9%)	2 (0%)	38	70

All (2) Ramachandran outliers are listed below:

Not For Manuscript Review

Mol	Chain	Res	Type
1	A	431	ALA
2	B	434	ILE

5.3.2 Protein sidechains (i)

In the following table, the Percentiles column shows the percent sidechain outliers of the chain as a percentile score with respect to all PDB entries followed by that with respect to all EM entries.

The Analysed column shows the number of residues for which the sidechain conformation was analysed, and the total number of residues.

Mol	Chain	Analysed	Rotameric	Outliers	Percentiles	
1	A	264/264 (100%)	242 (92%)	22 (8%)	9	34
2	B	264/264 (100%)	243 (92%)	21 (8%)	10	35
All	All	528/528 (100%)	485 (92%)	43 (8%)	12	34

All (43) residues with a non-rotameric sidechain are listed below:

Mol	Chain	Res	Type
1	A	242	LEU
1	A	254	ASP
1	A	257	TRP
1	A	262	VAL
1	A	264	LEU
1	A	272	SER
1	A	278	TRP
1	A	291	ARG
1	A	294	ASP
1	A	317	TYR
1	A	339	MET
1	A	369	ASN
1	A	372	THR
1	A	378	LEU
1	A	421	MET
1	A	428	THR
1	A	430	SER
1	A	442	ARG
1	A	462	MET
1	A	512	PHE
1	A	516	GLN
1	A	544	LEU

Continued on next page...

Not For Manuscript Review

Continued from previous page...

Mol	Chain	Res	Type
2	B	232	LYS
2	B	242	LEU
2	B	254	ASP
2	B	257	TRP
2	B	260	GLN
2	B	264	LEU
2	B	267	VAL
2	B	279	ASN
2	B	297	HIS
2	B	299	ARG
2	B	304	TYR
2	B	313	LEU
2	B	314	GLN
2	B	315	PHE
2	B	317	TYR
2	B	402	LYS
2	B	421	MET
2	B	442	ARG
2	B	469	ARG
2	B	492	LEU
2	B	512	PHE

Sometimes sidechains can be flipped to improve hydrogen bonding and reduce clashes. There are no such sidechains identified.

5.3.3 RNA (i)

There are no RNA molecules in this entry.

5.4 Non-standard residues in protein, DNA, RNA chains (i)

There are no non-standard protein/DNA/RNA residues in this entry.

5.5 Carbohydrates (i)

There are no oligosaccharides in this entry.

5.6 Ligand geometry (i)

There are no ligands in this entry.

Not For Manuscript Review

5.7 Other polymers (i)

There are no such residues in this entry.

5.8 Polymer linkage issues (i)

There are no chain breaks in this entry.

Not For Manuscript Review

6 Map visualisation (i)

This section contains visualisations of the EMDB entry D_1292144938. These allow visual inspection of the internal detail of the map and identification of artifacts.

Images derived from a raw map, generated by summing the deposited half-maps, are presented below the corresponding image components of the primary map to allow further visual inspection and comparison with those of the primary map.

6.1 Orthogonal projections (i)

6.1.1 Primary map

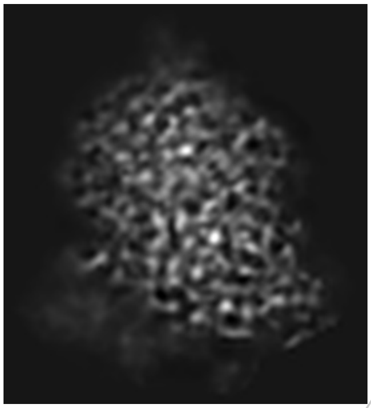
X

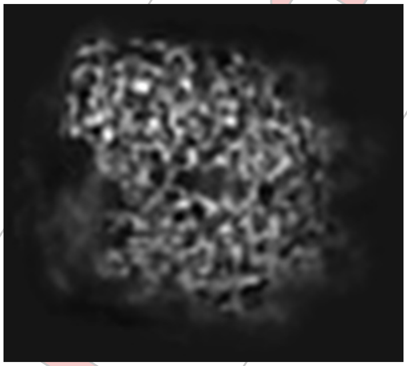
Y

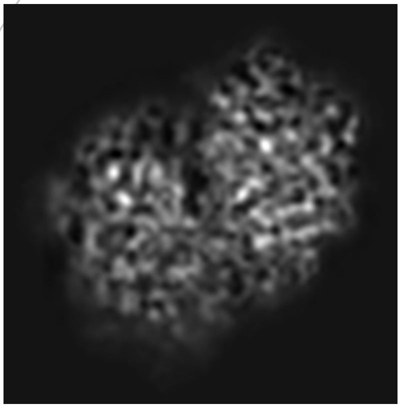
Z

6.1.2 Raw map

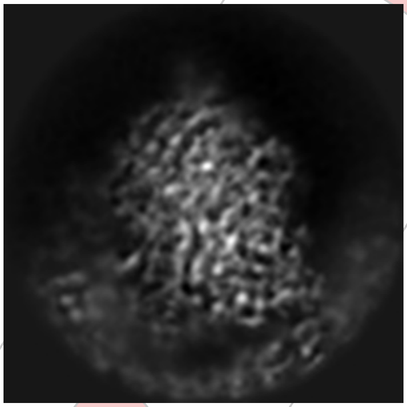
X

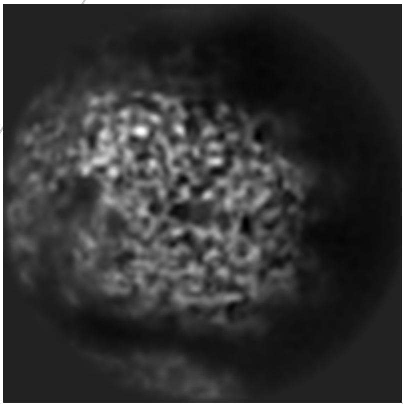
Y

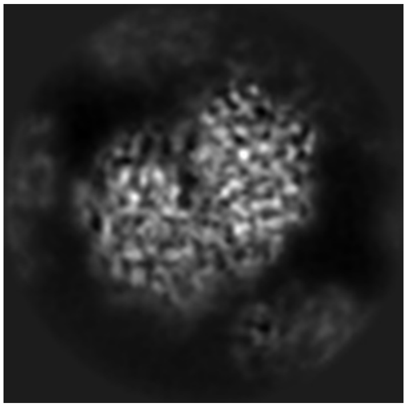
Z

The images above show the map projected in three orthogonal directions.

Not For Manuscript Review

6.2 Central slices (i)

6.2.1 Primary map

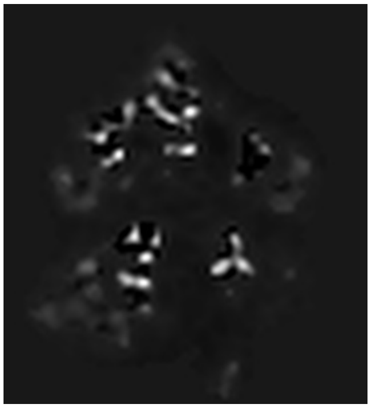
X Index: 34

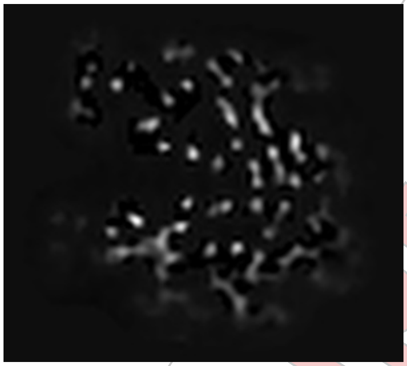
Y Index: 35

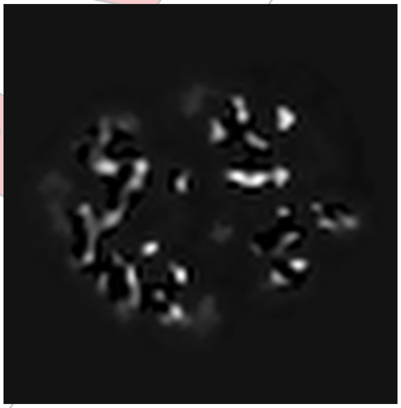
Z Index: 38

6.2.2 Raw map

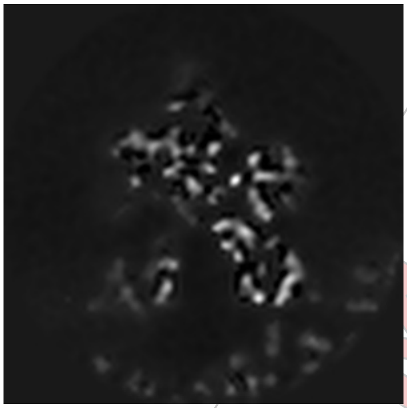
X Index: 45

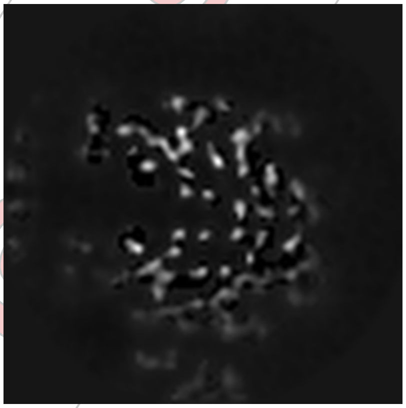
Y Index: 45

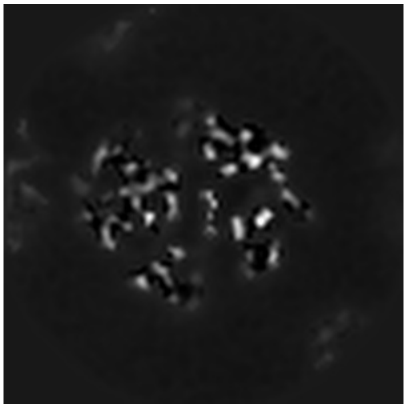
Z Index: 45

The images above show central slices of the map in three orthogonal directions.

Not For Manuscript Review

6.3 Largest variance slices (i)

6.3.1 Primary map

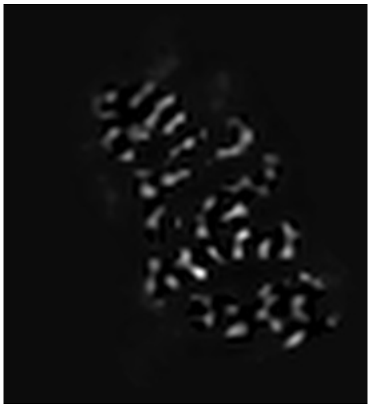
X Index: 45

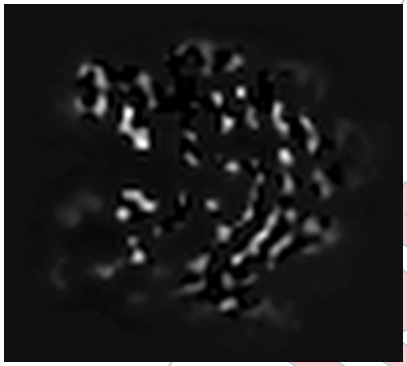
Y Index: 32

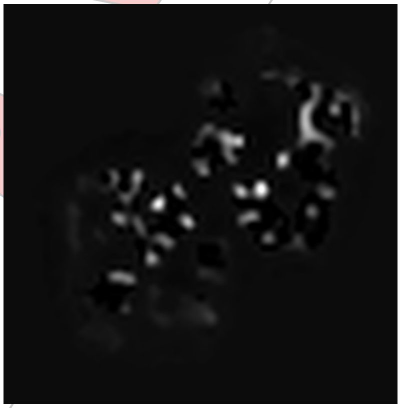
Z Index: 25

6.3.2 Raw map

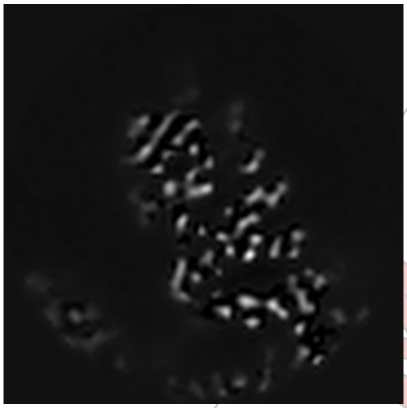
X Index: 54

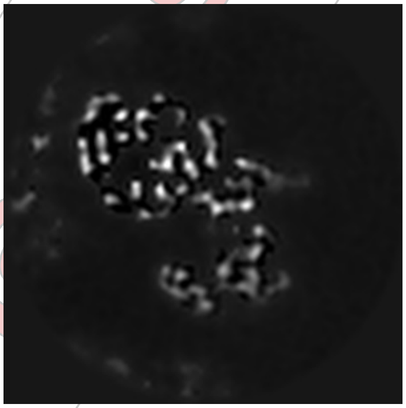
Y Index: 56

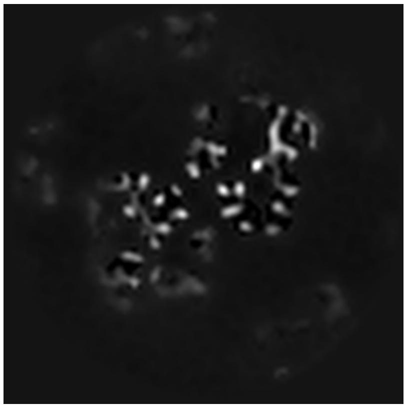
Z Index: 30

The images above show the largest variance slices of the map in three orthogonal directions.

Not For Manuscript Review

6.4 Orthogonal standard-deviation projections (False-color) (i)

6.4.1 Primary map

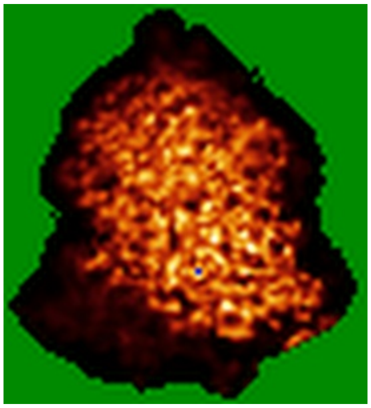
X

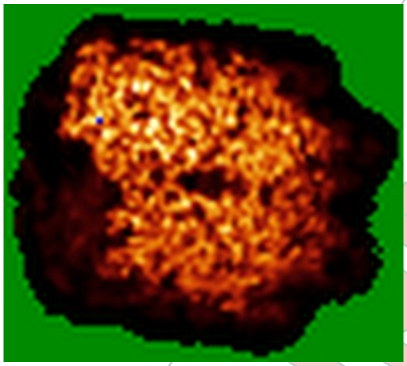
Y

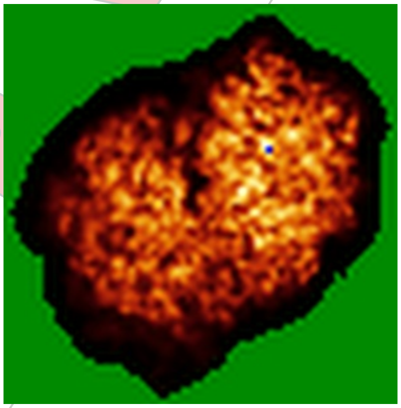
Z

6.4.2 Raw map

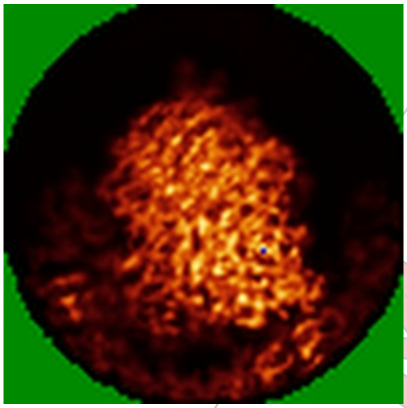
X

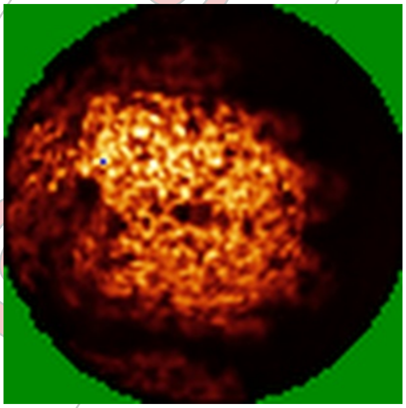
Y

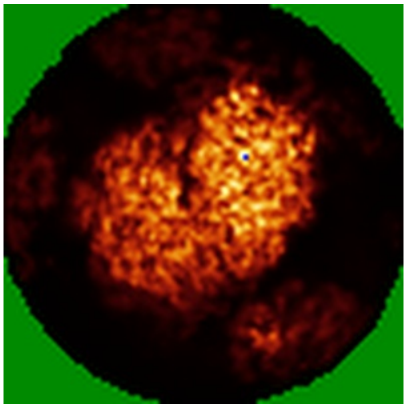
Z

The images above show the map standard deviation projections with false color in three orthogonal directions. Minimum values are shown in green, max in blue, and dark to light orange shades represent small to large values respectively.

Not For Manuscript Review

6.5 Orthogonal surface views (i)

6.5.1 Primary map

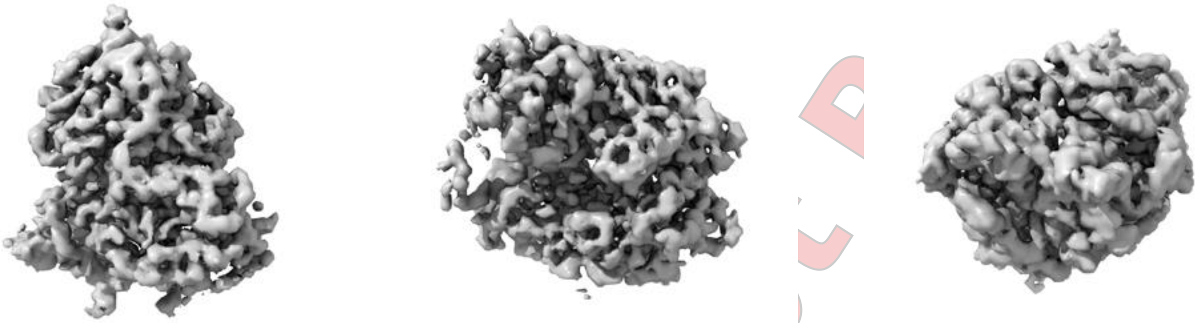

X Y Z

The images above show the 3D surface view of the map at the recommended contour level 0.014. These images, in conjunction with the slice images, may facilitate assessment of whether an appropriate contour level has been provided.

6.5.2 Raw map

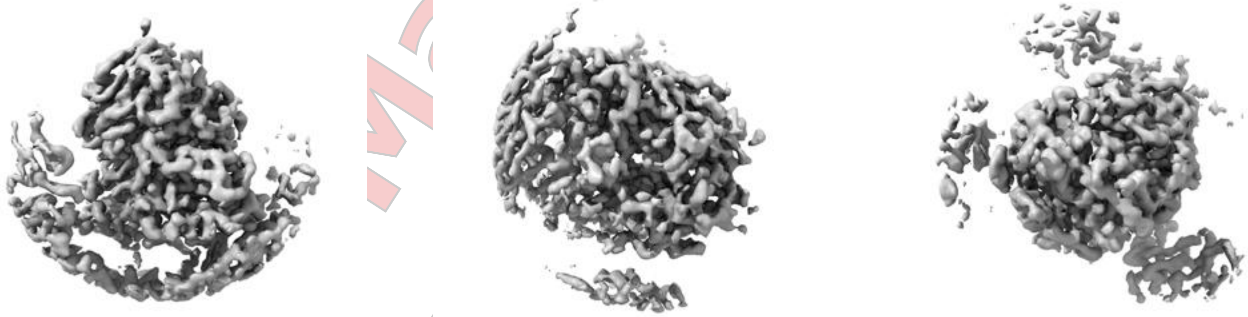

X Y Z

These images show the 3D surface of the raw map. The raw map's contour level was selected so that its surface encloses the same volume as the primary map does at its recommended contour level.

6.6 Mask visualisation (i)

This section was not generated. No masks/segmentation were deposited.

Not For Manuscript Review

7 Map analysis (i)

This section contains the results of statistical analysis of the map.

7.1 Map-value distribution (i)

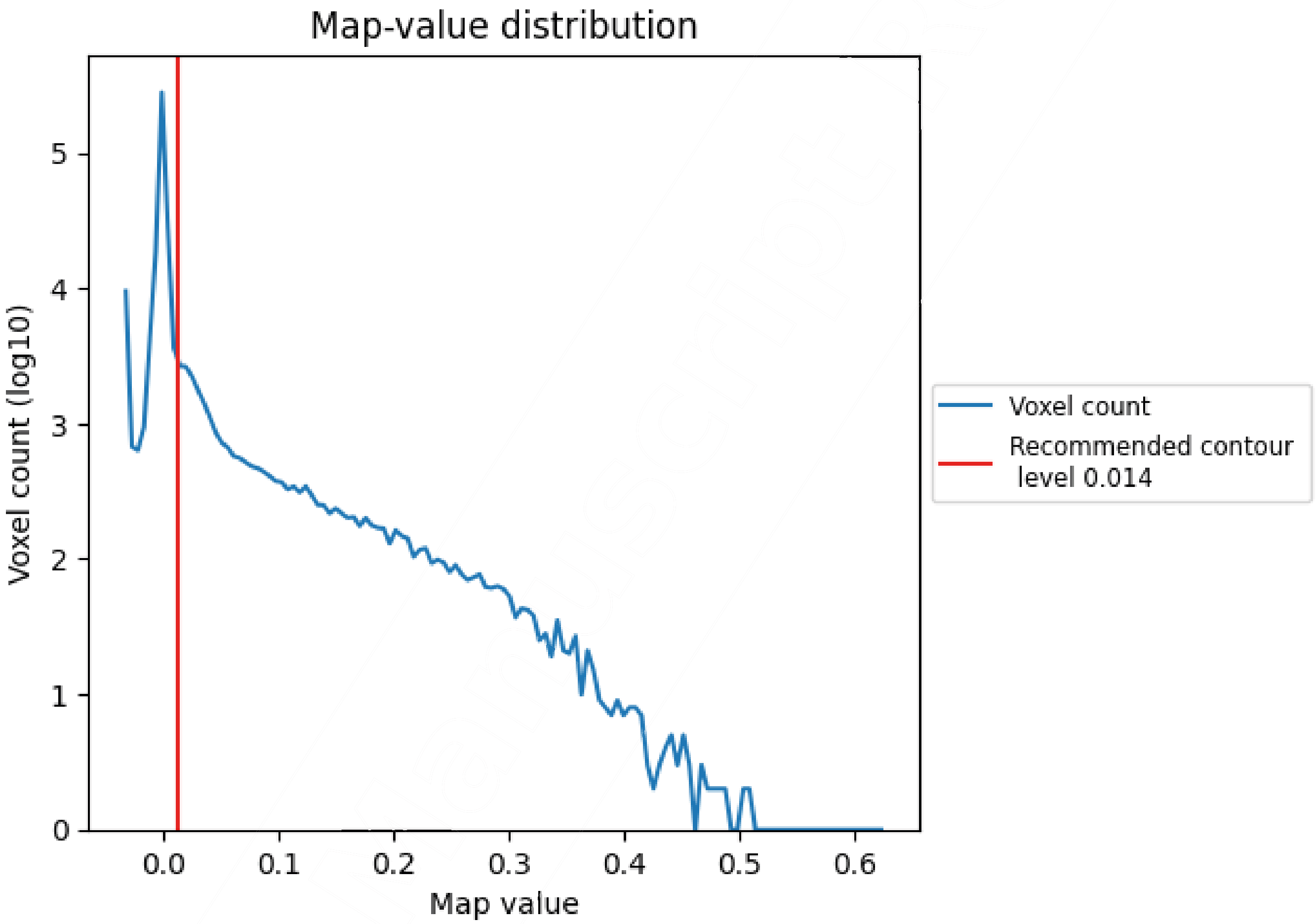

The map-value distribution is plotted in 128 intervals along the x-axis. The y-axis is logarithmic. A spike in this graph at zero usually indicates that the volume has been masked.

Not For Manuscript Review

7.2 Volume estimate (i)

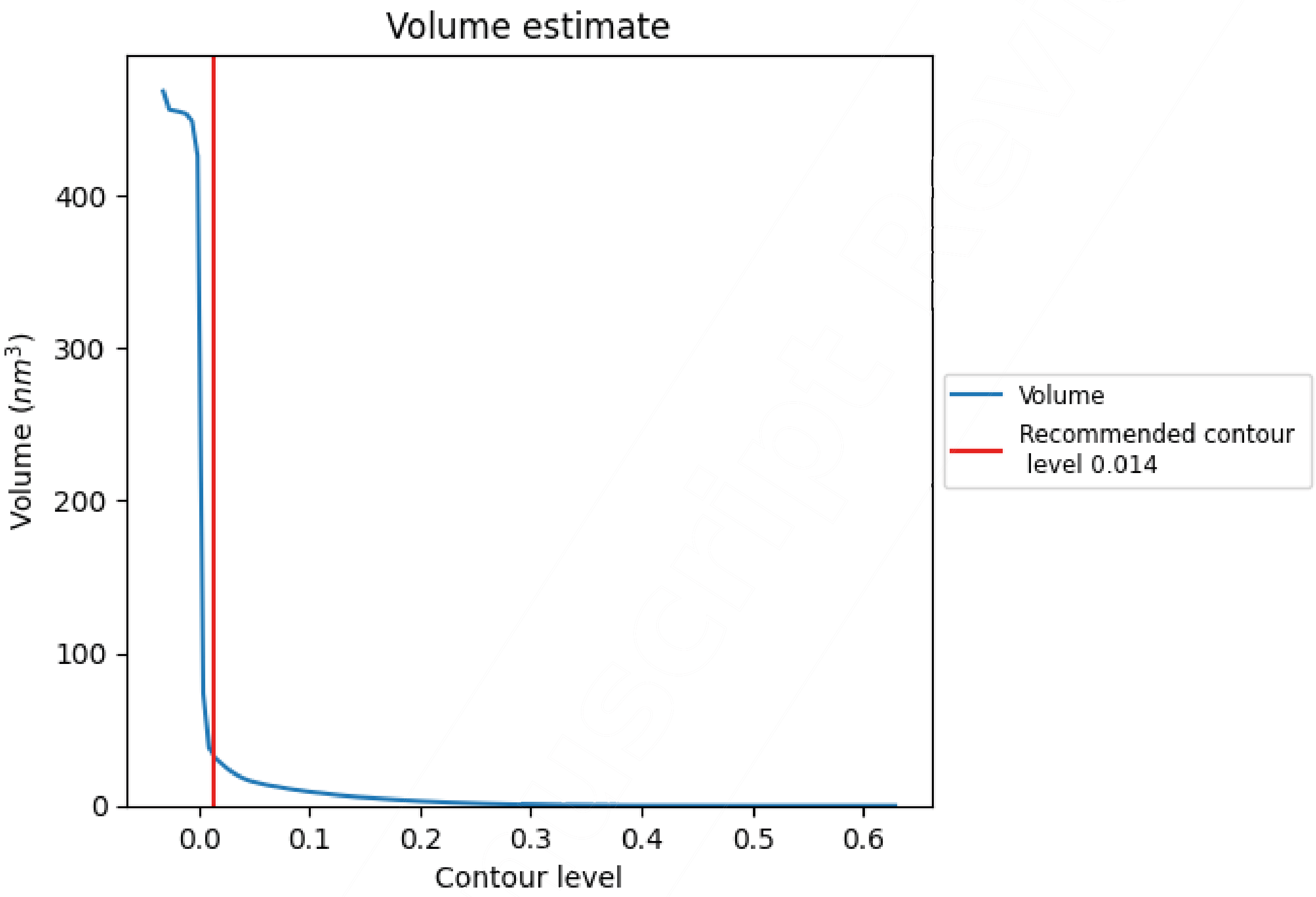

The volume at the recommended contour level is 32 nm^3; this corresponds to an approximate mass of 29 kDa.

The volume estimate graph shows how the enclosed volume varies with the contour level. The recommended contour level is shown as a vertical line and the intersection between the line and the curve gives the volume of the enclosed surface at the given level.

7.3 Rotationally averaged power spectrum (i)

This section was not generated. The rotationally averaged power spectrum is only generated for cubic maps.

Not For Manuscript Review

8 Fourier-Shell correlation (i)

Fourier-Shell Correlation (FSC) is the most commonly used method to estimate the resolution of single-particle and subtomogram-averaged maps. The shape of the curve depends on the imposed symmetry, mask and whether or not the two 3D reconstructions used were processed from a common reference. The reported resolution is shown as a black line. A curve is displayed for the half-bit criterion in addition to lines showing the 0.143 gold standard cut-off and 0.5 cut-off.

8.1 FSC (i)

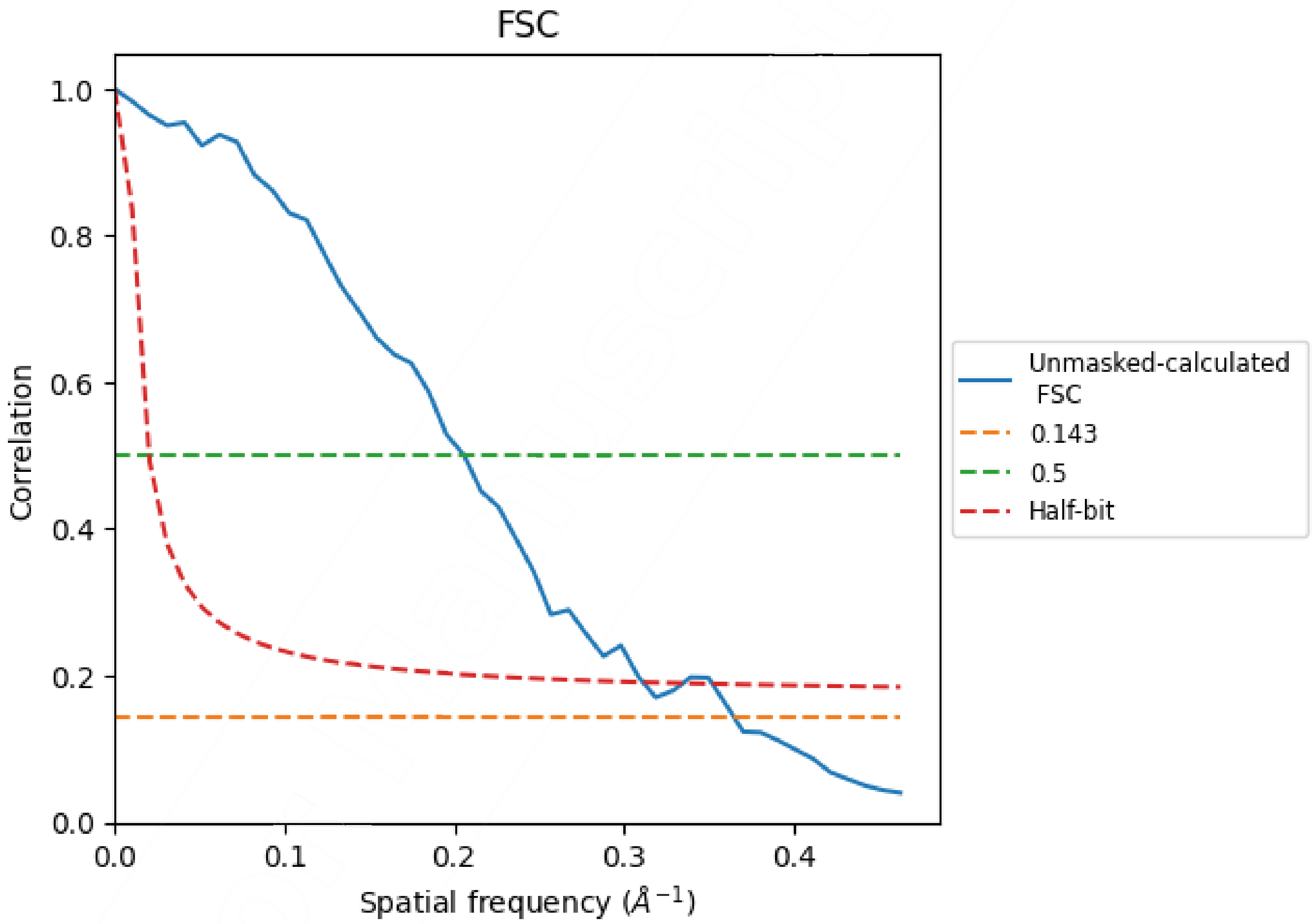

Not For Manuscript Review

8.2 Resolution estimates (i)

Resolution estimate (Å)	Estimation criterion (FSC cut-off)		
	0.143	0.5	Half-bit
Reported by author	-	-	-
Author-provided FSC curve	-	-	-
Unmasked-calculated*	2.74	4.85	3.21

*Resolution estimate based on FSC curve calculated by comparison of deposited half-maps.

Not For Manuscript Review

9 Map-model fit (i)

This section contains information regarding the fit between EMDB map D_1292144938 and PDB model D_1292144938. Per-residue inclusion information can be found in section 3 on page 4.

9.1 Map-model overlay (i)

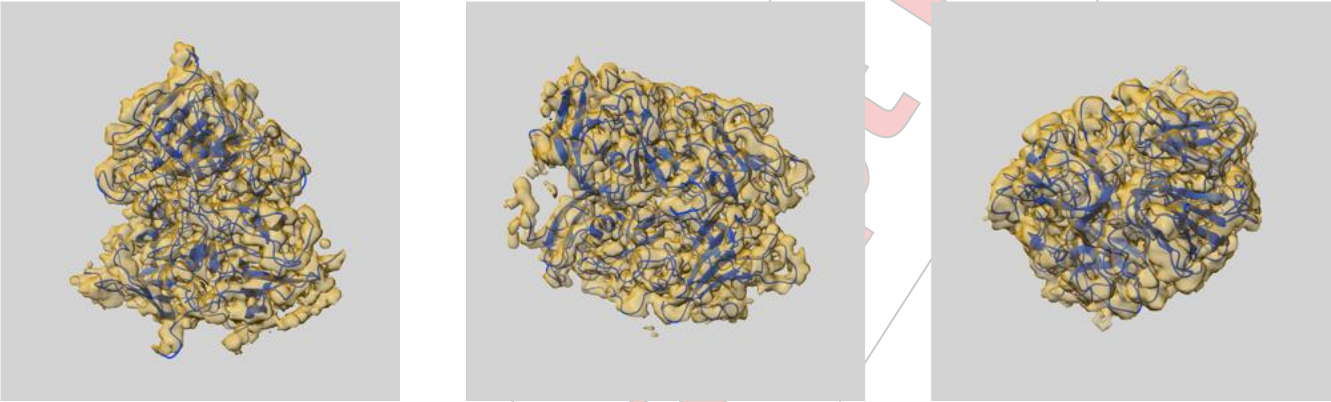

X Y Z

The images above show the 3D surface view of the map at the recommended contour level 0.014 at 50% transparency in yellow overlaid with a ribbon representation of the model coloured in blue. These images allow for the visual assessment of the quality of fit between the atomic model and the map.

Not For Manuscript Review

9.2 Q-score mapped to coordinate model (i)

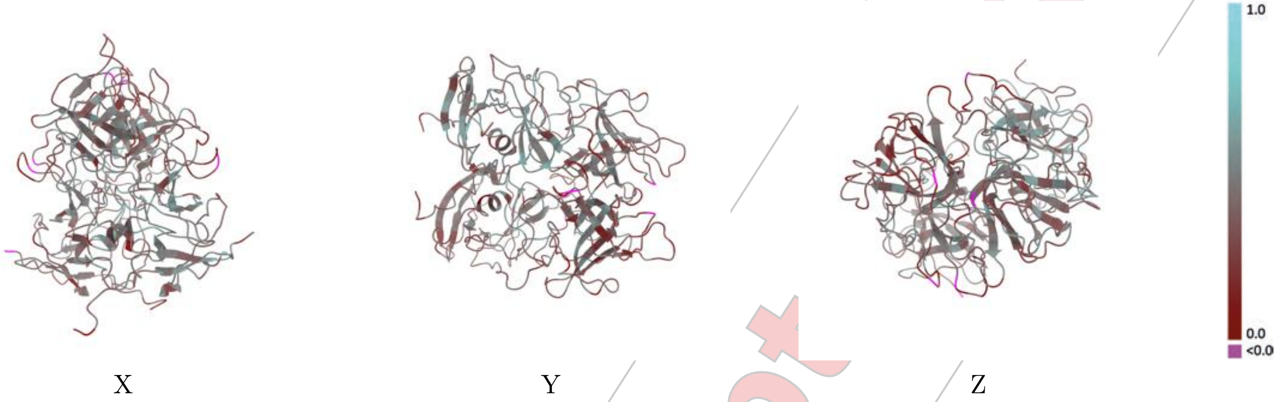

The images above show the model with each residue coloured according its Q-score. This shows their resolvability in the map with higher Q-score values reflecting better resolvability. Please note: Q-score is calculating the resolvability of atoms, and thus high values are only expected at resolutions at which atoms can be resolved. Low Q-score values may therefore be expected for many entries.

9.3 Atom inclusion mapped to coordinate model (i)

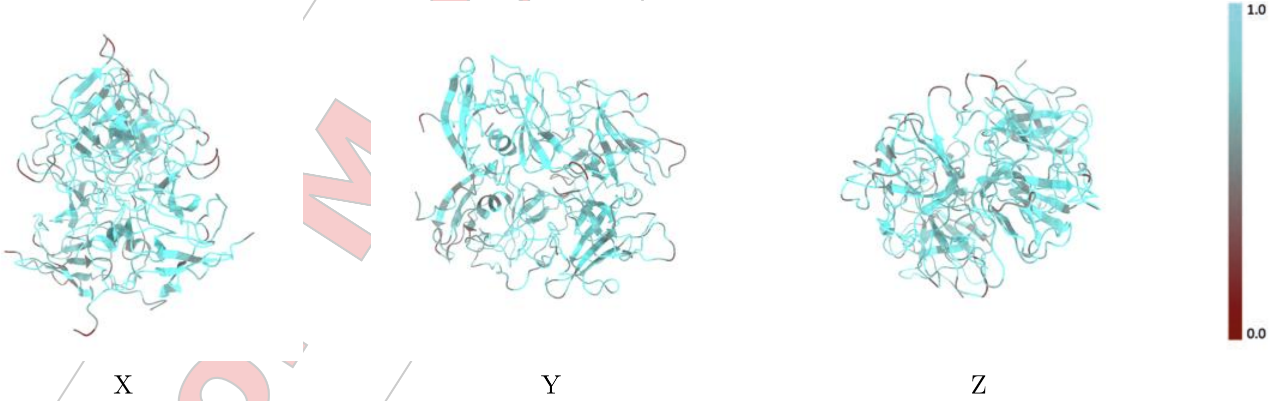

The images above show the model with each residue coloured according to its atom inclusion. This shows to what extent they are inside the map at the recommended contour level (0.014).

Not For Manuscript Review

9.4 Atom inclusion (i)

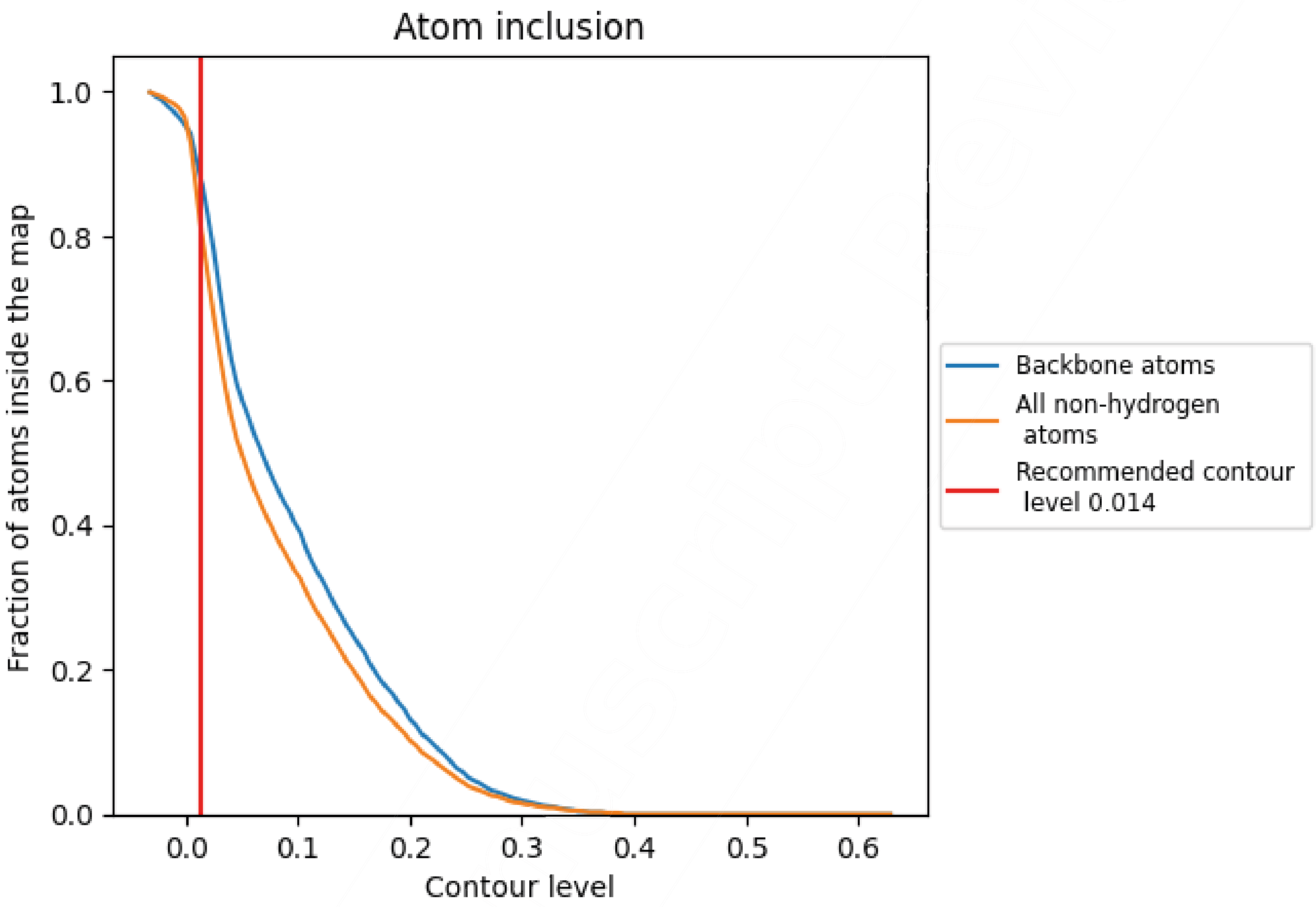

At the recommended contour level, 87% of all backbone atoms, 80% of all non-hydrogen atoms, are inside the map.

Not For Manuscript Review

9.5 Map-model fit summary (i)

The table lists the average atom inclusion at the recommended contour level (0.014) and Q-score for the entire model and for each chain.

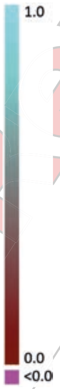

Chain	Atom inclusion	Q-score
All	0.8040	0.3910
A	0.8310	0.4240
B	0.7770	0.3580

Not For Manuscript Review

9.5 Map-model fit summary (i)

The table lists the average atom inclusion at the recommended contour level (0.55) and Q-score for the entire model and for each chain.

Chain	Atom inclusion	Q-score
All	0.7470	0.3740
A	0.6890	0.3190
B	0.6600	0.3050
C	0.8910	0.4970

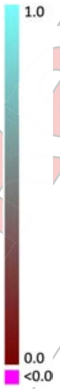

Not For Manuscript Review
