# Supplementary material for: Calicivirus assembly and stability are mediated by the N-terminal domain of the capsid protein with the involvement of the viral genome
Source: PLoS Pathog. 2025 Dec 11;21(12):e1013364. doi: 10.1371/journal.ppat.1013364 (PMC12707631; doi:10.1371/journal.ppat.1013364)
Supplement: Attachment — Submitted filename: Response to Reviewers.docx [file ppat.1013364.s019.docx]

Manuscript: PPATHOGENS-D-25-01689

Point-by-point reply to Reviewer #1

***Part I - Summary***

*The authors studied RHDV virions and several virus-like particle structures at near atomic resolutions using cryo-EM. The reconstructions achieve much higher resolutions than previously published studies, thereby allowing detailed investigation of specific amino acid residue interactions within the protein building blocks of the virus. The study revealed that the critical molecular components (the NTA network and the viral RNA genome) determine structural polymorphisms of VP1 dimers in an icosahedral capsid. The molecular interactions are compared with those of other caliciviruses, highlighting shared features and differences. The authors also used AFM to study the mechanical stability of both T=3 empty and full particles, as well as the T=1 empty particles. The experiments suggested that RNA not only facilitates particle assembly but also contributes to its stability under mechanical stress. Overall, this is a well-written paper that provides a comprehensive structural analysis of the virus structure, representing a significant advancement in the structural biology of caliciviruses*

***Part II – Major Issues: Key Experiments Required for Acceptance***

*3DFlex analysis shows that the protrusion spikes swing relative to the shell base. This is a very interesting observation that also raises many questions. Some of the following questions could be addressed in the discussion section or explored further if computationally feasible.*

*What is the nature of the motion? Is it directional relative to the base for different dimers (C/C and A/B)? The VP1 dimer contains two short hinge regions (residues 230-237) in the VP1 dimer, which should limit the swing direction by structural configuration. Is the direction of swing perpendicular to the orientation of these hinges in the dimer? Is there also a twist or swelling relative to the local 2-fold axis? Could there be a local shift of the whole dimer? Could the motion observed in 3DFlex represent a combination of all these movements? Furthermore, if particle size varies, would curvature differences also contribute to apparent motion in 3DFlex?*

We appreciate this point and agree we did not sufficiently address the motion of the dimeric spikes that indicates the capsid is a dynamic structure. The movies calculated by 3DFlex analysis are based on 20 or 40 3D maps (a default value) whose resolution is low for a detailed description of this movement. In addition, as stated in the main text, spike swing angles are relatively small in the T=3 capsid compared with the T=1 capsid. We have now reanalyzed the dimers of the T=1 Δ29N capsid, which were extracted as independent subparticles and subsequently subjected to 3D classification without angular assignment to describe the swing angle of the spikes. The resulting 20 classes, characterized with different swing angles (Fig. 1, below), were grouped into five major classes (Fig. 2, below) encompassing 100% of the particles. In these five classes, in addition to the initial state of the dimer (equivalent to that resulting after imposing icosahedral symmetry in the entire particle), two states showed the spikes with the highest swing angles in opposite directions, exactly as shown by the 3Dflex data, and the other two states showed intermediate swing angles. We next fitted the atomic coordinates of a VP1 dimer to determine that the direction of swing is indeed perpendicular to the hinge orientation in the dimer. These results suggest a unique, allowed movement for the spikes, in which both P domains swing together.

**Fig. 1.** Movement of the dimeric spikes of the T=1 capsid. 3D classification without angular assignment of Δ29N dimeric spikes into 20 classes (1–20, upper left) showing the relative abundance of each class (upper right). The density maps of the different classes (gray) are superimposed with a spike extracted from the Δ29N capsid reconstruction calculated with imposed icosahedral symmetry (blue transparent surface).

**Fig. 2.** Protrusion spikes swing relative to the shell base. (A) The analysis of Δ29N dimeric spikes yields five major classes that comprise 100% of particles. Class I (∼10.1%) includes classes 4 and 7, represented by class 4; class II (∼25.6%) includes classes 10, 12, 15, 16 and 18, represented by class 12; class III (∼35.1%) includes classes 2, 3, 5, 6, 13, 17 and 19, represented by class 2; Class IV (∼19.5%) includes classes 1, 11, 14, and 20, represented by class 1; and class V (∼9.7%) includes classes 8 and 9, represented by class 9. The gray density maps of the different classes are superimposed with the icosahedral spike extracted from the Δ29N capsid (blue transparent surface). Classes I and V display the spikes with the largest swing angles in opposite directions. (B) Superposition of VP1 dimers fitted in classes I (gray) and V (red) shows a swing angle of ~27º perpendicular to the hinge orientation.

Figure 2 is now included in the new manuscript version as a new supplementary figure, S2 Fig. In addition, an added new paragraph reads (l. 166–170): “Analysis with 3DFlex [41] of T=1 Δ29N capsids and by 3D classification of Δ29N extracted dimers allowed us to determine that the protrusion swinging angle ranges between ~10° and ~27° (S1 Video, S2A Fig). In the T=1 capsid, the direction of swing is perpendicular to the orientation of the hinge regions within the dimer (S2B Fig). These results suggest a unique, allowed movement of the spikes”.

In both the T=1 and T=3 capsids, spike movement is possible because the P domain is not tightly attached to the shell via the hinge region, *i.e.*, the P domain “floats” above the shell domain. Nevertheless, in some caliciviruses (including RHDV GI.2), the P domain might also exist in a conformation resting upon the shell domain. These two states, “up” and “down” of the protruding spikes, are due to changes in the interactions between the S and P domain residues (ref. 15). We have added a new supplementary figure in the revised manuscript (S3 Fig, also requested by the journal) that compares the A/B dimer in virions and VLPs of RHDV GI.1, in which we observed no differences. In addition, a paragraph is added to clarify the flexibility of the protrusion spikes (l. 218–221): “Although ‘up’ and ‘down’ conformations of the protruding domain have been reported for RHDV GI.2 [15] and human norovirus [19], we observed no conformational differences of the protruding domain between the mature virion and the VLP after alignment of the A–B dimers (S3 Fig)”.

Following the reviewer’s suggestion, we have also expanded the discussion of this point to include the following paragraph (l. 448-452): “3DFlex and subparticle analyses of T=1 capsids indicate that the protruding spikes undergo a coordinated swinging motion of the VP1 dimers, restricted by the geometry of the hinge regions and occurring perpendicular to their orientation. This collective flexibility likely represents an intrinsic property of calicivirus capsids that balances structural stability with conformational adaptability”.

*3DFLex analysis shows that the T=3 capsid (empty) exhibits reduced protrusion swing angles comparing to the T=1 Δ29N particle (~5 degrees vs. 10-15 degrees). Is this reduction due to the stabilizing effect of NTA? Would RNA interactions further stabilize the virus structure? Would 3DFlex analysis of a full virion containing RNA reveal an even further reduction in motion?*

We thank the reviewer for this thoughtful comment. The 3DFlex analysis for full RHDV virions was calculated and is shown in the revised manuscript as S2 Video. The swing angles for both empty and full T=3 capsids are similarly small (≲5°), indicating limited motion regardless of RNA presence. This suggests that the reduced flexibility of the T=3 capsid is primarily intrinsic to its structural configuration. However, NTA–RNA interactions might contribute further stabilization, which could help maintain the observed rigidity. These aspects are now discussed in the revised manuscript (l. 452-456): “3DFlex analysis of both empty and full T=3 capsids revealed similarly small swing angles (~5°), indicating limited protrusion mobility regardless of RNA presence. This reduced motion likely reflects intrinsic structural constraints of the T=3 capsid, although NTA–RNA interactions could contribute additional stabilization by anchoring the inner capsid layer to the packaged genome”.

*Given the observed swinging of the protrusions, the calculated angles of the S domain relative to the P domain shown in the Figure 3 B and C may need to be reconsidered. The ~30d and ~13d for A and B relative to the S domain in the RHDV reconstruction map are likely the averaged measurements of these angles.*

The referee is correct; this is stated explicitly in the main text (l. 212-213): “Given the swinging of the protrusions, these values are averaged measurements”.

*One computational approach could be to perform 3D classification using a larger box to extract local dimer regions. If the particles are aligned based on one end of the dimer (either the base or the tip), the 3D classification might reveal distinct classes with different swing angles.*

This approach was shown above in Figures 1 and 2.

***Part III – Minor Issues: Editorial and Data Presentation Modifications***

*1) The P domains adopt flexible conformations relative to the S domains, as shown in Movies S1 and S2. This flexibility limits the overall resolution when whole-particle images and icosahedral symmetry are used for reconstruction. Therefore, masks were applied for localized reconstructions to achieve higher resolutions (Figure 1G). Please indicate the size of the mask in a figure or in a supplementary figure.*

To achieve a high resolution map, the A/B spikes were extracted as subparticles included in a 97 Å diameter sphere without any mask. This is indicated in the legend of Fig 2C.

*2) The paper concludes that the NTA network and RNA interactions determines the structural polymorphisms of VP1 dimers in an icosahedral capsid – specifically, the relative angles between the S and P domains in molecule A, B and C. However, it is important to note that an even more significant factor may be the icosahedral packing of the molecules. Higher order molecular interactions likely play a critical role in driving the conformational variation of the protein domains.*

This is correct and we have included a new paragraph that reads (l. 484-487): “While RNA and NTA interactions influence the polymorphism observed, the icosahedral packing of the VP1 molecules might be an even more critical determinant, as higher-order molecular interactions are likely to be responsible for the conformational variation among protein domains”.

*3) For readers unfamiliar with RHDV research, the biological significance of N15 VLPs is not immediately clear. Does the FMDV T cell epitope (residues 21-35 of the nonstructural protein 3A) share sequence or biochemical similarity with the NTD of RHDV VP1? Please provide a clarification in the Introduction or Results section regarding N15 (i.e. the inserted T epitope in the N15 capsid). Additionally, would co-expression of this peptide affect infectivity of the virion?*

The chimeric construct N15, consisting of RHDV VLPs that harbor a FMDV-derived T-cell epitope incorporated at the VP1 N terminus, was generated as part of our ongoing research to develop RHDV-based engineered VLPs as a vaccine platform (refs: 34, 35 and 37). The inserted foreign epitope (residues 21-35 of the FMDV nonstructural protein 3A) shares no relevant sequence or biochemical similarity with the NTD of RHDV VP1. Regarding the potential effect of the foreign epitope insertion on infectivity of the resulting chimeric virion, both the lack of an efficient cell culture system for RHDV propagation and a functional reverse genetic system currently precludes performing such virological studies.

A new sentence has been added (l. 140-141): *“*…which assembled into chimeric VLPs displaying a foreign epitope to be used as a vaccine platform”.

*4) Line 425-426 states “the Δ29N capsid T=1 capsid would also be anticipated to be stiffer than the large VP1 T=3 capsid”. However, since the T=1 capsid lacks the NTA, wouldn’t this make the capsid softer, even though its smaller size might render a stiffer particle? In fact, the measured spring constant for the T=1 particle (0.172 ± 0.039 N/m) is smaller than those of the T=3 full and empty particles (0.433 ± 0.103 and 0.218 ± 0.046 N/m), suggesting that the T=1 particles are softer. So please clarify this statement (line 425-426).*

The paragraph has been re-written as follows (l. 472-477): *“*A small empty icosahedron, such as the Δ29N T=1 capsid, would be anticipated to show greater stiffness than the larger VP1 T=3 capsid, since both are assembled from nearly identical building blocks. Our results nevertheless indicate that the T=1 and T=3 particles show comparable softness; this suggests that the increased disorder at the VP1 N-terminus in the Δ29N capsid (in which the first ordered residue is Gly56) compensates for the difference in capsid size”.

*5) It will be very helpful to readers if the figures and movies included more labels. For example:*

*(a) Movie S3: please label the NTA, S and P1/P2 domains*

Done. It is now S4 Video.

*(b) Figure 1: please label the sample names in panels C-F.*

Done.

*(c) Line 248 states “The molecular switch responsible for VP1 structural polymorphism is located within the NTA in the first 29 N-terminal residues. These molecules are superimposed well”. Please label where the residue 29 is in Figure 5B.*

Done. Residue 29 is shown as a white sphere (Fig. 5B) or as a black sphere (S4 Fig).

*(d) Figure 6D-F: please clarify the meaning of the bar colors (yellow orange, green)*

This is clarified in the Fig. 6 legend, which now reads: “(**D**) Histogram showing the van der Waals/hydrophobic interactions (solid bars), hydrogen bonds (green dashed bars) and a salt bridge (grey dashed bar) formed by the A subunit NTA segment with its cognate subunit A and with the adjacent A_5_ and A_5`_ subunits (defined in panel A). Bar colors indicate the subunit that interacts with the A subunit NTA (orange for subunit A_5,_ yellow for its cognate subunit A, and green for subunit A_5`_). Asterisk color indicates the subunit with which hydrogen bonds and salt bridges are established. (**E)** Histogram showing the interactions formed by the B subunit NTA segment with its cognate subunit (bright blue) and the adjacent subunits C_3_ (pink) and C_3`_ (purple), as for panel D. (**F**) Histogram showing the interactions formed by the C subunit NTA segment with its cognate subunit (purple) and the adjacent subunits B_3_ (bright blue) and B_3`_ (dark blue), as for panel D”.

Point-by-point reply to Reviewer #2

***Part I - Summary***

*This study investigated the cryo-EM structures of the Rabbit hemorrhagic disease virus (RHDV) virion and three recombinant RHVD virus-like particles with differing N-termini. The focus of this study was on the role of the N-terminal arm (NTA) in virus assembly, virus rigidity, and interaction with the viral RNA genome. Comparison of the structures revealed high similarity of T=3 icosahedral capsid in the RHDV virion and the full-length virus-like particles, whereas the delta29 capsid lacking the N-terminal 29 amino acids formed T=1 capsids, indicating the importance of the NTA in virus assembly. Evaluation of the N15 capsid containing a FMDV-derived T-cell epitope enabled visualization of the epitope inside the capsid in some cases. The NTA structures differed in A/B/C subunits and provided a molecular basis for their roles in promoting A/B and C/C dimers, and subsequently pentamers and hexamers. Additionally, the RHVD virion electron density maps showed electron density around the ordered N-terminal ends (Ala20) at the 3-fold axis, which the authors attribute to interacting viral RNA genome. Finally, atomic force microscopy (AFM) studies reveal that full virions are more rigid than empty T=3 capsid, which the authors conclude is a “mechanical role” for genomic RNA in RHVD virions.*

*Overall, this work is consistent with and forms many similar conclusions on the NTA as the previous study on the structures of RHVD2 virions and virus-like particles (Ref. 15). A difference of this work is that it concludes that the extra electron density near the 3-fold NTEs is viral genomic RNA whereas the previous study concluded that this density is from the remaining disordered NTA amino acids 1-19. Also, a novelty of this study is the AFM work that reveals that the genomic RNA contributes to viral rigidity.*

***Part II – Major Issues: Key Experiments Required for Acceptance***

*1. The conclusion that the extra electron density near the 3-fold NTEs is viral genomic RNA is not clearly supported. It appears possible that the electron density is from amino acids 1-19, as was concluded in Ref. 15. 2. The authors should compare their electron density maps from the RHVD virion and the empty N15 and VP1 capsid structures in Figure 7 to support their conclusion that this density is only in the RHVD virion, supporting it is viral RNA. The authors should also comment as to why they conclude it is viral RNA and not the NTA, 3. and why their data/conclusion differs from Ref. 15.*

1. We thank the reviewer for this insight. The reviewer refers to the study by Ruan et al. (J. Virol **98**:e0127524, 2024; cited in our manuscript as Ref. 15). The electron density near the threefold axis in the capsid of mature virions and VLPs mentioned by the reviewer responds to an uninterpreted density, as stated by the authors themselves (their Fig. S7). As this feature lies close to the modeled N termini of the B and C subunits, whose first ordered residue is Gly24, Ruan et al. suggested the density could correspond to the unresolved portions of the NTAs of these subunits. A similar density was also observed in their VLPs.

In our 3D map of RHDV virions, the first ordered residue of B and C subunits is Ala20, located at the tip of stalactite-like densities formed by the ordered N-terminal arms of the B and C subunits (residues Ala20-Gly24) (see new S7A Fig, arrows). These six arms are positioned at a ~15 Å radius from the threefold axis at the center of the hexamer (S7B Fig, solid circle). In our reconstructions, we also observed a comparable, poorly defined density near the threefold axis, but it was not possible to model additional NTA residues because no continuous density connects this feature with Ala20. The small protrusions forming this “jellyfish-like” density are located at a radius of ~11 Å relative to the three-fold axis (S7B Fig, dashed circle), *i.e*., they are surrounded by the six NTAs of the B and C subunits. This arrangement can be visualized clearly from the virion interior. We also include a new supplementary figure (requested by another reviewer; S6 Fig) that shows two sections of the full RHDV 3DR, in which the NTA-mediated connections (S6B Fig) and the jellyfish-like densities (S6D Fig) are shown and clearly correspond to different structures.

As we lacked further experimental evidence, we did not comment on this undefined density in the previous version of the manuscript. Following the reviewer’s suggestion, however, we now include a clarifying sentence (l. 305-307): “The six NTAs of the B and C subunits surround a poorly defined, jellyfish-like density located at the threefold axis (S7 Fig, orange), similar to that previously described in the RHDV G.II system [15]”.

In new figures 7B and S7, the jellyfish-like density is highlighted. Note that the new version of Fig 7B is not a section of the icosahedral map but a section of an asymmetric map obtained after symmetry relaxation (as requested by another reviewer), in which the six NTA-mediated connections are well-resolved (the map is contoured at 1σ above the mean density).

2. Following the reviewer’s recommendation to demonstrate that the internal density observed in the RHVD virion corresponds to viral RNA*,* we calculated spherically averaged radial density profiles from the 3DRs of full and empty RHDV particles (Fig 7A). These profiles were nearly superimposable at the protein shell (radius 113-202 Å). The full RHDV virions nonetheless displayed an additional internal density at a ≲113 Å radius (Fig 7A, horizontal arrows), which we ascribe to the packaged RNA genome. New Fig. 7A includes the radial density profiles as well as the difference map obtained by subtracting the empty capsid from the full capsid reconstruction. The RHDV genome appears as (at least) two concentric shells of density (Fig. 7A, vertical arrows), consistent with observations from other cryo-EM structures of ssRNA viruses [Toropova et al., 2008, J. Mol. Biol. 375:824; Bakker et al., 2014, J. Virol. 88:6093; Hesketh et al., 2015, Nat. Commun. 6:10113]. These references have been added to the revised manuscript (refs 44-46).

An explanatory paragraph has now been included (l. 291-299): “Radial density profiles of the 3DRs for full and empty RHDV particles were nearly superimposable at the protein shell (r = 113-202 Å) (Fig 7A). Full particles contain an additional density inside the capsid (r ≲ 113 Å) that corresponds to the packaged RNA genome (Fig 7A, orange line). The RNA density is lower than that of the maximum of the protein shell; although the innermost RNA shell appears diffuse, two outer layers are discernible (Fig 7A, arrows), as reported for other ssRNA viruses [44-46]. In the empty capsid, the internal density matched that of the surrounding solvent (Fig 7A, green line). The calculated difference map (full subtracted from empty capsid) highlighted the genome densities (Fig 7A, dashed line; S5 Fig).”

We also added a supplementary figure (S5 Fig) showing the radial density profiles of both sharpened and unsharpened 3DRs for full and empty RHDV particles (S5A Fig), together with their corresponding central sections (S5B Fig). The B-factor correction, applied to sharpen the map and reveal high-resolution features, acts as a strong high-pass Fourier filter, which attenuates low-resolution features such as the diffuse genomic RNA. This effect is clearly visible when sharpened and unsharpened central sections of the full map are compared. Nevertheless, the internal density corresponding to the packaged RNA genome is evident in the radial density profiles derived from unsharpened (S5A Fig, thick yellow lines) and sharpened (S5A Fig, thin yellow lines) 3D maps of full particles. Unsharpened maps are also included in all depositions in the EM Data Bank.

As requested by the reviewer, we have also included the radial density profiles from the VP1 and empty RHDV 3D reconstructions (S5C Fig), showing that both profiles are superimposable at the protein shell.

We suspect that part of this confusion arose from our previous representation of the maps and sections in the original Fig. 1, now improved. First, we had not indicated the contour thresholds used for the isosurface representations. In the revised figure, the contour level for the RHDV-related maps in Fig. 1H–K (upper row) is set at 2σ above the mean, whereas that for the sections in Fig. 1H–L (lower row) is 1.2σ above the mean. These values are now specified in the figure legend. Second, as stated in the original legend, the “density corresponding to the genome of full virions was removed computationally” not because it was absent, but because no structural details were discernible at that contour level. In the updated Fig. 1, we have retained the density for the viral RNA. We apologize for this earlier inconsistency.

In addition, to highlight the RNA density, we also prepared a new supplementary figure (S1 Fig) comparing the central sections from the 3DR (left half) with 8 Å–thick slabs (right half) contoured at 1.2 σ above the mean density. Brighter shading indicates higher density. A cross-section through the 3D map is considered the objective gold standard for cryo-EM maps, as it is not exposed to subjective thresholding; a density section shows structural details that cannot be appreciated from simple isosurface renderings. The density inside the capsid corresponding to packaged RNA, visible in the full capsids (S1 Fig), is nonetheless lower than that of the protein shell. For this reason, different contour levels must be used to visualize RNA density appropriately. To clarify this point, the Fig. 1 legend has been extended to read: “8 Å-thick slabs contoured at 1.2σ above the mean density to highlight the RNA density”.

3. Differences between our results and those reported by Ruan et al. (Ref. 15) could arise from several factors, including the use of different RHDV strains. For instance, while the RHDV GI.2 strain analyzed by Ruan et al. shows both “up” and “down” conformations of the P domain, these states were not observed in the RHDV GI.1 strain used in our study. Another source of variation could be differences in purification protocols, which can yield particles with distinct levels of structural preservation. Such differences could influence local resolution in the reconstructed capsid, and consequently, the extent of the ordered regions. In our atomic models, the VP1 subunits begin at residues 20 (for subunits B and C) and 30 (for subunit A), whereas in the work by Ruan et al., these subunits start at residues 24 (B and C) and 41 (A).

We believe many of our findings are consistent with those reported by Ruan et al., while others provide novel or complementary structural insights. These aspects are discussed in detail throughout the revised manuscript.

***Part III – Minor Issues: Editorial and Data Presentation Modifications***

*1. Line 122: Incomplete sentence: “…information regarding the spatial organization of the ssRNA within these virions and its interactions with the capsid.” Likely should end with “is lacking.”*

Corrected.

*2. Many figures would benefit from labels for panels, so the reader doesn’t have to go through the legends (i.e. Fig. 1H-K, Fig. 4A (add legend for the 2 colors), and Fig. 8).*

Done. New figures/parts are similarly labeled.

*3. Fig. 6DEF legend needs to be clearer in describing the figures, and these figures should be referred to in the text.*

Colors of subunits A_5_, A, and A_5’_ in part A are shown now in orange, yellow and green to match the colors in part D. We also used different colored asterisks to indicate the subunit that establishes hydrogen bonds (and a salt bridge) with the NTAs.

The legend is now clarified in the new Fig. 6 legend, which reads: “(**D**) Histogram showing the van der Waals/hydrophobic interactions (solid bars), hydrogen bonds (green dashed bars) and a salt bridge (grey dashed bar) formed by the A subunit NTA segment with its cognate subunit A and with the adjacent A_5_ and A_5`_ subunits (as in panel A). Bar colors indicate the subunit that interacts with the A subunit NTA (orange for subunit A_5,_ yellow for its cognate subunit A, and green for subunit A_5`_). Asterisk color indicates the subunit with which hydrogen bonds and salt bridges are established. (**E)** Histogram showing the interactions formed by the B subunit NTA segment with its cognate subunit (bright blue) and with adjacent subunits C_3_ (pink) and C_3`_ (purple), as for panel D. (**F**) Histogram showing the interactions formed by the C subunit NTA segment with its cognate subunit (purple) and with adjacent subunits B_3_ (bright blue) and B_3`_ (dark blue), as for panel D”.

*4. Lines 307-328: In most cases, there should be 1 significant digit in the standard deviations, and the last significant digit in the reported mean should align with the standard deviation (i.e. 0.214 +/- 0.037 should be reported as 0.21 +/- 0.04).*

Done.

*5. Line 320: Should be Fig. 1C, not 1G.*

Corrected.

Point-by-point reply to Reviewer #3

***Part I - Summary***

*The manuscript “Calicivirus assembly and stability are mediated by the N-terminal domain of the capsid protein with the involvement of the viral genome” by Novoa et al reports high-resolution cryo-EM structures (2.5–3.3 Å) of rabbit hemorrhagic disease virus (RHDV) virions along with a set of related virus-like particles (VLPs), combined with atomic force microscopy to probe the mechanical properties of capsids. The key finding is that the N-terminal arm (NTA) of the capsid protein VP1 does not function in isolation, but rather requires interaction with the viral RNA genome to stabilize specific conformations of VP1 dimers. These RNA–NTA contacts act as a “molecular co-switch” that guides T=3 assembly and strengthens capsid rigidity. The study nicely integrates structural and biophysical approaches and provides a new way of thinking about how the genome itself contributes to assembly and stability. Some strengths include: 1) the cryo-EM structures of authentic virions at near-atomic resolution are a significant technical achievement, allowing the authors to visualize NTA–RNA contacts directly; 2) the combination with AFM is clever and powerful, showing that RNA packaging makes virions mechanically stiffer than empty shells and thus adding a functional dimension to the structural observations; 3) the conceptual advance is important: while many calicivirus structures exist, this is the first to demonstrate that the genome actively shapes NTA conformations and assembly, rather than being a passive passenger; and 4) he paper is also placed thoughtfully in the broader calicivirus context, with comparisons to noroviruses, vesiviruses, and sapoviruses. Despite the overall strength of the manuscript, it could be improved by addressing a few issues listed below:*

***Part II – Major Issues: Key Experiments Required for Acceptance***

*How specific are the RNA–NTA interactions: The data clearly show RNA density contacting the NTAs, could the authors comment as to whether these are sequence-specific interactions or simply electrostatic. A bit more discussion would help here.*

We appreciate this comment and concur that we did not comment sufficiently on NTA interactions, partly due to the limited experimental information currently available. As there is no reverse-genetics system for RHDV, testing the predictions derived from our structural findings would constitute an entirely new project. We have nonetheless already initiated analysis of the NTA region by introducing single and deletion mutations and examining the resulting VLPs. Preliminary structural analyses of these apparently empty VLPs, in which the only nucleic acids available are from the host, suggest that electrostatic interactions have a major role in modulating capsid stability.

We had previously highlighted the relevance of electrostatics in the original manuscript [(l. 421-424): “The N-terminal end of VP1 contains only two proximal basic residues, Lys4 and Arg6, which are probably involved in interactions with the viral genome; this attractive interaction could be negatively modulated by two nearby acidic residues, Glu2 and Glu12”. To address the reviewer’s suggestion, we have expanded the discussion to include the possibility of sequence-specific contributions, as follows (l. 424–425): “Further studies are needed to assess the potential contribution of sequence-specific interactions in addition to electrostatics”.

*How general is this mechanism: The authors compare across caliciviruses, which is very helpful. But most of the human calicivirus structures come from empty VLPs, so it is hard to know whether RNA plays a similar co-switch role there. It would be useful if the authors more explicitly noted these limits, while still arguing for likely conservation.*

We agree that the proposed mechanism should be interpreted with caution, as most available human calicivirus structures correspond to empty VLPs, making it difficult to evaluate whether the genomic RNA exerts a similar co-switching function. To address this point, we have moderated and clarified our discussion as follows (l. 406–409): “Our findings emphasize the importance of the genomic RNA; however, since most human calicivirus structures are derived from empty VLPs, further studies are needed to determine whether RNA fulfills a similar co-switching role”.

*Functional assays: The paper is entirely structural and biophysical. This is understandable given that RHDV can’t be propagated in cell culture, but it would strengthen the manuscript if the authors directly acknowledged this limitation, so readers don’t over-interpret the structural results as functional proof.*

We thank the reviewer for this insight. We agree that, since RHDV cannot be propagated in cell culture, our study is limited to structural and biophysical observations. We have added a sentence in the Discussion to explicitly acknowledge this and to clarify that our conclusions are not intended as direct functional proof. The new sentence reads (l. 370-373): “As RHDV cannot be propagated in cell culture, the present study is restricted to structural and biophysical analyses. Our interpretations should thus not be regarded as direct functional evidence, but rather as structural insights that may guide future functional studies.

***Part III – Minor Issues: Editorial and Data Presentation Modifications***

*The comparisons to HIV and MS2 are interesting but might mislead non-expert readers into thinking these are mechanistic parallels. It would help to clarify that these are only conceptual analogies.*

We agree that the analogies to HIV and MS2 are conceptual rather than mechanistic. To make this clear and to avoid potential misinterpretation, we have revised the text as follows (l. 442–447): “This assembly pathway is conceptually reminiscent of packaging signal-mediated assembly of the T=3 MS2 bacteriophage [50]. Likewise, as observed in HIV assembly [51], interference with intermediate assemblies through VP1-genome interactions (or the NTA network promoted by the genome) might provide a conceptual framework for exploring antiviral strategies against human caliciviruses such as norovirus and sapovirus”.

*Try to be consistent with terminology: the manuscript flips between “molecular switch” and “co-switch.”*

The referee’s observation is opportune. We now used the term “molecular co-switch” for the genomic RNA throughout the manuscript.

*The supplementary movies (S1–S6) could be described in more detail in the text so the reader knows what they’re meant to illustrate.*

Done.

Point-by-point reply to Reviewer #4

***Part I - Summary***

*Authors describe 2.5-3.5A cryo-EM structure of RHDV mature virion, empty virion particles, recombinant VLP assembled from VP1, and recombinant VLPs assembled from a N-terminal truncation. In addition, the authors have also performed AFM studies to investigate whether capsid stability is affected by the presence of RNA by comparing full and empty virions and VLPs. Overall, from these studies, the authors suggest that interactions between the NTA and genomic RNA plays a guiding role in conferring proper conformational states of VP1 dimers and the capsid assembly.*

*Although the structural analysis is well-performed and the manuscript is well-written, including all relevant methodological details, and appropriate illustrations and supplementary material.*

*However, in my opinion, this study falls short in terms of significance and general interest. It is perhaps more suited for a specialized journal. The cryo-EM structures of the mature G1.2 RHDV virion, VLP assembled from the full-length VP60, and VLP assembled from the NTA-truncated VP60 of RHDV GI.2 (at resolutions of 2.5 Å, 2.5 Å, and 2.4 Å), respectively, have already been published. I appreciate that the structural analysis in this manuscript focuses on G1.1 RHDV, which has not been published before.*

*Most of the inferences about the network of NTA in RHDV, as well as other members of the Caliciviridae, have been thoroughly examined and visualized either by crystallography or high-resolution cryo-EM. One unique aspect of this manuscript is the supposed visualization of the effect of genomic RNA on the differential conformation of the NTAs and their interaction with the neighboring subunits.*

*But this aspect is not convincing at all. 1 It is unclear whether the density supposedly attributed to RNA is indeed that of RNA, particularly considering that the density is visualized in an unprocessed map at a 0.6 sigma level. That level borders on the noise; it could be noise emanating from the local breakdown of icosahedral symmetry, or perhaps even due to VP2, which, in fact, has been shown to contribute to the stability of the capsid in norovirus VLP. 2 A stronger validation of this aspect would have garnered more enthusiasm; this perhaps requires performing symmetry relaxation and systematic focused refinements. 3 AFM studies suggest that genomic RNA may play a critical role in capsid assembly and stability; however, this observation alone is insufficient and non-confirmatory, as it is equally possible that VP2 (alone or in combination with genomic RNA) also plays a role.*

1. This criticism coincides in part with a similar request by another reviewer, to which we respond as follows:

“To demonstrate that the internal density observed in the RHVD virion corresponds to viral RNA*,* we calculated spherically averaged radial density profiles from the 3DRs of full and empty RHDV particles (Fig 7A). These profiles were nearly superimposable at the protein shell (radius 113-202 Å). The full RHDV virions nonetheless displayed an additional internal density at ≲113 Å radius (Fig 7A, horizontal arrows), which we ascribe to the packaged RNA genome. New Fig. 7A includes the radial density profiles as well as the difference map obtained by subtracting the empty capsid from the full capsid reconstruction. The RHDV genome appears as (at least) two concentric shells of density (Fig. 7A, vertical arrows), consistent with observations from other cryo-EM structures of ssRNA viruses [Toropova et al., 2008, J. Mol. Biol. 375:824; Bakker et al., 2014, J. Virol. 88:6093; Hesketh et al., 2015, Nat. Commun. 6:10113]. These references have been added to the revised manuscript (refs 44-46).

The following explanatory paragraph has been included (l. 291-299): “Radial density profiles of the 3DRs for full and empty RHDV particles were nearly superimposable at the protein shell (r = 113-202 Å) (Fig 7A). Full particles contain an additional density inside the capsid (r ≲ 113 Å) that corresponds to the packaged RNA genome (Fig 7A, orange line). The RNA density is lower than that of the maximum of the protein shell and, although the innermost RNA shell appears diffuse, two outer layers are discernible (Fig 7A, arrows), as reported for other ssRNA viruses [44-46]. In the empty capsid, the internal density matched that of the surrounding solvent (Fig 7A, green line). The calculated difference map (full subtracted from empty capsid) highlighted the genome densities (Fig 7A, dashed line; S5 Fig)”.

We also added a supplementary figure (S5 Fig) showing the radial density profiles of both sharpened and unsharpened 3DRs for full and empty RHDV particles (S5A Fig), together with their corresponding central sections (S5B Fig). The B-factor correction applied to sharpen the map and reveal high-resolution features acts as a strong high-pass Fourier filter, which attenuates low-resolution features such as the diffuse genomic RNA. This effect is clearly visible when sharpened and unsharpened central sections of the full map are compared. Nevertheless, the internal density corresponding to the packaged RNA genome is evident in the radial density profiles derived from both unsharpened (S5A Fig, thick yellow lines) and sharpened (S5A Fig, thin yellow lines) 3D maps of full particles. Unsharpened maps are also included in all depositions in the EM Data Bank.

As requested by the reviewer, we have also included the radial density profiles from the VP1 and empty RHDV 3D reconstructions (S5C Fig), showing that both profiles are superimposable at the protein shell.

We suspect that part of this confusion arose from our previous representation of the maps and sections in the original Fig. 1, now improved. First, we had not indicated the contour thresholds used for the isosurface representations. In the revised figure, the contour level for the RHDV-related maps in Fig. 1H–K (upper row) is set at 2σ above the mean, whereas that for the sections in Fig. 1H–L (lower row) is 1.2σ above the mean. These values are now specified in the figure legend. Second, as stated in the original legend, the “density corresponding to the genome of full virions was removed computationally” not because it was absent, but because no structural details were discernible at that contour level. In the updated Fig. 1, we have retained the density corresponding to the viral ssRNA. We apologize for this earlier inconsistency.

In addition, we have prepared a new supplementary figure (S1 Fig) to show the comparison of the central sections from the 3DR (left half) with 8 Å–thick slabs (right half) contoured at 1.2 σ above the mean density to highlight the RNA density. Brighter shading indicates higher density. A cross-section through the 3D map is considered the objective gold standard for cryo-EM maps, as it is not exposed to subjective thresholding; a density section shows structural details that cannot be appreciated from simple isosurface renderings. The density inside the capsid corresponding to packaged RNA, visible in the full capsids (S1 Fig), is nonetheless lower than that of the protein shell. For this reason, different contour levels must be used to visualize RNA density appropriately. To clarify this point, the legend of Fig. 1 has been extended to read: “8 Å-thick slabs contoured at 1.2 σ above the mean density to highlight the RNA density”.”

We respectfully disagree with the reviewer’s suggestion that VP2, which is present in the virion at a low copy number, could be visualized in 3D reconstructions where icosahedral symmetry has been imposed. Any asymmetric distribution or density corresponding to VP2 would be averaged out under these symmetry constraints. The structure formed by VP2 has only been resolved in feline calicivirus (FCV) capsids labeled with a soluble fragment of its cellular receptor (fJAM-A), using a focused classification approach similar to the localized reconstructions we employed to resolve the VP1 P-domain structure. In that study [Conley et al. (2019), Nature 565:377], 12 copies of VP2 form a portal-like assembly; however, the structure of VP2 remains unresolved in undecorated FCV capsids.

2. We appreciate the reviewer’s careful evaluation and acknowledge the concern regarding the interpretation of the low-sigma density as genomic RNA. As s/he correctly notes, the intensity of the connections between the inner capsid surface and the RNA outer layer depends on the selected contour level. In our analysis, these connections are preserved even after imposing icosahedral symmetry to calculate the 3DR. If such connections were not present at equivalent positions within the asymmetric unit, they would have been smeared out after icosahedral averaging.

The evidence supporting genuine protein-RNA interactions is based on electron density maps rather than on thresholded surface-shaded renditions. These connections are not buried in the noise, as the RNA-protein connections indeed conform local icosahedral symmetry. The connections are directly visible in a section of the full RHDV 3DR, now included as S6 Fig in the revised version (the jellyfish-like density is also shown as requested by another reviewer). We selected a 0.6σ contour level to visualize direct contacts between the last visible residue of the NTAs and the outer layer of the genome inside the virions, but this is certainly a subjective thresholding. A higher contour level would indicate protein-RNA interactions mediated by the NTAs of B and C subunits. To clarify this point, the previous Fig 7A has been moved to a new supplementary figure (S7 Fig), which now shows the unsharpened, icosahedral map of the RHDV full capsid around the threefold axis to highlight the uninterpreted, jellyfish-like density at the threefold axis (requested by another reviewer).

In addition, following the reviewer’s suggestion, we reanalyzed the full RHDV data. For this, we performed a symmetry relaxation and systematic focused refinements of the hexameric regions, which were extracted as independent subparticles to resolve structural heterogeneity around the threefold axes of symmetry. This analysis revealed deviations from icosahedral symmetry and yielded multiple reconstructions showing variable connectivity densities at the threefold axes, indicative of partial or complete connections in subsets (or classes) of particles. Among these, we isolated two subsets of hexameric arrangements with six well-resolved NTA-mediated connections that, when contoured at 1 σ above the mean density, bridge the outer surface of the outermost RNA layer with the inner surface of the protein shell. These two subsets (representing 25% of total hexamers), together with two additional subsets showing five well-resolved connections, accounted for ~62% of the hexamers in the 3D classification across six classes. Comparable results were obtained when four or eight classes were computed.

These results are presented in two new figures: Fig. 7B, which shows a section of the refined map (without symmetry) by merging the four classes with six and five NTA-mediated connections, and S8 Fig, which displays the six 3D class averages (without symmetry) of the region surrounding the threefold axis of the full RHDV capsid. A new paragraph has been added to the “RNA-protein interactions in the RHDV virions” section (l. 308-315): **“**We reanalyzed the full RHDV data using 3D classification with symmetry relaxation and systematic focused refinements of the hexameric regions, extracted as independent subparticles, to resolve structural heterogeneity at the threefold axes of symmetry. This analysis yielded cryo-EM maps with variations in the number of NTA-mediated connections, indicating that they are flexible and/or transient (S8 Fig). This approach allowed us to isolate hexameric arrangements with six well-resolved connections that bridge the outer surface of the outermost layer of the genome with the inner surface of the protein shell, which made up 62% of the hexamers included in this analysis (Fig. 7B)”.

This methodology is now also described in the “Image processing” section (l. 593-598): “To identify and classify different arrangements of the hexameric regions in full RHDV particles, local areas corresponding to these regions were extracted from the original images and treated as subparticles, and the C3 symmetry was relaxed. Six classes were reconstructed without applying any symmetry and subjected to focused refinements using a truncated conical mask encompassing the region that includes the NTA-mediated connections. The atomic coordinates derived from the icosahedral map were then fitted with Phenix [65]”.

We thank the reviewer for this excellent suggestion, which notably improved the structural analysis and interpretation.

3. We thank the reviewer for this valuable comment. We fully agree that the AFM observations alone cannot unambiguously distinguish between the respective contributions of genomic RNA and VP2 to capsid stability. We have revised the text to clarify that, while our data are consistent with a role of genomic RNA in stabilizing the capsid, the participation of VP2 –either alone or in combination with RNA– cannot be excluded. This clarification has been added to the Discussion (l. 460-462): “Although AFM data suggest that genomic RNA contributes to the stability of the RHDV capsid, we cannot exclude the possibility that VP2, alone or together with the genomic RNA, also participates in this process”.

*Minor:*

*Reference citations in several places could be improved by providing references in chronological order, based on when a particular structural observation was first made and to which caliciviruses it pertains.*

Corrected. We apologize for this inconsistency.

***Part II – Major Issues: Key Experiments Required for Acceptance***

*As discussed above*

***Part III – Minor Issues: Editorial and Data Presentation Modifications***

*Overall okay*
